# Azobenzene‐Functionalized Ionic Liquids: Light‐Responsive Surfactants With Catalytic Potential

**DOI:** 10.1002/open.70203

**Published:** 2026-04-20

**Authors:** Markus Hegelmann, Stefan Frei, Julian Zuber, Johannes Luibl, Wolfgang Korth, Andreas Jess, Peter Coburger, Mirza Cokoja

**Affiliations:** ^1^ Technical University of Munich Department of Chemistry Catalysis Research Center and School of Natural Sciences Garching bei München Germany; ^2^ Technical University of Munich Department of Chemistry School of Natural Sciences Garching bei München Germany; ^3^ Faculty of Engineering Science Chair of Chemical Engineering University of Bayreuth Bayreuth Germany

**Keywords:** ionic liquid, isomerization, light‐responsive, phase transfer catalysis, switchable surfactants

## Abstract

We report on the synthesis of surface‐active azobenzene‐based surface‐active ionic liquids (AzoSAILs) with tunable and photoswitchable physicochemical properties. The reversible isomerization between the *trans*‐ and the *cis*‐form was studied in different solvents and concentration by spectroscopic methods. The potential of ionic AzoSAILs as light‐controllable epoxidation catalysts was studied regarding their catalytic activity and switchable micelle formation in aqueous media. Zwitterionic AzoSAILs were investigated regarding their unprecedented light‐controlled solubility, surface‐activity in aqueous media and potential application in biphasic epoxidations. Additional valuable insight into the photoisomerizations is provided by computational studies.

## Graphical abstract

Surface‐active imidazolium‐based ionic liquids containing an azobenzene group exhibit different micellization properties upon photoisomerization. Applying a sulfonate tag to the azobenzene moiety profoundly affects the physicochemical behavior of both isomers in water. While the *cis*‐isomer forms micellar aggregates, the *trans*‐isomer is insoluble and precipitates. Such a behavior bears a perspective for the recycling of molecular catalysts attached to such surfactants.

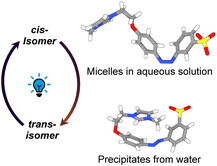

## 1 Introduction

Although catalysts are central to most industrial processes, the majority are heterogeneous—primarily due to the inherent challenges in recycling homogeneous catalysts, and the hazardous nature of common oxidants or solvents, which limit large scale applications working in closed chemical cycles [1]. In the last decades, significant efforts have been directed toward developing strategies that enable efficient recovery and reuse of molecular catalysts. Biphasic liquid–liquid systems, which exploit differential partitioning of the catalytic species and organic substrate across immiscible phases, have emerged as promising solutions [2]. This concept was successfully implemented in industrial hydroformylations [3], C—C cross couplings [4], and hydrogenation reactions [5] by introducing highly water soluble ligands to the transition metal catalysts.

However, similar systems for oxidation catalysis—and in particular epoxidations—are lacking, despite the high demand for such products [6]. Biphasic epoxidations typically rely on hydrogen peroxide (H_2_O_2_) as a green oxidant and utilize phase‐transfer catalysts (PTCs) [7]. Most reported systems involve highly water‐soluble polyoxometalates (POMs), which are shuttled into the organic phase by external PTCs, rendering catalyst recycling tedious [8]. In contrast, epoxidations in aqueous media, facilitated by the transfer of olefins via surfactants, offer greater potential for sustainable catalysis systems [7]. Imidazolium‐based surface‐active ionic liquids (SAILs) have shown remarkable versatility as multifunctional epoxidation catalysts: they solubilize olefins in water, enable efficient catalysis, and remain immiscible with the organic product phase, allowing for phase separation and reuse [9, 10, 11, 12]. Yet, unlike industrial biphasic systems where aqueous catalyst phases can be directly recycled, micellar epoxidation systems face challenges such as water accumulation and catalyst dilution, due to the consumption of aqueous H_2_O_2_. Over successive cycles, the catalyst concentration falls below the critical micelle concentration (CMC), eventually leading to disaggregation of micelles and declining catalytic activity [13].

To address these limitations, precise control over micellization and solubility of the catalyst in the reaction phases is essential. Stimuli‐responsive surfactants that reversibly aggregate/disaggregate, change phases, or precipitate in response to external stimuli (e.g., pH, temperature, light) have been confined to biochemical applications [14, 15, 16]. Previously, we demonstrated that fluorinated SAILs (FSAILs) exhibit reversible, temperature‐controlled solubility in water. However, the increased polarity of the epoxidation product leads to catalyst migration into the organic phase, hampering recyclability [17].

The structural tunability of imidazolium‐ and ammonium‐based ionic liquids offers vast potential for engineering stimuli‐responsive phase behavior, solubility, and ultimately, catalytic activity [18]. Among these, azobenzene‐based photoresponsive surfactants are particularly attractive: their *trans*‐isomer is surface‐active, while under analogous conditions the *cis*‐form is not, enabling light‐triggered changes in interfacial properties [19, 20, 21, 22]. Yet, the integration of such systems into catalytic applications remains scarce. Only few reports explore light‐controlled micellization to regulate catalytic activity, with few notable examples, such as POMs encapsulated by photoresponsive surfactants for light‐controlled phase transfer [23]. Other systems, such as shell cross‐linked micelles focus on targeted substrate selectivity in transfer hydrogenations [24], or activity modulation in hydrolysis [25], but do not address recyclability of the catalysts

In this report, we aim to control the catalytic activity in biphasic epoxidations and facilitate the recycling of catalysts from aqueous media. Azobenzene‐functionalized surface‐active ionic liquids (AzoSAILs) suitable for such reactions must be highly soluble in water, immiscible with organic substrates and products, and possess a sufficiently high CMC to support efficient catalysis. Ideally, they should also exhibit a large difference in CMC (ΔCMC) between the *trans* and *cis* photoisomers. To meet these criteria, we investigate both ionic and zwitterionic AzoSAIL derivatives, as these structural classes are expected to significantly influence the physicochemical properties of the surfactants. Although zwitterionic AzoSAILs do not inherently contain catalytically active anions, catalytic functionality can be introduced by the addition of suitable metal salts [26]. Based on this rationale, we pursued two design strategies: (a) modifying conventional ionic surfactants by incorporating azobenzene‐functionalized imidazolium cations paired with various counterions and (b) developing zwitterionic surfactants with oppositely charged headgroups. The investigated structural motifs comprise three types of ionic surfactants: (i) [R^1^AzoOR^2^ImR^3^][X] (R^1^ = H, Bu; R^2^ = OC_2_H_4_, OC_6_H_12_; R^3^ = Me, Oct; X = ReO_4_^−^, WO_4_^2−^) (ii) [AzoCH_2_ImMe]_2_[WO_4_], (iii) [PhAzoImMe_2_][X] (X = WO_4_^2−^, NO_3_^−^), and three zwitterionic surfactants with varying substitution patterns: [RAzoOCH_2_ImMe] (R = *m*‐SO_3_, *o*‐SO_3_, *p*‐SO_3_).The reversible *trans*/*cis* isomerization of the azobenzene units was investigated, particularly in relation to its effects on solubility and micelle formation. Additionally, the impact of different substitution patterns on phase transfer behavior, catalytic efficiency, and recyclability is discussed. The experimental results were supported by quantum mechanical calculations regarding solvent interactions and photo isomerization mechanisms.

## 2 Results and Discussion

### 2.1 Synthesis and Characterization of AzoSAILs

The synthesis of bromide‐AzoSAILs (with cations **1**–**8**) in which the chromophoric azobenzene unit is separated from the imidazolium head group with ethoxy spacers (–OC_2_H_4_–), follows a three‐step procedure. In the first step, aniline derivatives and phenol undergo diazotization, followed by a Williamson ether synthesis at the hydroxide group using a dihalidealkane [20]. Finally, a 1‐alkylimidazolium unit is introduced in an alkylation reaction (see Scheme S1, Supporting Information) [17].

**SCHEME 1:**
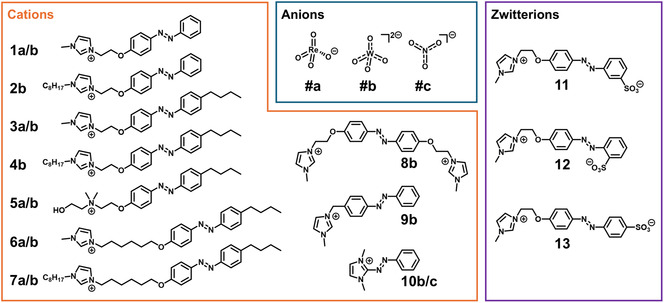
Overview of the cations and corresponding anions of AzoSAILs (**1**–**10**) as well as zwitterionic AzoSAILs (**11**–**13**).

The bromide‐AzoSAIL (**9**), in which the imidazolium moiety is linked to the azobenzene core via a methylene bridge, is prepared via a four‐step synthesis starting with the diazotation of 4‐aminobenzoic acid (Scheme S2, Supporting Information). The diazonium compound is subsequently reduced with lithium aluminum hydride to afford the corresponding alcohol, which is then converted to the bromide using a modified Appel‐type bromination (Scheme S3, Supporting Information) [27]. The bromide‐AzoSAIL was obtained by an analogous alkylation reaction [17]. For iodide‐AzoSAIL **10**, which features a significantly smaller organic moiety and thus is expected to exhibit altered solubility and micellization behavior, a two‐step route is used. This involves the diazotation of aniline and imidazole, which followed up by an alkylation with methyl iodide [28]. In all cases, AzoSAILs with halide counterions were subjected to ion exchange over a strongly basic resin to obtain the corresponding hydroxide forms. Subsequently, the basic solutions were reacted with ammonium perrhenate, tungstic acid or diluted nitric acid to obtain AzoSAILs with [ReO_4_]^−^, [WO_4_]^2−^, or [NO_3_]^−^ counterions [11, 17]. Zwitterionic AzoSAILs (**11–** **13**) were synthesized using sulfonic acid derivatives of aniline, following a similar procedure to that of AzoSAILs **1**–**8** (Scheme S4, Supporting Information). After alkylation with methylimidazole, halide contaminations are removed by elution of the AzoSAIL solution over the basic ion exchange resin. Note that the synthesis of sulfonated derivatives of **1–** **8** with a metal oxo anion were unsuccessful, presumably due to the high stability of the zwitterionic species. This most likely results from the possible protonation of imidazole by the acidic conditions and the lower reactivity of imidazole as a coupling partner compared to phenol, whose aromatic ring is activated towards electrophilic substitutions by the OH group. All AzoSAILs were characterized by nuclear magnetic resonance (NMR) spectroscopy and elemental analysis. Additionally, the absence of halide contaminations was confirmed by silver chromate tests [29]. An overview of the synthesized AzoSAILs is provided in Scheme 1; details are available in the Supporting Information.

**FIGURE 1:**
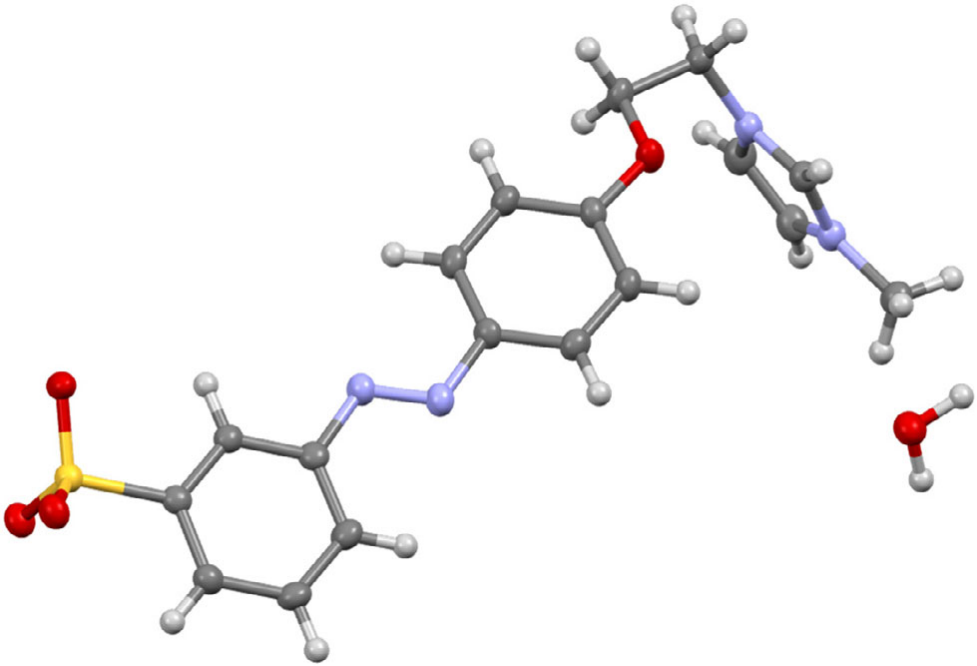
Molecular structure of [*m*‐SO_3_AzoC_2_ImC_1_]·H_2_O (**11**) obtained by single‐crystal X‐ray diffraction (H = white, N = blue, C = gray, O = red, S = yellow) with ellipsoids represented at 50% probability level.

**FIGURE 2:**
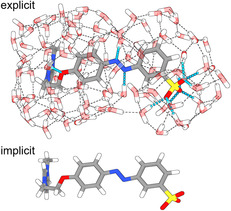
Optimized structures of **11** for the *trans*‐isomer. Top: optimized using the QM/QM approach including 100 water molecules. Bottom: CPCM model for water. H‐bonds were predicted with the ChimeraX software and those between the molecule and the solvent shell are highlighted in blue.

Single crystals suitable for X‐ray diffraction were obtained for compounds **6a**, **11**, and **13** by slow evaporation of their aqueous solutions at room temperature. In **6a**, the perrhenate anion is tetrahedrally coordinated and forms hydrogen bonds with imidazolium protons at the backbone and C2 positions (Figure S55, Supporting Information), consistent with interactions observed in previously reported FSAILs [17]. Molecular packing reveals parallel alignment of azobenzene units with intramolecular π–π interactions, with centroid‐to‐centroid distances ranging from 4.948 to 4.991 Å (Figure S56, Supporting Information). In the zwitterionic AzoSAILs **11** and **13**, the molecular structure incorporates one water molecule per formula unit (Figure 1 and Figure S59, Supporting Information), which indicates a strong interaction with the solvent. This is further supported by the short H‐contacts ranging from 1.867 to 1.915 Å and 1.895 to 1.927 Å for **11** and **13**, respectively (Figures S57 and S60, Supporting Information). Notably, the packing of these zwitterions displays a layered arrangement with intercalated water layers (Figures S58 and S61, Supporting Information). Unlike 6a, π–π‐stacking of the phenyl rings is absent; instead, intramolecular interactions involving the imidazolium cation dominate the packing motif. The solid‐state structures of **11** and **13** were the starting points for our quantum‐chemical studies. These calculations highlight the influence of solvent interactions on the molecular structures: In the absence of a solvent model, geometry optimizations yield folded structures, likely stemming from *π*‐stacking and electrostatic interactions between the separated charges (Figures S65 and S66, Supporting Information). Inclusion of a small solvent shell of 100 water molecules by means of QM/QM calculations, however, yields structures close to the experimental ones including several hydrogen bonds (Figures S67–S70, Supporting Information). Notably, the solvent shell can be effectively approximated with an implicit solvation model, which made further studies of the thermodynamics and photoisomerization processes feasible (see Figure 2 and Figures S65–S70, Supporting Information).

### 2.2 Studies on the Reversible Isomerization of AzoSAILs

The photoisomerization of azobenzene‐based surfactants induces substantial geometry changes in molecular structure, leading to significant alterations in physicochemical properties. These changes are particularly relevant for catalytic applications, as they offer the potential to modulate micellization behavior and consequently catalytic activity in a controllable manner. Therefore, the impact of different structural motifs on (i) the quantitative formation of the photostationary states (PSS), (ii) solvent effects on the isomerization process, and (iii) the (thermal) stability of the *cis*‐isomer is of particular interest.

**FIGURE 3:**
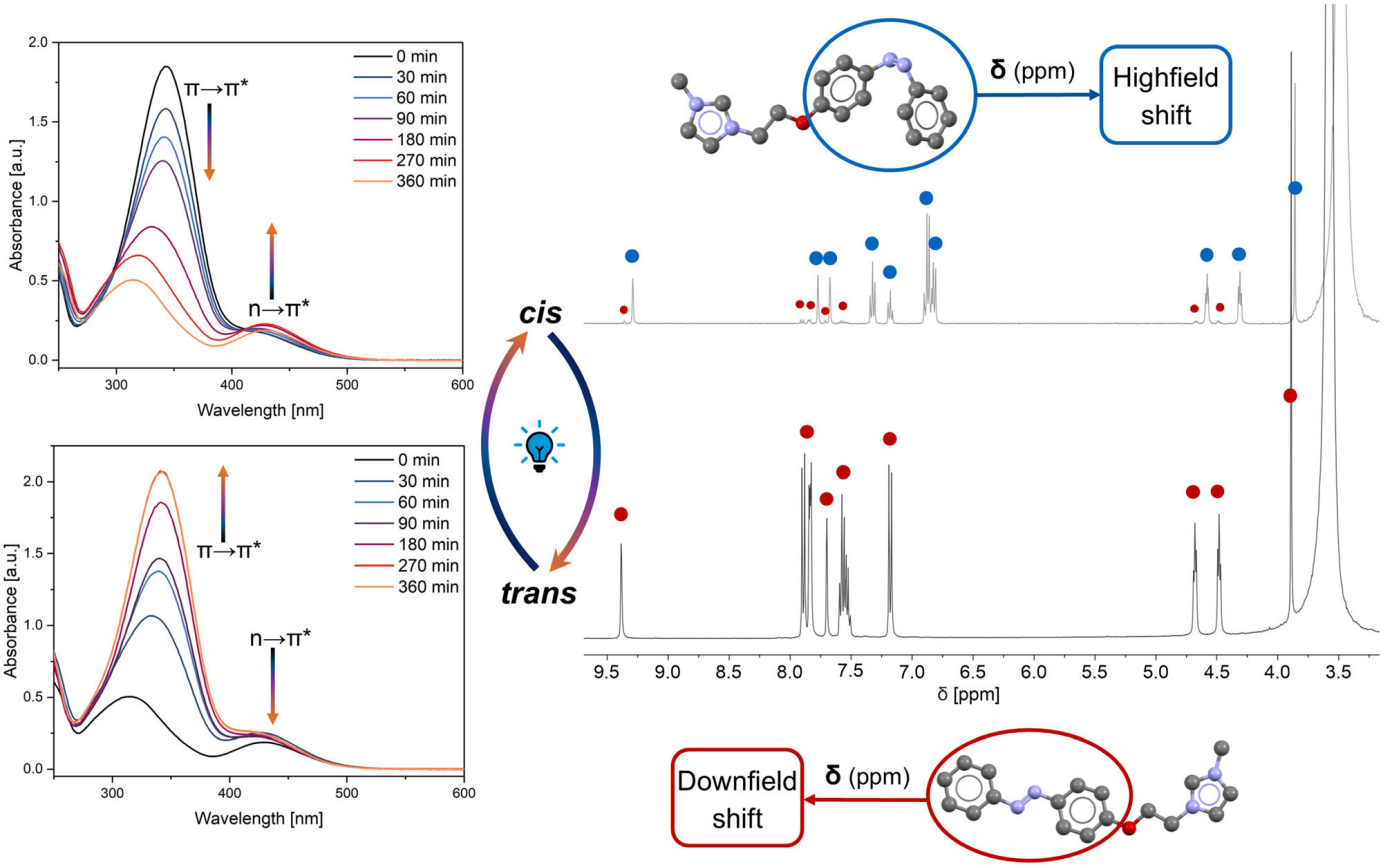
Left: UV–vis spectra of the *trans*‐ to *cis*‐isomerization of a 178 mmol/L solution of **1b**, resulting in decrease of the *π *→ π*‐ and increase of the *π *→ π*‐absorption bands (top) and reverse *cis*‐ to *trans*‐isomerization where the absorption bands behave vice versa (bottom). Right: ^1^H NMR spectra of a 10 mmol/L solution of **1b** in DMSO‐d_6_ after irradiation with 365 nm for 15 h (top) resulting in a upfield shift of the measured resonances and after subsequent heating of the *cis*‐**1b** solution for 24 h to quantitatively form the *trans*‐form, underlined by the downfield shift of the proton resonances (bottom).

To isomerize the AzoSAILs from the thermodynamically stable *trans*‐form to the meta‐stable *cis*‐form, AzoSAIL solutions were irradiated with 365 nm light. For ether‐bridged AzoSAILs (compounds **1**–**8**), photoisomerization to the *cis*‐form results in two distinct peaks in the UV–vis spectra at 310–327 and 427–436 nm, corresponding to the *π *→ π* and *n *→ π* transitions, respectively (Figure 3 and Figures S3, S5, S7, S12–S14, Supporting Information) [30]. Reversion to the *trans*‐form can be achieved either by irradiation at 460 nm (Figure 3 and Figures S2, S4, S6, S8, Supporting Information) or by thermal treatment at 50°C (Figure 3 and Figures S11–S13, Supporting Information), restoring a single *π *→ π* transition band in the range of 343–348 nm. Notably, a slight bathochromic shift of the absorption maximum to 353 nm is observed for the dicationic derivative, likely due to an extended conjugated system. Solvent effects on the isomerization process were found to be minimal. In all tested solvents (DMSO, water, and 50 wt.% aq. H_2_O_2_), quantitative formation of both PSS was observed (Figures S1–S4, S9 and S10, Supporting Information). However, UV irradiation in the presence of H_2_O_2_ over long time periods leads to partial decomposition due to the generation of OH radicals, which attack the diazo moiety and result in surfactant degradation [31]. Interestingly, although the *trans*‐form is thermodynamically more stable, the reverse *cis *→ *trans* isomerization upon 460 nm irradiation proceeds more slowly. This behavior is attributed to the lower photon energy associated with longer wavelengths (Figures S1 vs. S2 and S7 vs. S8, Supporting Information). Moreover, simultaneous heating to 50°C and irradiation at 365 nm does not result in *cis*‐isomer formation, as the thermal energy accelerates the *trans*‐reversion (Figure S11, Supporting Information). Another parameter that influences the kinetics of the isomerization is the surfactant concentration, with the isomerization being slower at higher concentrations (Figures S5 vs. S7, Supporting Information), as previously shown in literature [20]. To precisely determine the PSS composition after irradiation, samples were further analyzed via ^1^H NMR spectroscopy (Figure 3 and Figures S20 and S21, Supporting Information). Photoisomerization to the *cis*‐form results in upfield shifts of signals, especially those of the phenyl rings, due to the anisotropic effect of the *π*‐system of the aromatic rings from the altered geometry [32, 33]. In all examined cases the *cis*‐form is formed nearly in quantitative amounts (90%–99%) by irradiation with UV‐light (Table S1, Supporting Information). Structural variations, including *para*‐substitution of the azobenzene moiety or the use of longer ether‐based spacers (–OC_6_H_12_–), did not significantly affect PSS formation or absorption characteristics. Perrhenate and tungstate counterions, selected for their relevance in epoxidation catalysis, also showed no substantial influence on isomerization behavior. The only exception was the lower solubility of [ReO_4_]^−^ in water relative to [WO_4_]^2−^, consistent with literature on alkylimidazole perrhenates [9, 10]. In all cases, the *cis*‐form remained stable in the dark at room temperature (or below) for more than 24 h, with only 10%–20% reverting to the *trans*‐form. Note that at concentrations above the CMC, the micellar environment influences the kinetics of the *trans*‐to‐*cis* isomerization due to increased steric hindrance within the confined space of the supramolecular aggregates [34, 35]. However, since the surface activity provided by micelles is crucial for overcoming mass transport limitations in biphasic systems, the majority of experiments are conducted above the CMC. As a result, this effect is not explored in further detail. Unlike the other derivatives, compound **9** exhibits unique behavior: while the *trans*‐form can be quantitatively isolated, the *cis*‐form reaches a maximum of only ∼55% conversion. This is not due to rapid thermal back‐isomerization, as the *cis* content remains stable for over 5 h at room temperature. Instead, this behavior is likely due to steric constraints: the rigid methylene bridge in compound **9**, in contrast to the flexible ether linkage in other derivatives, restricts conformational flexibility. The reduced degrees of freedom limit the ability of the imidazolium head group to accommodate the geometric rearrangement required for efficient *cis*‐isomer formation. Additionally, the *π *→ π* absorption band of the *trans*‐form at 321 nm shows a marked hypsochromic shift (Figure S15, Supporting Information), likely reflecting the reduced conjugation and weaker electron‐donating properties of the methylene bridge compared to the ether linkage.

Direct linkage of the imidazole moiety to the azobenzene unit induces a noticeable bathochromic shift in the *π *→ π* absorption band, attributed to efficient delocalization of electron density across the conjugated aromatic and heterocyclic systems, as well as enhanced intramolecular push–pull effects (Figures S16 and S17, Supporting Information). Despite this favorable electronic interaction, photoisomerization of compound **10b** to its *cis*‐form upon 365 nm irradiation could not be detected by spectroscopic methods. In contrast **10c**, which contains a [NO_3_]^−^ instead of a [WO_4_]^2−^ anion shows a PSS of the *cis*‐form up to 86% (Figure S23, Supporting Information). However, the thermal stability of the *cis*‐isomer is significantly lower than that observed for other AzoSAILs. After only 15 min in the dark at room temperature, the *cis*‐content decreases from 86% to 46%, and drops further to 26% after an additional 15 min. This rapid back‐isomerization is consistent with known behavior of N—H arylazoimidazoles, which typically exhibit very short *cis*‐lifetimes (*t*_1_/_2_ ≪ 1 min) due to facile reisomerization to the *trans*‐form via tautomeric pathways [36]. However, *N*‐methylation at one nitrogen position on the imidazole ring is known to inhibit tautomerism, significantly extending the *cis*‐lifetime to 6–24 h [36].

The contrasting behaviors observed between **10b** and **10c** also highlight the role of the counterion [37, 38]. The energy difference between the *trans* and *cis*‐isomer of **10b** is the highest among all within this study investigated structures (Table S8, Supporting Information). Most likely, the bivalent [WO_4_]^2−^ anion can engage in stronger intra‐ or intermolecular interactions with the imidazolium cation, potentially restricting the molecular flexibility necessary for efficient *cis*‐isomer stabilization. In contrast, the monovalent and less coordinating [NO_3_]^−^ anion imposes fewer structural constraints, possibly allowing for higher conversion to the *cis*‐form. Given the limited isomerization behavior of AzoSAILs **9** and **10b/c**, particularly in terms of *cis*‐form formation and stability, these compounds were not considered suitable for further investigation as switchable surfactants.

Sulfonate groups were introduced into the azobenzene scaffold, as these groups are not only lipophobic and reduce the miscibility with organic media but also allow systematic investigation of *ortho*‐, *meta*‐, and *para*‐substitution of zwitterionic AzoSAILs effects on solubility and self‐assembly compared to ionic ones. Zwitterionic AzoSAILs **11** and **12** show reversible and quantitative isomerization. The absorption bands resulting from the two PSS are in the same range as their ionic derivatives (Figures S18 and S19, Supporting Information). Additionally, the *cis*‐form is stable at room temperature for several days (Table 1 and the Figures S25 and S29, Supporting Information). Note that the effect of Na_2_WO_4_ on the isomerization, and therefore also on the solubility, is negligible (loss of 1% vs. 3% and 16% vs. 21% after 8 and 48 h for the pristine sample vs. the metal salt containing one, Figures S25 and S26, Supporting Information). As expected, thermal activation accelerates back‐isomerization: heating to 50°C reduces the *cis*‐content of **11** to 49%, 12%, and 4% after 8, 24, and 48 h, respectively (Figure S27, Supporting Information).

**TABLE 1:** Overview of the achievable PSS of zwitterionic AzoSAILs 11–13, their solubilities and the stability of 200 mmol/L solutions in *cis*‐form at r.t. The stability is given in % in regard to the remaining *cis*‐form of the PSS.

Surfactant	Isomer	PSS, %	Solubility, mmol/L	PSS stability, %
11	*trans*	>99	1.3	—
	*cis*	96	209.9	8 h: 97^a^
				24 h: 86^a^
				48 h: 84^a^
12	*trans*	>99	97.5	—
	*cis*	86	2964	8 h: 95
				24 h: 88
				48 h: 72
13	*trans*	>99	trace	—
	*cis*	80	3.4	n.a.

a 2 equiv. Na_2_WO_4_ were added to determine to impact of metal salts.

**FIGURE 4:**
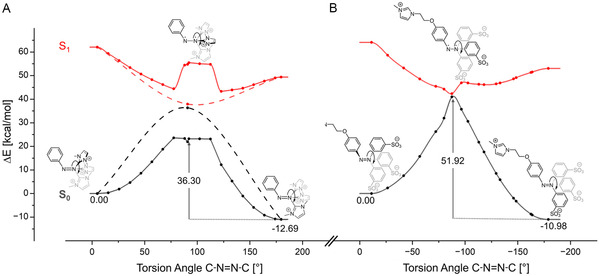
PES of the ground state and first excited state of **10** ^**+**^ (left, A) and **11** (right, B). The dashed line is physically not meaningful, instead, only used for visualization purposes to connect the explicitly found CI with the *cis* and *trans* isomers.

To understand the fundamentally different (photo)isomerization behavior of **10b** and **11**, we performed quantum chemical studies. For **10b**, only the cationic fragment of the AzoSAIL was considered. A dedicated conical intersection (CI) search yields, as anticipated, a structure with a C—N=N—C dihedral angle close to 90° (90.76°). Energetically, the CI lies below the first excited state of the *cis* and *trans* isomer of **10** ^**+**^, thus agreeing with the experimentally observed photoisomerization. Additionally, a relaxed surface scan along the twisting of the N=N double bond was performed and for each step, the energies of the ground and first excited state were calculated using TDDFT. The results are summarized in Figure 4 and indicate a rather low‐lying activation barrier for the back‐isomerization of the *cis* into the *trans* isomer of ca. 23.61 kcal/mol. Within the error of typical DFT calculations, this activation barrier supports the experimentally observed spontaneous back isomerization, further promoted by the energy gain of 12.69 kcal by relaxing the system into the *trans*‐isomer. Together, these calculations explain the experimentally observed incomplete photoisomerization, as the photoisomerization from *trans* to *cis* competes with the thermal reverse reaction.

In the case of the zwitterionic species **11**, twisting of the N=N double bond results in an activation barrier of 51.92 kcal/mol, prohibiting any thermal reactions. Notably, the approximated transition state is identical to a CI, which again lies energetically below the first excited states of both the *cis* and the *trans* isomer. Therefore, the results are in agreement with the experimentally observed photoisomerization of *trans*‐**11** to *cis*‐**11**. The presence of the CIs with a strong biradical character of the *S*_0_ and *S*_1_ states is further corroborated by CASSCF (see the Supporting Information for further details).

In contrast to AzoSAILs **1**–**8,** the formation of the *cis*‐isomer in zwitterionic AzoSAILs induces an extraordinary change on the solubility, which were determined by ^1^H qNMR. The sulfonate‐group in meta‐position leads to significant changes in the solubility of the two PSS of **11**; while *trans*‐**11** is poorly soluble in water (1.3 mmol/L) isomerization increases the solubility by a factor of >160–210 mmol/L for *cis‐* **11** (Table 1 and Figure S25, Supporting Information). This most likely results from the increased ionic intramolecular interactions, disrupting π–π stacking within the molecule due to geometry changes, and resulting in close a proximity of the charged groups that promotes solvation by enhancing interactions with the solute. Conversely, *trans*‐**11** is stabilized by hydrophobic and π–π interactions, and molecules appear to adopt a head‐to‐tail arrangement in which the sulfonate group of one molecule aligns with the imidazolium moiety of another. This spatial organization may facilitate intermolecular charge neutralization, as indicated by the molecular packing, thereby reducing water solubility (Figure S58, Supporting Information). The solubility of ortho‐substituted **12** is higher in its *trans*‐ as well as in the *cis*‐isomer. In the trans‐state, the closer spatial proximity of charged groups yields moderate solubility (98 mmol L^−1^), while isomerization increases this by a factor of 34. The sulfonate group in *para*‐position did not result in any favorable properties as **13** is hardly soluble and the isomerization is not quantitative, which most likely is due to the insolubility, which results from the intramolecular charge separation that leads to two ionic head groups and the intermolecular charge neutralization of the molecules (Figure S59, Supporting Information). These results indicate a strong influence of the position of the sulfonate group on the physicochemical properties. To further investigate the role of the anionic charge in such zwitterionic AzoSAILs, structure **11** was modified by replacing the sulfonate with an alkoxide in a theoretical study (structure **14** in the Supporting Information). However, no influence on the absolute energy difference compared to the other zwitterionic AzoSAILs can be observed, indicating no significant impact of a more localized negative charge. Importantly, neither zwitterion is soluble in olefins or the corresponding epoxides. This study reveals the impact of marginal changes of substitution patterns, such as permutating the constitution of the phenyl moiety, of azobenzene derivatives can profoundly impact physicochemical properties.

### 2.3 Micellar Aggregation of Photoisomers

The distinct physicochemical properties of the two PSS of AzoSAILs enable modulation of micellization behavior [19, 20], and emulsification of immiscible liquids [21, 22]. Both directly influence surface activity, which is detrimental for catalytic activity in biphasic systems. To investigate the potential for light‐induced aggregation and disaggregation, the behavior of selected AzoSAILs in aqueous media was examined at a concentration of 5 mmol/L. AzoSAIL **3b** forms micelles in the single digit nm range, while the size distribution obtained via dynamic light scattering (DLS) measurements, indicates that the *cis*‐isomer forms slightly larger aggregates than the *trans*‐form (Figure S34, Supporting Information). In either case, the micelles swell up and increase in size upon treatment with cyclooctene (Figures S35 and S36, Supporting Information). Similarly, **5b** forms micelles of comparable size for both PSS, and both states remain surface‐active (Figure S37, Supporting Information). DLS measurements revealed that *trans*‐**1b** forms small micelles in the same order of magnitude as other AzoSAILs, other significantly larger aggregates in the μm range are present as well, while for the isomerized *cis*‐**1b** forms only large micelles are detected (Figures, S32 and S33, Supporting Information). These aggregates are larger in water than in aqueous H_2_O_2_, which is further confirmed by TEM imaging (Figure S42, Supporting Information). In general, the micellar structures formed by AzoSAILs display polydispersity, and the large size of the aggregates of *cis*‐**1b** indicate weak intramolecular interactions present, however, micellar collapse as reported for structurally analogous compounds does not occur [19]. The surface‐activity of both PPS at a concentration of 5 mmol/L is supported by CMC measurements of **1b** in aq. H_2_O_2_, which show a CMC of 4.0 and 4.2 mmol/L for *trans*‐ and *cis‐* **1b**, respectively (Figures S30 and S31, Supporting Information), indicating a negligible difference (ΔCMC = 0.2 mmol/L). This minimal impact of photoisomerization on CMC is consistent with previous literature on azobenzene surfactants [28, 39, 40, 41]. Consequently, the AzoSAILs examined here are unsuitable for use as photoresponsive catalysts in biphasic epoxidation reactions. This is due to the very limited CMC shift induced by photoisomerization, and the inherently low concentrations required, which are insufficient for effective surfactant‐based catalysis. Therefore, design strategies to develop AzoSAILs in which the solubility and micellization behavior of each photoswitchable state (PSS) can be precisely tuned, enhancing not only photoswitchable catalytic activity but also improving catalyst recovery and recycling are desired. In particular, light‐controlled water solubility while reducing miscibility with organic media allow for more efficient phase separation and catalyst reuse as demonstrated by zwitterionic derivatives. Despite its low solubility, *trans*‐**11** forms micellar aggregates in aqueous media, exhibiting a highly polydisperse size distribution with nanostructures ranging from tens to hundreds of nanometers (Figures S38 and S41, Supporting Information). Upon addition of substrate, aggregate size increases, indicating substrate incorporation (Figure S39, Supporting Information). The more soluble *cis*‐isomer also shows surfactant properties and forms larger supramolecular aggregates, consistent with previous AzoSAIL studies (Figures S38, S40 and S41, Supporting Information). TEM analysis supports this, revealing spherical micelles that further aggregate into nonuniform structures (Figure S43, Supporting Information). Unlike classical ionic surfactants that solubilize substrate in micelle cores [10, 12, 17], **11** forms oil‐in‐surfactant microemulsions with encapsulated olefin droplets (Figure S44, Supporting Information) [42, 43].

### 2.4 Screening of AzoSAILs as Epoxidation Catalysts

To evaluate their catalytic performance and phase behavior, selected AzoSAILs were tested as catalysts for the biphasic epoxidation of cyclooctene with aq. H_2_O_2_ (see Supporting Information for detailed procedures). Unless stated otherwise, all experiments were conducted with the AzoSAILs in their thermodynamically stable *trans*‐form. Consistent with previous studies, AzoSAILs bearing the perrhenate anion exhibited moderate catalytic activity at elevated temperatures (Figure S45, Supporting Information), comparable to alkylimidazolium and FSAIL derivatives [9, 17]. As observed for FSAILs, the formation of the epoxide product, which also functions as a phase‐transfer agent, leads to the migration of the catalyst into the organic phase. However, due to the inherently low solubility of perrhenate AzoSAILs, a fraction of the catalyst remains undissolved.

In case of tungstate AzoSAILs, phenylphosphonic acid (PPA) is used as an additive as the formation of a phosphonate‐di(peroxo)‐tungstate which is catalytically more active compared to the peroxotungstate that is formed in the presence of H_2_O_2_ [10, 17, 44]. The catalytic performance of **1b** was found to be comparable to that of pristine 1‐octyl‐3‐methylimidazolium tungstate under analogous conditions (Figure S46, Supporting Information). This is attributed to the epoxidation occurring in the aq. phase. At high surfactant concentrations (178 mmol/L), the effect of isomerization was negligible, with the *cis*‐form catalysis yielding similar results as the *trans*‐form (Figure S47, Supporting Information). However, at lower concentrations (10 mmol/L) at room temperature, conditions required for a photoswitchable system based on changes in the CMC, only modest conversions were observed, reaching 3% and 15% after 16 h in the absence and presence of PPA, respectively.

Increasing the amount of lipophilic organic moieties such long alkyl chains at the phenyl‐ or imidazole moiety or increasing the length of the aliphatic bridge results in a significant increase of the catalytic activity (Figure S48, Supporting Information, left). Notably, **5b**, which features an ammonium cation in place of the imidazolium, achieved complete conversion within 60 s while maintaining >99% selectivity for cyclooctene oxide. This exceptional performance is comparable to that of tungstate FSAILs and can be attributed to quasihomogeneous reaction conditions arising from full phase transfer of the catalyst into the organic phase (Figure S49, Supporting Information, right) [17]. As a result, AzoSAILs **2**–**8**, regardless of the counterion used, are not suitable for the development of a light‐switchable biphasic epoxidation system. Such a system would ideally allow reversible control over catalytic activity via micelle assembly/disassembly in the aqueous phase and efficient recovery and recycling of the catalyst from the aqueous phase. In this context, the high stability of the *cis*‐isomer alongside the switchable solubility are desirable properties for catalytic applications. To evaluate sulfonate‐mediated H_2_O_2_ activation, epoxidation of cyclooctene was attempted using **12** at high concentration (357 mmol L^−1^, 80°C). However, only minimal conversion (5% after 24 h; Figure S49, Supporting Information) was observed, underlining that the sulfonate functionalization is unable to efficiently activate H_2_O_2_. Subsequent studies focused on **11** due to its light‐controllable solubility, enabling phototriggered increase in surface activity via 365 nm irradiation and facile recovery of the insoluble *trans*‐form. Due to the inactivity of the sulfonate group an external epoxidation catalyst has to be used to create an efficient catalytic system. Prior to each run, **11** was quantitatively isomerized to the soluble *cis*‐form in water to avoid decomposition due to irradiation in H_2_O_2_. Sodium tungstate was tested as active metal salt but yielded low conversions at ambient temperature, likely due to slow formation of the active tetraperoxotungstate species (Figure S50, Supporting Information). Moreover, prolonged reactions led to precipitation of **11** as the *trans*‐isomer, limiting activity due to diffusion constraints in the biphasic system. Combining Na_2_WO_4_ with PPA modestly improved conversion (Figure S51, Supporting Information) but also accelerated precipitation. In contrast to one component AzoSAIL systems with perrhenate and tungstate as counterion, which are transferred to the organic phase and selectively convert the olefin, zwitterionic **11** remains in the aqueous alongside the external tungstate—and peroxotungstate—which then oxidizes the diazo‐moiety. Anilines or azobenzene derivatives are known to be oxidized to azooxybenzene under the presence of H_2_O_2_ and metal oxide catalysts based on tungsten and other metals [45, 46, 47, 48]. Thus, methyltrioxorhenium (MTO), which is known to be a very active epoxidation catalyst under mild conditions [49], is tested. In conventional systems, coordinating bases are used to stabilize the catalyst, however, they also facilitate the phase transfer of MTO into the organic phase, which complicates postcatalysis separation, posing a significant challenge for the recovery of costly transition metal catalysts [50, 51, 52]. It is known that sulfonate is also able to coordinate to the rhenium center and enhance its catalytic performance [53]. Therefore, it was aimed to contain MTO in the aq. phase while stabilizing it with **11** as a phase transfer catalyst. As intended MTO was kept in the aq. phase, but owing to the acidic condition, MTO degrades to the perrhenate anion, which is a significantly slower catalyst in micellar media compared to MTO or tungstate. Furthermore, and in analogy to Na_2_WO_4_, extended reaction times led to oxidation of **11** to the *trans*‐azooxybenzene. This leads to instant the isomerization to the *trans*‐form and hence to precipitation of **11** due to the low solubility of the *trans*‐isomer. The SC‐XRD analysis verifies both the interaction of **11** with MTO as its diperoxo‐species and the oxidation of the N=N group (Figure S62, Supporting Information). To mitigate catalyst degradation, pyrazole (24 equiv. relative to MTO) was added [52], but no synergistic effect was observed, likely due to the higher affinity of **11** for MTO (Figure S53, Supporting Information). Switching to urea‐hydrogen peroxide (UHP) as a milder oxidant did not prevent catalyst deactivation. Lowering the reaction temperature to 0°C and increasing pyrazole concentration also failed to yield full conversion (Table S3, Supporting Information). Only at 150 equiv. of pyrazole was full conversion achieved after 1 h, but at the cost of MTO transferring into the organic phase (Figure S54, Supporting Information), impeding a sustainable system that offers feasible catalyst recycling. Therefore, while **11** offers a light‐controlled that can reversibly enhance the solubility/miscibility of organic media with water and an easy recycling from therein by applying an external stimulus, the use in systems for oxidation catalysis with additional catalysts is limited due to the affinity of the azo‐bridge being oxidized.

## 3 Conclusions

This study presents a detailed theoretical and experimental investigation into the structure–property relationships of AzoSAILs, highlighting how structural variations can profoundly affect their physicochemical behavior. While ionic AzoSAILs **1**–**8** demonstrate high catalytic activity in epoxidation reactions, photoswitchable control over catalysis could not be achieved within practical concentration ranges. For zwitterionic AzoSAILs subtle modifications, such as the positional isomerism of sulfonate substituents, exhibit unprecedented photoresponsive behavior. This is reiterated in the computational analysis, highlighting the interaction between AzoSAILs with the solvent as well as the fundamentally difference in the photo isomerization behavior between the ionic structure **10b/c** and zwitterionic molecule **11**. This latter structure, featuring a *meta*‐sulfonate group, is of particular interest, offering light‐controlled solubility with its *cis*‐isomer showing a >160‐fold increase in water solubility compared to the corresponding *trans*‐form.

Although its application in sustainable oxidation catalysis is limited due to oxidation of the diazo‐moiety under typical reaction conditions (e.g., H_2_O_2_ in the presence of metal‐oxo catalysts), its unique switchable solubility and immiscibility with organic media, along with its ability to form micellar aggregates, enable efficient encapsulation of hydrophobic substrates. These properties offer promising opportunities in stimuli‐responsive self‐assembly, molecular delivery, and due to the metal‐coordinating sulfonate group, for applications in separation technologies and wastewater treatment.

Overall, this work underscores the significance of precise substitution patterning in tuning azobenzene‐based systems and reveals both the potential and challenges in designing photoswitchable materials for catalysis and beyond, opening pathways toward smart, light‐responsive materials for multiphase environments.

## 4 Experimental

A detailed description of the analytical methods, synthetic procedures of the precursors and the catalytic setup are given in the Supporting Information.

## Supporting Information

Additional supporting information can be found online in the Supporting Information section.

## Funding

This study was supported by Deutsche Forschungsgemeinschaft (Co 1543/1‐2, Je 257/24‐2, Co 2329/2‐1).

## Conflicts of Interest

The authors declare no conflicts of interest.

## Supporting information

Supplementary Material

## Data Availability

The data that support the findings of this study are available from the corresponding author upon reasonable request.
