# Supplementary material for: Azobenzene‐Functionalized Ionic Liquids: Light‐Responsive Surfactants With Catalytic Potential
Source: ChemistryOpen. 2026 Apr 20;15(5):e70203. doi: 10.1002/open.70203 (PMC13096569; doi:10.1002/open.70203)
Supplement: Supplementary file 1 — Supplementary Material [file OPEN-15-e70203-s001.pdf]

Azobenzene-Functionalized Ionic Liquids: Light-Responsive Surfactants with Catalytic Potential

Markus Hegelmann,‡,[a] Stefan Frei, ‡,[b] Julian Zuber,[a] Johannes Luibl,[c] Wolfgang Korth,[c] Andreas Jess,[c] Peter Coburger,[b] and Mirza Cokoja [a],*

[a] Technical University of Munich, Department of Chemistry and Catalysis Research Center, Ernst-Otto-Fischer-Straße 1, D-85748 Garching bei München, Germany. E-mail: mirza.cokoja@tum.de

[b] Technical University of Munich, School of Natural Sciences, Department of Chemistry, Lichtenbergstraße 4, D-85748 Garching bei München, Germany.

[c] University of Bayreuth, Faculty of Engineering Science, Chair of Chemical Engineering, Universitätsstraße 30, D-95447 Bayreuth, Germany.

Supporting Information

Table of Contents

1. Experimental **3**

1.1. Analytical methods, calculations, and characterization techniques 3

1.1.1. Materials 3
1.1.2. Dynamic light scattering (DLS) 3
1.1.3. Elemental analysis (EA) 3
1.1.4. Isomerization experiments 3
1.1.5. Nuclear magnetic resonance spectroscopy (NMR) 4
1.1.6. Surface tension measurements 4
1.1.7. Transmission electron microscopy (TEM) 4

1.2. Synthesis of azobenzene-functionalized surface-active ionic liquids (AzoSAILs) 5

1.2.1. Overview of the synthetic routes to AzoSAILs with different structure motifs 5
1.2.2. Miscellaneous compounds 6
1.2.3. Azobenzene derivatives 7
1.2.4. Bromoethoxy-functionalized diphenyldiazenes 11
1.2.5. Azobenzene-functionalized potassium benzenesulfonates 13
1.2.6. Azobenzene-functionalized imidazolium ammonium halides 15
1.2.7. Azobenzene-functionalized imidazolium and ammonium perrhenates 20
1.2.8. Azobenzene-functionalized imidazolium and ammonium tungstates 23
1.2.9. Azobenzene-functionalized imidazolium nitrates 28
1.2.10. Azobenzene-functionalized imidazolium benzenesulfonates 29

2. Analysis of AzoSAILs **31**

2.1. Isomerization studies 31

2.1.1. UV-Vis measurements 31
2.1.2. ^{1}H-NMR measurements 38

2.2. Solubility measurements 45
2.3. Critical micelle concentrations (CMCs) 46
2.4. Dynamic light scattering (DLS) 47
2.5. Transmission electron microscopy (TEM) 51

3. Epoxidation catalysis **53**

3.1. Epoxidation catalysis with perrhenate AzoSAILs 53
3.2. Epoxidation catalysis with tungstate AzoSAILs 54
3.3. Epoxidation catalysis with zwitterionic AzoSAILs 56

4. Crystallographic details **60**

4.1. Molecular structure and packing of [$C_4AzoC_6ImC_1$][ReO_4] (**6a**) 60
4.2. Molecular structure and packing of [*m*-$SO_3AzoC_2ImC_1$]·H_2O (**11**) 61
4.3. Molecular structure and packing of [*p*-$SO_3AzoC_2ImC_1$]·H_2O (**13**) 63
4.4. Molecular structure and packing of [*m*-$SO_3BnN_2OBnC_2ImC_1$]·H_2O·CH_3ReO·H_2O 64
4.5. Crystallographic data of [$C_4AzoC_6ImC_1$][ReO_4] (6a) 66
4.6. Crystallographic data of [m-$SO_3AzoC_2ImC_1$]·H_2O (**11**) 68
4.7. Crystallographic data of [*p*-$SO_3AzoC_2ImC_1$]·H_2O (**13**) 70
4.8. Crystallographic data of [*m*-$SO_3BnN_2OBnC_2ImC_1$]·H_2O·CH_3ReO·H_2O 72

5. Theoretical investigations **74**

5.1 Geometry optimization 74
5.2 Isomerization and excited states analysis of **11**, **10c** and **10**$^{+}$ 77
5.3 Cartesian coordinates of optimised structures 90

6. References **244**

1. Experimental

1.1. Analytical methods, calculations, and characterization techniques

1.1.1. Materials

1-Bromooctane, 1-methylimidazole, acetonitrile (MeCN, HPLC), Amberlite® IRA-402 (chloride form), ammonia (NH_3, 25%), aniline, chloroform ($CHCl_3$, puriss. and $CDCl_3$), cis-cyclooctene (COE, 95 %), concentrated hydrochloric acid (HCl, 37 %), diethyl ether (Et_2O, HPLC), dichloromethane (CH_2Cl_2, puriss.), DMSO-d6, D_2O, dibromoethane, dibromohexane, ethyl acetate (EtOAc, HPLC), lithium aluminium hydride ($LiAlH_4$), mesitylene, methyl iodide (MeI), naphthalene, N-bromosuccinimide, nitrosobenzene, n-hexane (HPLC), phenylphosphonic acid (PPA), potassium carbonate (K_2CO_3), rhenium oxide (Re_2O_7), sodium acetate (NaOAc), sodium bicarbonate ($NaHCO_3$), sodium carbonate (Na_2CO_3), sodium hydroxide (NaOH), sodium nitrite ($NaNO_2$), sodium tungstate dihydrate ($Na_2WO_4{\cdot}2H_2O$), tetrahydrofuran (THF, puriss.), triphenylphosphine (PPh_3), and tungstic acid (H_2WO_4) were purchased from *Sigma Aldrich*. 8 dram- and 4 ml vials for epoxidation catalysis (TraceClean®) and hydrogen peroxide (H_2O_2, 50 wt.% in water) were purchased from *VWR*. Carbon grids for TEM measurements were purchased from *Micro to Nano*. 1-Bromo-2-chloroethane, 18-crown-6, and phenol were purchased from *TCI chemicals*. 2-Aminobenzenesulfonic acid, 3-aminobenzenesulfonic acid, 4-aminophenol, butylaniline, and sulfanilic acid were purchased from *abcr*. and 1-Methylimidazole and all alkyl halides were distilled under vacuum and stored under an argon atmosphere until further use. All other purchased chemicals were used without further purification. Alkylation reactions were carried out under *Schlenk* conditions, the following synthesis steps and catalysis runs were carried out under air, if not stated otherwise.

1.1.2. Dynamic light scattering (DLS)

DLS was performed on a Malvern Zetasizer Nano in quartz cuvettes using 173° angle backscattering mode. The compounds were dissolved in 1.4 mL aqueous H_2O_2 (25 mmol) and measured at 20 °C in H_2O_2 or 25 °C in H_2O and D_2O. In order to investigate the substrate interaction, Cyclooctene (40 eq.) was added to the mixture. The biphasic system was shaken and after phase separation the aqueous phase is used for the measurement. Each sample was filtered by a syringe filter (LLG-Syringe filters SPHEROS, PTFE, 0.22 µm, 13 mm diameter by Lab Logistics Group GmbH) prior to the measurements. Micelle size distributions were interpreted from the correlograms using a general-purpose method.

1.1.3. Elemental analysis (EA)

Elemental analyses were performed at the Microanalytical Laboratory of the Technical University of Munich, Germany on a HEKAtech Euro EA CHNSO-Analyzer and a Varian AA280FS fast sequential AAS spectrometer.

1.1.4. Isomerization experiments

The isomerization of azobenzene-functionalized compounds was carried out by irradiating the solution with light at specific wavelengths: 365 nm (*trans* → *cis*) and 460 nm for the reverse process (*cis* → *trans*). In each case 3W LED lamps installed in an aluminum block with a cooling unit are used. In both cases, 3 W LED lamps were used, mounted in an aluminum block equipped with a cooling unit. The light was evenly dispersed into the solution via a sandblasted glass rod connected to the LED-containing aluminum block.

1.1.5. Nuclear magnetic resonance spectroscopy (NMR)

Liquid state NMR spectra were recorded by a *Bruker* AVIII 400 US (^{1}H: 400 MHz, 16 scans; ^{13}C: 101 MHz, 1024 scans) at ambient temperature (298 K). The ^{1}H NMR spectroscopic chemical shifts δ are reported in ppm relative to tetramethylsilane (for measurements in $CDCl_3$ and DMSO-d_6) and sodium 3-(trimethylsilyl)propionate (for measurements in D_2O). ^{1}H NMR spectra are calibrated against the residual proton and natural abundance carbon resonances of the respective deuterated solvent as an internal standard ($CDCl_3$: δ (^{1}H) = 7.26 ppm, DMSO-d_6: δ (^{1}H) = 2.50 ppm, D_2O: δ (^{1}H) = 4.79 ppm). The following abbreviations are used to describe signal multiplicities: s = singlet, d = doublet, t = triplet, p = quintet, h = sextet, dd = doublet of doublets, m = multiplet.

1.1.6. Surface tension measurements

The CMCs were determined by surface tension measurements with a K11 tensiometer (Krüss GmbH, Germany) according to the Wilhelmy plate method with an accuracy of 0.01 N m^{-2}. A vertically suspended plate touches the liquid, wets the plate and a force (F) can be measured. The surface tension is calculated according to the following equation:

$$\sigma ¼ F L \times \cos(\theta)$$

L is the length of the plate and θ the contact angle between liquid and solid surface of the plate. Due to the decomposition of hydrogen peroxide on platinum surfaces, the usually used platinum plates were substituted with glass plates. The data were drawn in a figure with surface tension over the logarithmic concentration of the surfactant to see a sharp break point at the CMC.

1.1.7. Transmission electron microscopy (TEM)

TEM measurements were carried out with a JEM 1400 plus microscope at 120 kV equipped with a Ruby CCD detector from JOEL. The copper grids (300 mesh) were glow discharged for one minute before being impregnated with the sample. For the impregnation each sample (5 µL) was coated onto the continuous carbon film of the copper grid by drop casting. After impregnation with the sample for 30 s the grids were flushed with 20 µL water. Excessive liquid was carefully removed with a filter paper.

1.2. Synthesis of azobenzene-functionalized surface-active ionic liquids (AzoSAILs)

1.2.1. Overview of the synthetic routes to AzoSAILs with different structure motifs

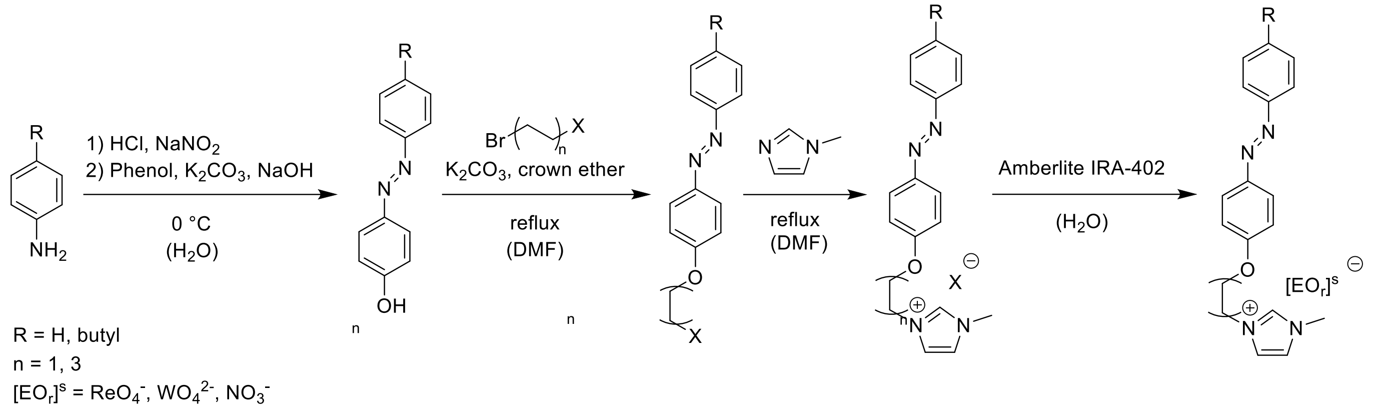

Scheme S1. Synthetic route towards AzoSAILs **1**-**8**.

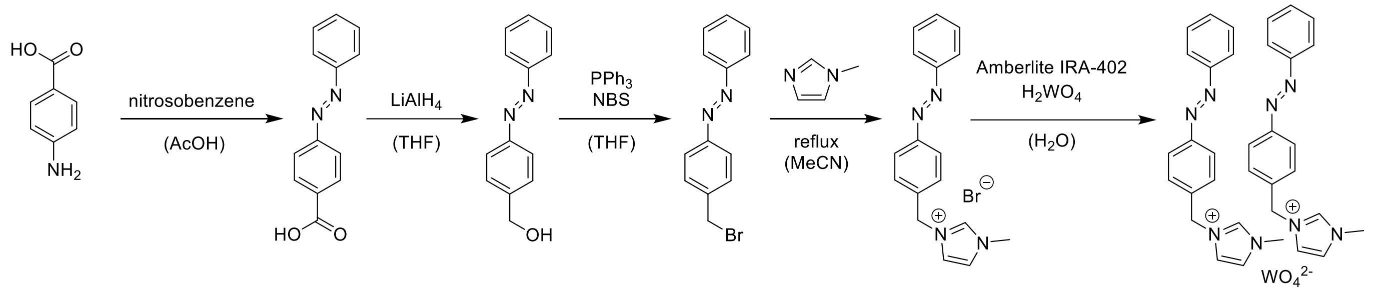

Scheme S2. Synthetic route towards AzoSAIL **9b**.

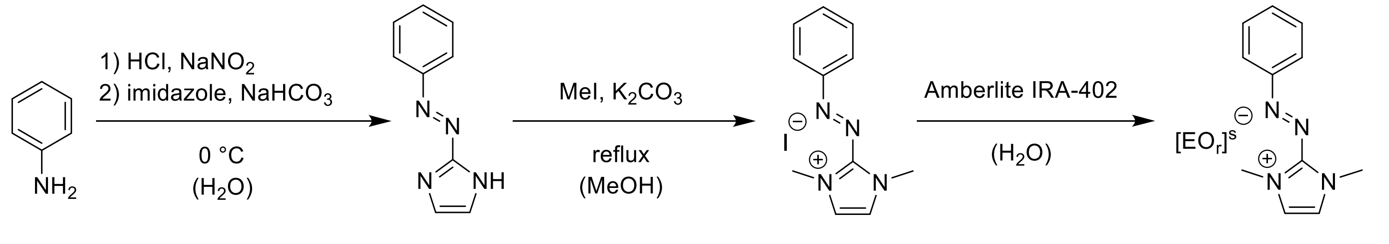

Scheme S3. Synthetic route towards AzoSAILs **10b** and **10c**.

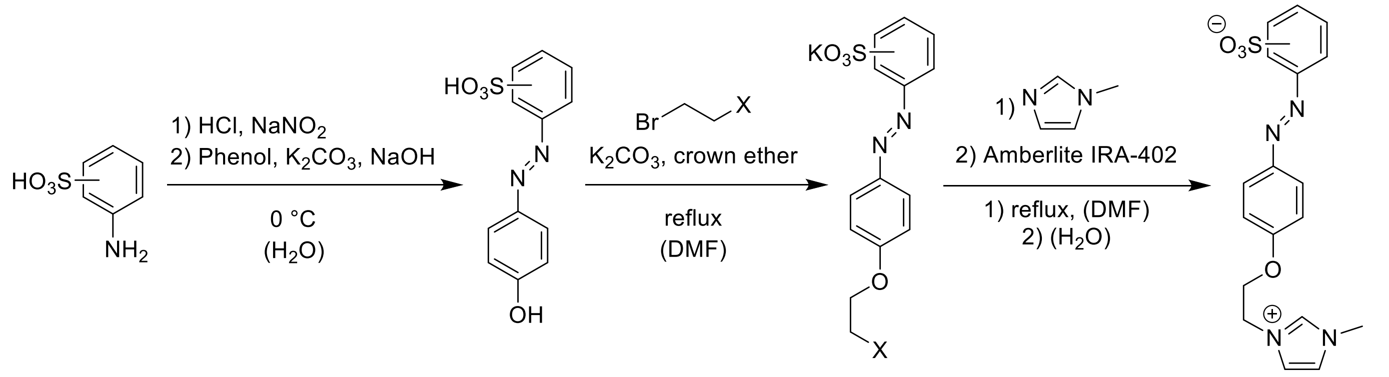

Scheme S4. Synthetic route towards AzoSAILs **11**, **12** and **13**.

1.2.2. Miscellaneous compounds

Ammonium perrhenate

The procedure was adapted according to previous literature reports.[1] Rhenium(VII) oxide (Re_2O_7, 7.5 g, 15.5 mmol, 1.0 equiv.) was stepwise dissolved in 100 mL of distilled water. The aqueous solution is neutralized by dropwise addition of 12.5 M aq. ammonia solution (2.7 ml, 33.8 ml, 2.2 equiv.) and subsequent stirring for 1 h at room temperature. After solvent removal the pale blue crude product is recrystallized in water to obtain 8.1 g (97 %) ammonium perrhenate (NH_4ReO_4) as colorless crystals.

Methyltrioxorhenium

The compound was synthesized according to previous literature.[2] Methyltrioxorhenium (MTO) was obtained as white solid and its analytical data are in accordance to the previously published data.

1-Octylimidazole

The procedure was adapted according to previous literature reports.[3] Imidazole (20.0 g, 293.8 mmol, 1.0 equiv.) and 1-bromooctane (51.1 ml, 293.8 mmol, 1.0 equiv.) were dissolved in 150 mL THF. Then, NaOH (12.9 g, 323.2 mmol, 1.2 equiv.) was dissolved in 15 ml water and the solution was added to the reaction mixture. The mixture was stirred under refluxed at 93 °C for 72 h. After cooling to room temperature 100 mL of distilled water was added, subsequently the aqueous phase was extracted with DCM (3x75 mL). Then, the solvent was removed under reduced pressure to obtain a yellow oil. The residue was purified by fractional vacuum distillation ($1x10^{-3}$ mbar) to yield 37.6 g (71 %) 1-octylimidazole as colorless liquid.
^{1}H-NMR (400 MHz, CDCl3, 298 K) δ [ppm]: 7.43 (s, 1H), 7.02 (s, 1H), 6.87 (s, 1H), 3.89 (t, *J* = 7.1 Hz, 2H), 1.78 – 1.70 (m, 2H), 1.31 – 1.17 (m, 10H), 0.85 (t, *J* = 6.8 Hz, 3H). ^{13}C-NMR (101 MHz, $CDCl_3$, 298 K) δ [ppm]: 137.09, 129.37, 118.83, 47.07, 31.76, 31.13, 29.12, 29.08, 26.59, 22.64, 14.09.

1.2.3. Azobenzene derivatives

4-(Phenyldiazenyl)phenol

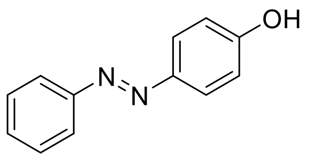

The procedure was adapted from literature.[4] A solution of aniline (18.4 g, 200.0 mmol, 1.0 equiv.) and 54 ml concentrated HCl (3.6 equiv.) in water (250 ml) was cooled to 0 °C. To this, a solution of $NaNO_2$ (13.8 g, 200.5 mmol, 1.0 equiv.) in water (30 ml) was added, and the reaction mixture was stirred for 10 min. Subsequently, a solution of phenol (18.8 g, 200.0 mmol, 1.0 equiv.), Na_2CO_3 (21.5 g, 202.3 mmol, 1.0 equiv.) and NaOH (8.0 g, 201.1 mmol, 1.0 equiv.) in water (120 mL) was added dropwise to the reaction mixture. The reaction was stirred at 0 °C for 4 h. Afterwards, the crude product was precipitated by addition of conc. HCl until pH 2. The solid was collected by vacuum filtration. The crude was recrystallized from EtOH:water (1:1, 60 ml) two times and dried under vacuum (10^{-3} mbar) to yield 33.7 g (85 %) 4-(phenyldiazenyl)phenol as a brown crystalline solid.

^{1}H-NMR (400 MHz, DMSO-d_6, 298 K) δ [ppm]: 7.85 – 7.76 (m, 4H), 7.58 – 7.45 (m, 3H), 6.99 – 6.91 (m, 2H). ^{13}C-NMR (101 MHz, DMSO-d_6, 298 K) δ [ppm]: 161.04, 152.12, 145.22, 130.49, 129.35 (2C), 124.88 (2C), 122.12 (2C), 115.98 (2C).

4-((4-Butylphenyl)diazenyl)phenol

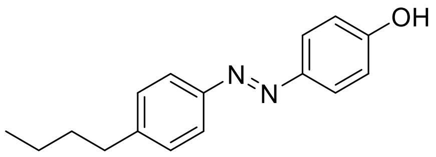

The procedure was adapted from literature.[4] A solution of butylaniline (37.3 g, 249.9 mmol, 1.0 equiv.) and 68 ml concentrated HCl (3.6 equiv.) in water (300 ml) was cooled to 0 °C. To this, a solution of $NaNO_2$ (17.3 g, 250.7 mmol, 1.0 equiv.) in water (40 ml) was added, and the reaction mixture was stirred for 10 min. Subsequently, a solution of phenol (23.5 g, 249.9 mmol, 1.0 equiv.), Na_2CO_3 (27.2 g, 256.5 mmol, 1.0 equiv.) and NaOH (10.3 g, 257.3 mmol, 1.0 equiv.) in water (150 mL) was added dropwise to the reaction mixture. The reaction was stirred at 0 °C for 4 h. Afterwards, the crude product was precipitated by addition of conc. HCl until pH 2. The solid was collected by vacuum filtration. The crude was recrystallized from EtOH:water (1:1, 100 ml) two times and dried under vacuum (10^{-3} mbar) to yield 51.5 g (81 %) 4-((4-butylphenyl)diazenyl)phenol as a brown crystalline solid.

^{1}H-NMR (400 MHz, DMSO-d_6, 298 K) δ [ppm]: 7.76 (dd, J = 17.7, 8.2 Hz, 4H), 7.36 (d, J = 8.0 Hz, 2H), 6.94 (d, J = 8.6 Hz, 2H), 2.65 (t, J = 7.7 Hz, 2H), 1.59 (p, J = 7.5 Hz, 2H), 1.32 (h, J = 7.4 Hz, 2H), 0.91 (t, J = 7.3 Hz, 3H).* ^{13}C-NMR (101 MHz, DMSO-d_6, 298 K) δ [ppm]: 160.75, 150.35, 145.30, 145.24, 129.15 (2C), 124.66 (2C), 122.13 (2C), 115.91 (2C), 34.65, 32.95, 21.77, 13.78.

4,4'-(diazene)diphenol

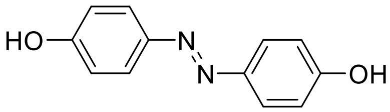

The procedure was adapted and modified from literature.[4] A solution of 4-aminophenol (27.3 g, 249.9 mmol, 1.0 equiv.) and 68 ml concentrated HCl (3.6 equiv.) in water (300 ml) was cooled to 0 °C. To this, a solution of $NaNO_2$ (17.2 g, 249.0 mmol, 1.0 equiv.) in water (40 ml) was added, and the reaction mixture was stirred for 10 min. Subsequently, a solution of phenol (23.7 g, 252.2 mmol, 1.0 equiv.), Na_2CO_3 (26.5 g, 249.9 mmol, 1.0 equiv.) and NaOH (10.0 g, 250.0 mmol, 1.0 equiv.) in water (150 mL) was added dropwise to the reaction mixture. The reaction was stirred at 0 °C for 4 h. Afterwards, the crude product was precipitated by addition of conc. HCl until pH 2. The solid was collected by vacuum filtration. The crude was recrystallized from EtOH:water (1:1, 25 ml) two times and dried under vacuum (10^{-3} mbar) to yield 1.9 g (5 %) 4,4'-(diazene)diphenol as a yellow crystalline solid.
^{1}H-NMR (400 MHz, DMSO-d_6, 298 K) δ [ppm]: 10.12 (s, 2H), 7.70 (m, 4H), 6.91 (m, 4H).

4 -(phenyldiazenyl)benzoic acid

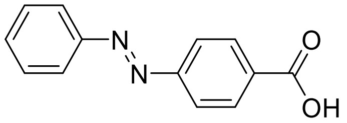

The procedure was adapted from literature.[5] A solution of nitrosobenzene (2.0 g, 18.7 mmol, 1.0 equiv.) in glacial acetic acid (15 ml) was added dropwise to a solution of 4-aminobenzoic acid (3.1 g, 22.4 mmol, 1.2 equiv.) in glacial acetic acid (20 ml). The reaction mixture was stirred for 24 h at room temperature. Subsequently, the solvent was removed under reduced pressure. The residual was suspended in water (25 ml) and lyophilized. The crude was recrystallized from EtOAc (25 ml) two times and dried under vacuum (10^{-3} mbar) to yield 3.0 g (71 %) 4-(phenyldiazenyl)benzoic acid as an orange crystalline solid.
^{1}H-NMR (400 MHz, DMSO-d_6, 298 K) δ [ppm]: 8.18 – 8.13 (m, 2H), 8.00 – 7.92 (m, 4H), 7.67 – 7.59 (m, 3H).

4-((phenyldiazenyl)phenyl)methanol

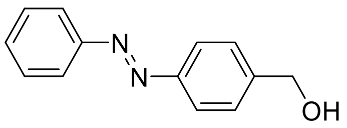

The procedure was adapted from literature.[5] A solution of 4-(phenyldiazenyl)benzoic acid (3.0 g, 13.3 mmol, 1.0 equiv.) in dry THF (60 ml) was added dropwise to a precooled suspension of $LiAlH_4$ (0.6 g, 15.9 mmol, 1.2 equiv.) in dry THF (40 ml). The mixture was stirred at 0 °C until complete addition and then allowed to warm up to room temperature under continuous stirring for 24 h. To quench excessive $LiAlH_4$, the reaction mixture was cooled to 0 °C and followed by addition of 2 ml water and 1.2 ml NaOH (10 wt.%). The precipitate was removed via celite filtration. Subsequently, the solvent was removed under reduced pressure. The crude product was purified by column chromatography (silica, hexane:EtOAc (6:4)) to yield 1.3 g 4-((phenyldiazenyl)phenyl)methanol (45 %) as orange solid after removing of the solvent under reduced pressure and drying in vacuum (10^{-3} mbar).
^{1}H-NMR (400 MHz, DMSO-d_6, 298 K) δ [ppm]: 7.91 – 7.85 (m, 4H), 7.63 – 7.51 (m, 5H), 4.61 (d, J = 4.3 Hz, 2H)

1-(4-(bromomethyl)phenyl)-2-phenyldiazene

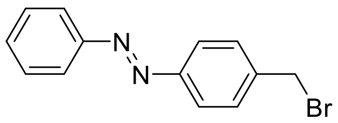

The procedure was adapted from literature.[5] 4-((phenyldiazenyl)phenyl) (1.3 g, 6.1 mmol, 1.0 equiv.) was dissolved in dry THF (30 ml) and precooled to 0 °C. To this solution triphenylphosphine (2.4 g, 9.2 mmol, 1.5 equiv.) and N-bromosuccinimide (1.7 g, 9.2 mmol, 1.5 equiv.) were added in alternating portions. The reaction mixture was stirred at room temperature for 24 h. Afterwards, the precipitate was removed via celite filtration. The solvent was removed under reduced pressure and the crude was purified by column chromatography (silica, hexane:EtOAc (8:2)). After solvent removal under reduced pressure and drying in vacuum (10^{-3} mbar) 1.4 g 1-(4-(bromomethyl)phenyl)-2-phenyldiazene (83 %) was obtained as orange solid.

^{1}H-NMR (400 MHz, DMSO-d_6, 298 K) δ [ppm]: 7.94 – 7.85 (m, 4H), 7.73 – 7.56 (m, 5H), 4.81 (s, 2H).

2-(Phenyldiazenyl)imidazole

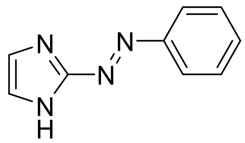

The synthesis was adapted and modified from existing literature.[6] A solution of $NaNO_2$ (5.27 g, 76.35 mmol, 1.05 equiv.) in water (30 mL) was added dropwise to a precooled solution of aniline (6.64 mL, 72.72 mmol, 1.0 equiv.) and 6M HCl (43.63 mL, 261.78 mmol, 3.6 equiv.) and stirred for 30 minutes. The diazotized solution was added dropwise to a precooled solution of imidazole (5.00 g, 73.44 mmol, 1.01 equiv.), $NaHCO_3$ (25.05 g, 298.14 mmol, 4.1 equiv.) in water (110 mL). The reaction mixture was stirred overnight at 0 °C. The precipitate was isolated by centrifugation, extracted with 2M HCl (3 x 30 ml), and reprecipitated by neutralization to pH 7 with a saturated Na_2CO_3 solution. The final product was washed with acetone (3 x 40 ml) and dried under vacuum (10^{-3} mbar) yielding 5.70 g (45.5%) of 2-(phenyldiazenyl))imidazole as yellow solid.

^{1}H-NMR (400 MHz, DMSO-d_6, 298 K) δ [ppm]: 7.86 (m, 2 H), 7.57 (m, 3 H), 7.36 (s, 2 H). ^{13}C-NMR (101 MHz, DMSO-d_6, 298 K) δ [ppm]: 154.80, 152.06, 131.44, 129.60 (2 C), 125.91, 122.32 (2 C).
*OH proton is not detected due to H/D exchange

3-(4-Hydroxyphenyl(diazenyl))benzenesulfonic acid

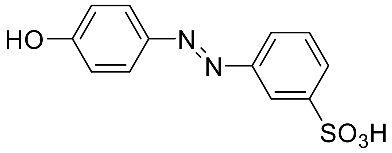

A solution of 3-aminobenzenesulfonic acid (10.00 g, 57.74 mmol, 1.0 equiv.) and 17 ml concentrated HCl (3.6 equiv.) in water (30 ml) was cooled to 0 °C. To this, a solution of $NaNO_2$ (3.98 g, 57.74 mmol, 1.0 equiv.) in water (25 mL) was added, and the reaction mixture was stirred for 10 min. Subsequently, a solution of phenol (5.43 g, 57.74 mmol, 1.0 equiv.), K_2CO_3 (15.96 g, 115.48 mmol, 2.0 equiv.) and NaOH (2.89 g, 72.18 mmol, 1.25 equiv.) in water (150 mL) was added dropwise to the reaction mixture. The reaction was stirred at 0 °C for 4 h. Afterwards, the crude product was precipitated by addition of conc. HCl until pH 2. The solid was collected by vacuum filtration. The crude was recrystallized from EtOH:water (1:1, 75 ml) two times and dried under vacuum (10^{-3} mbar) to yield 9.55 g (59 %) 3-(4-hydroxyphenyl(diazenyl))benzenesulfonic acid as a brown crystalline solid.

^{1}H-NMR (400 MHz, DMSO-d_6, 298 K) δ [ppm]: 10.38 (s, 1 H), 8.02 (t, J = 1.90 Hz, 1 H), 7.82 (m, 3 H), 7.73 (dt, J = 7.58 Hz, J = 1.41 Hz, 1 H), 7.53 (t, J = 7.76 Hz, 1 H), 6.96 (ddd, J = 8.86 Hz, J = 2.99 Hz, J =

1.92 Hz, 2 H). ^{13}C-NMR (101 MHz, DMSO-d_6, 298 K) δ [ppm]: 161.15, 151.57, 149.41, 145.23, 128.92, 127.49, 124.97 (2 C), 124.07, 117.46, 116.03 (2 C).

2-(4-Hydroxyphenyl(diazenyl))benzenesulfonic acid

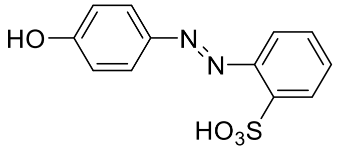

A solution of 2-aminobenzenesulfonic acid (10.00 g, 57.74 mmol, 1.0 equiv.) and 17 ml concentrated HCl (3.6 equiv.) in water (30 ml) was cooled to 0 °C. To this, a solution of $NaNO_2$ (3.98 g, 57.74 mmol, 1.0 equiv.) in water (25 mL) was added, and the reaction mixture was stirred for 10 min. Subsequently, a solution of phenol (5.43 g, 57.74 mmol, 1.0 equiv.), K_2CO_3 (15.96 g, 115.48 mmol, 2.0 equiv.) and NaOH (2.89 g, 72.18 mmol, 1.25 equiv.) in water (150 mL) was added dropwise to the reaction mixture. The reaction was stirred at 0 °C for 4 h. Afterwards, the crude product was precipitated by addition of conc. HCl until pH 2. The solid was collected by vacuum filtration. The crude was recrystallized from EtOH:water (1:1, 7 ml) two times and dried under vacuum (10^{-3} mbar) to yield 8.60 g (54 %) 2-(4-hydroxyphenyl(diazenyl))benzenesulfonic acid as a brown crystalline solid.

^{1}H-NMR (400 MHz, DMSO-d_6, 298 K) δ [ppm]: 10.20 (s, 1 H), 7.89 (dd, J = 6.99 Hz, J = 2.08 Hz, 1 H), 7.80 (ddd, J = 8.75 Hz, J = 2.99 Hz, J = 1.92 Hz, 2 H), 7.39 (tt, J = 7.35 Hz, J = 3.73 Hz, 2 H), 7.32 (dd, J = 7.11 Hz, J = 2.02 Hz, 1 H), 6.92 (ddd, J = 8.75 Hz, J = 2.87 Hz, J = 1.97 Hz, 2 H). ^{13}C-NMR (101 MHz, DMSO-d_6, 298 K) δ [ppm]: 160.52, 149.12, 146.09, 144.55, 129.45, 128.80, 128.11, 125.38 (2 C), 115.58 (2 C), 115.34.

4-(4-Hydroxyphenyl(diazenyl))benzenesulfonic acid

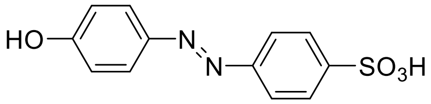

A solution of sulfanilic acid (10.00 g, 57.74 mmol, 1.0 equiv.) and 17 ml concentrated HCl (3.6 equiv.) in water (30 ml) was cooled to 0 °C. To this, a solution of $NaNO_2$ (3.98 g, 57.74 mmol, 1.0 equiv.) in water (25 mL) was added, and the reaction mixture was stirred for 10 min. Subsequently, a solution of phenol (5.43 g, 57.74 mmol, 1.0 equiv.), K_2CO_3 (15.96 g, 115.48 mmol, 2.0 equiv.) and NaOH (2.89 g, 72.18 mmol, 1.25 equiv.) in water (150 mL) was added dropwise to the reaction mixture. The reaction was stirred at 0 °C for 4 h. Afterwards, the crude product was precipitated by addition of conc. HCl until pH 2. The solid was collected by vacuum filtration. The crude was recrystallized from EtOH:water (1:1, 240 ml) two times and dried under vacuum (10^{-3} mbar) to yield 13.92 g (87 %) 4-(4-hydroxyphenyl(diazenyl))benzenesulfonic acid as a brown crystalline solid.

^{1}H-NMR (400 MHz, DMSO-d_6, 298 K) δ [ppm]: 10.36 (s, 1H), 7.83 – 7.79 (m, 2H), 7.76 (s, 4H), 6.99 – 6.91 (m, 2H). ^{13}C-NMR (101 MHz, DMSO-d_6, 298 K) δ [ppm]: 161.20, 151.93, 149.63, 145.28, 126.72 (2C), 124.99 (2C), 121.67 (2C), 116.06 (2C).

1.2.4. Bromoethoxy-functionalized diphenyldiazenes

1-(4-(2-Bromoethoxy)phenyl)-2-phenyldiazene

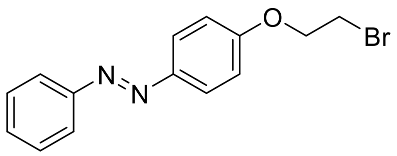

4-(phenyldiazenyl)phenol (7.00 g, 35.3 mmol, 1.0 equiv.) and dibromoethane (11.86 mL, 137.61 mmol, 5.0 equiv.) were dissolved in 200 mL acetonitrile. After addition of potassium carbonate (9.76 g, 70.6 mmol, 2.0 equiv.) and 18-crown-6 (0.19 g, 0.71 mmol, 0.02 equiv.) the reaction mixture was refluxed for 24 h under inert conditions. Afterwards, the solvent was removed under reduced pressure. The crude product was purified by column chromatography (silica, EtOAc:hexane (1:50)). The product fractions (monitored via thin layer chromatography) were combined. The solvent was removed and the product dried in vacuum (10^{-3} mbar) to yield 6.44 g (60 %) 1-(4-(2-bromoethoxy)phenyl)-2-phenyldiazene as orange solid.
^{1}H-NMR (400 MHz, DMSO-d_6, 298 K) δ [ppm]: 7.93 – 7.87 (m, 2H), 7.87 – 7.83 (m, 2H), 7.63 – 7.50 (m, 3H), 7.22 – 7.13 (m, 2H), 4.45 (t, *J* = 6.3 Hz, 2H), 3.86 (t, *J* = 6.3 Hz, 2H).

1-(4-(2-Bromoethoxy)phenyl)-2-(4-butylphenyl)diazene

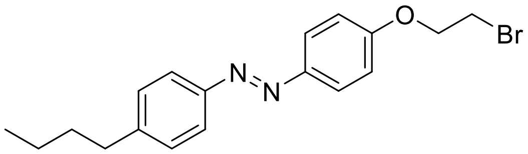

4-((4-butylphenyl)diazenyl)phenol (7.00 g, 27.5 mmol, 1.0 equiv.) and dibromoethane (11.86 mL, 137.6 mmol, 5.0 equiv.) were dissolved in 200 mL acetonitrile. After addition of potassium carbonate (7.61 g, 55.0 mmol, 2.0 equiv.) and 18-crown-6 (0.15 g, 0.55 mmol, 0.02 equiv.) the reaction mixture was refluxed for 24 h under inert conditions. Afterwards, the solvent was removed under reduced pressure. The crude product was purified by column chromatography (silica, EtOAc:hexane (1:50)). The product fractions (monitored via thin layer chromatography) were combined. The solvent was removed and the product dried in vacuum (10^{-3} mbar) to yield 5.50 g (55%) 1-(4-(2-bromoethoxy)phenyl)-2-(4-butylphenyl)diazene as orange solid.
^{1}H-NMR (400 MHz, DMSO-d_6, 298 K) δ [ppm]: 7.91 – 7.83 (m, 2H), 7.80 – 7.74 (m, 2H), 7.43 –7.36 (m, 2H), 7.20 – 7.11 (m, 2H), 4.48 – 4.40 (m, 2H), 3.89 – 3.82 (m, 2H), 2.67 (t, J = 7.7 Hz, 2H), 1.66 – 1.54 (m, 2H), 1.40 – 1.26 (m, 2H), 0.91 (t, J = 7.3 Hz, 3H).

1-(4-((6-Bromohexyl)oxy)phenyl)-2-(4-butylphenyl)diazene

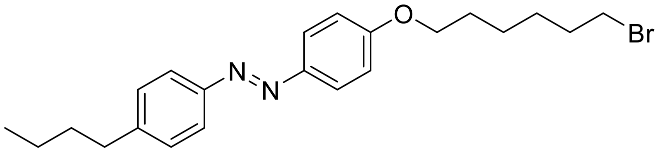

4-((4-butylphenyl)diazenyl)phenol (6.00 g, 23.6 mmol, 1.0 equiv.) and dibromohexane (18.25 mL, 118.0 mmol, 5.0 equiv.) were dissolved in 200 mL acetonitrile. After addition of potassium carbonate (6.52 g, 47.2 mmol, 2.0 equiv.) and 18-crown-6 (0.12 g, 0.47 mmol, 0.02 equiv.) the reaction mixture was refluxed for 24 h under inert conditions. Afterwards, the solvent was removed under reduced pressure. The crude

product was purified by column chromatography (silica, EtOAc:hexane (1:50)). The product fractions (monitored via thin layer chromatography) were combined. The solvent was removed and the product dried in vacuum (10^{-3} mbar) to yield 7.58 g (77%) 1-(4-((6-bromohexyl)oxy)phenyl)-2-(4-butylphenyl)diazene as orange solid.
^{1}H-NMR (400 MHz, DMSO-d_6, 298 K) δ [ppm]: 7.88 – 7.80 (m, 2H), 7.79 – 7.72 (m, 2H), 7.41 – 7.34 (m, 2H), 7.15 – 7.07 (m, 2H), 4.07 (t, J = 6.4 Hz, 2H), 3.54 (t, J = 6.7 Hz, 2H), 2.66 (t, J = 7.7 Hz, 2H), 1.87 – 1.71 (m, 4H), 1.65 – 1.53 (m, 2H), 1.49 – 1.41 (m, 4H), 1.37 – 1.27 (m, 2H), 0.93 – 0.88 (t, 3H, CH3).

1,2-bis(4-(2-bromoethoxy)phenyl)diazene

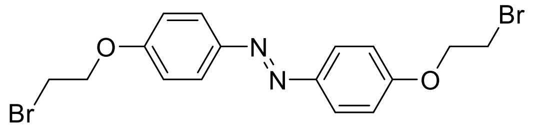

4,4'-(diazene)diphenol (1.91 g, 8.9 mmol, 1.0 equiv.) and dibromoethane (16.75 g, 89.2 mmol, 10.0 equiv.) were dissolved in 75 mL acetonitrile. After addition of potassium carbonate (4.93 g, 35.7 mmol, 4.0 equiv.) and 18-crown-6 (0.05 g, 0.18 mmol, 0.02 equiv.) the reaction mixture was refluxed for 48 h under inert conditions. Afterwards, the solvent was removed under reduced pressure. The crude product was purified by column chromatography (silica, EtOAc:hexane (1:50)). The product fractions (monitored via thin layer chromatography) were combined. The solvent was removed and the product dried in vacuum (10^{-3} mbar) to yield 0.43 g (11 %) 1,2-bis(4-(2-bromoethoxy)phenyl)diazene as orange solid.
^{1}H-NMR (400 MHz, DMSO-d_6, 298 K) δ [ppm]: 7.84 (s, 4H), 7.16 (s, 4H), 4.43 (t, 4H), 3.85 (t, 4H).

1.2.5. Azobenzene-functionalized potassium benzenesulfonates

Potassium 3-((4-(2-chloroethoxy)phenyl)diazenyl)benzenesulfonate

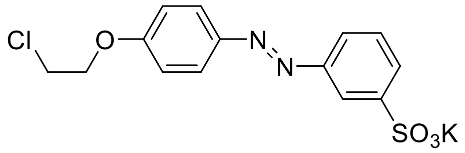

3-(4-Hydroxyphenyl(diazenyl))benzenesulfonic acid (2.00 g, 7.19 mmol, 1.0 equiv.) and 1-bromo-2-chloroethane (1.97 mL, 21.56 mmol, 3.0 equiv.) were suspended in dry DMF (100 ml) under inert conditions. After K_2CO_3 (1.99 g, 14.37 mmol, 2.0 equiv.) and crown ether (0.04 mg, 0.14 mmol, 0.02 equiv.) were added, the solution was degassed three times and stirred for 4 h at 75 °C. After reaction, the solvent was removed under reduced pressure. The crude was purified by reversed-phase column chromatography (C_{18} silica, water → water:MeCN (9:1)). Subsequently, the solvent was removed under reduced pressure and dried under vacuum (10^{-3} mbar) to yield 0.76 g (28 %) potassium 3-((4-(2-chloroethoxy)phenyl)diazenyl)benzenesulfonate as yellow powder.
^{1}H-NMR (400 MHz, DMSO-d_6, 298 K) δ [ppm]: 8.04 (t, J = 1.96 Hz, 1 H), 7.93 (d, J = 8.80 Hz, 2 H), 7.85 (d, J = 7.83 Hz, 1 H), 7.75 (d, J = 7.58 Hz, 1 H), 7.55 (t, J = 7.76 Hz, 1 H), 7.18 (d, J = 8.93 Hz, 2 H), 4.38 (t, J = 5.07 Hz, 2 H), 4.00 (t, J = 5.07 Hz, 2 H). ^{13}C-NMR (101 MHz, DMSO-d_6, 298 K) δ [ppm]: 160.86, 151.46, 149.53, 146.43, 128.99, 127.90, 124.72 (2 C), 124.29, 117.54, 115.31 (2 C), 68.40, 43.03.

Potassium 2-((4-(2-bromoethoxy)phenyl)diazenyl)benzenesulfonate

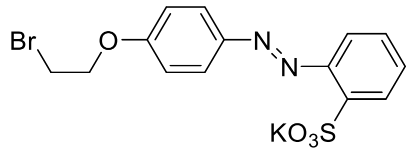

2-(4-Hydroxyphenyl(diazenyl))benzenesulfonic acid (2.00 g, 7.19 mmol, 1.0 equiv.) and 1,2-dibromoethane (4.05 g, 21.56 mmol, 3.0 equiv.) were suspended in dry DMF (100 ml) under inert conditions. After K_2CO_3 (1.99 g, 14.37 mmol, 2.0 equiv.) and crown ether (0.04 mg, 0.14 mmol, 0.02 equiv.) were added, the solution was degassed three times and stirred for 4 h at 75 °C. After reaction, the solvent was removed under reduced pressure. The crude was purified by reversed-phase column chromatography (C_{18} silica, water → water:MeCN (9:1)). Subsequently, the solvent was removed under reduced pressure and dried under vacuum (10^{-3} mbar) to yield 0.80 g (29 %) potassium 2-((4-(2-bromoethoxy)phenyl)diazenyl)benzenesulfonate as red powder.
^{1}H-NMR (400 MHz, DMSO-d_6, 298 K) δ [ppm]: 7.91 (m, 3 H), 7.42 (dt, J = 5.99 Hz, J = 2.63 Hz, 2 H), 7.35 (m, 1 H), 7.16 (m, 2 H), 4.44 (t, J = 5.38 Hz, 2 H), 3.86 (t, J = 5.38 Hz, 2 H). ^{13}C-NMR (101 MHz, DMSO-d_6, 298 K) δ [ppm]: 160.29, 148.97, 147.27, 144.77, 129.49, 129.22, 128.15, 125.15 (2 C), 115.27 (2 C), 114.94, 68.13, 31.32.
*Note 1-bromo-2-chloroethane is recommended instead of 1,2-dibromoethane as the chlorine derivative reduces the amount of dimer formed as byproduct.

potassium 4-((4-(2-chloroethoxy)phenyl)diazenyl)benzenesulfonate

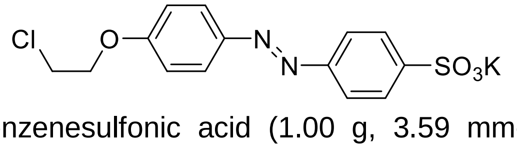

3-(4-Hydroxyphenyl(diazenyl))benzenesulfonic acid (1.00 g, 3.59 mmol, 1.0 equiv.) and 1-bromo-2-chloroethane (1.50 mL, 17.97 mmol, 5.0 equiv.) were suspended in dry DMF (100 ml) under inert conditions. After K_2CO_3 (1.84 g, 13.30 mmol, 3.7 equiv.) and crown ether (0.03 mg, 0.11 mmol, 0.03 equiv.) were added, the solution was degassed three times and stirred for 4 h at 75 °C. After reaction, the solvent was removed under reduced pressure. The crude was purified by reversed-phase column chromatography

(C_{18} silica, water → water:MeCN (9:1)). Subsequently, the solvent was removed under reduced pressure and dried under vacuum (10^{-3} mbar) to yield 0.50 g (37 %) potassium 4-((4-(2-chloroethoxy)phenyl)diazenyl)benzenesulfonate as orange powder.
^{1}H-NMR (400 MHz, DMSO-d_6, 298 K) δ [ppm]: 7.95 – 7.90 (m, 2H), 7.84 – 7.73 (m, 4H), 7.22 – 7.13 (m, 2H), 4.39 (t, *J* = 5.1 Hz, 2H), 4.00 (t, *J* = 5.1 Hz, 2H). ^{13}C-NMR (101 MHz, DMSO-d_6, 298 K) δ [ppm]: 161.31, 152.02, 149.53, 145.21, 126.65(2C), 124.92(2C), 121.59(2C), 116.02 (2C), 68.51, 37.02.

1.2.6. Azobenzene-functionalized imidazolium ammonium halides

1-Methyl-3-(2-(4-(phenyldiazenyl)phenoxy)ethyl)imidazolium bromide

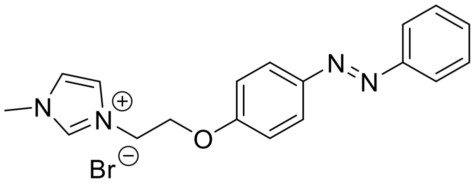

1-(4-(2-bromoethoxy)phenyl)-2-phenyldiazene (2.40 g, 7.9 mmol, 1.0 equiv.) was dissolved in 50 ml dry MeCN. After addition of imidazole (3.23 g, 39.3 mmol, 5.0 equiv.) the reaction was stirred at 75 °C under inert conditions for 72 h. Afterwards the solvent and excessive imidazole were removed under reduced pressure. The crude was dissolved in little amounts of DCM and precipitated in hexane. This process was repeated before the crude is washed with hexane (3 x 30 ml). The product is dried under vacuum (10^{-3} mbar) to yield 2.89 g (95 %) 1-methyl-3-(2-(4-(phenyldiazenyl)phenoxy)ethyl)imidazolium bromide as orange solid.

^{1}H-NMR (400 MHz, DMSO-d_6, 298 K) δ [ppm]: 9.24 (s, 1H), 7.91 (d, *J* = 8.6 Hz, 2H), 7.88 – 7.81 (m, 3H), 7.81 – 7.71 (m, 1H), 7.57 (m, 3H), 7.18 (d, *J* = 8.6 Hz, 2H), 4.66 (t, *J* = 4.8 Hz, 2H), 4.49 (t, *J* = 4.9 Hz, 2H), 3.89 (s, 3H).

Elemental analysis calcd. (%) for $C_{18}H_{19}BrN_4O$: C 55.82, H 4.95, N 14.47; found C 55.13, H 4.78, N 14.24.

1-octyl-3-(2-(4-(phenyldiazenyl)phenoxy)ethyl)imidazolium bromide

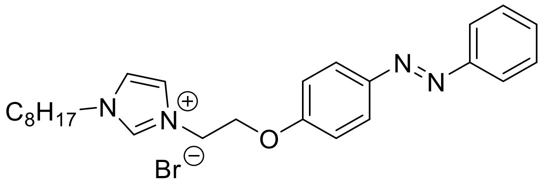

1-(4-(2-bromoethoxy)phenyl)-2-phenyldiazene (2.40 g, 7.9 mmol, 1.0 equiv.) was dissolved in 50 ml dry MeCN. After addition of 1-octylimidazole (7.09 g, 39.3 mmol, 5.0 equiv.) the reaction was stirred at 75 °C under inert conditions for 72 h. Afterwards the solvent and excessive imidazole were removed under reduced pressure. The crude was dissolved in little amounts of DCM and precipitated in hexane. This process was repeated before the crude is washed with hexane (3 x 30 ml). The product is dried under vacuum (10^{-3} mbar) to yield 3.82 g (93 %) octyl-3-(2-(4-(phenyldiazenyl)phenoxy)ethyl)imidazolium bromide as orange solid.

^{1}H-NMR (400 MHz, DMSO-d_6, 298 K) δ [ppm]: 9.29 (s, 1H), 7.92 – 7.90 (m, 1H), 7.90 – 7.88 (m, 1H), 7.88 – 7.86 (m, 1H), 7.86 – 7.84 (m, 1H), 7.84 – 7.81 (m, 2H), 7.61 – 7.58 (m, 1H), 7.58 – 7.50 (m, 2H), 7.17 – 7.12 (m, 2H), 4.65 (t, J = 4.8 Hz, 2H), 4.51 – 4.47 (m, 2H), 1.20 (d, J = 11.2 Hz, 14H), 0.83 – 0.78 (m, 3H).

Elemental analysis calcd. (%) for $C_{25}H_{33}BrN_4O$: C 61.85, H 6.85, N 11.54; found: C 61.14, H 6.69, N 11.33.

3-(2-(4-((4-Butylphenyl)diazenyl)phenoxy)ethyl)methylimidazolium bromide

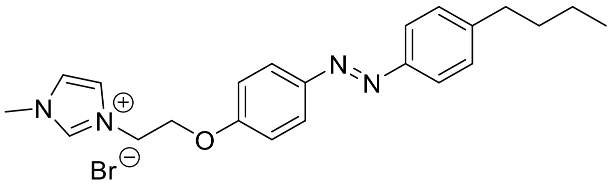

1-(4-(2-Bromoethoxy)phenyl)-2-(4-butylphenyl)diazene (1.45 g, 4.0 mmol, 1.0 equiv.) was dissolved in 25 ml dry MeCN. After addition of imidazole (1.65 g, 20.0 mmol, 5.0 equiv.) the reaction was stirred at 75 °C under inert conditions for 72 h. Afterwards the solvent and excessive imidazole were removed under

reduced pressure. The crude was dissolved in little amounts of DCM and precipitated in hexane. This process was repeated before the crude is washed with hexane (3 x 30 ml). The product is dried under vacuum (10^{-3} mbar) to yield 1.56 g (87 %) 3-(2-(4-((4-butylphenyl)diazenyl)phenoxy)ethyl)methylimidazolium bromide as orange solid.

^{1}H-NMR (400 MHz, DMSO-d_6, 298 K) δ [ppm]: 9.23 (s, 1H), 7.90 – 7.87 (m, 1H), 7.87 – 7.84 (m, 2H), 7.79 – 7.74 (m, 1H), 7.77 – 7.71 (m, 2H), 7.38 (d, J = 8.4 Hz, 2H), 7.20 – 7.12 (m, 2H), 4.65 (t, J = 4.8 Hz, 2H), 4.47 (t, J = 4.9 Hz, 2H), 3.88 (s, 3H), 2.66 (t, J = 7.7 Hz, 2H), 1.65 – 1.47 (m, 2H), 1.39 - 1.24 (m, 2H), 0.90 (t, J = 7.4 Hz, 3H).

Elemental analysis calcd. (%) for $C_{22}H_{27}BrN_4O$: C 59.60, H 6.14, N 12.64; found: C 58,73, H 6.14, N 12.44.

3-(2-(4-((4-butylphenyl)diazenyl)phenoxy)ethyl)-1-octylimidazolium bromide

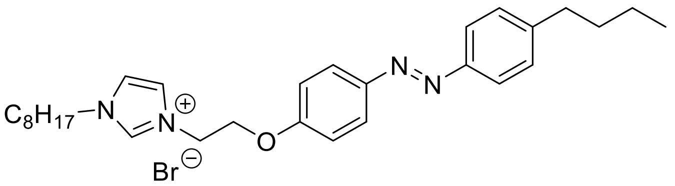

1-(4-(2-Bromoethoxy)phenyl)-2-(4-butylphenyl)diazene (1.07 g, 3.0 mmol, 1.0 equiv.) was dissolved in 25 ml dry MeCN. After addition of 1-octylimidazole (2.67 g, 14.8 mmol, 5.0 equiv.) the reaction was stirred at 75 °C under inert conditions for 72 h. Afterwards the solvent and excessive imidazole were removed under reduced pressure. The crude was dissolved in little amounts of DCM and precipitated in hexane. This process was repeated before the crude is washed with hexane (3 x 30 ml). The product is dried under vacuum (10^{-3} mbar) to yield 1.54 g (96 %) 3-(2-(4-((4-butylphenyl)diazenyl)phenoxy)ethyl)-1-octylimidazolium bromide as orange solid.

^{1}H-NMR (400 MHz, DMSO-d_6, 298 K) δ [ppm]: 9.29 (s, 1H), 7.91 – 7.86 (m, 2H), 7.86 – 7.84 (m, 1H), 7.82 (s, 1H), 7.78 – 7.76 (m, 1H), 7.76 – 7.74 (m, 1H), 7.42 – 7.36 (m, 2H), 7.18 – 7.10 (m, 2H), 4.19 (t, J = 7.1 Hz, 2H), 1.77 (q, J = 7.2 Hz, 2H), 1.59 (p, J = 7.5 Hz, 2H), 1.36 – 1.29 (m, 2H), 1.26 – 1.15 (m, 12H), 0.91 (t, J = 7.4 Hz, 3H), 0.80 (t, 3H).

Elemental analysis calcd. (%) for $C_{29}H_{41}BrN_4O$: C 64.32, H 7.63, N 10.35; found: C 63.61, H 7.66, N 10.11.

2-(4-((4-butylphenyl)diazenyl)phenoxy)-N-(hydroxymethyl)-N,N-dimethylethan-1-aminium bromide

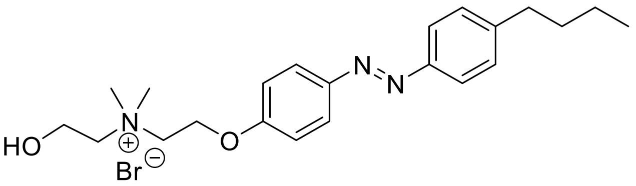

1-(4-(2-Bromoethoxy)phenyl)-2-(4-butylphenyl)diazene (1.40 g, 3.9 mmol, 1.0 equiv.) was dissolved in 25 ml dry MeCN. After addition of 2-(dimethylamino)ethan-1-ol (1.73 g, 19.4 mmol, 5.0 equiv.) the reaction was stirred at 75 °C under inert conditions for 72 h. Afterwards the solvent and excessive 2-(dimethylamino)ethan-1-ol were removed under reduced pressure. The crude was dissolved in little amounts of DCM and precipitated in Et_2O. This process was repeated before the crude is washed with Et_2O (3 x 30 ml). The product is dried under vacuum (10^{-3} mbar) to yield 1.93 g (93 %) 2-(4-((4-butylphenyl)diazenyl)phenoxy)-N-(hydroxymethyl)-N,N-dimethylethan-1-aminium bromide as orange solid.

^{1}H-NMR (400 MHz, DMSO-d_6, 298 K) δ [ppm]: 7.91 – 7.83 (m, 2H), 7.78 – 7.71 (m, 2H), 7.40 – 7.32 (m, 2H), 7.21 – 7.13 (m, 2H), 5.32 (t, J = 4.9 Hz, 1H), 4.59 – 4.52 (m, 2H), 3.91 – 3.83 (m, 4H), 3.58 – 3.52 (m, 2H), 3.20 (s, 6H), 2.63 (t, J = 7.7 Hz, 2H), 1.61 – 1.50 (m, 2H), 1.29 (h, J = 7.4 Hz, 2H), 0.87 (t, J = 7.3 Hz, 3H).

Elemental analysis calcd. (%) for $C_{21}H_{30}BrN_3O_2$: C 58.67, H 7.16, N 9.33; found: C 58.05, H 7.13, N 9.17.

3-(6-(4-((4-butylphenyl)diazenyl)phenoxy)hexyl)-1-methylimidazolium bromide

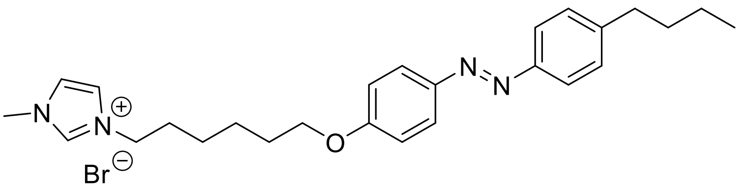

1-(4-((6-Bromohexyl)oxy)phenyl)-2-(4-butylphenyl)diazene (1.50 g, 3.6 mmol, 1.0 equiv.) was dissolved in 25 ml dry MeCN. After addition of 1-methylimidazole (2.95 g, 36.0 mmol, 10.0 equiv.) the reaction was stirred at 75 °C under inert conditions for 72 h. Afterwards the solvent and excessive imidazole were removed under reduced pressure. The crude was dissolved in little amounts of DCM and precipitated in Et_2O. This process was repeated before the crude is washed with Et_2O (3 x 30 ml). The product is dried under vacuum (10^{-3} mbar) to yield 1.57 g (87 %) 3-(6-(4-((4-butylphenyl)diazenyl)phenoxy)hexyl)-1-methylimidazolium bromide as orange solid.

^{1}H-NMR (400 MHz, DMSO-d_6, 298 K) δ [ppm]: 9.13 (s, 1H), 7.92 – 7.87 (m, 1H), 7.87 – 7.83 (m, 1H), 7.81 – 7.78 (m, 1H), 7.78 – 7.73 (m, 2H), 7.73 – 7.69 (m, 1H), 7.43 – 7.36 (m, 2H), 7.18 – 7.07 (m, 2H), 4.18 (t, J = 7.2 Hz, 2H), 4.08 (t, J = 6.4 Hz, 2H), 3.85 (s, 3H), 2.71 – 2.63 (m, 2H), 1.89 – 1.70 (m, 4H), 1.70 – 1.54 (m, 2H), 1.54 – 1.42 (m, 2H), 1.39 – 1.28 (m, 4H), 0.92 (t, J = 7.3 Hz, 3H).

Elemental analysis calcd. (%) for $C_{33}H_{49}BrN_4O$: C 62.52, H 7.06, N 11.22; found: C 61.01, H 7.27, N 10.96.

3-(6-(4-((4-butylphenyl)diazenyl)phenoxy)hexyl)-1-octylimidazolium bromide

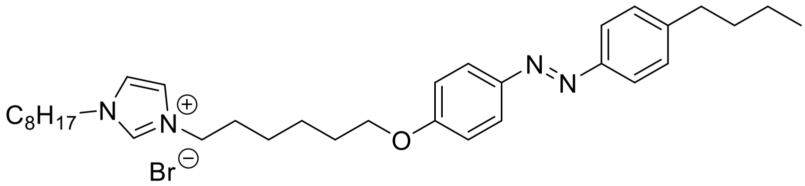

1-(4-((6-Bromohexyl)oxy)phenyl)-2-(4-butylphenyl)diazene (1.50 g, 3.6 mmol, 1.0 equiv.) was dissolved in 25 ml dry MeCN. After addition of 1-octylimidazole (3.24 g, 18.0 mmol, 5.0 equiv.) the reaction was stirred at 75 °C under inert conditions for 72 h. Afterwards the solvent and excessive imidazole were removed under reduced pressure. The crude was dissolved in little amounts of DCM and precipitated in Et_2O. This process was repeated before the crude is washed with Et_2O (3 x 30 ml). The product is dried under vacuum (10^{-3} mbar) to yield 1.92 g (89 %) 3-(6-(4-((4-butylphenyl)diazenyl)phenoxy)hexyl)-1-methylimidazolium bromide as orange solid.

^{1}H-NMR (400 MHz, DMSO-d_6, 298 K) δ [ppm]: 9.24 (s, 1H), 7.89 – 7.86 (m, 1H), 7.86 – 7.84 (m, 1H), 7.83 – 7.80 (m, 2H), 7.78 – 7.76 (m, 1H), 7.76 – 7.73 (m, 1H), 7.42 – 7.35 (m, 2H), 7.13 – 7.06 (m, 2H), 4.23 – 4.16 (m, 2H), 4.07 (t, J = 6.4 Hz, 2H), 2.66 (t, J = 7.7 Hz, 2H), 1.88 – 1.80 (m, 2H), 1.79 – 1.72 (m, 4H), 1.63 – 1.56 (m, 2H), 1.49 – 1.42 (m, 2H), 1.39 – 1.29 (m, 4H), 1.27 – 1.16 (m, 12H), 0.91 (t, J = 7.3 Hz, 3H), 0.86 – 0.81 (m, 3H).

Elemental analysis calcd. (%) for $C_{33}H_{49}BrN_4O$: C 66.32, H 8.26, N 9.37; found: C 64.95, H 8.39, N 9.98.

3,3'-(((diazene-1,2-diylbis(4,1-phenylene))bis(oxy))bis(ethane-2,1-diyl))bis(1-methylimidazolium) bromide

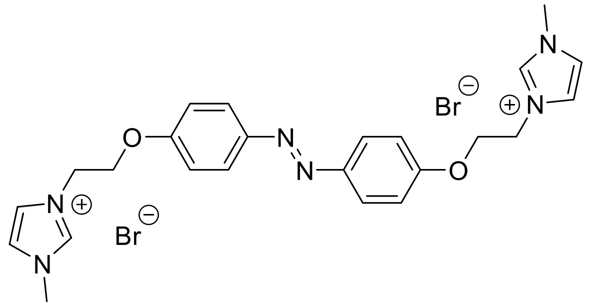

1,2-bis(4-(2-bromoethoxy)phenyl)diazene (0.34 g, 0.8 mmol, 1.0 equiv.) was dissolved in 30 ml dry MeCN. After addition of 1-methylimidazole (0.65 g, 7.9 mmol, 10.0 equiv.) the reaction was stirred at 75 °C under inert conditions for 72 h. Afterwards the solvent and excessive imidazole were removed under reduced pressure. The crude was dissolved in little amounts of DCM and precipitated in Et_2O. This process was repeated before the crude is washed with Et_2O (3 x 30 ml). The product is dried under vacuum (10^{-3} mbar) to yield 0.35 g (74 %) 3,3'-(((diazene-1,2-diylbis(4,1-phenylene))bis(oxy))bis(ethane-2,1-diyl))bis(1-methylimidazolium) bromide as orange solid.

^{1}H-NMR (400 MHz, DMSO-d$_6$, 298 K) δ [ppm]: 9.20 (s, 2H), 7.86 (d, J = 1.9 Hz, 2H), 7.84 (d, J = 1.3 Hz, 4H), 7.73 (t, J = 1.8 Hz, 2H), 7.17 – 7.13 (m, 4H), 4.64 (t, J = 4.9 Hz, 4H), 4.46 (t, J = 5.0 Hz, 4H), 3.89 (s, 6H).

Elemental analysis calcd. (%) for $C_{24}H_{28}Br_2N_6O_2$: C 48.67, H 4.76, N 14.19; found: C 48.03, H 4.88, N 13.92.

1-Methyl-3-(4-(phenyldiazenyl)benzyl)imidazolium bromide

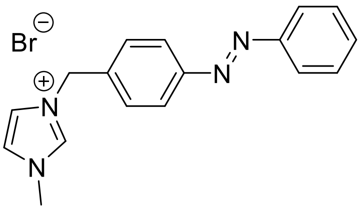

1-(4-(bromomethyl)phenyl)-2-phenyldiazene (0.97 g, 3.54 mmol, 1.0 equiv.) was dissolved in 25 mL dry acetonitrile. 1-Methylimidazole (1.45 g, 17.70 mmol, 5.0 equiv.) was added and the reaction was stirred at 75 °C under inert conditions. Afterwards, the solvent and excessive imidazole were removed under reduced pressure. The crude was dissolved in a small amount DCM and precipitated in Et_2O. The precipitate was washed with Et_2O (3 x 30 ml) and dried under vacuum (10^{-3} mbar) to yield 1.05 g 1-Methyl-3-(4-(phenyldiazenyl)benzyl)imidazolium bromide (87 %).

^{1}H-NMR (400 MHz, $CDCl_3$, 298 K) δ [ppm]: 10.67 (d, J = 1.7 Hz, 1H), 7.94 – 7.89 (m, 4H), 7.71 – 7.66 (m, 2H), 7.54 – 7.50 (m, 3H), 7.38 (dt, J = 9.6, 1.8 Hz, 2H), 5.75 (s, 2H), 4.10 (s, 3H). ^{13}C-NMR (101 MHz, $CDCl_3$, 298 K) δ [ppm]: 153.05, 152.42, 137.95, 135.32, 131.55, 129.94, 129.17, 123.76, 123.35, 123.05, 121.88, 52.97, 36.89.

Elemental analysis calcd. (%) for $C_{13}H_{11}BrN_2$: C 57.15, H 4.80, N 15.68; found: C 56.51, H 4.62, N 15.39

1,3-Dimethyl-2-(phenyldiazenyl)imidazolium iodide

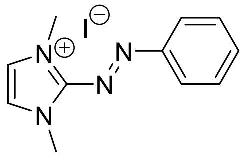

2-Phenylazoimidazole (3.00 g, 17.42 mmol, 1.0 equiv.) were dissolved in dry MeOH (50 mL). K_2CO_3 (2.65 g, 19.16 mmol, 1.1 equiv.) and methyl iodide (13.02 mL, 209.07 mmol, 12.0 equiv.) were added and the reaction was refluxed for 24 h at 70 °C under inert conditions.* Afterwards, the solvent was removed under reduced pressure. The crude product was recrystallized twice from water:EtOH (3:1, 15 ml) and dried under vacuum (10^{-3} mbar) to yield 3.32 g (58.1%) of 1,3-dimethyl-2-(phenylazo)imidazolium iodide as red solid.

^{1}H-NMR (400 MHz, DMSO-d_6, 298 K) δ [ppm]: 8.12 (m, 2 H), 8.05 (m, 2 H), 7.79 (m, 1 H), 7.72 (s, 2 H), 4.11 (s, 6 H). ^{13}C-NMR (101 MHz, DMSO-d_6, 298 K) δ [ppm]: 152.08, 143.11, 135.20, 130.06 (2 C), 124.91 (2 C), 123.90 (2 C), 36.75 (2 C).

Elemental analysis calcd. (%) for $C_{11}H_{13}IN_4$: C 40.26, H 3.99, N 17.07; found 39.86, H 3.88, N 16.91

*Note: A gas wash bottle containing 1M NaOH was connected to quench evaporating methyl iodide.

1.2.7. Azobenzene-functionalized imidazolium and ammonium perrhenates

1-Methyl-3-(2-(4-(phenyldiazenyl)phenoxy)ethyl)imidazolium perrhenate (**1a**)

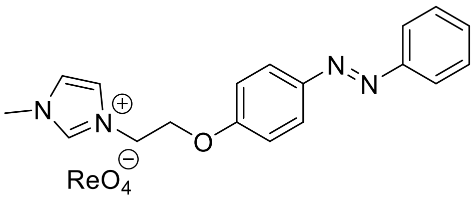

The synthesis was performed according to an anion exchange procedure.[1] The 1-methyl-3-(2-(4-(phenyldiazenyl)phenoxy)ethyl)imidazolium bromide (0.40 g, 1.0 mmol, 1.0 equiv.) was dissolved in 150 mL deionized water and rinsed slowly over 250 g Amberlite IRA 402(Cl), which was activated by 1 M NaOH solution (Cl^- → OH^-). The basic fractions were collected and ammonium perrhenate (NH_4ReO_4, 0.3 g, 1.1 mmol, 1.1 equiv.) was added to solution. The solution was stirred at 80 °C for 2 h. Afterwards, the solution was concentrated to approximately 20 mL under reduced pressure. The residue was extracted with DCM (3x50 mL) and the organic phase was filtered before the solvent was evaporated under reduced pressure. The product was dried in vacuum (10^{-3} mbar) to yield 0.48 g (83 %) 1-methyl-3-(2-(4-(phenyldiazenyl)phenoxy)ethyl)imidazolium perrhenate as orange solid.
^{1}H-NMR (400 MHz, DMSO-d_6, 298 K) δ [ppm]: 9.20 (s, 1H), 7.91 (d, *J* = 8.5 Hz, 2H), 7.85 (d, *J* = 6.7 Hz, 3H), 7.73 (s, 1H), 7.57 (dt, *J* = 12.8, 7.2 Hz, 3H), 7.17 (d, *J* = 8.5 Hz, 2H), 4.65 (t, *J* = 5.0 Hz, 2H), 4.48 (t, *J* = 4.9 Hz, 2H), 3.89 (s, 3H).
Elemental analysis calcd. (%) for $C_{18}H_{19}N_4O_5Re$: C 38.77, H 3.43, N 10.05; found: C 38.66, H 3.24, N 9.76.

3-(2-(4-((4-butylphenyl)diazenyl)phenoxy)ethyl)-1-methylimidazolium perrhenate (**3a**)

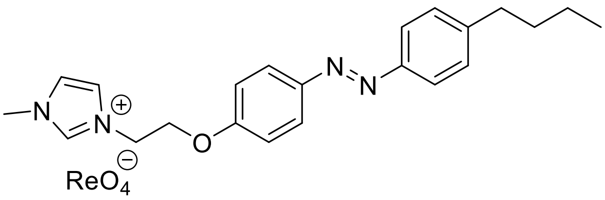

3-(2-(4-((4-butylphenyl)diazenyl)phenoxy)ethyl)-1-methylimidazolium bromide (0.72 g, 1.6 mmol, 1.0 equiv.) was dissolved in 100 mL deionized water and rinsed slowly over 250 g Amberlite IRA 402(Cl), which was activated by 1 M NaOH solution (Cl^- → OH^-). The basic fractions were collected and ammonium perrhenate (NH_4ReO_4, 0.46 g, 1.7 mmol, 1.05 equiv.) was added to solution. The solution was stirred at 80 °C for 2 h. Afterwards, the solution was concentrated to approximately 20 mL under reduced pressure. The residue was extracted with DCM (3x50 mL) and the organic phase was filtered before the solvent was evaporated under reduced pressure. The product was dried in vacuum (10^{-3} mbar) to yield 0.81 g (81 %) 3-(2-(4-((4-butylphenyl)diazenyl)phenoxy)ethyl)-1-methylimidazolium perrhenate as orange solid.
^{1}H-NMR (400 MHz, DMSO-d_6, 298 K) δ [ppm]: 9.20 (s, 1H), 7.92 – 7.88 (m, 1H), 7.88 – 7.87 (m, 1H), 7.85 (t, J = 1.8 Hz, 1H), 7.79 – 7.77 (m, 1H), 7.77 – 7.75 (m, 1H), 7.74 – 7.72 (m, 1H), 7.42 – 7.38 (m, 2H), 7.18 – 7.15 (m, 2H), 4.65 (t, J = 4.9 Hz, 2H), 4.47 (t, J = 4.9 Hz, 2H), 3.89 (s, 3H), 1.64 – 1.56 (m, 2H), 1.40 – 1.28 (m, 2H), 0.92 (t, J = 7.3 Hz, 3H).
Elemental analysis calcd. (%) for $C_{22}H_{27}N_4O_5Re$: C 43.06, H 4.43, N 9.13; found: C 42.97, H 4.07, N 8.86.

2-(4-((4-butylphenyl)diazenyl)phenoxy)-N-(2-hydroxyethyl)-N,N-dimethylethan-1-aminium perrhenate (**5a**)

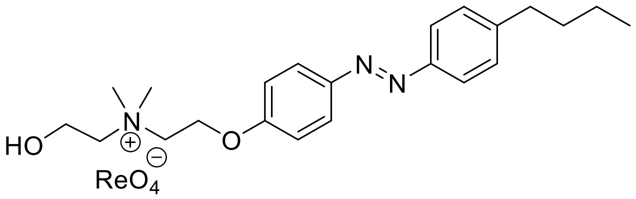

2-(4-((4-butylphenyl)diazenyl)phenoxy)-N-(2-hydroxyethyl)-N,N-dimethylethan-1-aminium bromide (0.86 g, 1.9 mmol, 1.0 equiv.) was dissolved in 200 mL deionized water and rinsed slowly over 200 g Amberlite IRA 402(Cl), which was activated by 1 M NaOH solution ($Cl^- \rightarrow OH^-$). The basic fractions were collected and ammonium perrhenate (NH_4ReO_4, 0.54 g, 2.0 mmol, 1.05 equiv.) was added to solution. The solution was stirred at 80 °C for 2 h. Afterwards, the solution was concentrated to approximately 20 mL under reduced pressure. The residue was extracted with DCM (3x50 mL) and the organic phase was filtered before the solvent was evaporated under reduced pressure. The product was dried in vacuum (10^{-3} mbar) to yield 0.98 g (82 %) 2-(4-((4-butylphenyl)diazenyl)phenoxy)-N-(2-hydroxyethyl)-N,N-dimethylethan-1-aminium perrhenate as orange solid.

^{1}H-NMR (400 MHz, DMSO-d_6, 298 K) δ [ppm]: 7.96 – 7.87 (m, 2H), 7.81 – 7.74 (m, 2H), 7.43 – 7.37 (m, 2H), 7.24 – 7.12 (m, 2H), 5.33 (t, J = 4.8 Hz, 1H), 4.61 – 4.56 (m, 4H), 3.21 (s, 6H), 3.92 – 3.85 (m, 4H), 3.59 – 3.52 (m, 2H), 2.67 (t, J = 7.7 Hz, 2H), 1.65 – 1.55 (m, 2H), 1.33 (h, J = 7.4 Hz, 2H), 0.91 (t, J = 7.4 Hz, 3H)

Elemental analysis calcd. (%) for $C_{22}H_{32}N_3O_6Re$: C 42.57, H 5.20, N 6.77; found: C 42.17, H 4.84, N 6.50.

3-(6-(4-((4-butylphenyl)diazenyl)phenoxy)hexyl)-1-methylimidazolium perrhenate (**6a**)

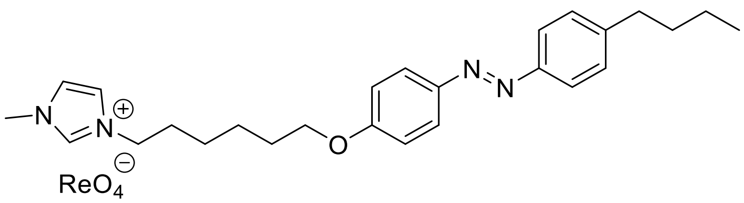

The synthesis was performed according to an anion exchange procedure.[1] The 3-(6-(4-((4-butylphenyl)diazenyl)phenoxy)hexyl)-1-methylimidazolium bromide (0.76 g, 1.5 mmol, 1.0 equiv.) was dissolved in 250 mL deionized water and rinsed slowly over 250 g Amberlite IRA 402(Cl), which was activated by 1 M NaOH solution ($Cl^- \rightarrow OH^-$). The basic fractions were collected and ammonium perrhenate (NH_4ReO_4, 0.43 g, 1.6 mmol, 1.1 equiv.) was added to solution. The solution was stirred at 80 °C for 2 h. Afterwards, the solution was concentrated to approximately 50 mL under reduced pressure. The residue was extracted with DCM (3x50 mL) and the organic phase was filtered before the solvent was evaporated under reduced pressure. The product was dried in vacuum (10^{-3} mbar) to yield 0.85 g (84 %) 3-(6-(4-((4-butylphenyl)diazenyl)phenoxy)hexyl)-1-methylimidazolium perrhenate as orange solid.

^{1}H-NMR (400 MHz, DMSO-d_6, 298 K) δ [ppm]: 9.09 (s, 1H), 7.90 – 7.82 (m, 2H), 7.80 – 7.73 (m, 3H), 7.77 – 7.66 (m, 1H), 7.39 (d, *J* = 8.4 Hz, 2H), 7.18 – 7.06 (m, 2H), 4.17 (t, *J* = 7.2 Hz, 2H), 4.08 (t, *J* = 6.4 Hz, 2H), 3.84 (s, 3H), 2.69 – 2.62 (m, 2H), 1.88 – 1.71 (m, 4H), 1.60 (p, *J* = 7.6 Hz, 2H), 1.47 (p, *J* = 7.3 Hz, 2H), 1.33 (h, *J* = 7.2 Hz, 4H), 0.89 (t, *J* = 7.4 Hz, 3H).

Elemental analysis calcd. (%) for $C_{26}H_{35}N_4O_5Re$: C 46.62, H 5.27, N 8.36; found: C 46.69, H 5.67, N 8.28.

3-(6-(4-((4-butylphenyl)diazenyl)phenoxy)hexyl)-1-octylimidazolium perrhenate (**7a**)

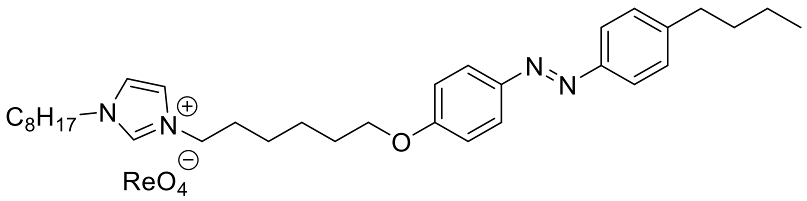

The synthesis was performed according to an anion exchange procedure.[1] The 3-(6-(4-((4-butylphenyl)diazenyl)phenoxy)hexyl)-1-octylimidazolium bromide (1.1 g, 1.8 mmol, 1.0 equiv.) was dissolved in 250 mL deionized water and rinsed slowly over 250 g Amberlite IRA 402(Cl), which was activated by 1 M NaOH solution ($Cl^- \rightarrow OH^-$). The basic fractions were collected and ammonium perrhenate (NH_4ReO_4, 0.44 g, 1.9 mmol, 1.1 equiv.) was added to solution. The solution was stirred at 80 °C for 2 h. Afterwards, the solution was concentrated to approximately 75 mL under reduced pressure. The residue was extracted with DCM (3x50 mL) and the organic phase was filtered before the solvent was evaporated under reduced pressure. The product was dried in vacuum (10^{-3} mbar) to yield 1.30 g (95 %) 3-(6-(4-((4-butylphenyl)diazenyl)phenoxy)hexyl)-1-octylimidazolium perrhenate as orange solid.

^{1}H-NMR (400 MHz, DMSO-d$_6$, 298 K) δ [ppm]: 9.21 – 9.18 (m, 1H), 7.90 – 7.86 (m, 1H), 7.86 – 7.83 (m, 1H), 7.82 – 7.79 (m, 2H), 7.78 – 7.76 (m, 1H), 7.76 – 7.74 (m, 1H), 7.42 – 7.36 (m, 2H), 7.12 – 7.07 (m, 2H), 4.17 – 4.12 (m, 2H), 4.07 (t, J = 6.4 Hz, 2H), 1.83 – 1.70 (m, 6H), 1.64 – 1.57 (m, 2H), 1.51 – 1.44 (m, 2H), 1.39 – 1.30 (m, 4H), 1.28 – 1.16 (m, 14H), 0.92 (t, J = 7.4 Hz, 3H), 0.87 – 0.82 (m, 3H).

Elemental analysis calcd. (%) for $C_{33}H_{49}N_4O_5Re$: C 51.61, H 6.43, N 7.30; found: C 51.38, H 6.39, N 7.28.

1.2.8. Azobenzene-functionalized imidazolium and ammonium tungstates

1-Methyl-3-(2-(4-(phenyldiazenyl)phenoxy)ethyl)imidazolium tungstate (**1b**)

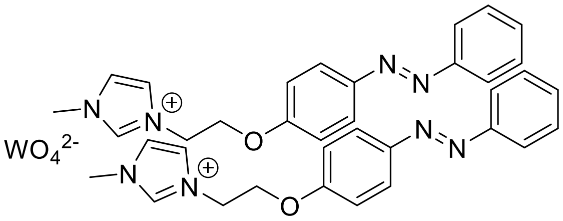

The synthesis was performed according to an anion exchange procedure.[1] 1-Methyl-3-(2-(4-(phenyldiazenyl)phenoxy)ethyl)imidazolium bromide (0.40 g, 1.0 mmol, 2.0 equiv.) was dissolved in 100 mL deionized water and rinsed slowly over 200 g Amberlite IRA 402(Cl), freshly activated by 1 M NaOH solution ($Cl^- \rightarrow OH^-$). The basic fractions were collected and tungstic acid (H_2WO_4, 0.14 g, 0.6 mmol, 1.05 equiv.) was added. The mixture was stirred at room temperature until a pH of 7. Excessive tungstic acid was filtered off and the solvent was removed under reduced pressure. After drying in vacuum (10^{-3} mbar) 0.37 g (82 %) 1-methyl-3-(2-(4-(phenyldiazenyl)phenoxy)ethyl)imidazolium tungstate was obtained as an orange solid.

^{1}H-NMR (400 MHz, DMSO-d$_6$, 298 K) δ [ppm]: 9.38 (s, 2H), 7.93 – 7.85 (m, 4H), 7.85 – 7.79 (m, 6H), 7.72 – 7.67 (m, 2H), 7.61 – 7.47 (m, 6H), 7.21 – 7.13 (m, 4H), 4.67 (t, *J* = 4.8 Hz, 4H), 4.47 (t, *J* = 4.9 Hz, 4H), 3.88 (s, 6H).

Elemental analysis calcd. (%) for $C_{36}H_{38}N_8O_6W$: C 50.13, H 4.44, N 12.99; found: C 48.96, H 4.72, N 12.68.

1-octyl-3-(2-(4-(phenyldiazenyl)phenoxy)ethyl)imidazolium tungstate (**2b**)

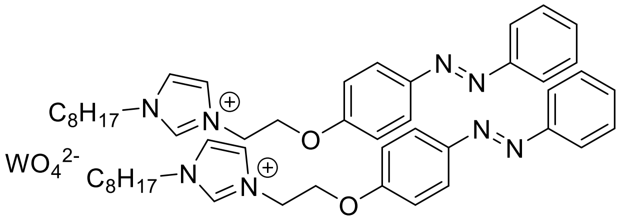

The synthesis was performed according to an anion exchange procedure.[1] The 1-octyl-3-(2-(4-(phenyldiazenyl)phenoxy)ethyl)imidazolium bromide (1.55 g, 2.9 mmol, 2.0 equiv.) was dissolved in 300 mL deionized water and rinsed slowly over 250 g Amberlite IRA 402(Cl), which was activated by 1 M NaOH solution ($Cl^- \rightarrow OH^-$). The basic fractions were collected and tungstic acid (H_2WO_4, 0.38 g, 1.5 mmol, 1.05 equiv.) was added. The mixture was stirred at room temperature until a pH of 7. Excessive tungstic acid was filtered off and the solvent was removed under reduced pressure. After drying in vacuum (10^{-3} mbar) 1.39 g (83 %) 1-octyl-3-(2-(4-(phenyldiazenyl)phenoxy)ethyl)imidazolium tungstate was obtained as an orange solid.

^{1}H-NMR (400 MHz, DMSO-d$_6$, 298 K) δ [ppm]: 9.39 (s, 2H), 7.91 – 7.84 (m, 2H), 7.88 – 7.81 (m, 4H), 7.84 – 7.74 (m, 2H), 7.63 – 7.48 (m, 6H), 7.20 – 7.10 (m, 4H), 4.69 (t, *J* = 4.9 Hz, 4H), 4.51 (t, J = 4.9 Hz, 4H), 1.77 – 1.73 (m, 4H), 1.26 – 1.14 (m, 28H), 0.79 (t, *J* = 6.8 Hz, 6H).

Elemental analysis calcd. (%) for $C_{50}H_{66}N_8O_6W$: C 56.71, H 6.28, N 10.58; found: C 55.14, H 6.39, N 10.23.

3-(2-(4-((4-butylphenyl)diazenyl)phenoxy)ethyl)-1-methylimidazolium tungstate (**3b**)

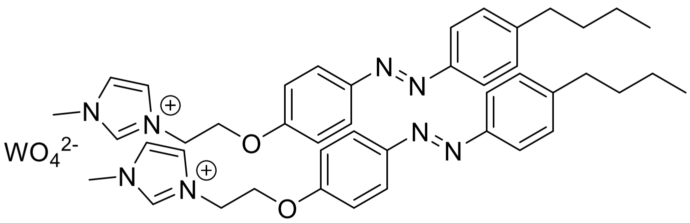

The synthesis was performed according to an anion exchange procedure.[1] The 3-(2-(4-((4-butylphenyl)diazenyl)phenoxy)ethyl)-1-methylimidazolium bromide (0.84 g, 1.9 mmol, 2.0 equiv.) was dissolved in 100 mL deionized water and rinsed slowly over 250 g Amberlite IRA 402(Cl), which was activated by 1 M NaOH solution (Cl^- → OH^-). The basic fractions were collected and tungstic acid (H_2WO_4, 0.25 g, 1.0 mmol, 1.05 equiv.) was added. The mixture was stirred at room temperature until a pH of 7. Excessive tungstic acid was filtered off and the solvent was removed under reduced pressure. After drying in vacuum (10^{-3} mbar) 0.79 g (86 %) 3-(2-(4-((4-butylphenyl)diazenyl)phenoxy)ethyl)-1-methylimidazolium tungstate was obtained as an orange solid.

^{1}H-NMR (400 MHz, DMSO-d_6, 298 K) δ [ppm]: 9.28 (s, 2H), 7.91 – 7.88 (m, 2H), 7.88 – 7.85 (m, 4H), 7.85 – 7.82 (m, 2H), 7.79 – 7.74 (m, 4H), 7.47 – 7.31 (m, 4H), 7.21 – 7.10 (m, 4H), 4.68 – 4.63 (m, 4H), 4.50 – 4.45 (m, 4H), 3.89 (s, 6H), 2.71 – 2.62 (m, 4H), 1.64 – 1.55 (m, 4H), 1.38 – 1.28 (m, 4H), 1.26 – 1.21 (m, 2H), 0.91 (t, J = 7.4 Hz, 6H).

Elemental analysis calcd. (%) for $C_{44}H_{54}N_8O_6W$: C 54.21, H 5.58, N 11.50; found: C 55.24, H 5.83, N 11.49.

3-(2-(4-((4-butylphenyl)diazenyl)phenoxy)ethyl)-1-octylimidazolium tungstate (**4b**)

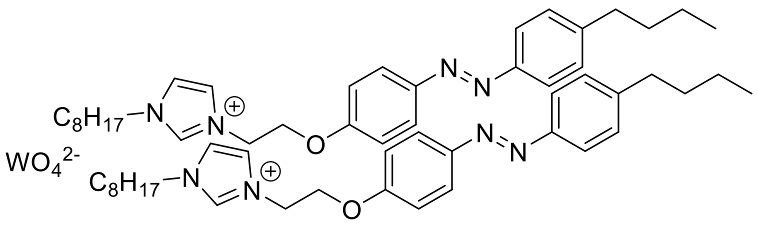

The synthesis was performed according to an anion exchange procedure.[1] The 3-(2-(4-((4-butylphenyl)diazenyl)phenoxy)ethyl)-1-octylimidazolium bromide (1.55 g, 2.9 mmol, 2.0 equiv.) was dissolved in 150 mL deionized water and rinsed slowly over 250 g Amberlite IRA 402(Cl), which was activated by 1 M NaOH solution (Cl^- → OH^-). The basic fractions were collected and tungstic acid (H_2WO_4, 0.38 g, 1.5 mmol, 1.05 equiv.) was added. The mixture was stirred at room temperature until a pH of 7. Excessive tungstic acid was filtered off and the solvent was removed under reduced pressure. After drying in vacuum (10^{-3} mbar) 1.39 g (83 %) 3-(2-(4-((4-butylphenyl)diazenyl)phenoxy)ethyl)-1-octylimidazolium tungstate was obtained as an orange solid.

^{1}H-NMR (400 MHz, DMSO-d_6, 298 K) δ [ppm]: 9.35 (s, 2H), 7.90 – 7.83 (m, 6H), 7.82 – 7.79 (m, 2H), 7.78 – 7.72 (m, 4H), 7.41 – 7.35 (m, 4H), 7.16 – 7.11 (m, 4H), 4.66 (t, *J* = 4.7 Hz, 4H), 4.52 – 4.43 (m, 4H), 2.70 – 2.62 (m, 4H), 1.77 (p, *J* = 7.2 Hz, 4H), 1.65 – 1.53 (m, 4H), 1.39 – 1.26 (m, 4H), 1.23 – 1.15 (m, 24H), 0.91 (t, *J* = 7.3 Hz, 6H), 0.83 (t, 7.1 Hz, 6H).

Elemental analysis calcd. (%) for $C_{58}H_{82}N_8O_6W$: C 59.48, H 7.06, N 9.57; found: C 57.82, H 7.33, N 9.29.

2-(4-((4-butylphenyl)diazenyl)phenoxy)-N-(2-hydroxyethyl)-N,N-dimethylethan-1-aminium tungstate (**5b**)

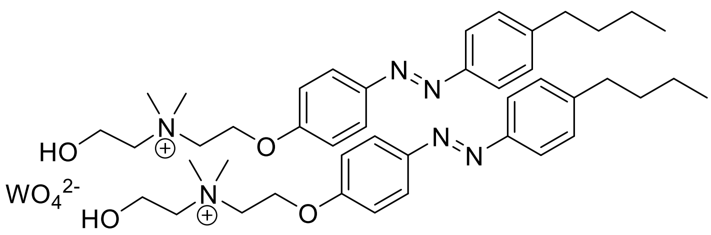

The synthesis was performed according to an anion exchange procedure.[1] 2-(4-((4-butylphenyl)diazenyl)phenoxy)-N-(2-hydroxyethyl)-N,N-dimethylethan-1-aminium bromide (0.89 g, 2.0 mmol, 2.0 equiv.) was dissolved in 200 mL deionized water and rinsed slowly over 200 g Amberlite IRA 402(Cl), freshly activated by 1 M NaOH solution (Cl^- → OH^-). The basic fractions were collected and tungstic acid (H_2WO_4, 0.26 g, 1.04 mmol, 1.05 equiv.) was added. The mixture was stirred at room temperature until a pH of 7. Excessive tungstic acid was filtered off and the solvent was removed under reduced pressure. After drying in vacuum (10^{-3} mbar) 0.86 g (88 %) 2-(4-((4-butylphenyl)diazenyl)phenoxy)-N-(2-hydroxyethyl)-N,N-dimethylethan-1-aminium tungstate was obtained as an orange solid.

^{1}H-NMR (400 MHz, DMSO-d_6, 298 K) δ [ppm]: 7.90 (d, J = 8.9 Hz, 4H), 7.78 (d, J = 8.3 Hz, 4H), 7.39 (d, J = 8.4 Hz, 4H), 7.22 (d, J = 9.0 Hz, 4H), 4.63 – 4.59 (m, 4H), 3.97 – 3.92 (m, 2H), 3.90 – 3.86 (m, 4H), 3.67 – 3.61 (m, 4H), 3.24 (s, 12H), 2.67 (t, J = 7.7 Hz, 4H), 1.66 – 1.48 (m, 4H), 1.34 (dt, J = 14.8, 7.4 Hz, 4H), 0.91 (t, J = 7.4 Hz, 6H).

Elemental analysis calcd. (%) for $C_{44}H_{64}N_6O_8W$: C 53.44, H 6.52, N 8.50; found: C 50.13, H 6.26, N 7.77.

3-(6-(4-((4-butylphenyl)diazenyl)phenoxy)hexyl)-1-methylimidazolium tungstate (**6b**)

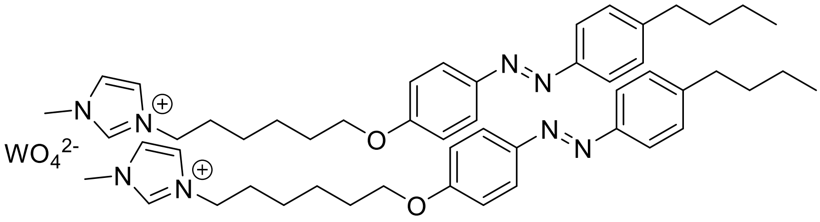

The synthesis was performed according to an anion exchange procedure.[1] 3-(6-(4-((4-butylphenyl)diazenyl)phenoxy)hexyl)-1-methylimidazolium bromide (0.80 g, 1.6 mmol, 2.0 equiv.) was dissolved in 200 mL deionized water and rinsed slowly over 200 g Amberlite IRA 402(Cl), freshly activated by 1 M NaOH solution (Cl^- → OH^-). The basic fractions were collected and tungstic acid (H_2WO_4, 0.24 g, 0.96 mmol, 1.2 equiv.) was added. The mixture was stirred at room temperature until a pH of 7. Excessive tungstic acid was filtered off and the solvent was removed under reduced pressure. After drying in vacuum (10^{-3} mbar) 0.83 g (84 %) 3-(6-(4-((4-butylphenyl)diazenyl)phenoxy)hexyl)-1-methylimidazolium tungstate was obtained as an orange solid.

^{1}H-NMR (400 MHz, DMSO-d_6, 298 K) δ [ppm]: 9.35 (s, 2H), 7.90 – 7.82 (m, 4H), 7.81 – 7.73 (m, 6H), 7.73 – 7.68 (m, 2H), 7.39 (d, *J* = 8.3 Hz, 4H), 7.11 (d, *J* = 9.0 Hz, 4H), 4.19 (m, 4H), 4.08 (t, *J* = 6.4 Hz, 4H), 3.86 (s, 6H), 2.71 – 2.63 (d, 4H), 1.89 – 1.71 (m, 8H), 1.65 – 1.56 (m, 4H), 1.52 – 1.43 (m, 4H), 1.35 – 1.22 (m, 8H), 0.90 (t, *J* = 7.4 Hz, 6H).

Elemental analysis calcd. (%) for $C_{52}H_{70}N_8O_6W$: C 57.46, H 6.49, N 10.31; found: C 54.20, H 6.39, N 9.64.

3-(6-(4-((4-butylphenyl)diazenyl)phenoxy)hexyl)-1-octylimidazolium tungstate (**7b**)

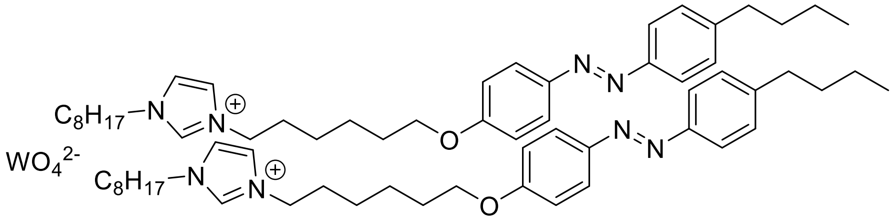

The synthesis was performed according to an anion exchange procedure.[1] 3-(6-(4-((4-butylphenyl)diazenyl)phenoxy)hexyl)-1-octylimidazolium bromide (1.06 g, 1.8 mmol, 2.0 equiv.) was dissolved in 200 mL deionized water and rinsed slowly over 200 g Amberlite IRA 402(Cl), freshly activated by 1 M NaOH solution ($Cl^- \rightarrow OH^-$). The basic fractions were collected and tungstic acid (H_2WO_4, 0.23 g, 0.94 mmol, 1.05 equiv.) was added. The mixture was stirred at room temperature until a pH of 7. Excessive tungstic acid was filtered off and the solvent was removed under reduced pressure. After drying in vacuum (10^{-3} mbar) 0.98 g (85 %) 3-(6-(4-((4-butylphenyl)diazenyl)phenoxy)hexyl)-1-octylimidazolium tungstate was obtained as an orange solid.

^{1}H-NMR (400 MHz, DMSO-d_6, 298 K) δ [ppm]: 9.38 (s, 2H), 7.93 – 7.86 (m, 2H), 7.85 (d, J = 3.0 Hz, 2H), 7.83 – 7.79 (m, 4H), 7.79 – 7.76 (m, 2H), 7.76 – 7.74 (m, 2H), 7.42 – 7.36 (m, 4H), 7.13 – 7.07 (m, 4H), 4.25 – 4.12 (m, 4H), 4.07 (t, J = 6.4 Hz, 4H), 1.88 – 1.70 (m, 16H), 1.63 – 1.57 (m, 4H), 1.52 – 1.42 (m, 4H), 1.38 – 1.30 (m, 4H), 1.25 – 1.21 (m, 28H), 0.92 (t, J = 7.3 Hz, 6H), 0.85 (q, J = 7.2 Hz, 6H).

Elemental analysis calcd. (%) for $C_{66}H_{98}N_8O_6W$: C 61.77, H 7.70, N 8.73; found: C 61.82, H 8.23, N 8.71.

3,3'-(((diazene-1,2-diylbis(4,1-phenylene))bis(oxy))bis(ethane-2,1-diyl))bis(1-methylimidazolium) tungstate (**8b**)

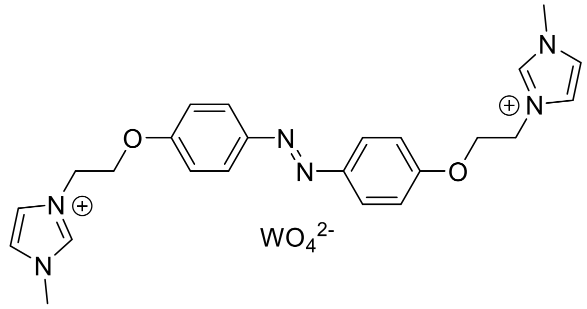

The synthesis was performed according to an anion exchange procedure.[1] 3,3'-(((diazene-1,2-diylbis(4,1-phenylene))bis(oxy))bis(ethane-2,1-diyl))bis(1-methylimidazolium) bromide (0.37 g, 0.57 mmol, 1.0 equiv.) was dissolved in 50 mL deionized water and rinsed slowly over 200 g Amberlite IRA 402(Cl), freshly activated by 1 M NaOH solution ($Cl^- \rightarrow OH^-$). The basic fractions were collected and tungstic acid (H_2WO_4, 0.16 g, 0.63 mmol, 1.1 equiv.) was added. The mixture was stirred at room temperature until a pH of 7. Excessive tungstic acid was filtered off and the solvent was removed under reduced pressure. After drying in vacuum (10^{-3} mbar) 0.35 g (90 %) 3,3'-(((diazene-1,2-diylbis(4,1-phenylene))bis(oxy))bis(ethane-2,1-diyl))bis(1-methylimidazolium)tungstate was obtained as an orange solid.

^{1}H-NMR (400 MHz, D_2O*, 298 K) δ [ppm]: 7.71 (d, *J* = 8.9 Hz, 4H), 7.54 (d, *J* = 1.9 Hz, 2H), 7.40 (d, *J* = 1.8 Hz, 2H), 7.05 (d, *J* = 9.0 Hz, 4H), 4.60 (t, 4H), 4.43 (t, 4H), 3.85 (s, 6H).

^{13}C-NMR (101 MHz, D_2O, 298 K) δ [ppm]: 159.87 (2C), 146.51 (2C), 124.18 (4C), 123.52 (2C), 122.57 (2C), 115.10 (4C), 66.16 (2C), 48.72 (2C), 35.66 (4C).

Elemental analysis calcd. (%) for $C_{24}H_{28}N_6O_6W$: C 42.37, H 4.15, N 12.35; found: C 41.23, H 3.97, N 12.06.

*Solubility in DMSO-d_6 not sufficient for analysis

1-Methyl-3-(4-(phenyldiazenyl)benzyl)imidazolium tungstate (**9b**)

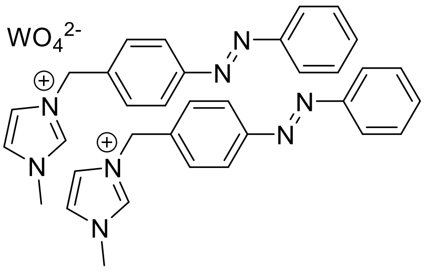

The synthesis was performed according to an anion exchange procedure.[1] 1-Methyl-3-(4-(phenyldiazenyl)benzyl)imidazolium bromide (1.00 g, 2.80 mmol, 2.0 equiv.) was dissolved in 100 mL deionized water and rinsed slowly over 200 g Amberlite IRA 402(Cl), freshly activated by 1 M NaOH solution ($Cl^- \rightarrow OH^-$). The basic fractions were collected and tungstic acid (H_2WO_4, 0.37 g, 1.47 mmol, 1.05 equiv.) was added. The mixture was stirred at room temperature until a pH of 7. Excessive tungstic acid was filtered off and the solvent was removed under reduced pressure. After drying in vacuum (10^{-3} mbar) 1.01 g 1-methyl-3-(4-(phenyldiazenyl)benzyl)imidazolium tungstate (90 %) was obtained as an orange solid.

^{1}H-NMR (400 MHz, DMSO-d_6, 298 K) δ [ppm]: 10.06 (s, 1H), 7.88 (dd, *J* = 7.8, 2.2 Hz, 4H), 7.85 (d, *J* = 2.0 Hz, 1H), 7.76 – 7.72 (m, 2H), 7.71 (d, *J* = 1.8 Hz, 1H), 7.61 – 7.56 (m, 3H), 5.66 (s, 2H), 3.91 (s, 3H).^{13}C-NMR (101 MHz, DMSO-d_6, 298 K) δ [ppm]: 151.88, 151.84, 138.36, 138.01, 131.82, 129.71 (2C), 129.54 (2C),124.01, 122.98 (2C), 122.65 (2C), 122.30, 51.47, 35.96.

Elemental analysis calcd. (%) for $C_{34}H_{34}N_8O_4W$: C 50.89, H 4.27, N 13.96; found: C 51.31, H 4.39, N 13.78.

1,3-Dimethyl-2-(phenyldiazenyl)imidazolium tungstate (**10b**)

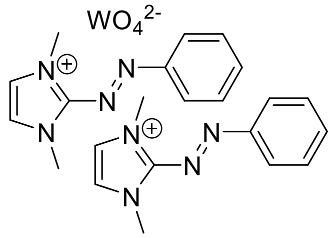

The synthesis was performed according to an anion exchange procedure.[1] 1,3-Dimethyl-2-(phenyldiazenyl)imidazolium iodide (0.7 g, 2.1 mmol, 2.0 equiv.) was dissolved in 150 mL deionized water and rinsed slowly over 200 g Amberlite IRA 402(Cl), freshly activated by 1 M NaOH solution ($Cl^- \rightarrow OH^-$). The basic fractions were collected and neutralized with 2M HNO_3 until pH = 7, using a calibrated pH meter. After solvent removal the product was purified by solvation in MeOH and reprecipitation with Et_2O. The product was dried under vacuum (10^{-3} mbar) to yield 0.59 g (49 %) 1,3-Dimethyl-2-(phenyldiazenyl)imidazolium nitrate as red powder.

^{1}H-NMR (400 MHz, DMSO-d_6, 298 K) δ [ppm]: 8.11 (m, 2 H), 8.03 (s, 2 H), 7.76 (m, 3 H), 4.11 (s, 6 H).

Elemental analysis calcd. (%) for $C_{22}H_{26}N_8O_4W$: C 40.63, H 4.03, N 17.23; found C 39.57, H 4.32, N 17.01.

1.2.9. Azobenzene-functionalized imidazolium nitrates

1,3-Dimethyl-2-(phenyldiazenyl)imidazolium nitrate (**10c**)

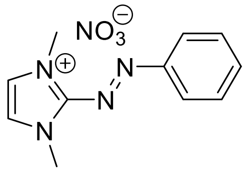

The synthesis was adapted according to a previously published anion exchange procedure.[7] 1,3-Dimethyl-2-(phenyldiazenyl)imidazolium iodide (0.7 g, 2.1 mmol, 1.0 equiv.) was dissolved in 150 mL deionized water and rinsed slowly over 200 g Amberlite IRA 402(Cl), freshly activated by 1 M NaOH solution ($Cl^- \rightarrow OH^-$). The basic fractions were collected and neutralized with 2M HNO_3 until pH = 7, using a calibrated pH meter. After solvent removal the product was purified by solvation in MeOH and reprecipitation with Et_2O. The product was dried under vacuum (10^{-3} mbar) to yield 0.59 g (49 %) 1,3-Dimethyl-2-(phenyldiazenyl)imidazolium nitrate as red powder.

^{1}H-NMR (400 MHz, DMSO-d_6, 298 K) δ [ppm]: 8.11 (m, 2 H), 8.03 (s, 2 H), 7.76 (m, 3 H), 4.11 (s, 6 H).

Elemental analysis calcd. (%) for $C_{11}H_{13}N_5O_3$: C 50.19, H 4.98, N 26.60; found C 48.95, H 5.39, N 26.98.

1.2.10. Azobenzene-functionalized imidazolium benzenesulfonates

3-((4-(2-(1-methylimidazolium)ethoxy)phenyl)diazenyl)benzenesulfonate (**11**)

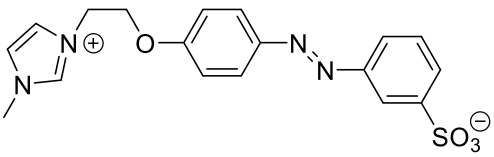

Potassium 4-((4-(2-chloroethoxy)phenyl)diazenyl)benzenesulfonate (0.32 g, 0.85 mmol, 1.0 equiv.) was dissolved in 20 ml dry DMF. 1-Methylimidazole (0.37 mL, 4.70 mmol, 5.5 equiv.) was added and the reaction was stirred at 70 °C overnight under inert conditions. Afterwards, the solvent and excessive imidazole were removed under reduced pressure. The residual was dissolved in small amounts of MeOH, precipitated in Et_2O and washed with Et_2O (3 x 30ml). To remove halide contaminations the solid was dissolved in water:MeCN (1:1) and rinsed slowly over 200 g Amberlite IRA 402(Cl), freshly activated by 1 M NaOH solution ($Cl^- \rightarrow OH^-$) with water:MeCN (1:3) as eluent.* The yellow fractions were collected and the solvent was removed under reduced pressure. The crude was dissolved in small amounts of MeOH, precipitated in Et_2O and washed with Et_2O (3 x 30ml). The product was dried under vacuum (10^{-3} mbar) to yield 0.11 g (33 %) 3-((4-(2-(1-methylimidazolium)ethoxy)phenyl)diazenyl)benzenesulfonate as orange solid.

^{1}H-NMR (400 MHz, DMSO-d_6, 298 K) δ [ppm]: 9.22 (s, 1 H), 7.90 (m, 3 H), 7.78 (dt, J = 50.06 Hz, J = 1.91 Hz, 2 H), 7.43 (m, 2 H), 7.34 (m, 1 H), 7.14 (d, J = 8.93 Hz, 2 H), 4.55 (dt, J = 68.22 Hz, J = 4.77 Hz, 4 H), 3.88 (s, 3 H). ^{13}C-NMR (101 MHz, DMSO-d_6, 298 K) δ [ppm]: 160.00, 148.95, 147.43, 144.71, 137.15, 129.57, 129.33, 128.16, 125.11 (2 C), 123.65, 122.87, 115.29, 114.99 (2 C), 66.20, 48.39, 35.87.

Elemental analysis calcd. (%) for $C_{18}H_{18}N_4O_4S$: C 55.95, H 4.70, N 14.50, S 8.30; found C 56.16, H 4.42, N 14.41, S 8.26.

*Note that the product remains in the stationary phase if only water is used as eluent, due to the strong interaction of the sulfonate group with the ammonium-functionalized anion exchange resin.

2-((4-(2-(1-methylimidazolium)ethoxy)phenyl)diazenyl)benzenesulfonate (**12**)

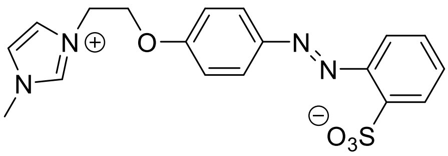

Potassium 2-((4-(2-bromoethoxy)phenyl)diazenyl)benzenesulfonate (0.80 g, 1.89 mmol, 1.0 equiv.) was dissolved in 20 ml dry DMF. 1-Methylimidazole (0.83 mL, 10.38 mmol, 5.0 equiv.) was added and the reaction was stirred at 70 °C overnight under inert conditions. Afterwards, the solvent and excessive imidazole were removed under reduced pressure. The residual was dissolved in small amounts of MeOH, precipitated in Et_2O and washed with Et_2O (3 x 30ml). To remove halide contaminations the solid was dissolved in water:MeCN (1:1) and rinsed slowly over 200 g Amberlite IRA 402(Cl), freshly activated by 1 M NaOH solution ($Cl^- \rightarrow OH^-$) with water:MeCN (1:3) as eluent.* The yellow fractions were collected and the solvent was removed under reduced pressure. The crude was dissolved in small amounts of MeOH, precipitated in Et_2O and washed with Et_2O (3 x 30ml). The product was dried under vacuum (10^{-3} mbar) to yield 0.27 g (37 %) 2-((4-(2-(1-methylimidazolium)ethoxy)phenyl)diazenyl)benzenesulfonate as red solid.

^{1}H-NMR (400 MHz, DMSO-d_6, 298 K) δ [ppm]: 9.22 (s, 1 H), 8.03 (t, J = 1.83 Hz, 1 H), 7.93 (m, 2 H), 7.77 (m, 4 H), 7.55 (t, J = 7.76 Hz, 1 H), 7.18 (m, 2 H), 4.58 (dt, J = 68.42 Hz, J = 4.47 Hz, 4 H), 3.89 (s, 3 H). ^{13}C-NMR (101 MHz, DMSO-d_6, 298 K) δ [ppm]: 160.43, 151.38, 149.64, 146.58, 137.13, 128.64, 127.94, 124.64 (2 C), 124.40, 123.60, 122.86, 117.34, 115.35 (2 C), 66.20, 48.35, 35.85.

Elemental analysis calcd. (%) for $C_{18}H_{18}N_4O_4S$: C 55.95, H 4.70, N 14.50, S 8.30; found C 55.71, H 4.66, N 14.41, S 8.23.

*Note that the product remains in the stationary phase if only water is used as eluent, due to the strong interaction of the sulfonate group with the ammonium-functionalized anion exchange resin.

4-((4-(2-(1-methylimidazolium)ethoxy)phenyl)diazenyl)benzenesulfonate (**13**)

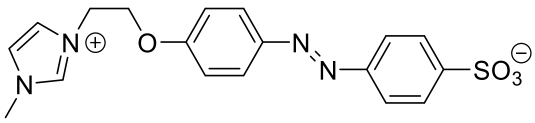

Potassium 4-((4-(2-chloroethoxy)phenyl)diazenyl)benzenesulfonate (1.55 g, 3.99 mmol, 1.0 equiv.) was dissolved in 20 ml dry DMF. 1-Methylimidazole (3.63 mL, 45.48 mmol, 11.5 equiv.) was added and the reaction was stirred at 70 °C overnight under inert conditions. Afterwards, the solvent and excessive imidazole were removed under reduced pressure. The residual was dissolved in small amounts of MeOH, precipitated in Et_2O and washed with Et_2O (3 x 30ml). To remove halide contaminations the solid was dissolved in water:MeCN (1:1) and rinsed slowly over 200 g Amberlite IRA 402(Cl), freshly activated by 1 M NaOH solution ($Cl^- \rightarrow OH^-$) with water:MeCN (1:3) as eluent.* The yellow fractions were collected and the solvent was removed under reduced pressure. The crude was dissolved in small amounts of MeOH, precipitated in Et_2O and washed with Et_2O (3 x 30ml). The product was dried under vacuum (10^{-3} mbar) to yield 0.37 g (24 %) 4-((4-(2-(1-methylimidazolium)ethoxy)phenyl)diazenyl)benzenesulfonate as orange solid.

^{1}H-NMR (400 MHz, DMSO-d_6, 298 K) δ [ppm]: 9.20 (s, 1 H), 8.03 (m, 1 H), 7.94 (m, 2 H), 7.83 (m, 2 H), 7.74 (m, 2 H), 7.55 (t, J = 7.70 Hz, 1 H), 7.18 (m, 2 H), 4.56 (dt, J = 65.50 Hz, J = 4.80 Hz, 4 H), 3.88 (s, 3 H). ^{13}C-NMR (101 MHz, DMSO-d_6, 298 K) δ [ppm]: 160.43, 149.66, 146.61, 137.12, 128.99 (2 C), 124.66 (2 C), 124.44, 123.62, 122.88, 117.32 (2 C), 115.37 (2 C), 66.20, 48.38, 35.87.

Elemental analysis calcd. (%) for $C_{18}H_{18}N_4O_4S$: C 55.95, H 4.70, N 14.50, S 8.30; found C 55.67, H 4.44, N 14.64, S 8.06.

*Note that the product remains in the stationary phase if only water is used as eluent, due to the strong interaction of the sulfonate group with the ammonium-functionalized anion exchange resin.

2. Analysis of AzoSAILs

2.1. Isomerization studies

Note that the measurements of the UV-Vis spectra and the ^{1}H-NMR spectra for samples with 10 mmol/L concentration were performed using the same sample if not stated otherwise. For the UV-Vis measurements an aliquot (3 µl) of the sample solution was diluted with D_2O to a volume of 3 ml if not stated otherwise. The measurement of **1b** was performed in DMSO-d_6 to demonstrate that the isomerization is not affected by different solvents. The measurements of perrhenates were performed with saturated solutions as the solubility is notably lower than 10 mmol/L.

2.1.1. UV-Vis measurements

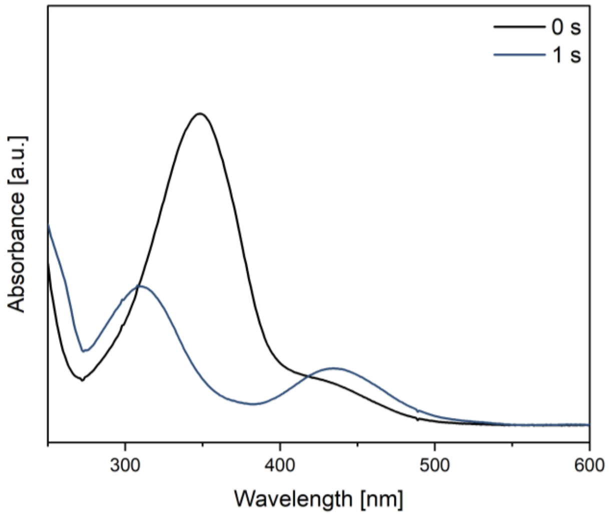

Figure S1. UV-Vis spectra of the reverse isomerization of the *trans*-form of **3a** to the *cis*-form by irradiation with 365 nm at room temperature and a sample concentration of $1 \cdot 10^{-4}$ mmol/L in D_2O. The absorption maxima are 433 as well as 321 nm for the *cis*-form.

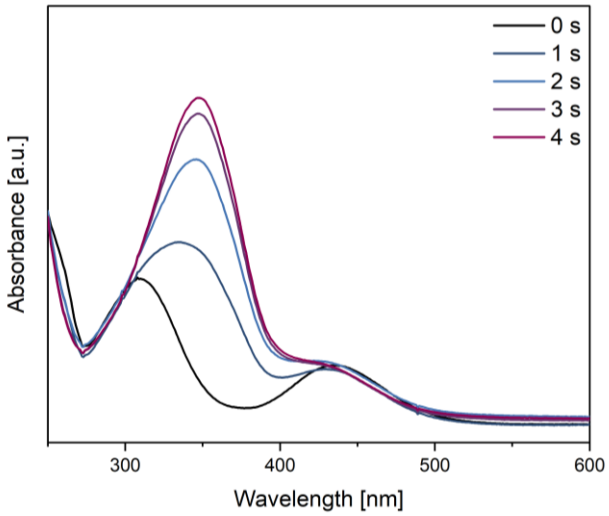

Figure S2. UV-Vis spectra of the reverse isomerization of the *cis*-form of **3a** to the *trans*-form by irradiation with 460 nm at room temperature and a sample concentration of $1 \cdot 10^{-4}$ mmol/L in D_2O. The absorption maxima is at 348 nm for the *trans*-form.

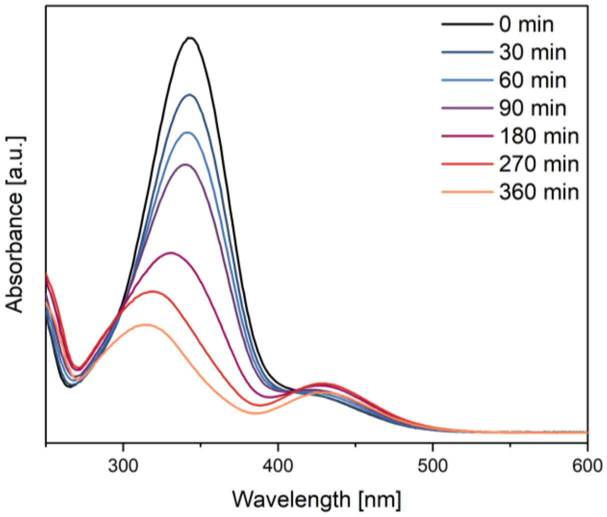

Figure S3. UV-Vis spectra of the isomerization of the *trans*-form of **1b** to the *cis*-form by irradiation with 365 nm at room temperature and a sample concentration of 178 mmol/L in DMSO-d_6. The absorption maxima are 431 as well as 313 nm for the *cis*-form.

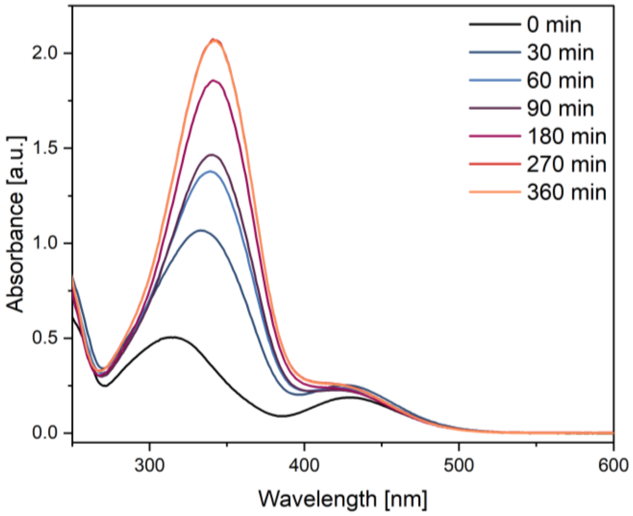

Figure S4. UV-Vis spectra of the reverse isomerization of the *cis*-form of **1b** to the *trans*-form by irradiation with 460 nm at room temperature and a sample concentration of 178 mmol/L in DMSO-d_6. The absorption maxima is at 343 nm for the *trans*-form.

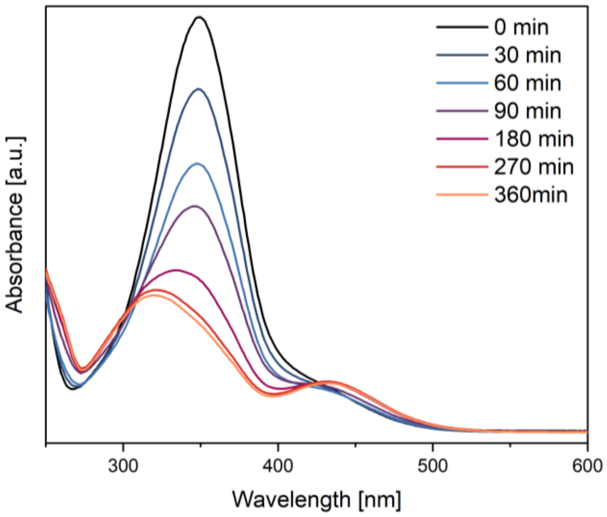

Figure S5. UV-Vis spectra of the isomerization of the *trans*-form of **3b** to the *cis*-form by irradiation with 365 nm at room temperature and a sample concentration of 178 mmol/L in D_2O. The absorption maxima are 431 as well as 310 nm for the *cis*-form.

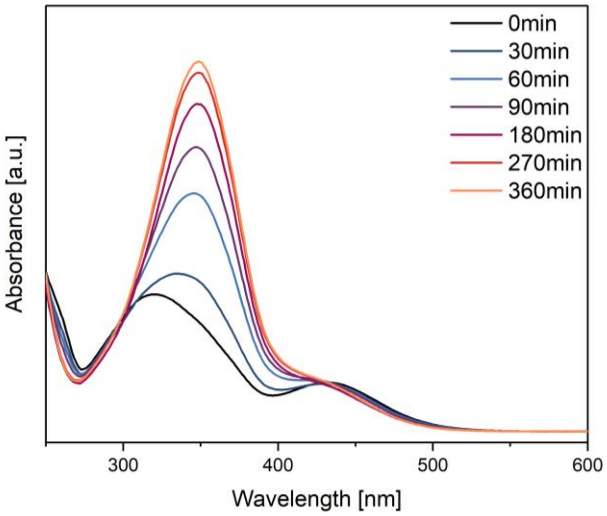

Figure S6. UV-Vis spectra of the reverse isomerization of the *cis*-form of **3b** to the *trans*-form by irradiation with 460 nm at room temperature and a sample concentration of 178 mmol/L in D_2O. The absorption maxima is at 347 nm for the *trans*-form.

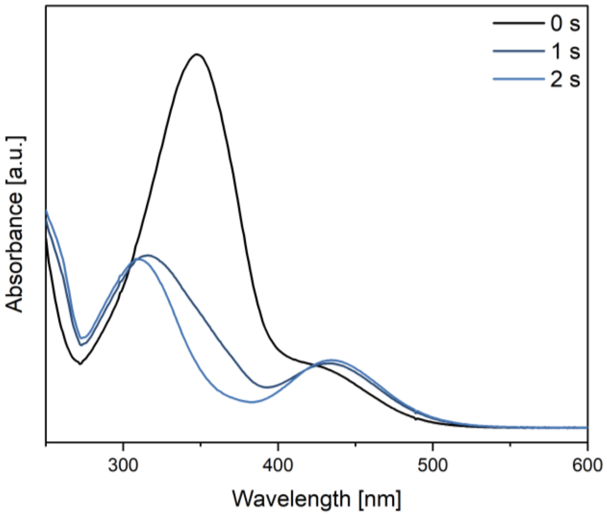

Figure S7. UV-Vis spectra of the reverse isomerization of the *trans*-form of **3b** to the *cis*-form by irradiation with 365 nm at room temperature and a sample concentration of $1 \cdot 10^{-4}$ mmol/L in D_2O.

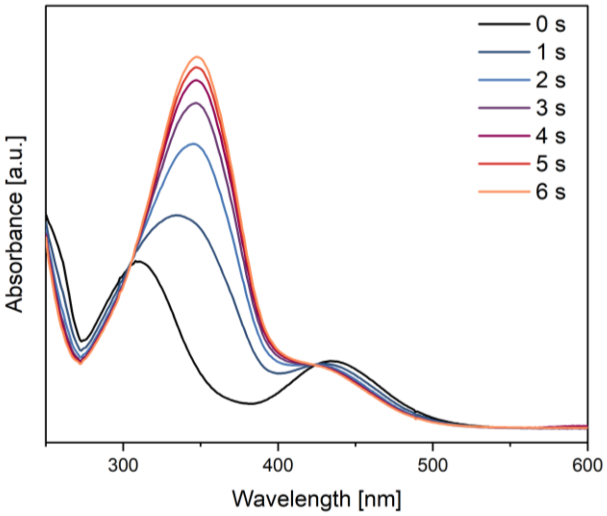

Figure S8. UV-Vis spectra of the reverse isomerization of the *cis*-form of **3b** to the *trans*-form by irradiation with 460 nm at room temperature and a sample concentration of $1 \cdot 10^{-4}$ mmol/L in D_2O.

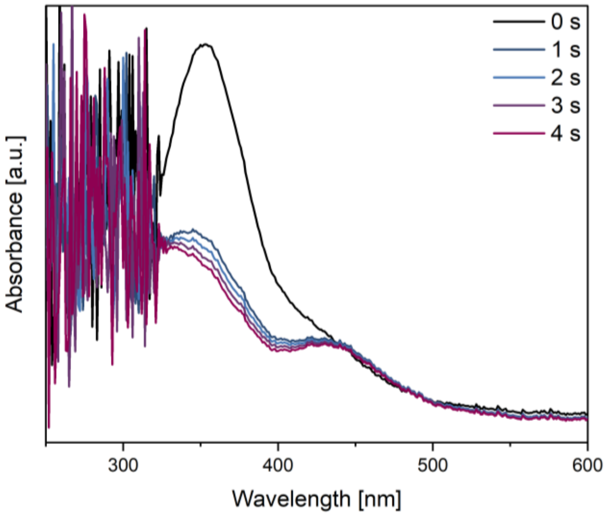

Figure S9. UV-Vis spectra of the reverse isomerization of the *trans*-form of **3b** to the *cis*-form by irradiation with 365 nm at room temperature and a sample concentration of $1{\cdot}10^{-4}$ mmol/L in 50 wt.% aq. H_2O_2. The absorption maxima are 430 as well as 333 nm for the *cis*-form.

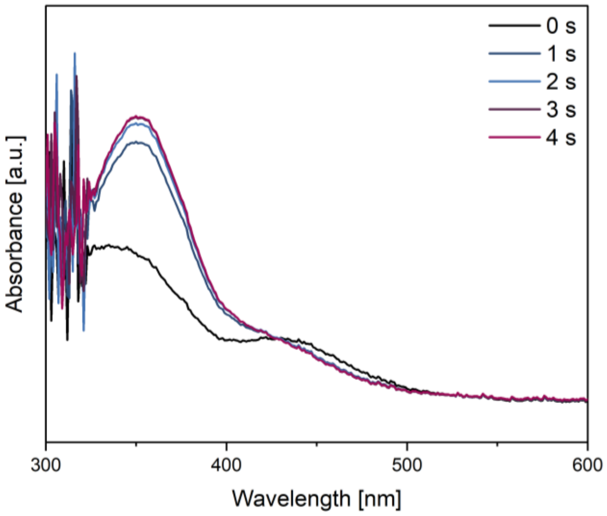

Figure S10. UV-Vis spectra of the reverse isomerization of the *cis*-form of **3b** to the *trans*-form by irradiation with 460 nm at room temperature and a sample concentration of $1{\cdot}10^{-4}$ mmol/L in D_2O. The absorption maxima are 350 nm for the *trans*-form.

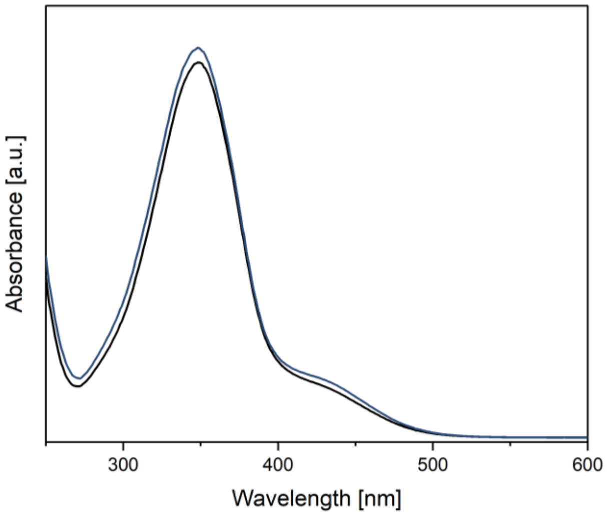

Figure S11. UV-Vis spectra of **3b** with a sample concentration of $1{\cdot}10^{-4}$ mmol/L in D_2O (black) and after irradiation with 365 nm for 90 min at 50 °C (blue).

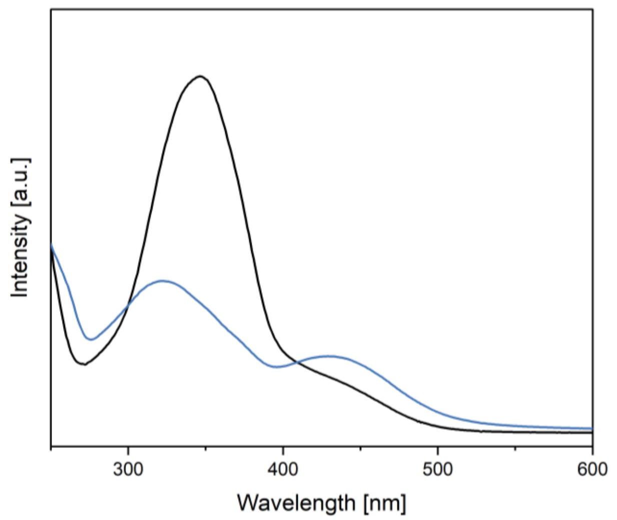

Figure S12. UV-Vis spectra of the *trans*-form (black) and *cis*-form (blue) of 10 mmol/L **4b** in D_2O with the absorption maxima at 347 nm for the *trans*- and 428 as well as 329 nm for the *cis*-form.

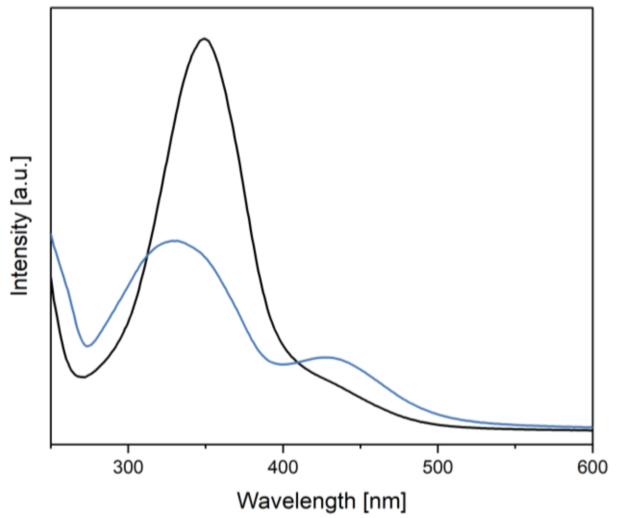

Figure S13. UV-Vis spectra of the *trans*-form (black) and *cis*-form (blue) of 10 mmol/L **5b** in D_2O with the absorption maxima at 349 nm for the *trans*- and 428 as well as 327 nm for the *cis*-form.

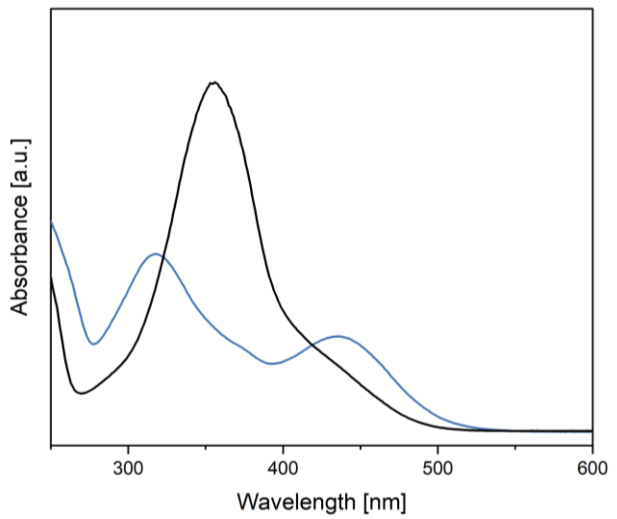

Figure S14. UV-Vis spectra of the *trans*-form (black) and *cis*-form (blue) of 10 mmol/L **8b** in D_2O with the absorption maxima at 353 nm for the *trans*- and 436 as well as 318 nm for the *cis*-form.

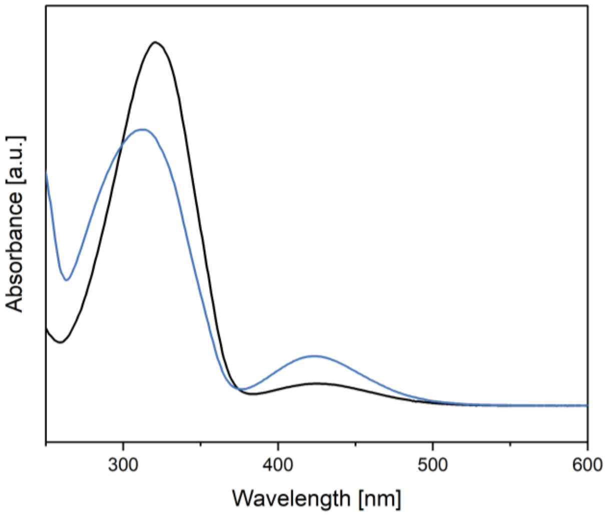

Figure S15. UV-Vis spectra of the *trans*-form (black) and *cis*-form (blue) of 10 mmol/L **9b** in D_2O with the absorption maxima at 321 nm for the *trans*- and 424 as well as 312 nm for the *cis*-enriched* form.
*Note: The terminology cis-enriched form is used, since this compound does not quantitatively form the cis-form as determined via ^{1}H-NMR (photostationary state at 56% cis).

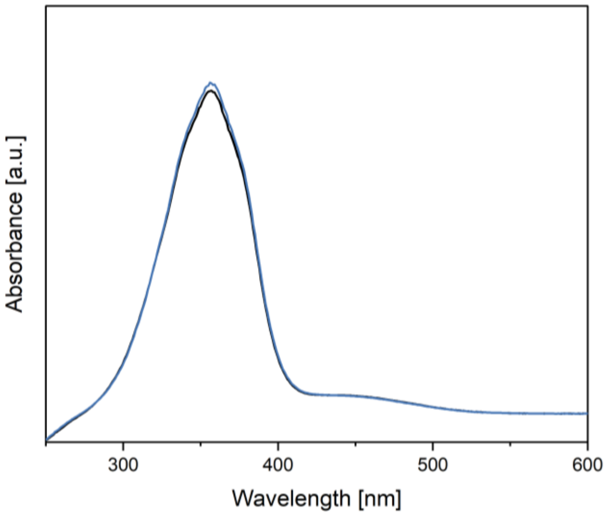

Figure S16. UV-Vis spectra of the *trans*-form (black) 10 mmol/L **10b** in D_2O with the absorption maxima at 353 nm for the *trans*-form. No isomerization is detected after Irradiation with 365 nm (blue).

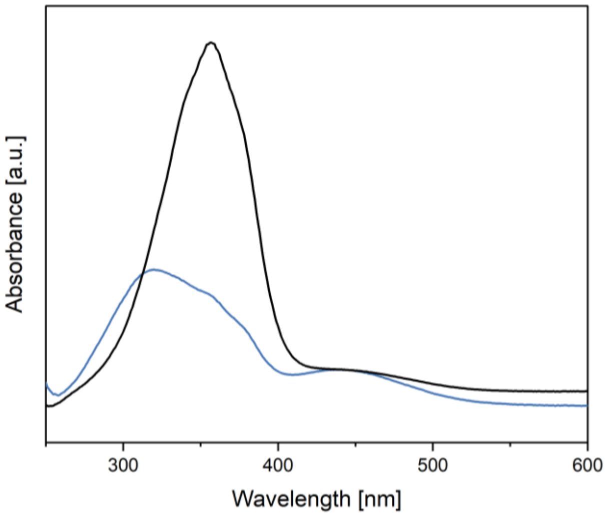

Figure S17. UV-Vis spectra of the *trans*-form (black) and *cis*-form (blue) of 10 mmol/L **10c** in D_2O with the absorption maxima at 357 nm for the *trans*- and 440 as well as 320 nm for the *cis*-form.

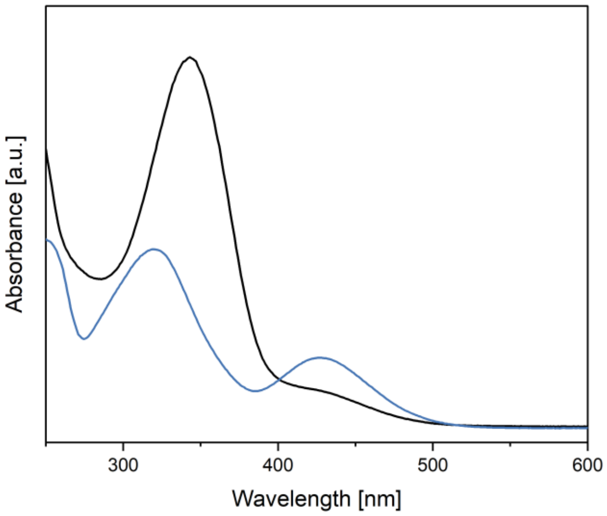

Figure S18. UV-Vis spectra of the *trans*-form (black) and *cis*-form (blue) of 10 mmol/L **11** in D_2O with the absorption maxima at 343 nm for the *trans*- and 427 as well as 320 nm for the *cis*-form.

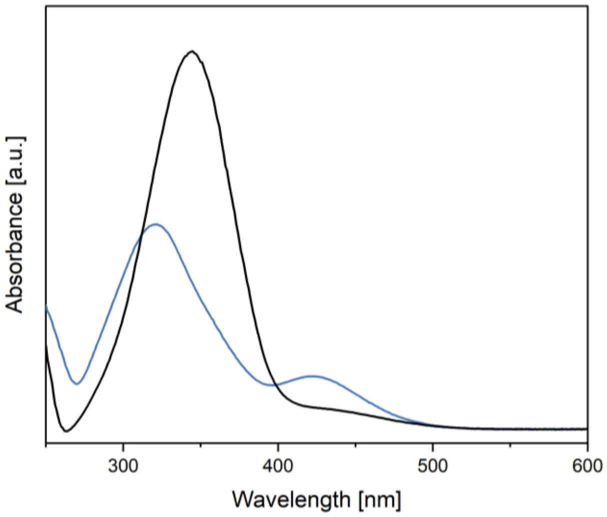

Figure S19. UV-Vis spectra of the *trans*-form (black) and *cis*-form (blue) of 10 mmol/L **12** in D_2O with the absorption maxima at 343 nm for the *trans*- and 427 as well as 319 nm for the *cis*-form.

2.1.2. ^{1}H-NMR measurements

In the following measurements of the two photostationary states are shown for an example of each structure motif and each anion. The measurement of **1b** was performed in DMSO-d_6 to demonstrate that the isomerization is not affected by different solvents. All following measurements were performed in D_2O. The measurements of perrhenates were performed with saturated solutions as the solubility is notably lower than 10 mmol/L.

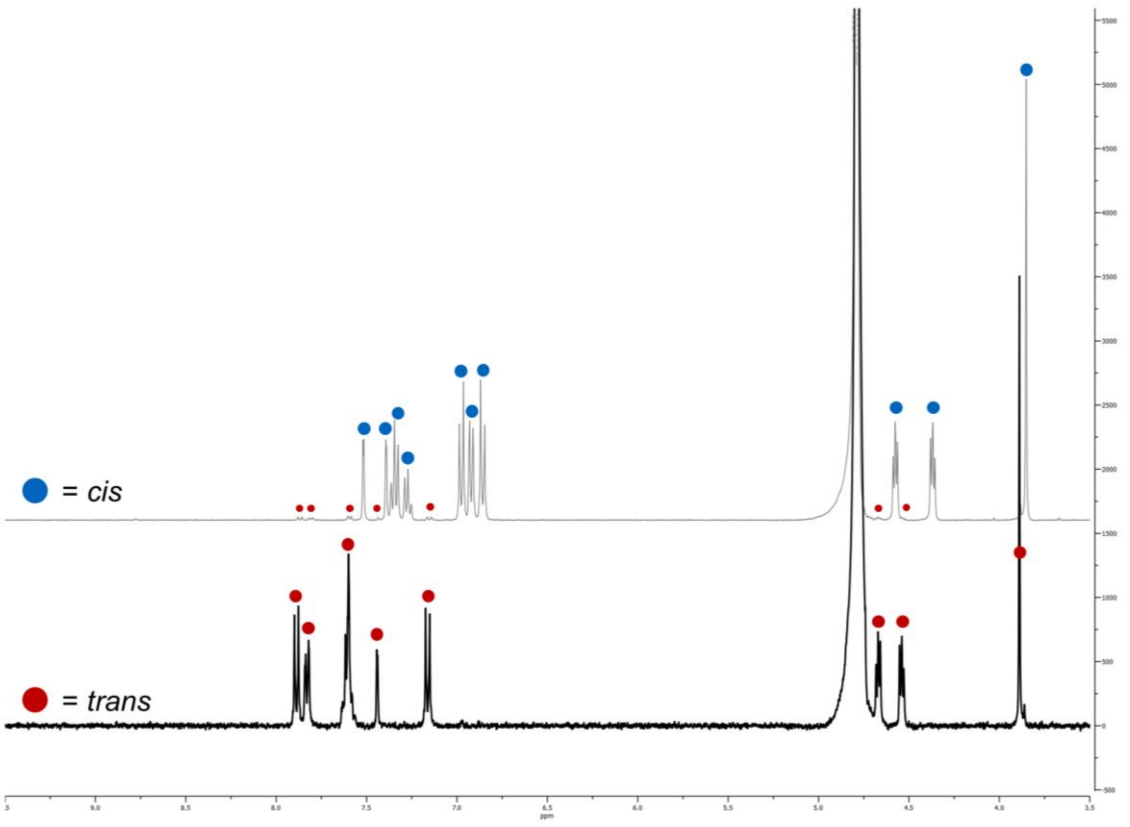

Figure S20. ^{1}H-NMR spectra of saturated solution of **1a** in D_2O after irradiation with 365 nm for 15 h (top) and reverse isomerization by heating of the solution at 50 °C for 24 h (bottom). The respective signals of the *trans*- and *cis*-form are marked with red and blue circles, respectively.

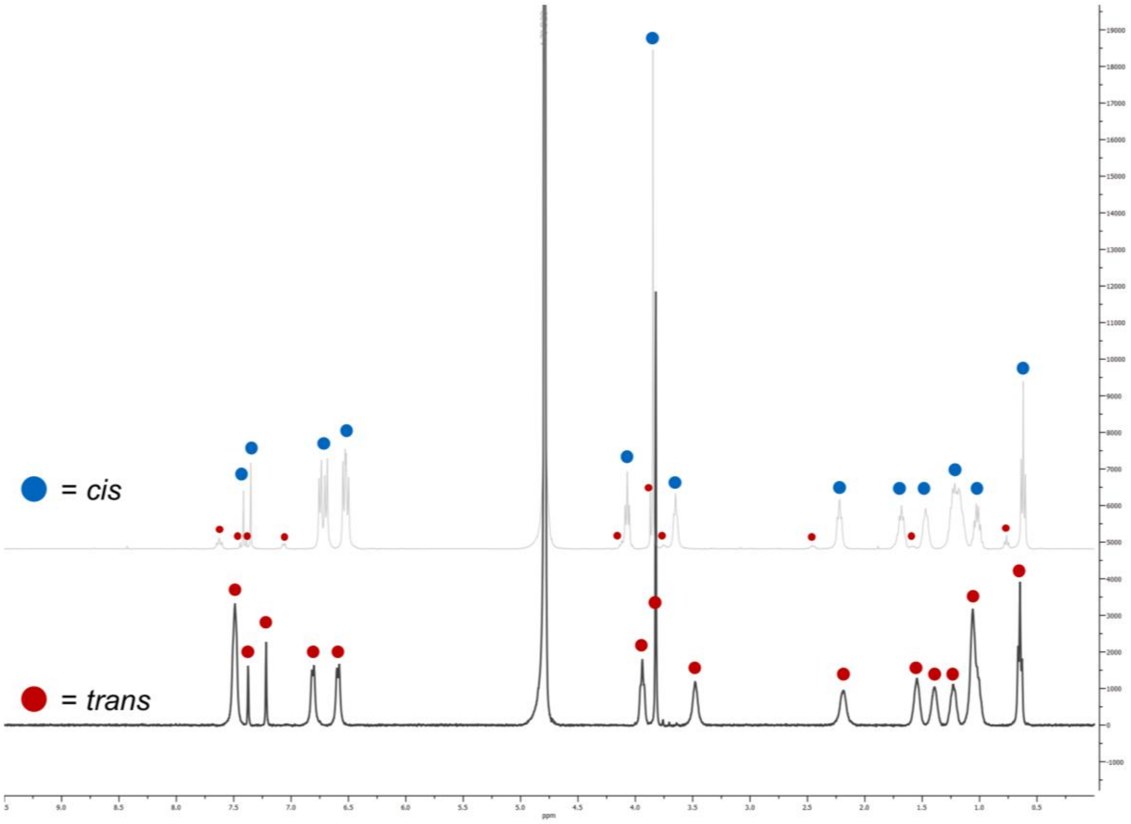

Figure S21. ^{1}H-NMR spectra of saturated solution of **6b** in D_2O after irradiation with 365 nm for 15 h (top) and reverse isomerization by heating of the solution at 50 °C for 24 h (bottom). The respective signals of the *trans*- and *cis*-form are marked with red and blue circles, respectively.

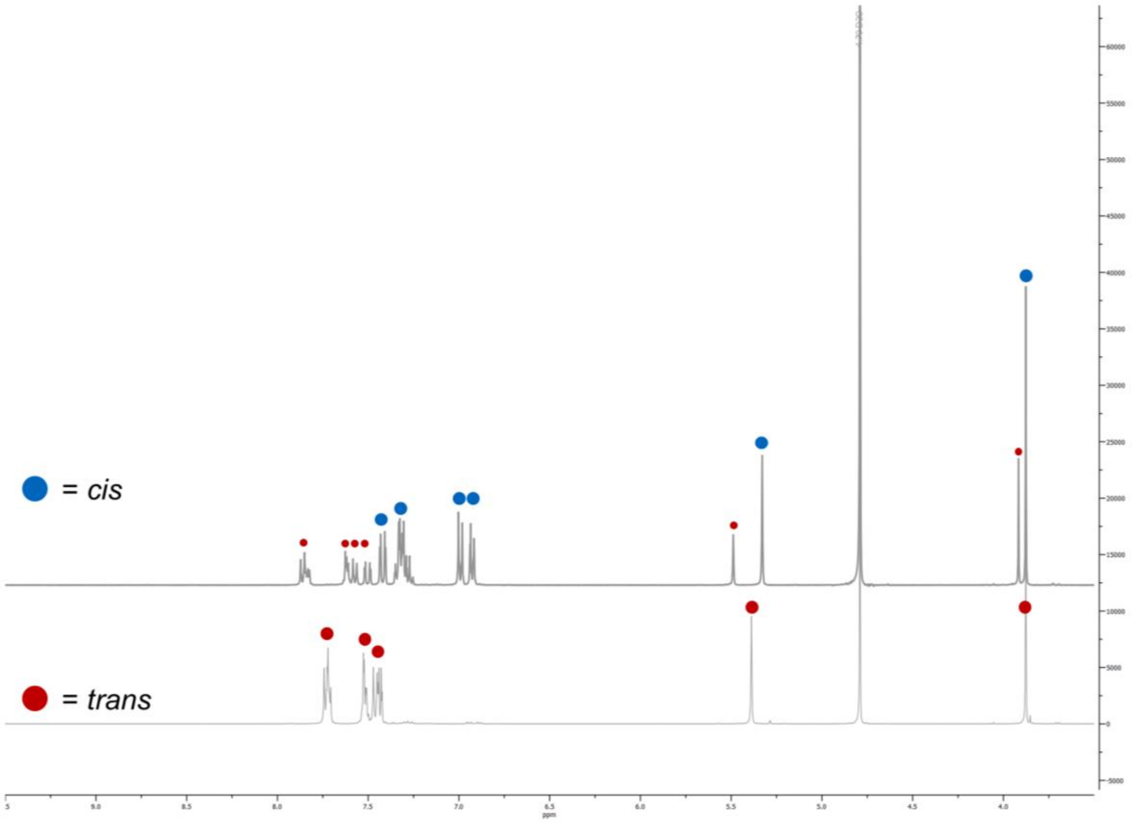

Figure S22. ^{1}H-NMR spectra of saturated solution of **9b** in D_2O after irradiation with 365 nm for 15 h (top) and reverse isomerization by heating of the solution at 50 °C for 24 h (bottom). The respective signals of the *trans*- and *cis*-form are marked with red and blue circles, respectively.

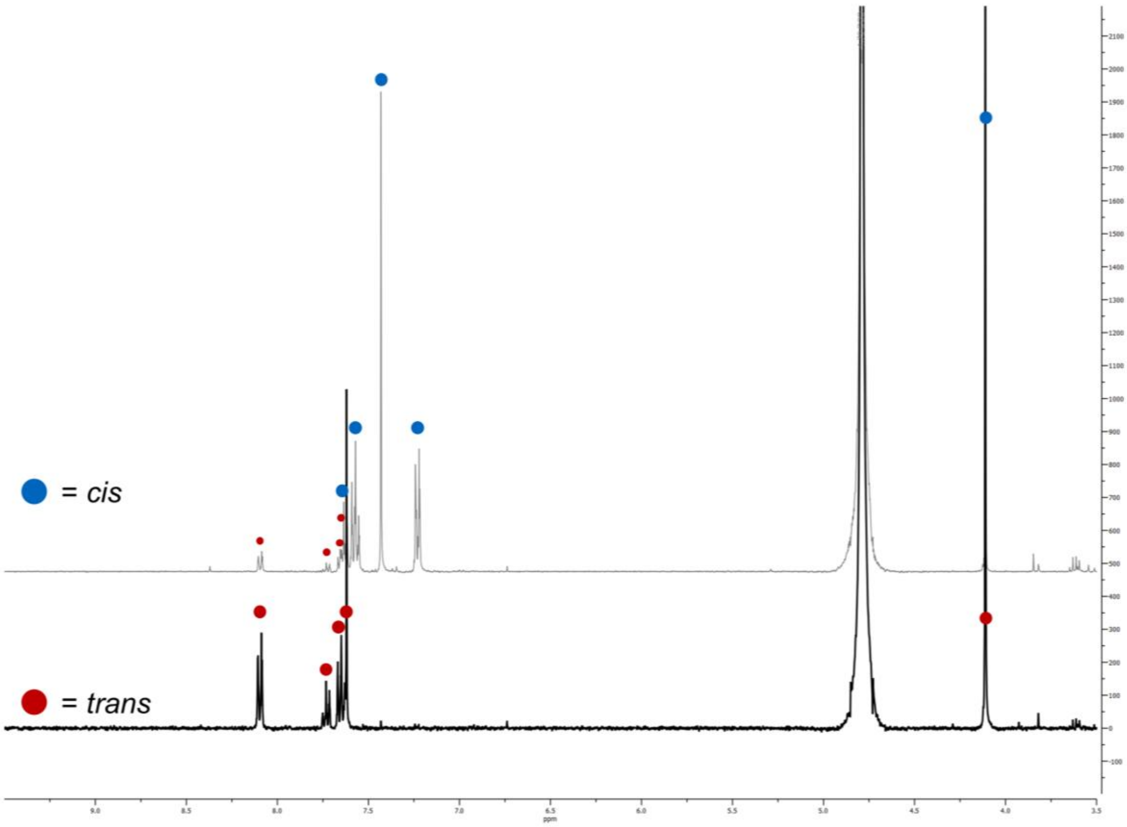

Figure S23. ^{1}H-NMR spectra of saturated solution of **10c** in D_2O after irradiation with 365 nm for 15 h (top) and reverse isomerization by heating of the solution at 50 °C for 24 h (bottom)). The respective signals of the *trans*- and *cis*-form are marked with red and blue circles, respectively.

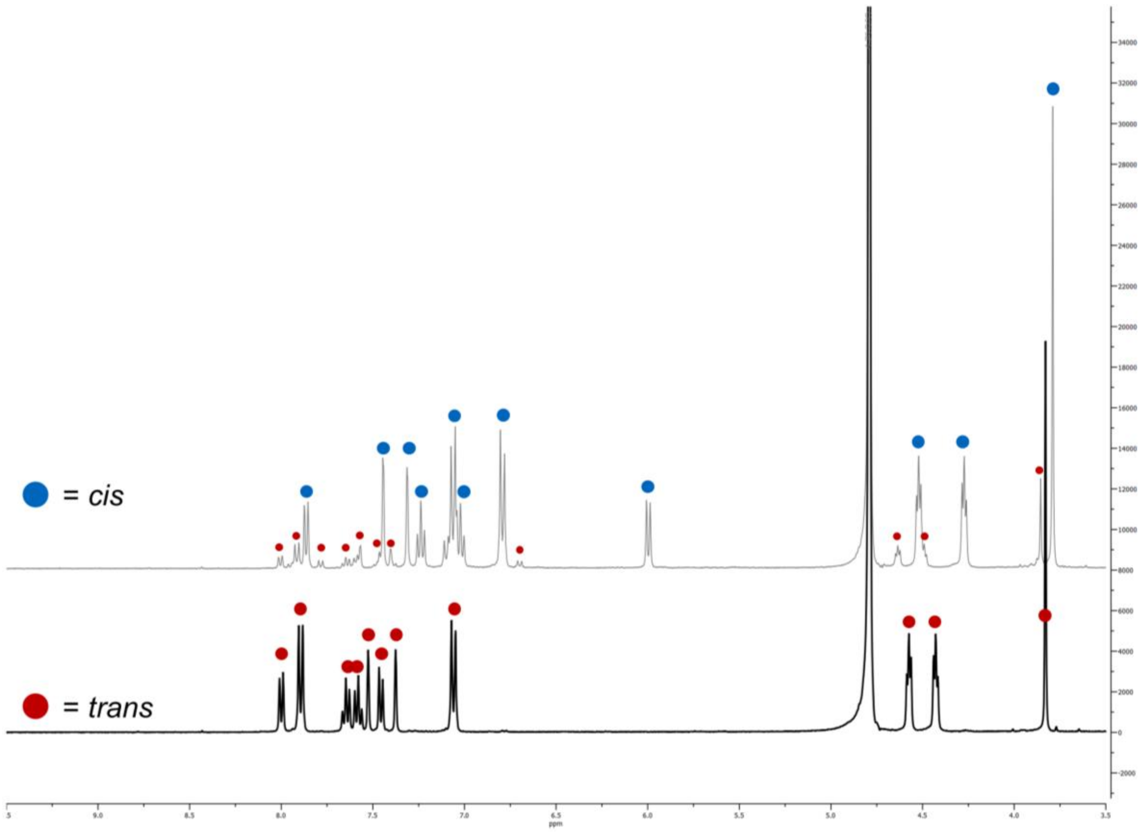

Figure S24. ^{1}H-NMR spectra of saturated solution of **12** in D_2O after irradiation with 365 nm for 15 h (top) and reverse isomerization by heating of the solution at 50 °C for 24 h (bottom).The respective signals of the *trans*- and *cis*-form are marked with red and blue circles, respectively.

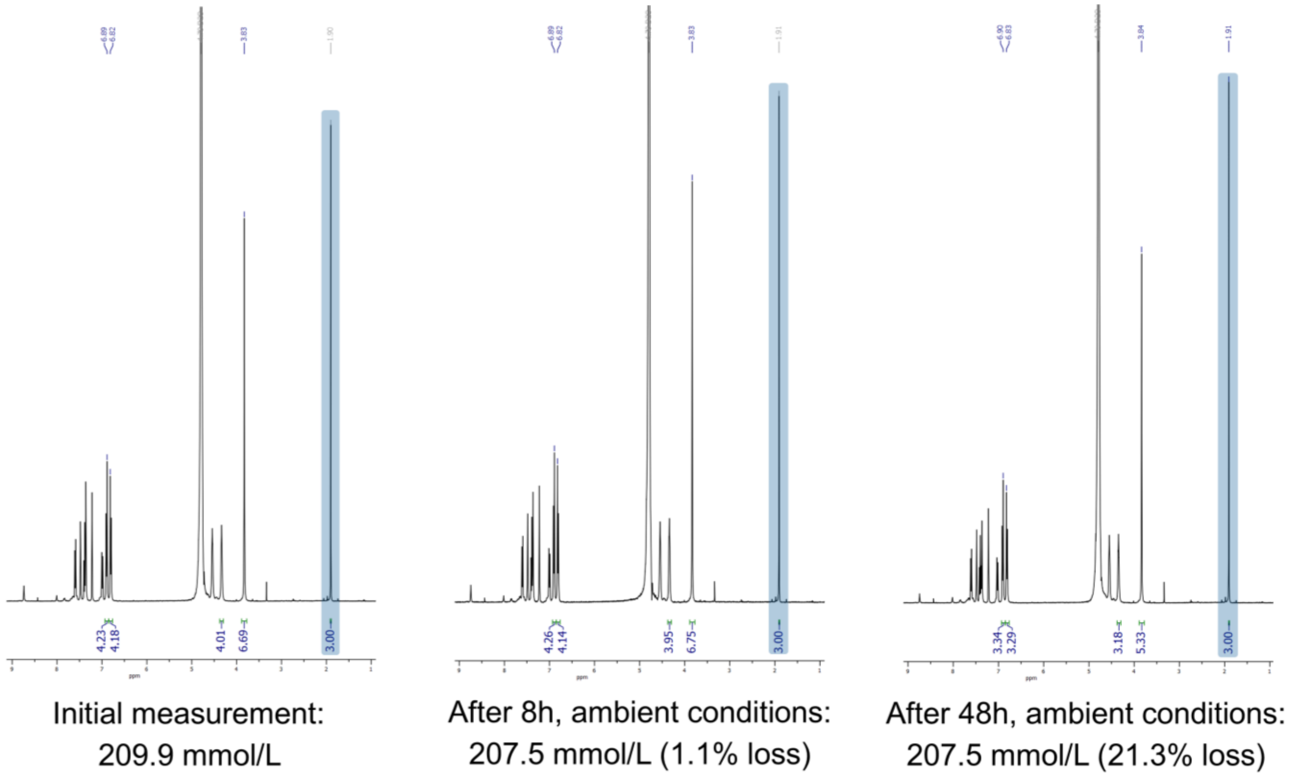

Figure S25. ^{1}H-NMR spectra of **11** in D_2O after irradiation with 365 nm for 15 h (left), 8 h after irradiation at room temperature (middle) and 48 h at room temperature (right). NaOAc (signal marked in blue) is used as reference to determine the loss of **11** due to back isomerization towards the *trans*-form and subsequent precipitation.

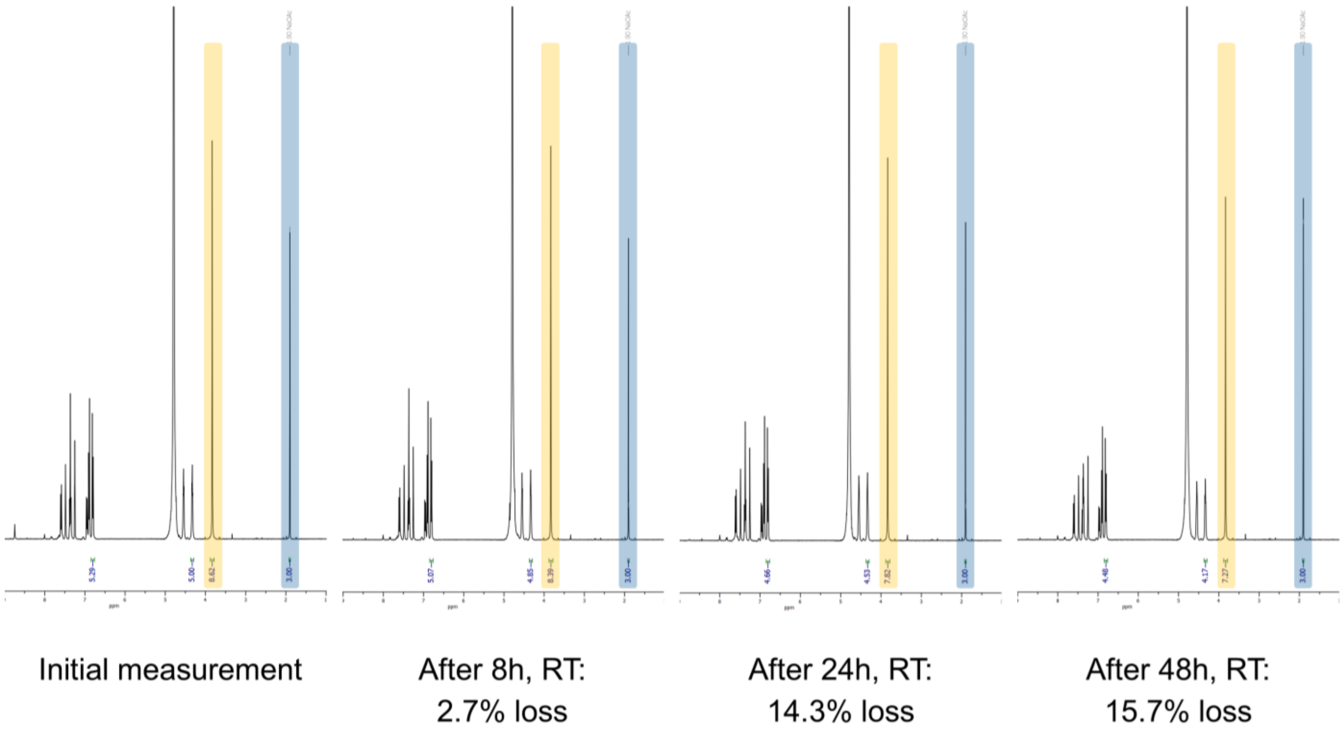

Figure S26. ^{1}H-NMR spectra of ~200 mmol/L **11** and 2 eq. Na_2WO_4 in D_2O after irradiation with 365 nm for 15 h (left), 8 h after irradiation (middle left), 24 h (middle right) and 48 h at room temperature (right). NaOAc (signal marked in blue) is used as reference to determine the loss of **11** (regarding the intensity of the CH_3 signal marked in yellow) due to back isomerization towards the *trans*-form and subsequent precipitation.

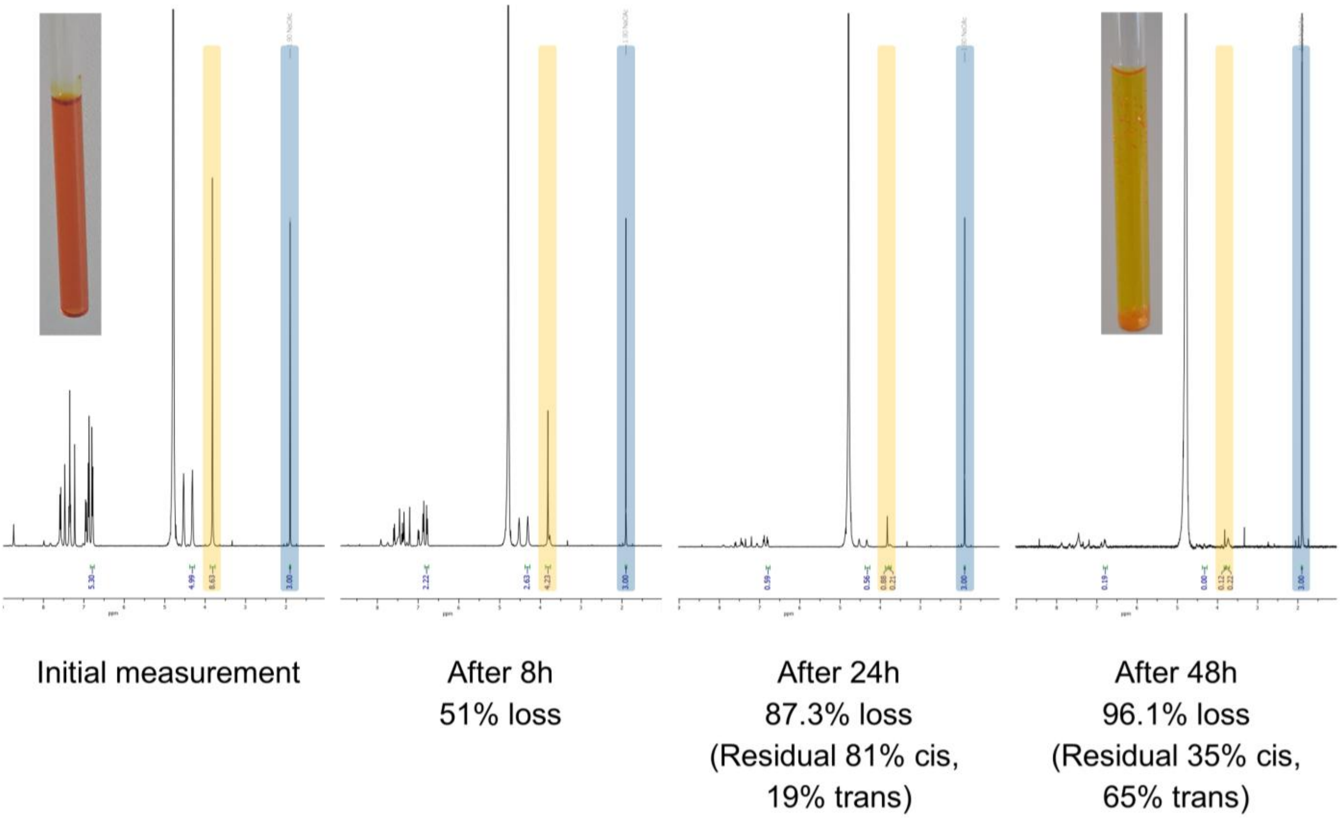

Figure S27. ^{1}H-NMR spectra of ~200 mmol/L **11** and 2 eq. Na_2WO_4 in D_2O after irradiation with 365 nm for 15 h (left, left photograph), 8 h after irradiation (middle left), 24 h (middle right) and 48 h at room temperature (right, right photograph). NaOAc (signal marked in blue) is used as reference to determine the loss of **11** (regarding the intensity of the CH_3 signal marked in yellow) due to back isomerization towards the *trans*-form and subsequent precipitation.

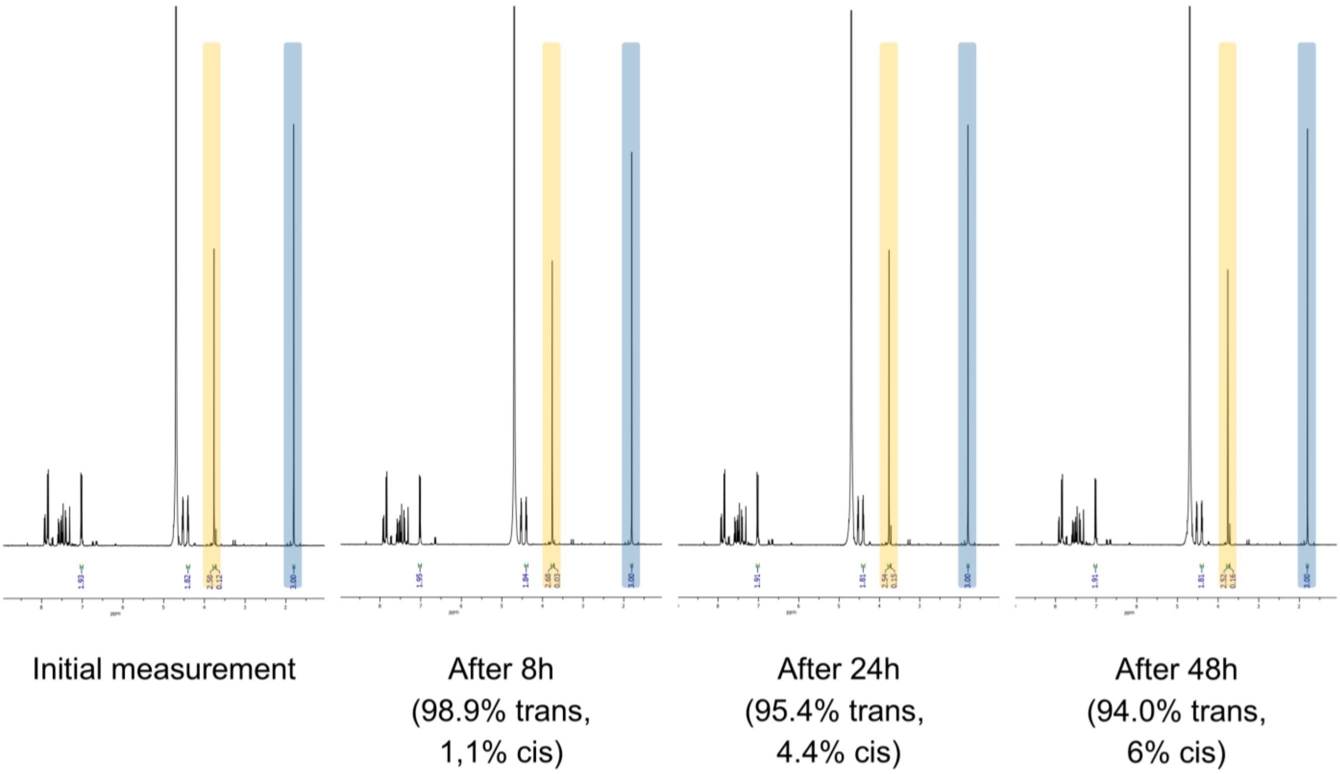

Figure S28. ^{1}H-NMR spectra of a saturated solution of **12** in its *trans*-form in D_2O after dissolving (left), after 8 h (middle left), 24 h (middle right) and 48 h at room temperature (right). NaOAc (signal marked in blue) is used as reference to determine the isomerization towards the *cis*-form under ambient conditions.

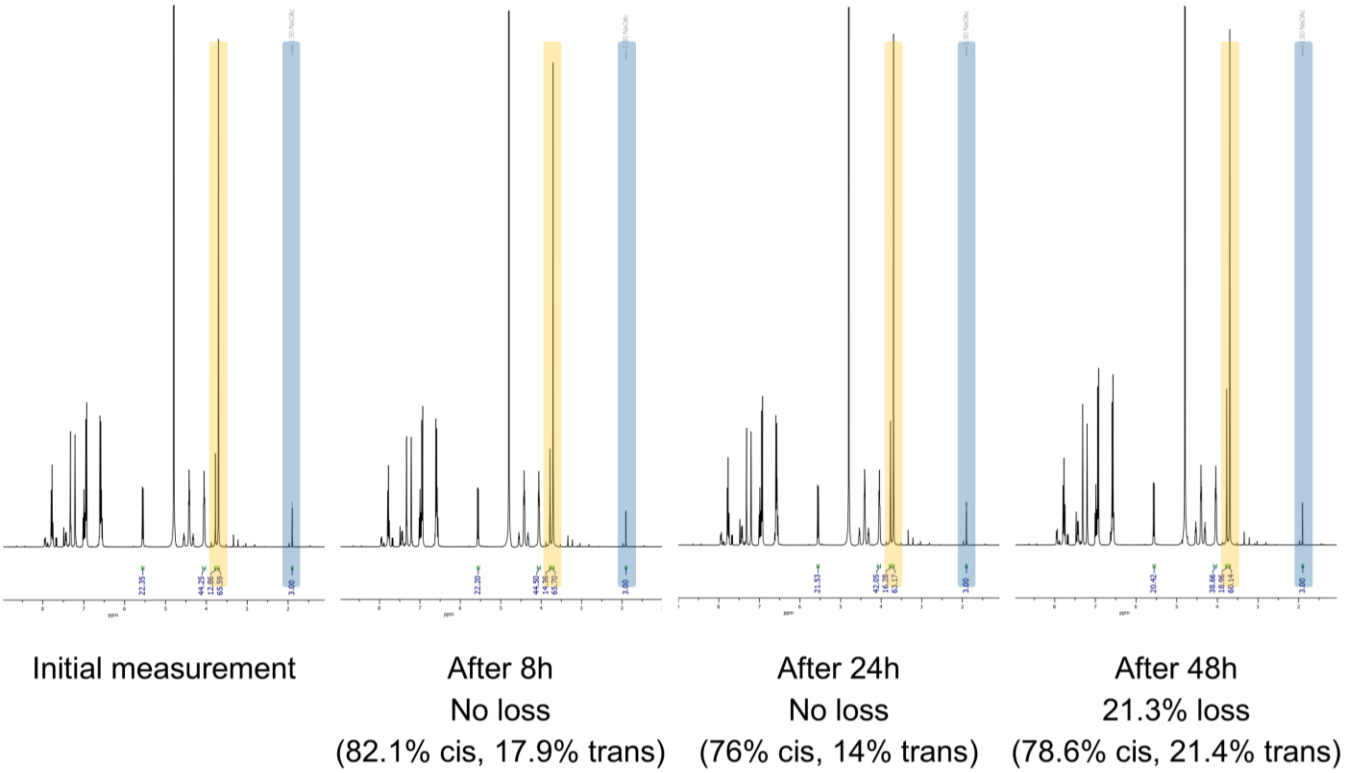

Figure S29. ^{1}H-NMR spectra of a saturated solution of **12** in its *cis*-form in D_2O after dissolving (left), after 8 h (middle left), 24 h (middle right) and 48 h at room temperature (right). NaOAc (signal marked in blue) is used as reference to determine the loss of **12** (regarding the intensity of the CH_3 signal marked in yellow) due to back isomerization towards the *trans*-form and subsequent precipitation.

Table S1. Summary of the maximum obtained *cis*-photostationary state of AzoSAILs by irradiation of a 10 mmol/L solution in D_2O with 365 nm for 15 h at room temperature. The respective amounts of the isomers was determined via ^{1}H-NMR.

Compound	Surfactant	Anion	PSS cis-form [%]
**1a**	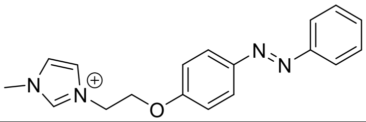	ReO_4^-	97
**1b**	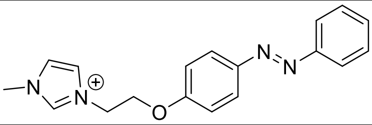	WO_4^{2-}	95
**3a**	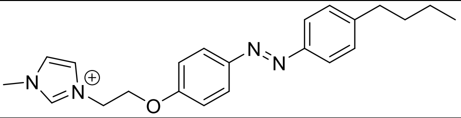	ReO_4^-	99
**3b**	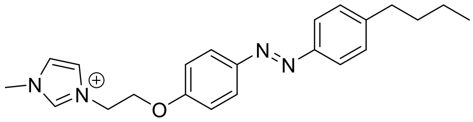	WO_4^{2-}	94
**5a**	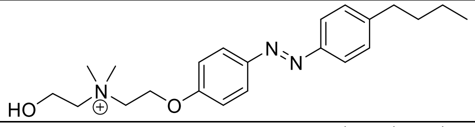	ReO_4^-	91
**5b**	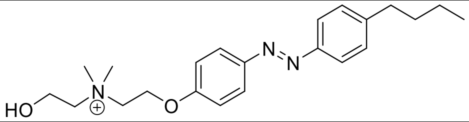	WO_4^{2-}	90
**6b**	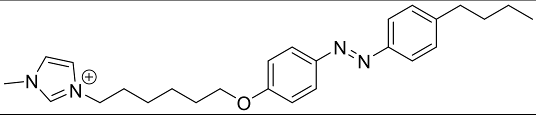	WO_4^{2-}	96
**8b**	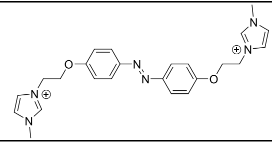	WO_4^{2-}	97
**9b**	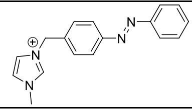	WO_4^{2-}	55
**10b**	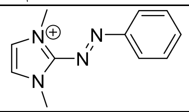	WO_4^{2-}	<1
**10c**	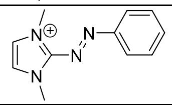	NO_3^-	86
**11**	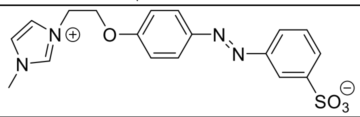	-	96
**12**	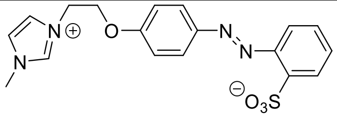	-	86
**13**	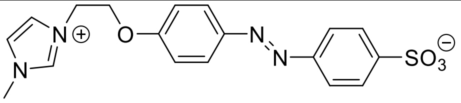	-	80

2.2. Solubility measurements

The solubility of each compound was determined by quantitative ^{1}H-NMR experiments. For each measurement the D_2O was saturated with an excessive amount of IL. The suspension was stirred for 15 h at room temperature to determine to solubility of the trans isomer. For the cis isomer the suspension was stirred and irradiated with 365 nm for 15 h. Then, each sample was filtered by a syringe filter (LLG-Syringe filters SPHEROS, PTFE, 0.22 µm, 13 mm diameter by Lab Logistics Group GmbH). For the solubility determination 100 µL of the aqueous aliquot was given to 400 µL D_2O containing 10 µmol sodium acetate. The integrals of the CH_3 protons of the AzoSAIL (in the range of 3.6–3.8 ppm) are referenced to the signal of the three acetate protons (set to 15) of the standard to obtain the amount of AzoSAIL (in µmol) in the 100 µL aliquot. The solubility of each AzoSAIL in mmol/L in obtained by multiplication of the previously obtained value by 10.

Table S2. Solubility of the photoisomers of the zwitterionic AzoSAILs **11-13** in deuterated water.

Surfactant	Isomer	Solubility [mmol/L]
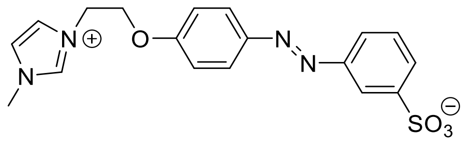	*trans*	1.3
	cis	209.9
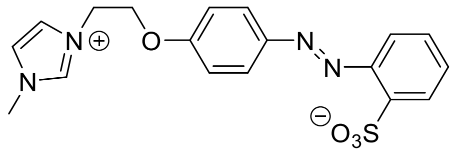	*trans*	97.5
	cis	2964*
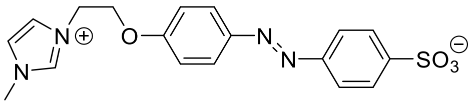	*trans*	trace
	cis	3.4

*Equals to 2560 mmol/L cis-form and 405 mmol/L trans-form in solution.

2.3. Critical micelle concentrations (CMCs)

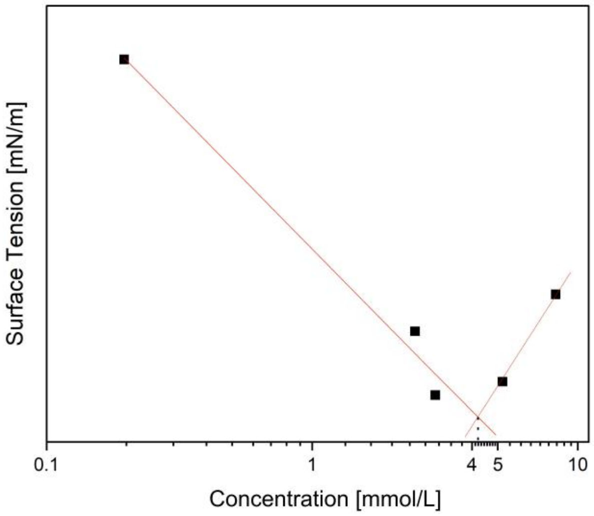

Figure S30. Surface tension as function of the concentration of *trans*-**1b** in 50% hydrogen peroxide at 50 °C. CMC values are corresponding to the dashed vertical lines.

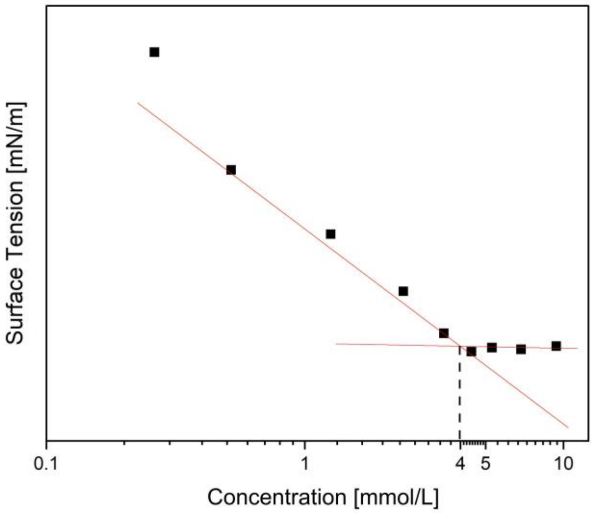

Figure S31. Surface tension as function of the concentration of *cis*-**1b** in 50% hydrogen peroxide at 50 °C. CMC values are corresponding to the dashed vertical lines.

2.4. Dynamic light scattering (DLS)

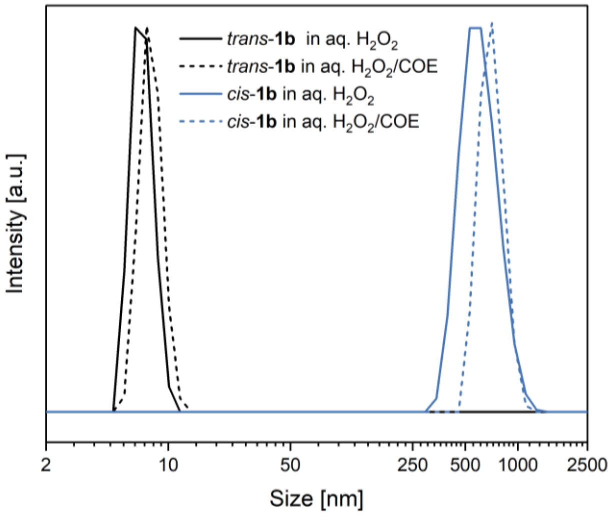

Figure S32. DLS measurement of the micellar size distribution of 5 mmol·L^{-1} **1b** in its *trans*-form prior (black, solid) and after COE treatment (black, dotted) and in its *cis*-form prior (blue) and after COE treatment in 50 wt.% aq. H_2O_2 at 20 °C, respectively.

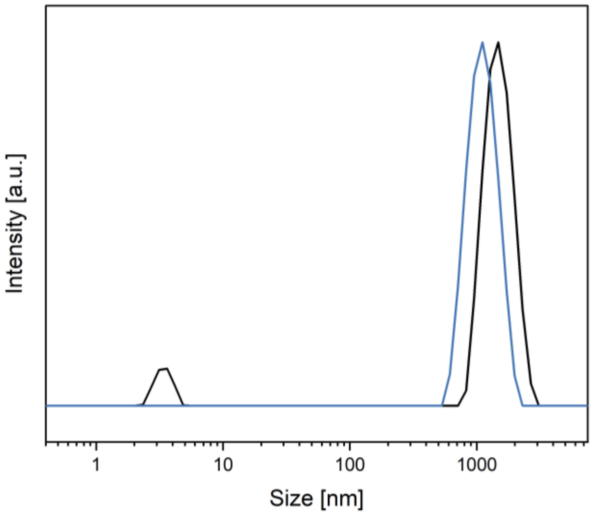

Figure S33. DLS measurement of the micellar size distribution of 5 mmol·L^{-1} **1b** in 50 H_2O in *trans*- (black) and in *cis*-form (blue) at 25 °C.

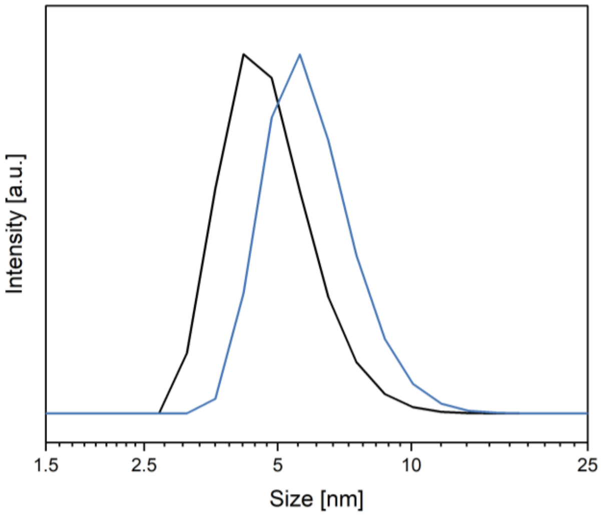

Figure S34. DLS measurement of the micellar size distribution of 5 mmol·L^{-1} **3b** in 50 wt.% aq. H_2O_2 in *trans*- (black) and in *cis*-form (blue) at 20 °C.

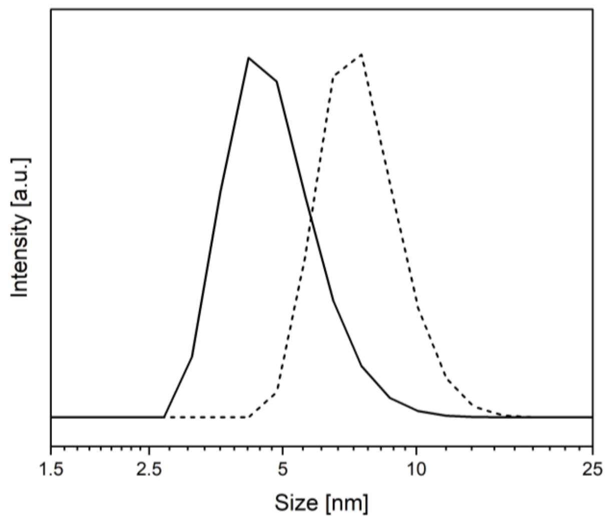

Figure S35. DLS measurement of the micellar size distribution of 5 mmol·L^{-1} *trans*-**3b** in 50 wt.% aq. H_2O_2 prior (solid) and after COE treatment (dotted) at 20 °C.

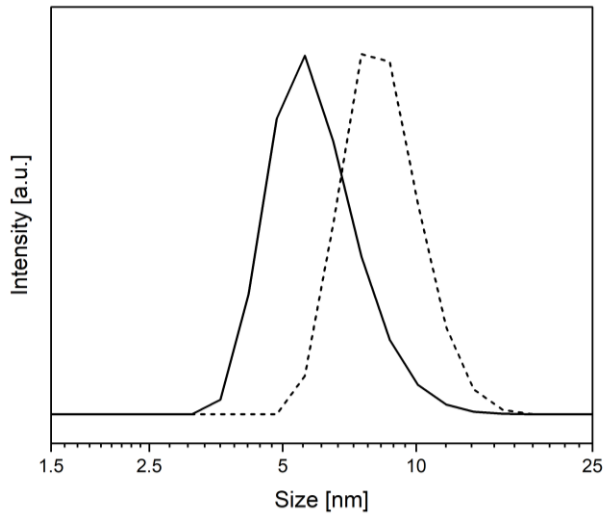

Figure S36. DLS measurement of the micellar size distribution of 5 mmol·L^{-1} *cis*-**3b** in 50 wt.% aq. H_2O_2 prior (solid) and after COE treatment (dotted) at 20 °C.

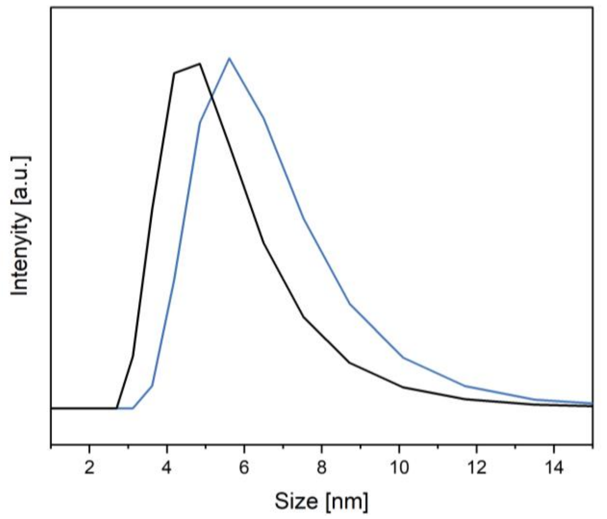

Figure S37. DLS measurement of the micellar size distribution of 5 mmol·L^{-1} **5b** in 50 wt.% aq. H_2O_2 in *trans*- (black) and in *cis*-form (blue) at 20 °C.

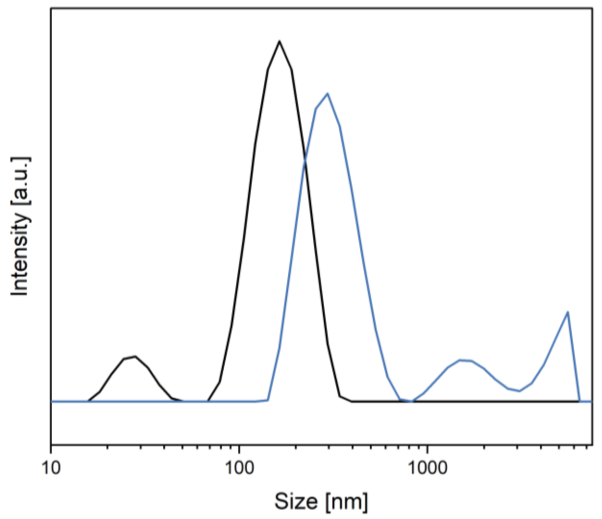

Figure S38. DLS measurement of the micellar size distribution of 10 mmol·L^{-1} **11** in 50 wt.% aq. H_2O_2 in *trans*- (black) and in *cis*-form (blue) at 20 °C.

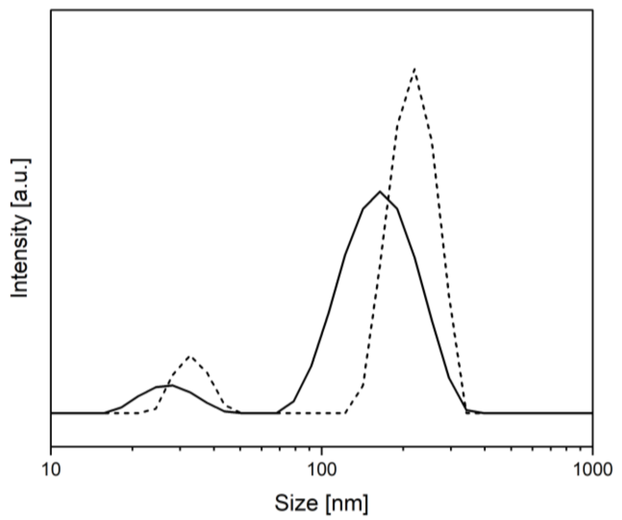

Figure S39. DLS measurement of the micellar size distribution of 10 mmol·L^{-1} *trans*-**11** in 50 wt.% aq. H_2O_2 (solid) and after COE treatment (dashed) at 20 °C.

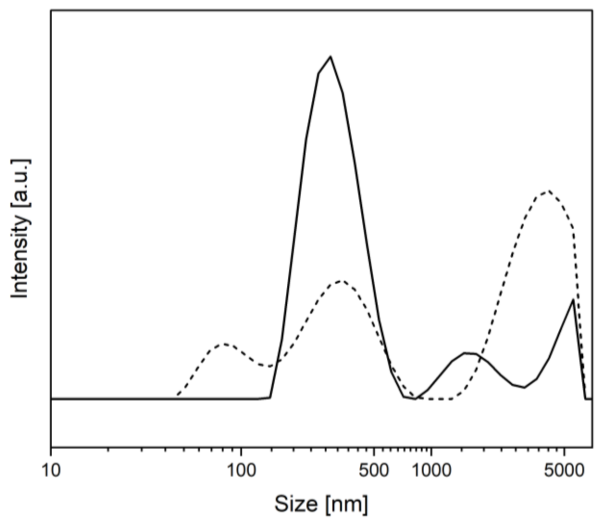

Figure S40. DLS measurement of the micellar size distribution of 10 mmol·L^{-1} *cis*-**11** in 50 wt.% aq. H_2O_2 (solid) and after COE treatment (dashed) at 20 °C.

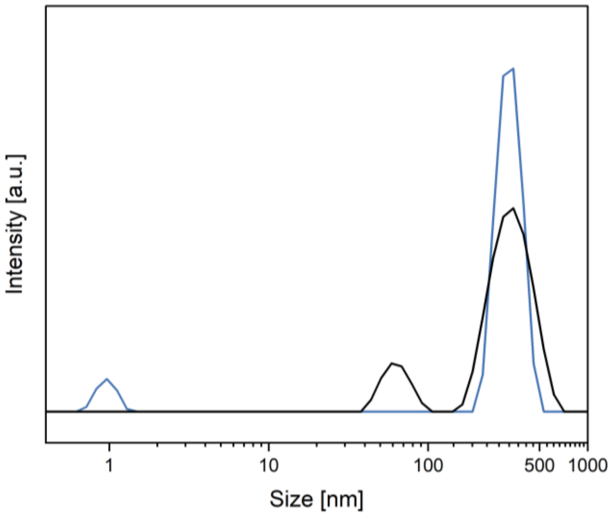

Figure S41. DLS measurement of the micellar size distribution of 10 mmol·L^{-1} **11** in D_2O in *trans*- (black) and in *cis*-form (blue) at 25 °C.

.

2.5. Transmission electron microscopy (TEM)

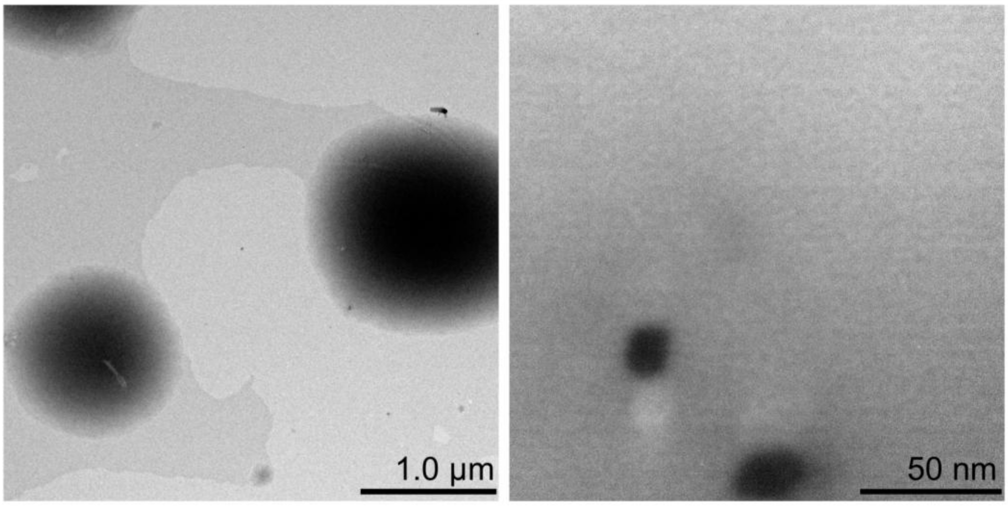

Figure S42. TEM images of 178 mmol/L solutions of **1b** in H_2O. Left: **1b** in its *cis*-form, which favorably forms large micellar aggregates. Right: **1b** in its *trans*-form, which favorably forms smaller micellar aggregates.

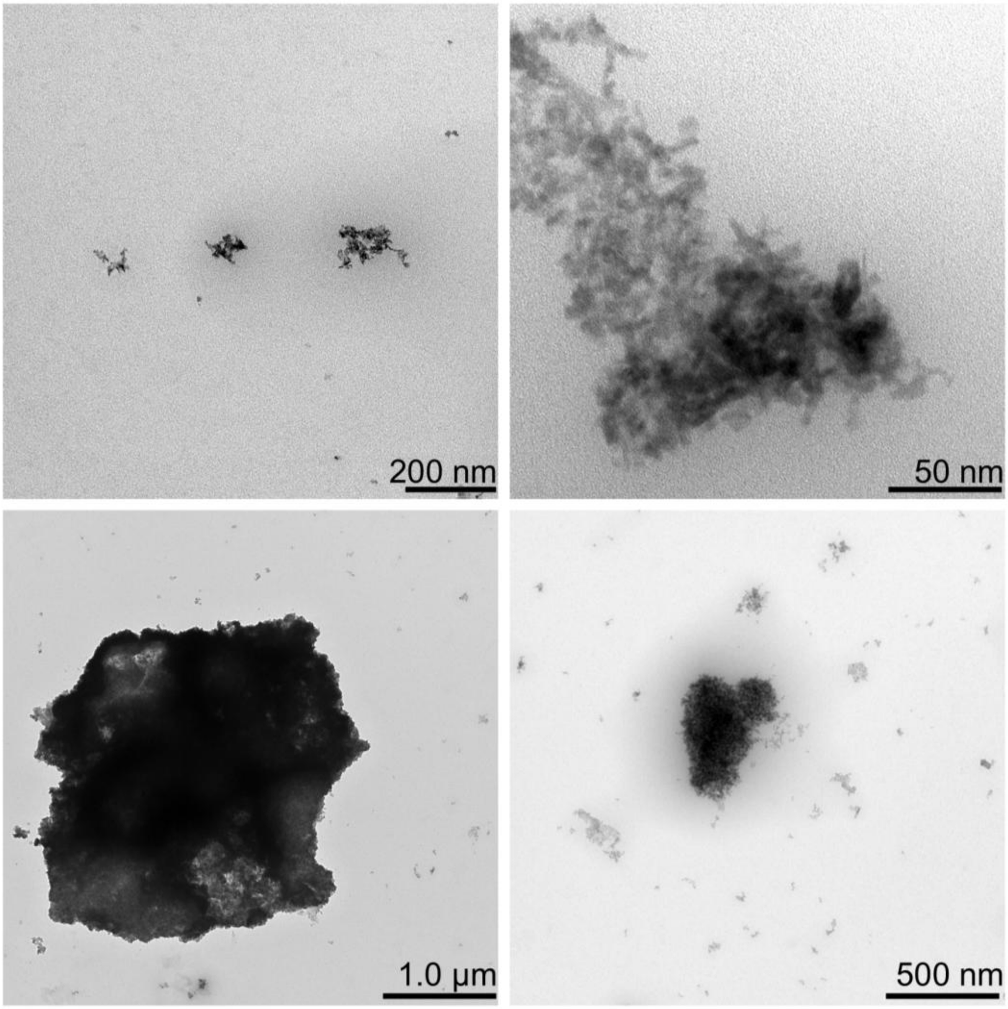

Figure S43. a) TEM images of a 10 mmol/L solution of **11** in its *cis*-form in D_2O.

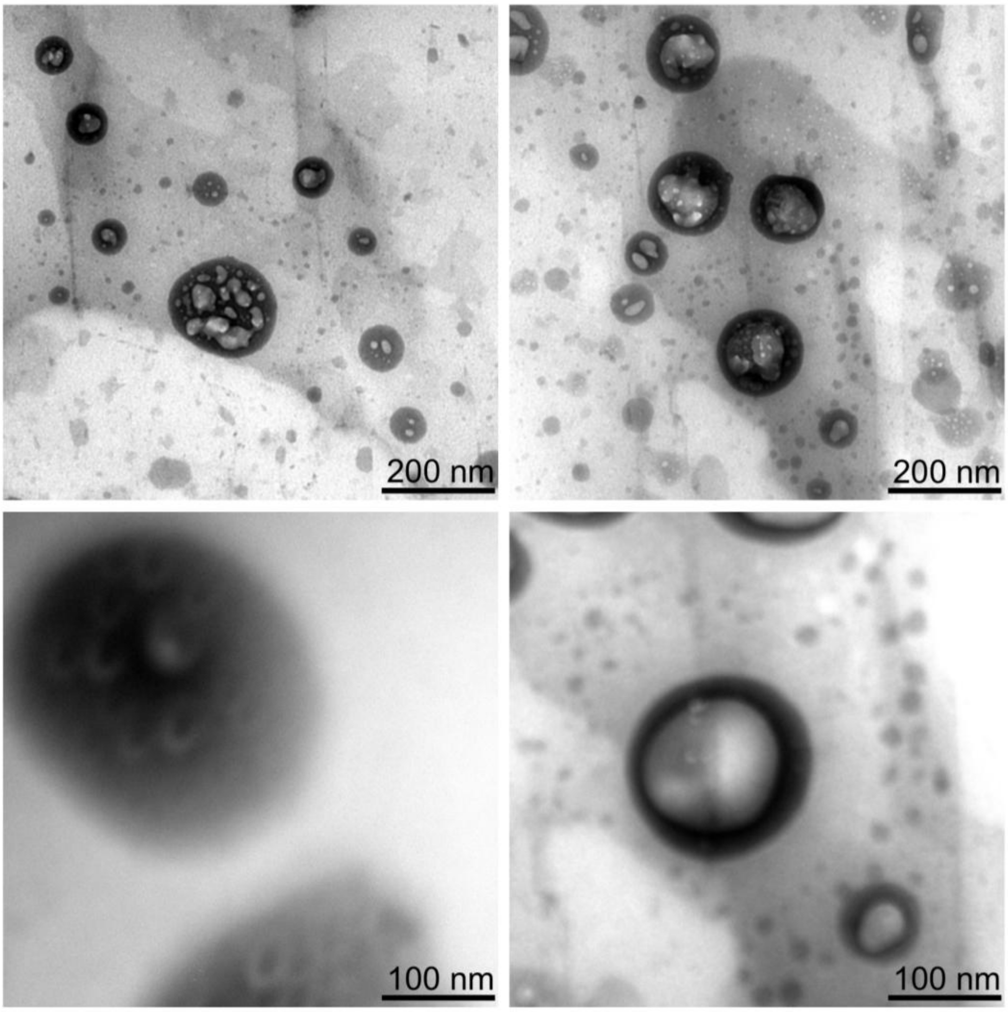

Figure S44. a) TEM images of the aqueous phase of a 10 mmol/L solution of **11** in its *cis*-form in D_2O treated with an excess of cyclooctene.

3. Epoxidation catalysis

Standard catalytic experiments were performed in 4 ml vials or 20 ml 8-dram TraceClean® vials with an air-tight cap depending on the scale.

3.1. Epoxidation catalysis with perrhenate AzoSAILs

For standard catalysis experiments with perrhenate AzoSAILs, 5 mol % of the respective AzoSAIL (0.5 mmol, 5.0 equiv.) are weight in before 50 wt.% aqueous hydrogen peroxide (1.4 mL, 25 mmol, 250 equiv.) and the internal standard mesitylene (69 µL, 5.0 mol %) were added and heated to 80 °C in an oil bath. Subsequently, cyclooctene (1.3mL, 10mmol, 100 equiv.) was added and the biphasic reaction mixture stirred at 500 rpm using a 20 mm cross-shaped magnetic stirring bar. For kinetic data after certain time intervals, the stirring was stopped and after phase separation aliquots were taken from the organic top layer and investigated by ^{1}H-NMR.

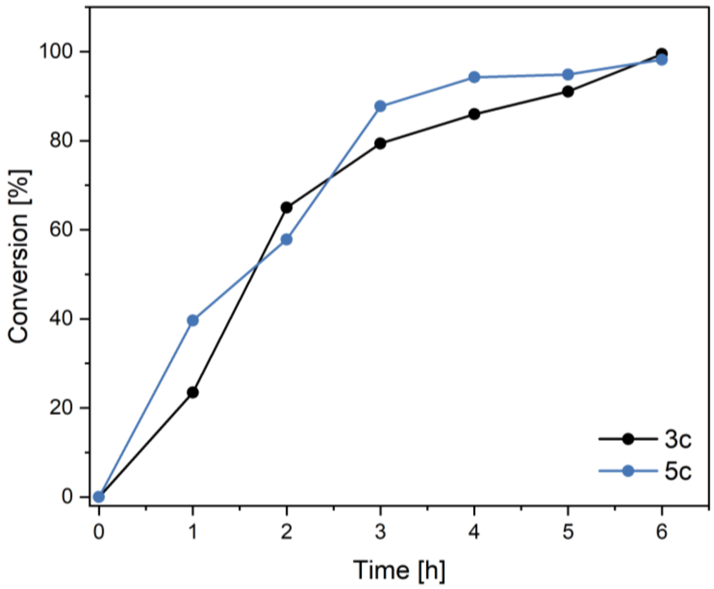

Figure S45. Kinetic curves of the epoxidation of *cis*-cyclooctene with different AzoSAILs. Reaction conditions: 80 °C, 500 rpm, 5 mol% AzoSAIL (0.5 mmol, 5 equiv.)*, 1.3 mL *cis*-cyclooctene (10 mmol, 100 equiv.) and 1.4 mL 50 wt.% aq. H_2O_2 (25 mmol, 250 equiv.).

*Note that the catalyst was also transferred to the organic phase and the majority of the catalyst remained undissolved.

3.2. Epoxidation catalysis with tungstate AzoSAILs

For standard catalysis experiments with tungstate AzoSAILs, 2.5 mol % of the AzoSAIL (0.25 mmol, 2.5 equiv.) and 79 mg phenyl phosphonic acid (0.5 mmol, 5.0 equiv.) were weight in before 50 wt.% aqueous hydrogen peroxide (1.4 mL, 25 mmol, 250 equiv.) and the internal standard mesitylene (69 µL, 5.0 mol %) were added and heated to 50 ° C in an oil bath. Subsequently, cyclooctene (10 mmol, 100 equiv.) was added and the biphasic reaction mixture stirred at 500 rpm using a 20 mm cross-shaped magnetic stirring bar. For kinetic data after certain time intervals, the stirring was stopped and after phase separation aliquots were taken from the organic top layer and investigated by ^{1}H-NMR.

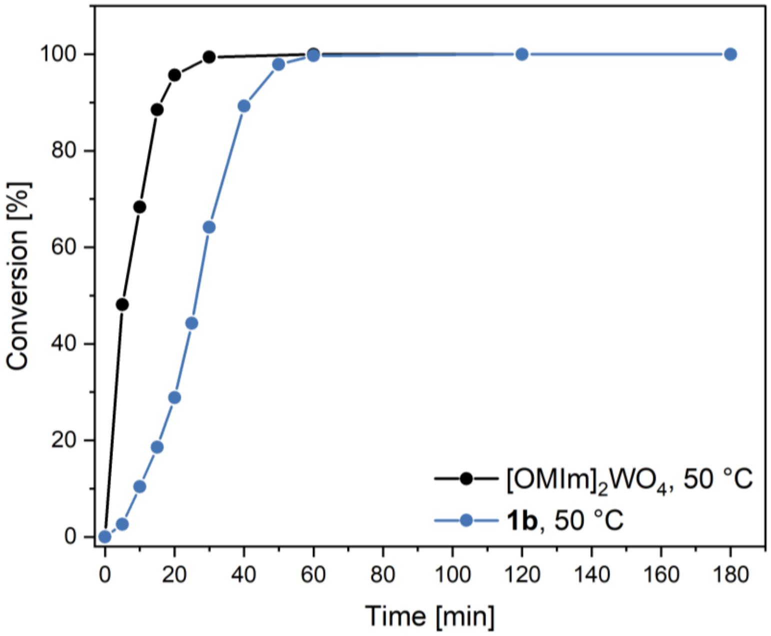

Figure S46. Comparison of the kinetics of the epoxidation of *cis*-cyclooctene with 1-octyl-3-methylimidazolium tungstate ($[OMIm_2]WO_4$) and **1b** as catalysts, respectively. Reaction conditions: 50 °C, 500 rpm, 2.5 mol% catalyst (0.25 mmol, 2.5 equiv.), 79 mg PPA (0.5 mmol, 5.0 equiv.), 1.3 mL *cis*-cyclooctene (10 mmol, 100 equiv.) and 1.4 mL 50 wt.% aq.H_2O_2 (25 mmol, 250 equiv.).

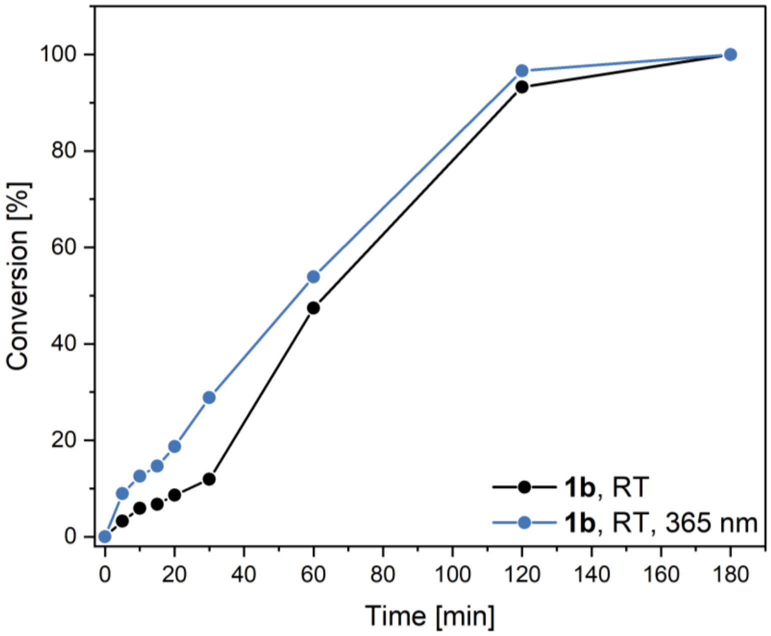

Figure S47. Comparison of the kinetics of the epoxidation of *cis*-cyclooctene with **1b** in *trans*-form and in *cis*-form (after 15 h irradiation with 365 nm), respectively. Reaction conditions: 50 °C, 500 rpm, 2.5 mol% catalyst (0.25 mmol, 2.5 equiv.), 79 mg PPA (0.5 mmol, 5.0 equiv.), 1.3 mL *cis*-cyclooctene (10 mmol, 100 equiv.) and 1.4 mL 50 wt.% aq.H_2O_2 (25 mmol, 250 equiv.).

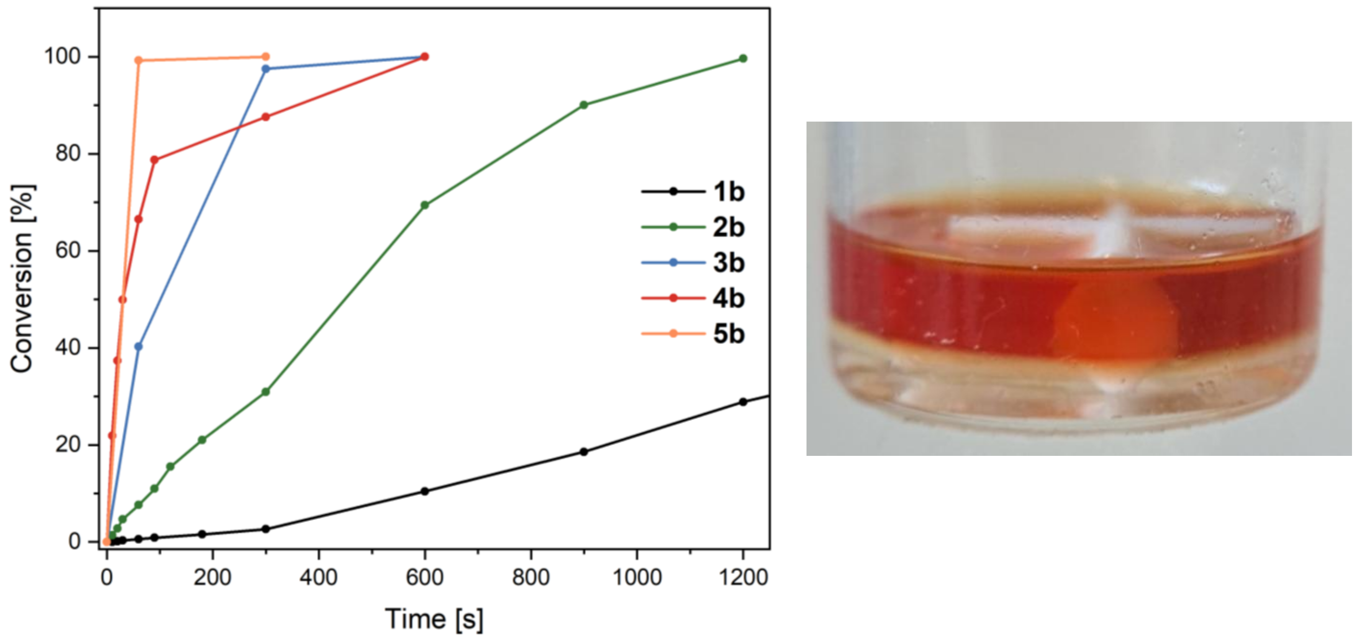

Figure S48. Left: Comparison of the kinetics of the epoxidation of *cis*-cyclooctene with different AzoSAILs in *trans*-form. Reaction conditions: 50 °C, 500 rpm, 2.5 mol% catalyst (0.25 mmol, 2.5 equiv.), 79 mg PPA (0.5 mmol, 5.0 equiv.), 1.3 mL *cis*-cyclooctene (10 mmol, 100 equiv.) and 1.4 mL 50 wt.% aq.H_2O_2 (25 mmol, 250 equiv.). Right: Reaction container after 60 s reaction time with **5b**. The colorless aqueous phase (lower phase) indicates a quantitative phase transfer of the catalyst to the organic phase (top phase, red due to catalyst).

Note that analogues experiments with 1a and 1b (with PPA) were performed using a catalyst concentration of 10 mmol/L (instead of 357 mmol/L or 178 mmol/L for perrhenates or tungstates, respectively) at RT. The conversions resulted in 3 % for 1a and 15% for 1b (with PPA) after 16 h reaction time.

3.3. Epoxidation catalysis with zwitterionic AzoSAILs

For standard catalysis experiments with zwitterionic AzoSAILs and Na_2WO_4, 15.5 mg AzoSAIL (0.04 mmol, 3.6 equiv.) are suspended in 200 µl water and irradiated for 15 h with 365 nm to obtain a homogenous solution. The AzoSAILs solution is given to 36.3 mg $Na_2WO_4 \cdot 2H_2O$ (0.11 mmol, 10 equiv.) and 38.3 mg phenyl phosphonic acid (0.22 mmol, 20 equiv.) before 136 µl 50 wt.% aqueous hydrogen peroxide (2.4 mmol, 220 equiv.) is being added. Afterwards, the internal standard mesitylene (16 µL, 5.0 mol %) and 286 µl cyclooctene (2.2 mmol, 200 equiv.) was added and the biphasic reaction mixture stirred at 500 rpm. For kinetic data after certain time intervals, the stirring was stopped and after phase separation aliquots were taken from the organic top layer and investigated by ^1H-NMR.

For standard catalysis experiments with zwitterionic AzoSAILs and Na_2WO_4, 15.5 mg AzoSAIL (0.04 mmol, 3.6 equiv.) are suspended in 200 µl water and irradiated for 15 h with 365 nm to obtain a homogenous solution. The AzoSAILs solution is given to 1.37 mg MTO (0.01 mmol, 1.0 equiv.) and 38.3 mg phenyl phosphonic acid (0.22 mmol, 20 equiv.) before 136 µl 50 wt.% aqueous hydrogen peroxide (2.4 mmol, 220 equiv.) is being added. Afterwards, the internal standard mesitylene (16 µL, 5.0 mol %) and 286 µl cyclooctene (2.2 mmol, 200 equiv.) was added and the biphasic reaction mixture stirred at 500 rpm. For kinetic data after certain time intervals, the stirring was stopped and after phase separation aliquots were taken from the organic top layer and investigated by ^1H-NMR.

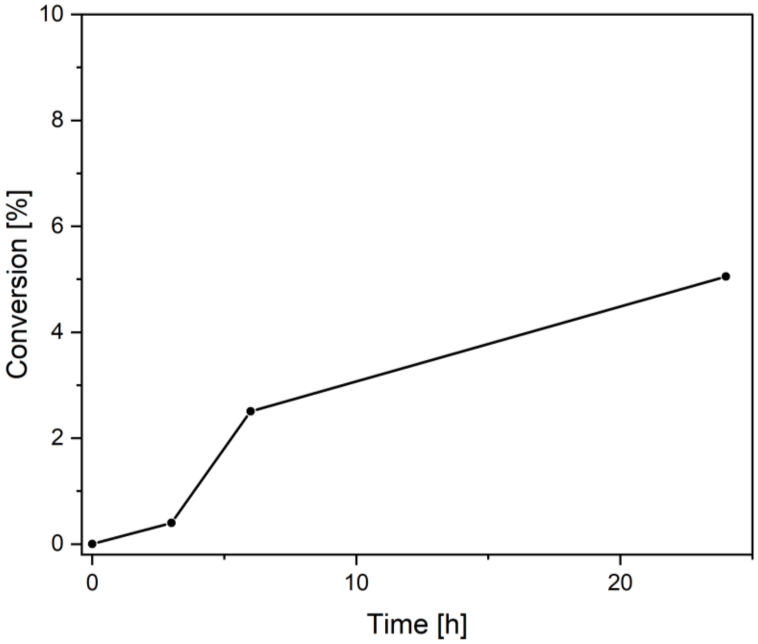

Figure S49. Kinetic curve of the epoxidation of *cis*-cyclooctene with **12**. Reaction conditions: 80 °C, 500 rpm, 5 mol% AzoSAIL (0.5 mmol, 5 equiv.), 1.3 mL *cis*-cyclooctene (10 mmol, 100 equiv.) and 1.4 mL 50 wt.% aq. H_2O_2 (25 mmol, 250 equiv.).

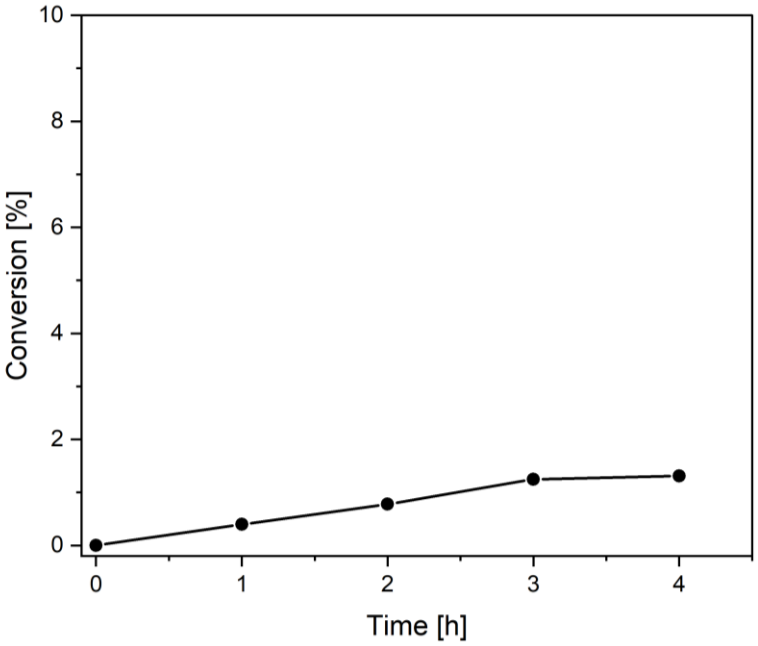

Figure S50. Kinetic curve of the epoxidation of *cis*-cyclooctene with *cis*-**11*** and Na_2WO_4. Reaction conditions: RT, 500 rpm, 1.8 mol% AzoSAIL (200 µl of a 200 mmol/L solution in H_2O, 0.04 mmol, 0.36 equiv.), 36.3 mg $Na_2WO_4 \cdot 2H_2O$ (0.11 mmol, 1.0 equiv.), 286 µL *cis*-cyclooctene (2.2 mmol, 20 equiv.) and 136 µL 50 wt.% aq. H_2O_2 (24 mmol, 22 equiv.), (Entry 1, Table S3).
*Note that **11** is isomerized by irradiation with 365 nm for 15 h in H_2O to avoid decomposition by OH-radicals formed from H_2O_2 coupled to UV irradiation.

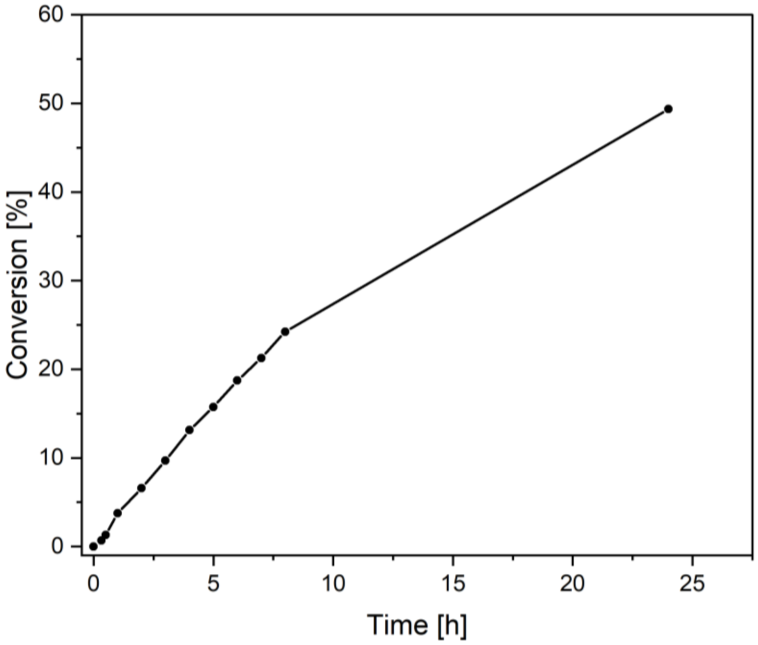

Figure S51. Kinetic curve of the epoxidation of *cis*-cyclooctene with *cis*-**11*** and Na_2WO_4/PPA. Reaction conditions: RT, 500 rpm, 1.8 mol% AzoSAIL (200 µl of a 200 mmol/L solution in H_2O, 0.04 mmol, 0.36 equiv.), 36.3 mg $Na_2WO_4 \cdot 2H_2O$ (0.11 mmol, 1.0 equiv.), 38.3 mg PPA (0.22 mmol, 2.0 equiv.), 286 µL *cis*-cyclooctene (2.2 mmol, 20 equiv.) and 136 µL 50 wt.% aq. H_2O_2 (24 mmol, 22 equiv.), (Entry 2, Table S3).

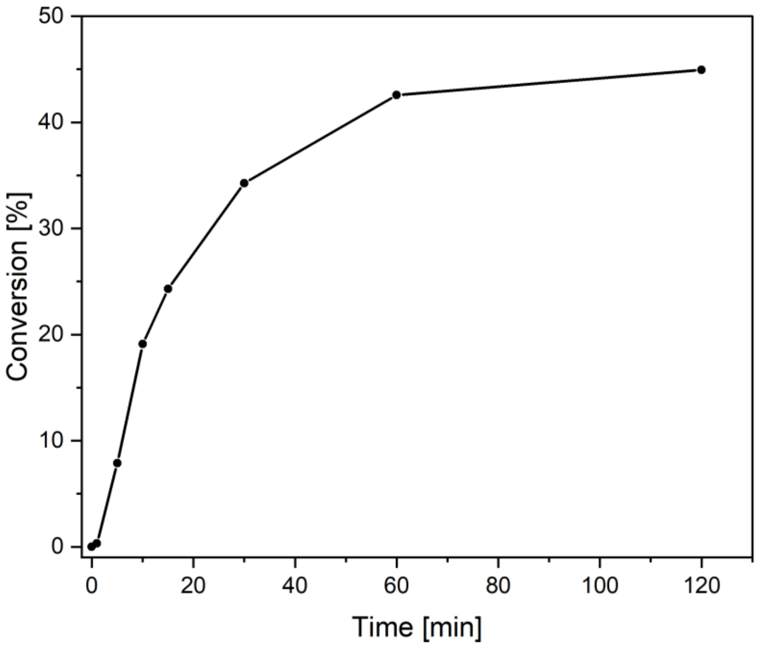

Figure S52. Kinetic curve of the epoxidation of *cis*-cyclooctene with *cis*-**11** and MTO. Reaction conditions: RT, 500 rpm, 1.8 mol% AzoSAIL (200 µl of a 200 mmol/L solution in H_2O, 0.04 mmol, 3.6 equiv.), 2.7 mg MTO (0.11 mmol, 1.0 equiv.), 286 µL *cis*-cyclooctene (2.2 mmol, 200 equiv.) and 136 µL 50 wt.% aq. H_2O_2 (24 mmol, 220 equiv.) (Entry 3, Table S3).

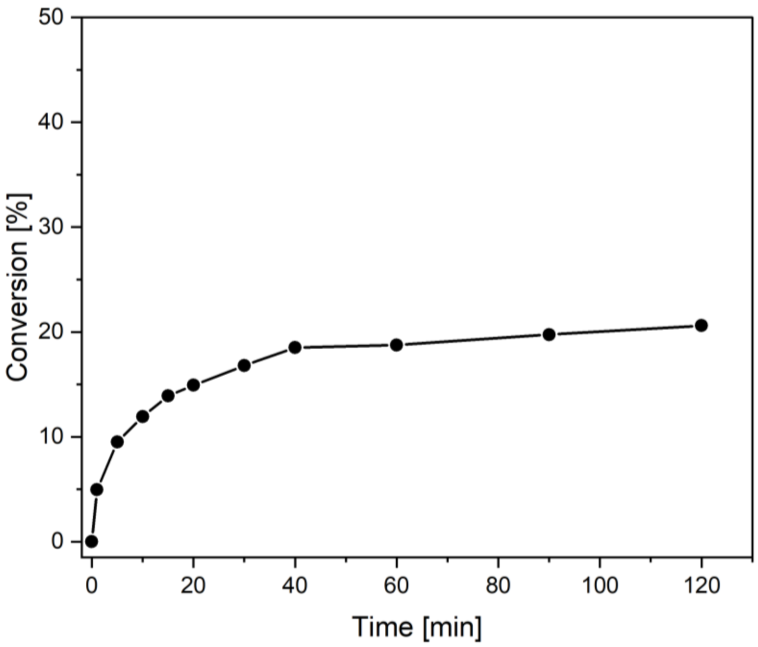

Figure S53. Kinetic curve of the epoxidation of *cis*-cyclooctene with *cis*-**11** and MTO. Reaction conditions: RT, 500 rpm, 1.8 mol% AzoSAIL (200 µl of a 200 mmol/L solution in H_2O, 0.04 mmol, 3.6 equiv.), 18.0 mg pyrazole (0.26 mmol, 24.0 eq). 2.7 mg MTO (0.11 mmol, 1.0 equiv.), 286 µL *cis*-cyclooctene (2.2 mmol, 200 equiv.) and 136 µL 50 wt.% aq. H_2O_2 (24 mmol, 220 equiv.), (Entry 4, Table S3).

Note that all further catalysis experiments in which MTO remained (partially) in the aqueous phase (Entries 1-5 and 7-8) catalyst deactivation occurred leading to a decline in activity, resulting in similar kinetic curves as the ones presented.

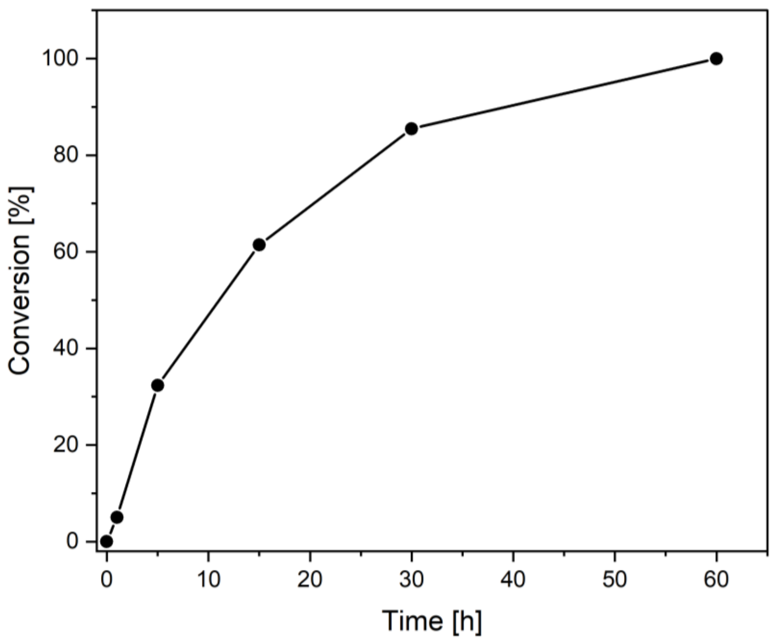

Figure S54. Kinetic curve of the epoxidation of *cis*-cyclooctene with *cis*-**11** and MTO. Reaction conditions: RT, 500 rpm, 1.8 mol% AzoSAIL (200 µl of a 200 mmol/L solution in H_2O, 0.04 mmol, 3.6 equiv.), 113.0 mg pyrazole (1.63 mmol, 250.0 eq). 2.7 mg MTO (0.11 mmol, 1.0 equiv.), 286 µL *cis*-cyclooctene (2.2 mmol, 200 equiv.) and 136 µL 50 wt.% aq. H_2O_2 (24 mmol, 220 equiv.), (Entry 6, Table S3).*

*Note that no catalyst deactivation occurred since MTO was transferred to the organic phase by the substantial excess of pyrazole, which acts as an additional phase transfer agent.

Table S3. Catalytic results of the biphasic epoxidation of COE using different catalysts coupled with the surfactant **11** after 1h reaction time. Conditions for Na_2WO_4: Surfactant : Cat. : PPA : H_2O_2 : COE = 0.36 : 1.0 : 2.0 : 22 : 20. Conditions for MTO: Surfactant : Cat. : Oxidant : COE = 3.6 : 1.0 : 220 : 200.

Entry	Catalyst	Additive	Oxidant	Temp. [°C]	Conv. [%]	Sel. [%]	Catalyst deactivation?
1	Na_2WO_4	-	H_2O_2	25	<1	>99	No[a]
2	Na_2WO_4	PPA	H_2O_2	50	4	>99	No[a]
3	MTO	-	H_2O_2	25	43	90	Yes[a]
4	MTO	24 eq. pyrazole	H_2O_2	25	19	92	Yes[a]
5	MTO	-	UHP	25	48	>99	Yes[a]
6	MTO	150 eq. pyrazole	UHP	0	100	>99	No[b]
7[c]	MTO	-	UHP	0	12	>99	Yes[a]
8[c]	MTO	24 eq. pyrazole	UHP	0	57	95	Yes[a,b]

[a] Additionally precipitation of the surfactant due to oxidation
[b] Phase transfer of the MTO to the organic phase
[c] Reaction was stopped after 30 min due to catalyst deactivation. The resulted conversions and selectivities are values for 30 min instead of 1 h.

4. Crystallographic details

4.1. Molecular structure and packing of [$C_4AzoC_6ImC_1$][ReO_4] (6a)

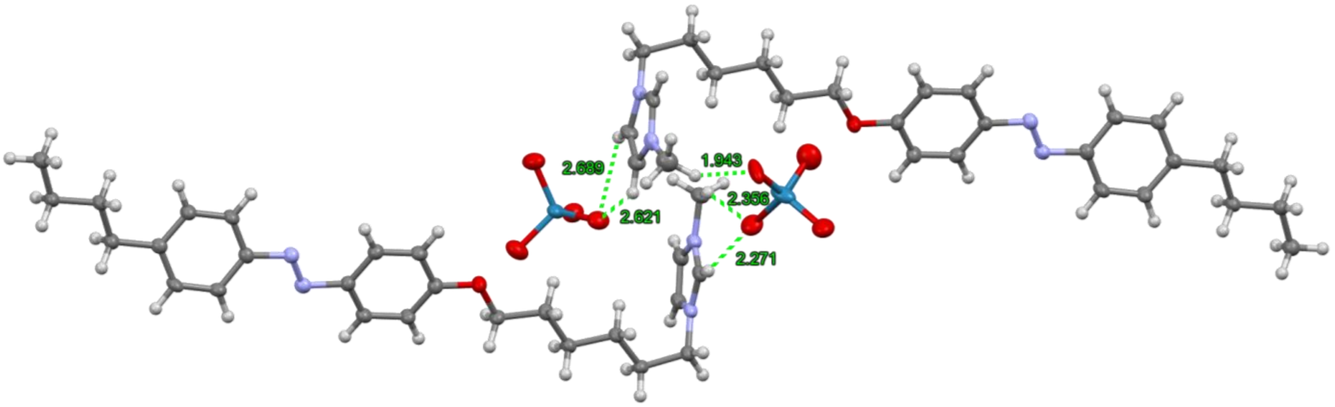

Figure S55. Molecular structure of [$C_4AzoC_6ImC_1$][ReO_4] (**6a**) obtained by single-crystal X-ray diffraction (H = white, N = blue, C = grey, Re = cyan, O = red) shown with 50 % probability displacement ellipsoids. The green lines indicate the interatomic O-H distances.

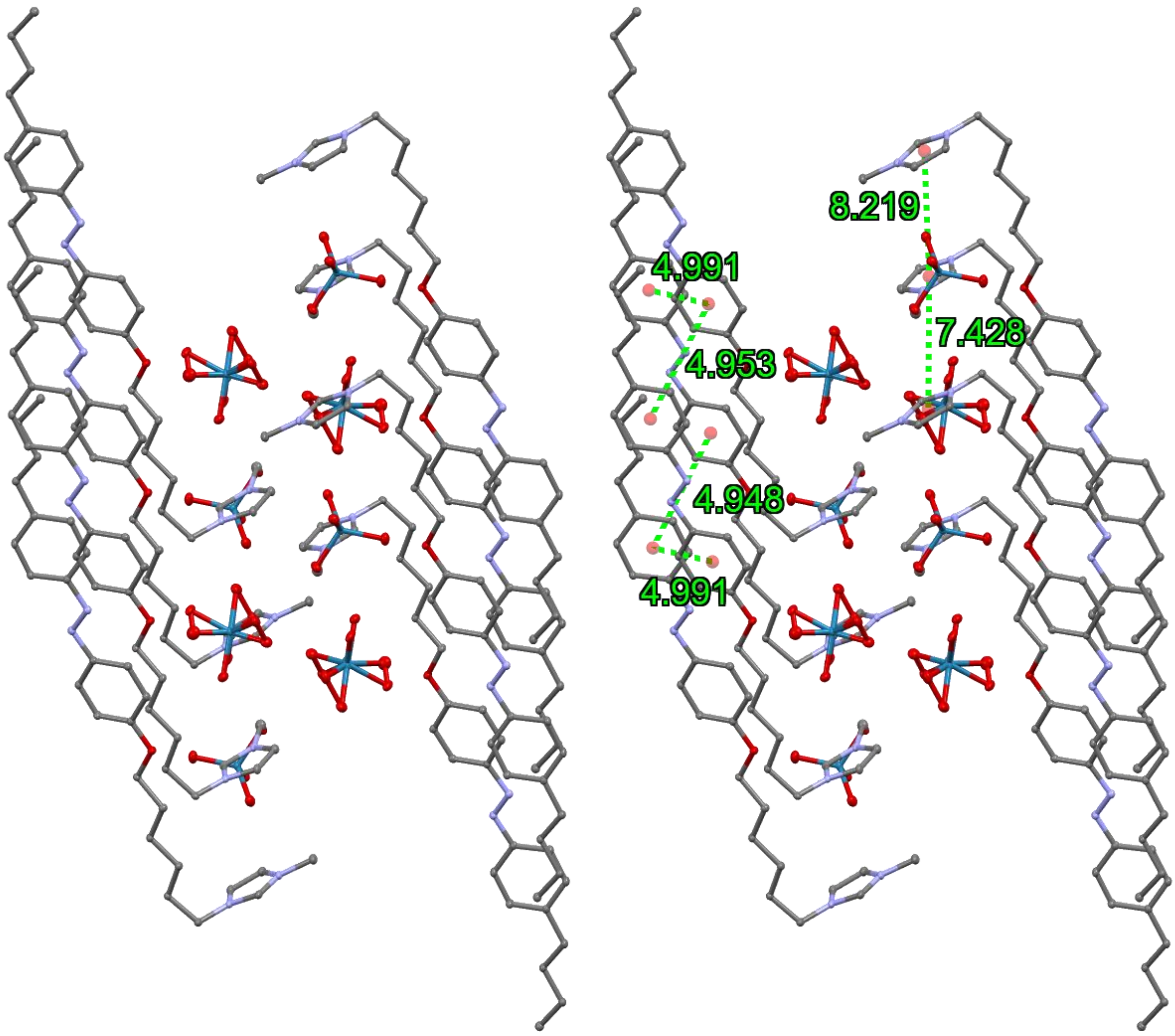

Figure S56. Packing of the solid-state structure of [$C_4AzoC_6ImC_1$][ReO_4] (**6a**) obtained by single-crystal X-ray diffraction (N = blue, C = grey, Re = cyan, O = red). Hydrogen atoms are omitted for clarity. The green lines indicate the distances of the centroids of the benzene and imidazolium rings.

4.2. Molecular structure and packing of [m-$SO_3AzoC_2ImC_1$]·H_2O (11)

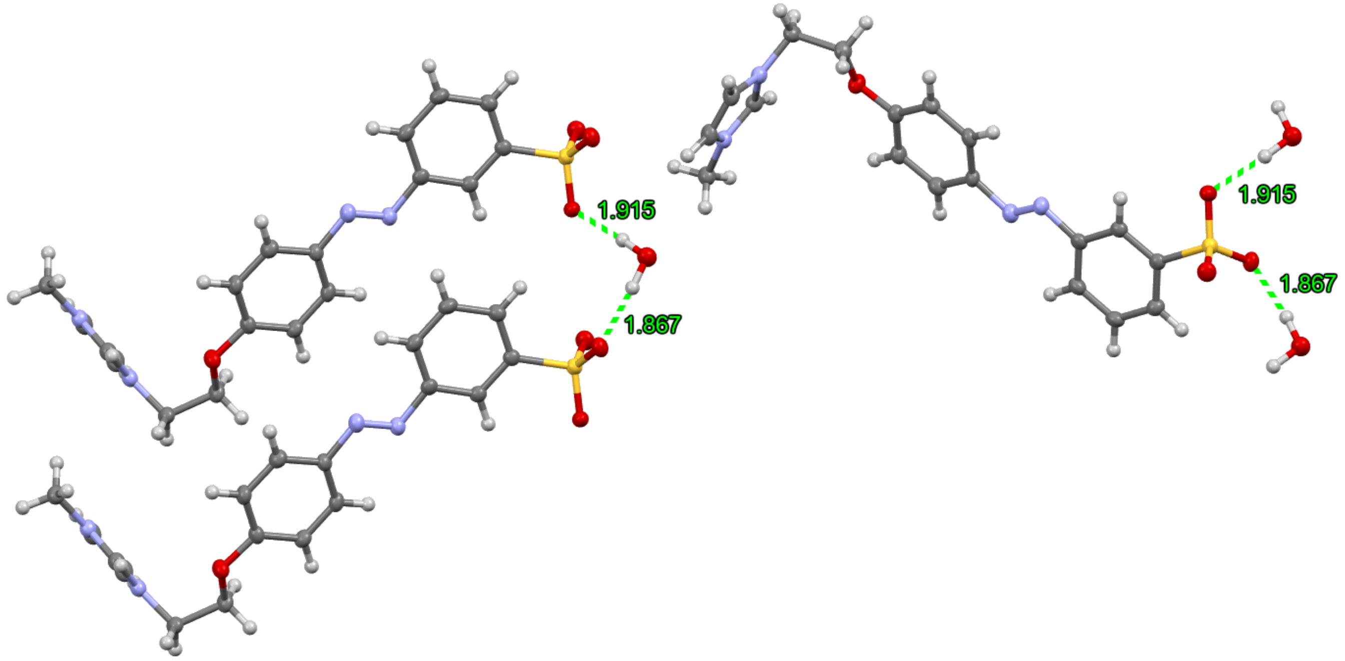

Figure S57. Molecular structure of [m-$SO_3AzoC_2ImC_1$]·H_2O (**11**) obtained by single-crystal X-ray diffraction shown with 50 % probability displacement ellipsoids (H = white, N = blue, C = grey, O = red, S = yellow) with expanded short contacts, indicating the interatomic O-H distances as green lines.

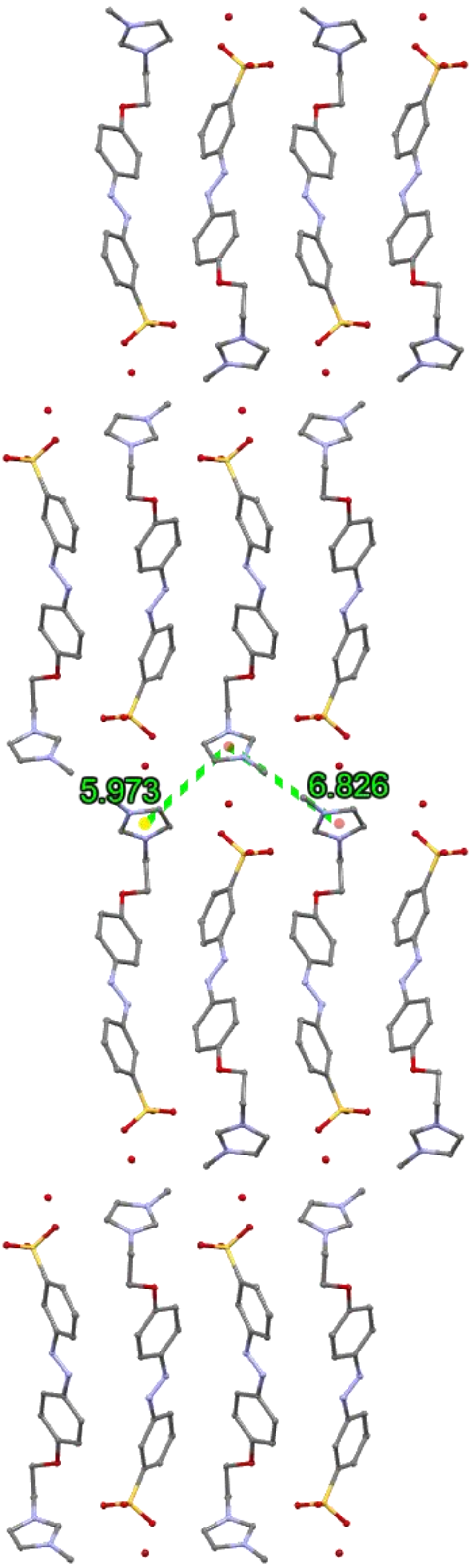

Figure S58. Packing of the solid-state structure of [*m*-$SO_3AzoC_2ImC_1$]·H_2O (**11**) obtained by single-crystal X-ray diffraction (N = blue, C = grey, O = red, S = yellow). Hydrogen atoms are omitted for clarity. The green lines indicate the distances of the centroids of the benzene and imidazolium rings.

4.3. Molecular structure and packing of [p-$SO_3AzoC_2ImC_1$]·H_2O (13)

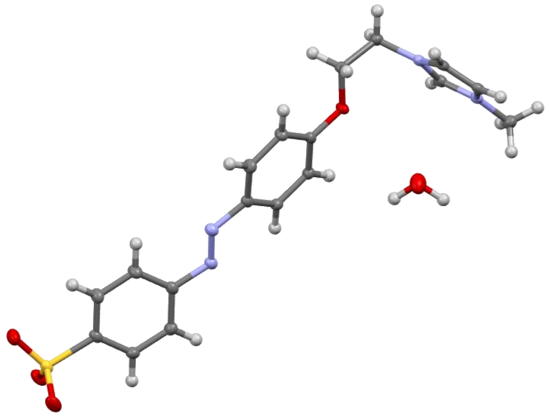

Figure S59. Molecular structure of [p-$SO_3AzoC_2ImC_1$]·H_2O (**13**) obtained by single-crystal X-ray diffraction shown with 50 % probability displacement ellipsoids (H = white, N = blue, C = grey, O = red, S = yellow).

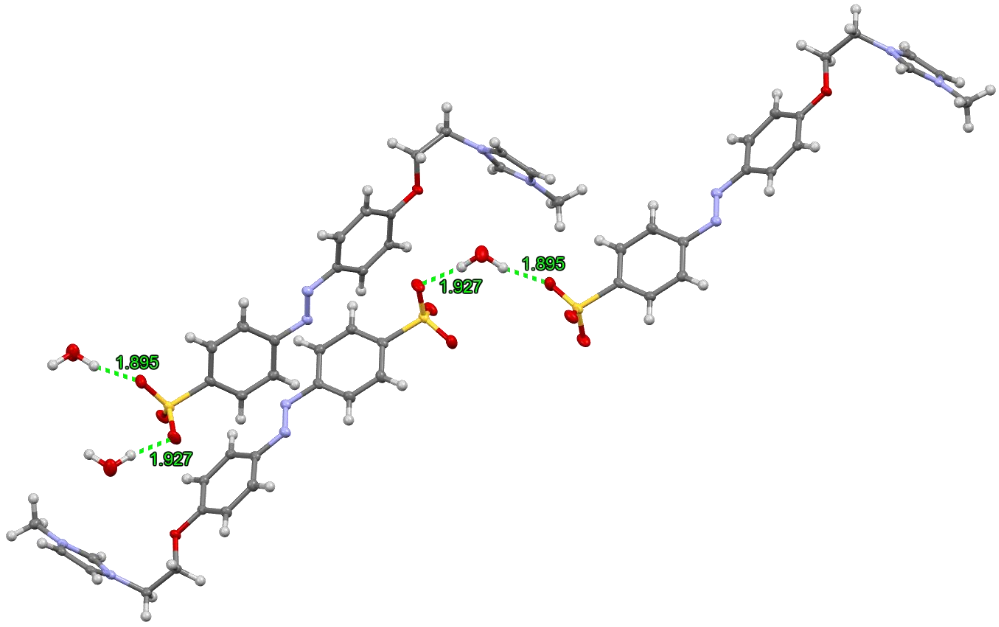

Figure S60. Molecular structure of [p-$SO_3AzoC_2ImC_1$]·H_2O (**13**) obtained by single-crystal X-ray diffraction shown with 50 % probability displacement ellipsoids (H = white, N = blue, C = grey, O = red, S = yellow) with expanded short contacts, indicating the interatomic O-H distances as green lines.

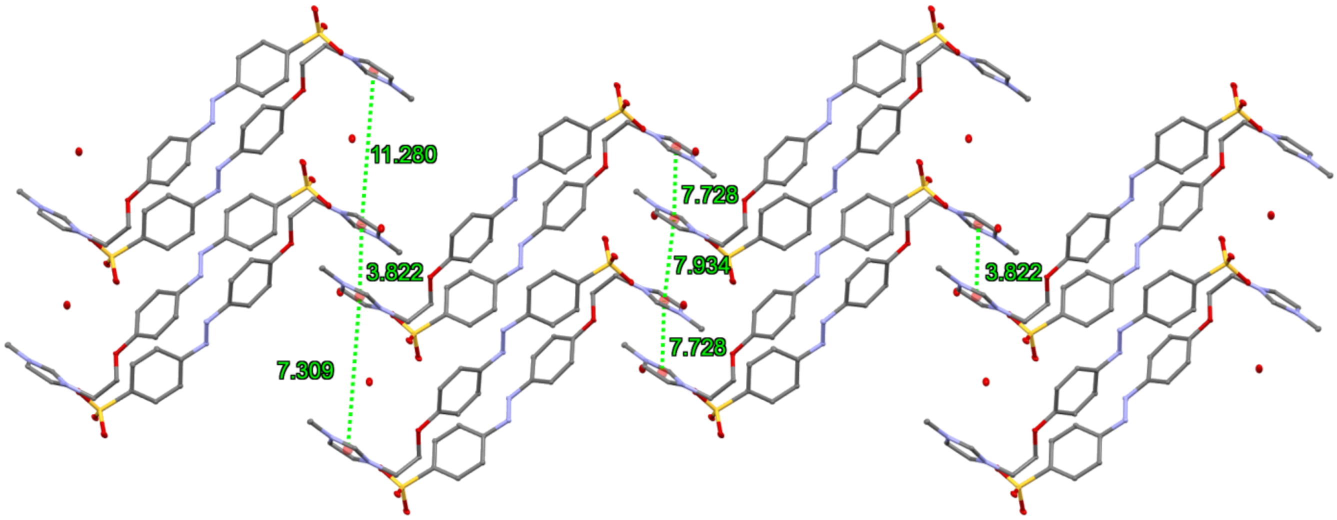

Figure S61. Packing of the solid-state structure of [p-$SO_3AzoC_2ImC_1$]·H_2O (**13**) obtained by single-crystal X-ray diffraction (N = blue, C = grey, O = red, S = yellow). Hydrogen atoms are omitted for clarity. The green lines indicate the distances of the centroids of the benzene and imidazolium rings.

4.4. Molecular structure and packing of [*m*-$SO_3BnN_2OBnC_2ImC_1$]·H_2O·CH_3ReO·H_2O

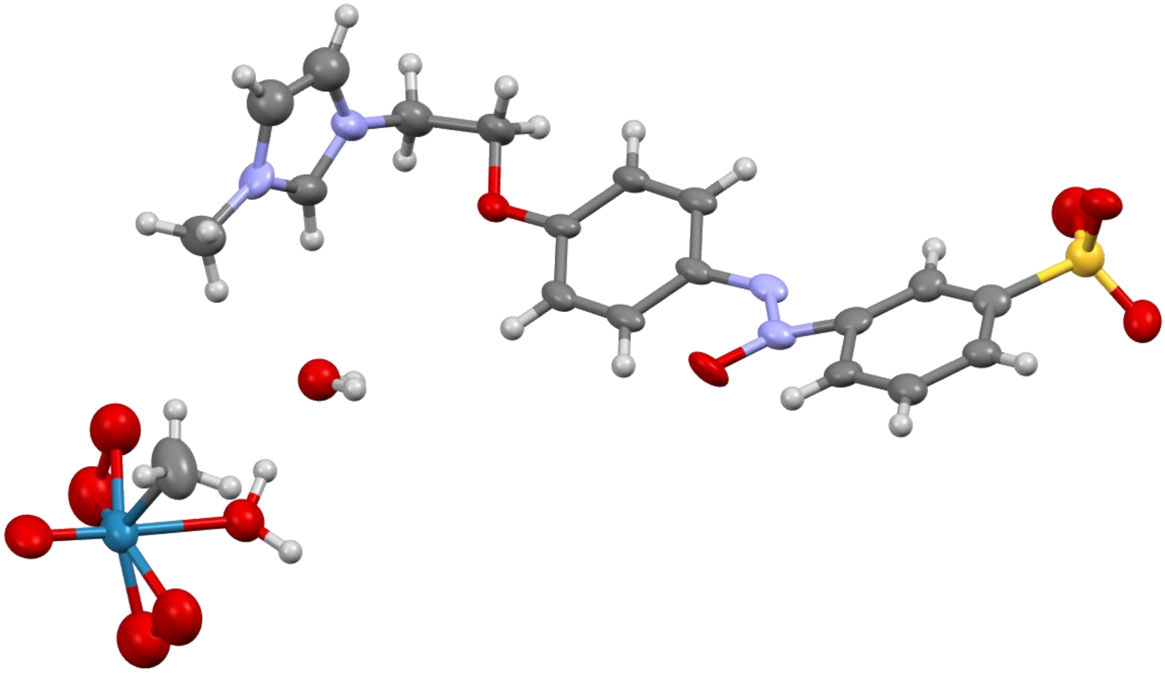

Figure S62. Molecular structure of [*m*-$SO_3BnN_2OBnC_2ImC_1$]·H_2O·CH_3ReO_6·H_2O obtained by single-crystal X-ray diffraction (H = white, N = blue, C = grey, Re = cyan, O = red, S = yellow). Ellipsoids are displayed at 50 % probability level. The disorder of the azobenzene moiety was omitted for clarity.

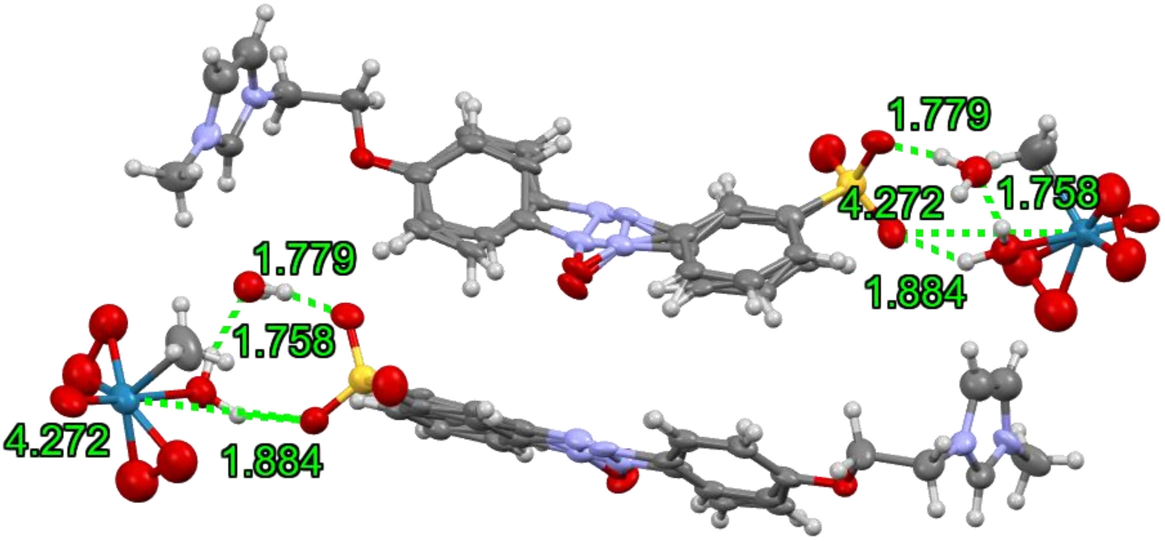

Figure S63. Molecular structure of [*m*-$SO_3BnN_2OBnC_2ImC_1$]·H_2O·CH_3ReO_6·H_2O obtained by single-crystal X-ray diffraction (H = white, N = blue, C = grey, Re = cyan, O = red, S = yellow) with expanded short contacts, indicating the interatomic O-H, O-O and O-Re distances as green lines. Note that the azobenzene moiety is disordered. Ellipsoids are displayed at 50 % probability level.

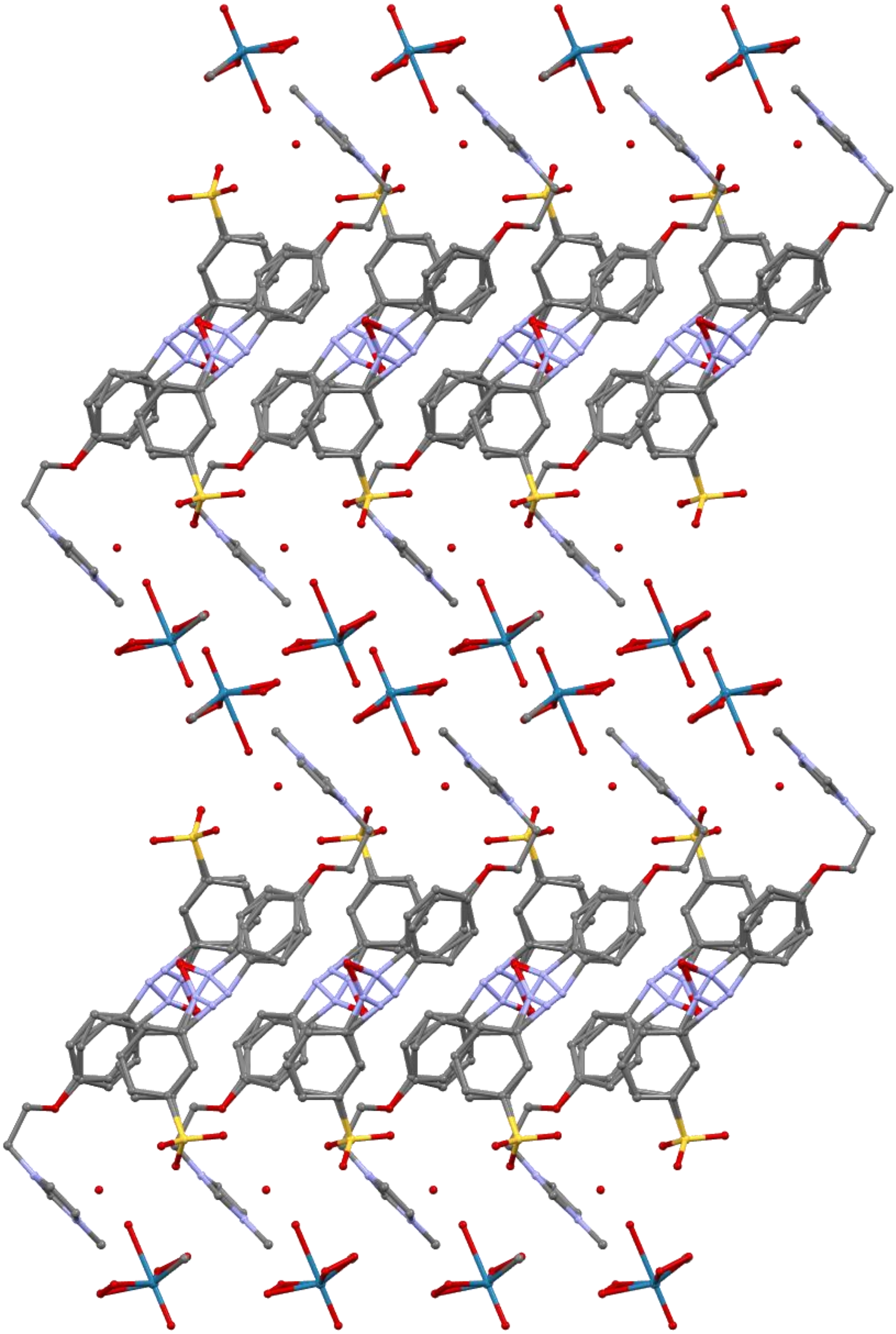

Figure S64. Packing of the solid-state structure of [*m*-$SO_3BnN_2OBnC_2ImC_1$]·H_2O·CH_3ReO_6·H_2O obtained by single-crystal X-ray diffraction (N = blue, C = grey, Re = cyan, O = red, S = yellow). Hydrogen atoms are omitted for clarity.

4.5. Crystallographic data of [$C_4AzoC_6ImC_1$][ReO_4] (6a)

A yellow, plate-shaped crystal of $C_{26}H_{35}N_4O_5Re$ coated with perfluorinated ether and fixed on top of a Kapton micro sampler was used for X-ray crystallographic analysis. The X-ray intensity data were collected at 100(2) K on a Bruker D8 VENTURE three-angle diffractometer with a TXS rotating anode with MoK_α radiation (λ=0.71073 Å) using APEX4.[8] The diffractometer was equipped with a Helios optic monochromator, a Bruker PHOTON III detector, and an Oxford Cryostream low temperature device.
A matrix scan was used to determine the initial lattice parameters. All data were integrated with the Bruker SAINT V8.40B software package using a narrow-frame algorithm and the reflections were corrected for Lorentz and polarization effects, scan speed, and background.[9] The integration of the data using a triclinic unit cell yielded a total of 106491 reflections within a 2θ range [°] of 4.19 to 50.94 (0.83 Å), of which 9878 were independent. Data were corrected for absorption effects including odd and even ordered spherical harmonics by the multi-scan method (SADABS 2016/2).[10] Space group assignment was based upon systematic absences, E statistics, and successful refinement of the structure.
The structure was solved by direct methods using SHELXT and refined by full-matrix least-squares methods against F^2 by minimizing $\Sigma w(F_o^2 - F_c^2)^2$ using SHELXL in conjunction with SHELXLE.[11–13] All non-hydrogen atoms were refined with anisotropic displacement parameters. Hydrogen atoms were refined isotropically on calculated positions using a riding model with their U_{iso} values constrained to 1.5 times the U_{eq} of their pivot atoms for terminal sp^3 carbon atoms and a C–H distance of 0.98 Å. Non-methyl hydrogen atoms were refined using a riding model with methylene, aromatic, and other C–H distances of 0.99 Å, 0.95 Å, and 1.00 Å, respectively, and U_{iso} values constrained to 1.2 times the U_{eq} of their pivot atoms.
Neutral atom scattering factors for all atoms and anomalous dispersion corrections for the non-hydrogen atoms were taken from International Tables for Crystallography.[14] Supplementary crystallographic data reported in this paper have been deposited with the Cambridge Crystallographic Data Centre (CCDC 2478998) and can be obtained free of charge from The Cambridge Crystallographic Data Centre via www.ccdc.cam.ac.uk/structures.[15] This report and the CIF file were generated using FinalCif.[16]

Table S4. Crystal data and structure refinement for compound [$C_4AzoC_6ImC_1$][ReO_4] (**6a**).

CCDC number	2478998
Empirical formula	$C_{26}H_{35}N_4O_5Re$
Formula weight	669.78
Temperature [K]	100(2)
Crystal system	triclinic
Space group (number)	$P\overline{1}$ (2)
a [Å]	9.7434(10)
b [Å]	12.2866(13)
c [Å]	23.128(2)
α [°]	77.496(4)
β [°]	87.586(4)
γ [°]	82.783(4)
Volume [Å^3]	2681.4(5)
Z	4
ρ_{calc} [gcm^{-3}]	1.659
μ [mm^{-1}]	4.574
F(000)	1336
Crystal size [mm^3]	0.057×0.095×0.106
Crystal colour	yellow
Crystal shape	plate
Radiation	MoK_α (λ=0.71073 Å)
2θ range [°]	4.19 to 50.94 (0.83 Å)
Index ranges	−11 ≤ h ≤ 11<br>−14 ≤ k ≤ 14<br>−27 ≤ l ≤ 27
Reflections collected	106491
Independent reflections	9878<br>R_{int} = 0.0703<br>R_{sigma} = 0.0349
Completeness to θ = 25.242°	99.9
Data / Restraints / Parameters	9878 / 724 / 700
Goodness-of-fit on F^2	1.064
Final *R* indexes [I≥2σ(I)]	R_1 = 0.0303<br>wR_2 = 0.0733
Final *R* indexes [all data]	R_1 = 0.0436<br>wR_2 = 0.0801
Largest peak/hole [eÅ^{-3}]	1.98/−0.63

4.6. Crystallographic data of [m-SO_3AzoC_2ImC_1]·H_2O (11)

A yellow, block-shaped crystal of $C_{18}H_{20}N_4O_5S$ coated with perfluorinated ether and fixed on top of a Kapton micro sampler was used for X-ray crystallographic analysis. The X-ray intensity data were collected at 100(2) K on a Bruker D8 VENTURE Duo three-angle diffractometer with an IMS microsource with MoK_α radiation (λ=0.71073 Å) using APEX4.[8] The diffractometer was equipped with a Helios optic monochromator, a Bruker PHOTON II detector, and an Oxford Cryostream low temperature device. A matrix scan was used to determine the initial lattice parameters. All data were integrated with the Bruker SAINT V8.40B software package using a narrow-frame algorithm and the reflections were corrected for Lorentz and polarisation effects, scan speed, and background.[9] The integration of the data using a orthorhombic unit cell yielded a total of 60457 reflections within a 2θ range [°] of 4.51 to 52.75 (0.80 Å), of which 3813 were independent. Data were corrected for absorption effects including odd and even ordered spherical harmonics by the multi-scan method (SADABS 2016/2).[10] Space group assignment was based upon systematic absences, E statistics, and successful refinement of the structure. The structure was solved by direct methods using SHELXT and refined by full-matrix least-squares methods against F2 by minimizing Σw(Fo 2 - Fc 2)2 using SHELXL in conjunction with SHELXLE.[11–13] All non-hydrogen atoms were refined with anisotropic displacement parameters. Hydrogen atoms were refined isotropically on calculated positions using a riding model with their U_{iso} values constrained to 1.5 times the U_{eq} of their pivot atoms for terminal sp3 carbon atoms and a C–H distance of 0.98 Å. Non-methyl hydrogen atoms were refined using a riding model with methylene, aromatic, and other C–H distances of 0.99 Å, 0.95 Å, and 1.00 Å, respectively, and U_{iso} values constrained to 1.2 times the U_{eq} of their pivot atoms. Neutral atom scattering factors for all atoms and anomalous dispersion corrections for the non-hydrogen atoms were taken from International Tables for Crystallography.[14] Supplementary crystallographic data reported in this paper have been deposited with the Cambridge Crystallographic Data Centre (CCDC 2478999) and can be obtained free of charge from The Cambridge Crystallographic Data Centre via www.ccdc.cam.ac.uk/structures.[15] This report and the CIF file were generated using FinalCif.[16]

Table S5. Crystal data and structure refinement for compound [m-$SO_3AzoC_2ImC_1$]·H_2O (**11**).

CCDC number	2478999
Empirical formula	$C_{18}H_{20}N_4O_5S$
Formula weight	404.44
Temperature [K]	100(2)
Crystal system	orthorhombic
Space group (number)	$P2_12_12_1$
a [Å]	5.8030(6)
b [Å]	8.8981(8)
c [Å]	36.097(4)
α [°]	90
β [°]	90
γ [°]	90
Volume [Å^3]	1863.9(3)
Z	4
ρ_{calc} [gcm^{-3}]	1.441
μ [mm^{-1}]	0.213
F(000)	848
Crystal size [mm^3]	0.048×0.153×0.213
Crystal colour	yellow
Crystal shape	block
Radiation	MoK_α (λ=0.71073 Å)
2θ range [°]	4.51 to 52.75 (0.80 Å)
Index ranges	−7 ≤ h ≤ 7<br>−11 ≤ k ≤ 11<br>−45 ≤ l ≤ 45
Reflections collected	60457
Independent reflections	3813<br>R_{int} = 0.0437<br>R_{sigma} = 0.0172
Completeness to θ = 25.242°	99.9
Data / Restraints / Parameters	3813 / 3 / 263
Goodness-of-fit on F^2	1.168
Final *R* indexes [I≥2σ(I)]	R_1 = 0.0390<br>wR_2 = 0.0853
Final *R* indexes [all data]	R_1 = 0.0514<br>wR_2 = 0.0991
Largest peak/hole [eÅ^{-3}]	0.34/−0.34
Extinction coefficient	0.0124(16)

4.7. Crystallographic data of [*p*-$SO_3AzoC_2ImC_1$]·H_2O (13)

A brown, needle-shaped crystal of $C_{18}H_{20}N_4O_5S$ coated with perfluorinated ether and fixed on top of a Kapton micro sampler was used for X-ray crystallographic analysis. The X-ray intensity data were collected at 100(2) K on a Bruker D8 VENTURE three-angle diffractometer with a TXS rotating anode with MoK_α radiation (λ=0.71073 Å) using APEX4.[8] The diffractometer was equipped with a Helios optic monochromator, a Bruker PHOTON III detector, and an Oxford Cryostream low temperature device.
A matrix scan was used to determine the initial lattice parameters. All data were integrated with the Bruker SAINT V8.40B software package using a narrow-frame algorithm and the reflections were corrected for Lorentz and polarisation effects, scan speed, and background.[9] The integration of the data using a monoclinic unit cell yielded a total of 64104 reflections within a 2θ range [°] of 4.94 to 50.72 (0.83 Å), of which 3378 were independent. Data were corrected for absorption effects including odd and even ordered spherical harmonics by the multi-scan method (SADABS 2016/2).[10] Space group assignment was based upon systematic absences, E statistics, and successful refinement of the structure.
The structure was solved by direct methods using SHELXT and refined by full-matrix least-squares methods against F^2 by minimizing $\Sigma w(F_o^2 - F_c^2)^2$ using SHELXL in conjunction with SHELXLE.[11–13] All non-hydrogen atoms were refined with anisotropic displacement parameters. Hydrogen atoms were refined isotropically on calculated positions using a riding model with their U_{iso} values constrained to 1.5 times the U_{eq} of their pivot atoms for terminal sp^3 carbon atoms and a C–H distance of 0.98 Å. Non-methyl hydrogen atoms were refined using a riding model with methylene, aromatic, and other C–H distances of 0.99 Å, 0.95 Å, and 1.00 Å, respectively, and U_{iso} values constrained to 1.2 times the U_{eq} of their pivot atoms.
Neutral atom scattering factors for all atoms and anomalous dispersion corrections for the non-hydrogen atoms were taken from International Tables for Crystallography.[14] Supplementary crystallographic data reported in this paper have been deposited with the Cambridge Crystallographic Data Centre (CCDC 2479000) and can be obtained free of charge from The Cambridge Crystallographic Data Centre via www.ccdc.cam.ac.uk/structures.[15] This report and the CIF file were generated using FinalCif.[16]

Table S6. Crystal data and structure refinement for compound [*p*-$SO_3AzoC_2ImC_1$]·H_2O (**13**).

CCDC number	2479000
Empirical formula	$C_{18}H_{20}N_4O_5S$
Formula weight	404.44
Temperature [K]	100(2)
Crystal system	monoclinic
Space group (number)	$P2_1/n$ (14)
a [Å]	7.3087(5)
b [Å]	8.5920(7)
c [Å]	29.326(2)
α [°]	90
β [°]	94.450(3)
γ [°]	90
Volume [Å^3]	1836.0(2)
Z	4
ρ_{calc} [gcm^{-3}]	1.463
μ [mm^{-1}]	0.216
F(000)	848
Crystal size [mm^3]	0.037×0.066×0.127
Crystal colour	brown
Crystal shape	needle
Radiation	MoK_α (λ=0.71073 Å)
2θ range [°]	4.94 to 50.72 (0.83 Å)
Index ranges	−8 ≤ h ≤ 8<br>−10 ≤ k ≤ 10<br>−35 ≤ l ≤ 35
Reflections collected	64104
Independent reflections	3378<br>R_{int} = 0.0778<br>R_{sigma} = 0.0278
Completeness to θ = 25.242°	99.9
Data / Restraints / Parameters	3378 / 87 / 262
Goodness-of-fit on F^2	1.060
Final *R* indexes [I≥2σ(I)]	R_1 = 0.0321<br>wR_2 = 0.0745
Final *R* indexes [all data]	R_1 = 0.0441<br>wR_2 = 0.0811
Largest peak/hole [eÅ^{-3}]	0.33/−0.40

4.8. Crystallographic data of [*m*-$SO_3BnN_2OBnC_2ImC_1$]·H_2O·CH_3ReO·H_2O

A A yellow, plate-shaped crystal of $C_{19}H_{25}N_4O_{12}ReS$ coated with perfluorinated ether and fixed on top of a Kapton micro sampler was used for X-ray crystallographic analysis. The X-ray intensity data were collected at 100(2) K on a Bruker D8 VENTURE three-angle diffractometer with a TXS rotating anode with MoK_α radiation (λ=0.71073 Å) using APEX4.[8] The diffractometer was equipped with a Helios optic monochromator, a Bruker PHOTON III detector, and an Oxford Cryostreamlow temperature device.

A matrix scan was used to determine the initial lattice parameters. All data were integrated with the Bruker SAINT V8.40B software package using a narrow-frame algorithm and the reflections were corrected for Lorentz and polarisation effects, scan speed, and background.[9] The integration of the data using a monoclinic unit cell yielded a total of 31163 reflections within a 2θ range [°] of 3.80 to 51.45 (0.82 Å), of which 4798 were independent. Data were corrected for absorption effects including odd and even ordered spherical harmonics by the multi-scan method (SADABS 2016/2).[10] Space group assignment was based upon systematic absences, E statistics, and successful refinement of the structure.

The structure was solved by direct methods using SHELXT and refined by full-matrix least-squares methods against F^2 by minimizing $\Sigma w(F_o^2 - F_c^2)^2$ using SHELXL in conjunction with SHELXLE.[11–13] All non-hydrogen atoms were refined with anisotropic displacement parameters. Hydrogen atoms were refined isotropically on calculated positions using a riding model with their U_{iso} values constrained to 1.5 times the U_{eq} of their pivot atoms for terminal sp^3 carbon atoms and a C–H distance of 0.98 Å. Non-methyl hydrogen atoms were refined using a riding model with methylene, aromatic, and other C–H distances of 0.99 Å, 0.95 Å, and 1.00 Å, respectively, and U_{iso} values constrained to 1.2 times the U_{eq} of their pivot atoms.

Neutral atom scattering factors for all atoms and anomalous dispersion corrections for the non-hydrogen atoms were taken from International Tables for Crystallography.[14] Supplementary crystallographic data reported in this paper have been deposited with the Cambridge Crystallographic Data Centre (CCDC 2479001) and can be obtained free of charge from The Cambridge Crystallographic Data Centre via www.ccdc.cam.ac.uk/structures.[15] This report and the CIF file were generated using FinalCif.[16]

Table S7. Crystal data and structure refinement for compound [*m*-$SO_3BnN_2OBnC_2ImC_1$]·H_2O·CH_3ReO·H_2O

CCDC number	2479001
Empirical formula	$C_{19}H_{25}N_4O_{12}ReS$
Formula weight	719.69
Temperature [K]	100(2)
Crystal system	monoclinic
Space group (number)	$P2_1/c$ (14)
a [Å]	22.962(3)
b [Å]	9.2150(14)
c [Å]	11.9549(18)
α [°]	90
β [°]	91.616(5)
γ [°]	90
Volume [Å^3]	2528.6(7)
Z	4
ρ_{calc} [gcm^{-3}]	1.890
μ [mm^{-1}]	4.957
F(000)	1416
Crystal size [mm^3]	0.052×0.074×0.274
Crystal colour	yellow
Crystal shape	plate
Radiation	MoK_α (λ=0.71073 Å)
2θ range [°]	3.80 to 51.45 (0.82 Å)
Index ranges	−27 ≤ h ≤ 27<br>−11 ≤ k ≤ 11<br>−14 ≤ l ≤ 14
Reflections collected	31163
Independent reflections	4798<br>R_{int} = 0.1319<br>R_{sigma} = 0.0909
Completeness to θ = 25.242°	99.8
Data / Restraints / Parameters	4798 / 552 / 427
Goodness-of-fit on F^2	1.030
Final *R* indexes [I≥2σ(I)]	R_1 = 0.0792<br>wR_2 = 0.1982
Final *R* indexes [all data]	R_1 = 0.1204<br>wR_2 = 0.2239
Largest peak/hole [eÅ^{-3}]	3.04/−1.35

5. Theoretical investigations

All calculations were carried out with the ORCA program package.[17–19]. For GGA calculations, the resolution-of-identity approximation (RI) was used,[20] while hybride-GGA calculations were carried out using the RIJCOSX approximation.[21] Atom-pairwise dispersion corrections were used for all DFT calculations.[22,23] All figures and calculated structures were rendered with the ChimeraX 1.10 software.[24]

5.1 Geometry optimization

Each geometry optimization was confirmed through the absence of imaginary frequences, proving an actual energetic minimum of the structure.

For the zwitterionic compounds **11** and **12**, as well as the respective cis isomers, optimizations in the gas phase were initially carried out at the BP86-D3BJ/def2-TZVP level of theory.[25,26] These optimization led to a folding of the structures (**Error! Reference source not found.**), presumably caused by π-π stacking and the electrostatic attraction of the locally separated charges within one molecule. To exclude the effect of the functional and basis set, the calculations were repeated at the B3LYP-D3BJ/6-311+G** level of theory,[27,28] giving the same outcome (**Error! Reference source not found.**). Hence, explicit solvation through an QM/QM2 approach was implemented.[29] First, the structures were solvated by 99 water molecules using the ALPB model.[30] Then, these solvated structures were optimized at the HF-3c/XTB level of theory.[31] This approach yields non-folded structures (**Error! Reference source not found.**) on the cost of higher computational effort. To reduce the computational cost, implicit solvation was tested. The optimization was carried out at the BP86 D3BJ def2-TZVP level of theory together with the CPCM model for water.[32] The resulting structures (**Error! Reference source not found.**) are in good agreement with these obtained from explicit solvation, however, with drastically decreased computational costs. Therefore, we are confident that the implicit solvation is a solid trade-off between computational effort and preciseness of quantum mechanical properties for this system. Accordingly, geometries of the structures **10$^+$**, **10b** and **10c** were optimized following the same protocol and all further calculations are carried out with the CPCM model for water.

After successful geometry optimization, single point energy calculations were carried out at the TPSS-D4/def2-TZVPP level of theory to compare the absolute energy difference between the respective *cis* and *trans* isomer.[33,34] Those energy differences are summed up in **Table S7**.

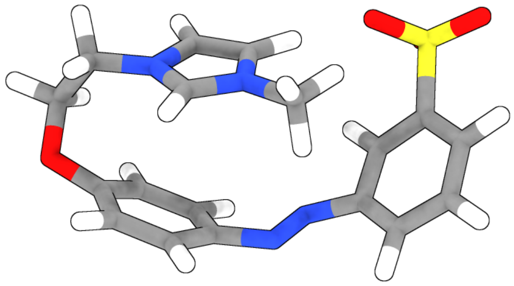

Figure S65. Optimized geometry of *trans*-**11** at the BP86-D3BJ/def2-TZVP level of theory.

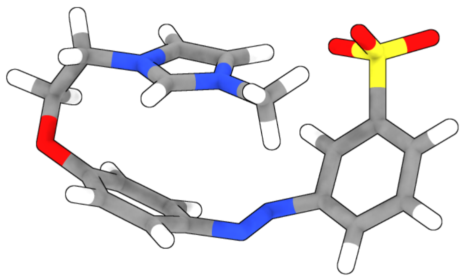

Figure S66. Optimized geometry of *trans*-**11** at the B3LYP-D3BJ/6-311+G** level of theory.

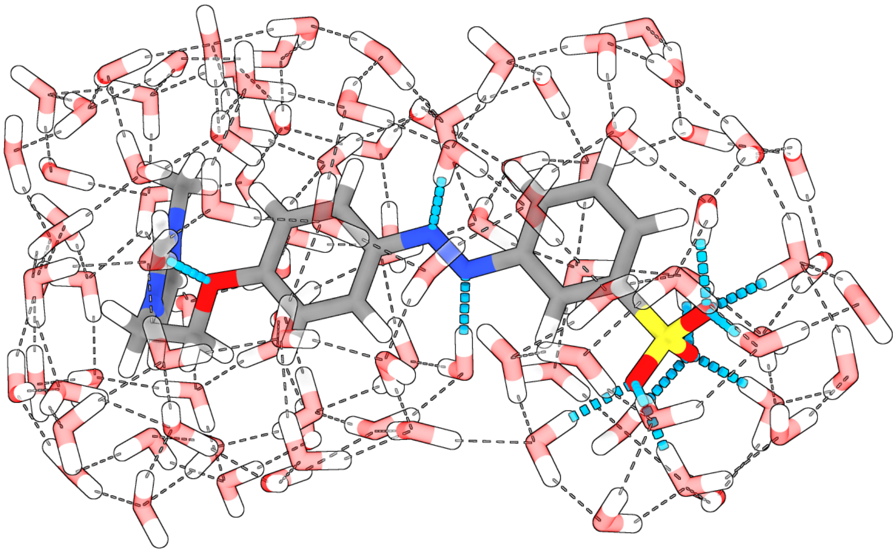

Figure S67*. Optimized geometry of *trans*-**11** using explicit solvation and a QM/QM approach at the HF-3c /XTB level of theory. Water molecules are set to a transparency of 70 % for clarity. Important hydrogen bonds are highlighted in blue.

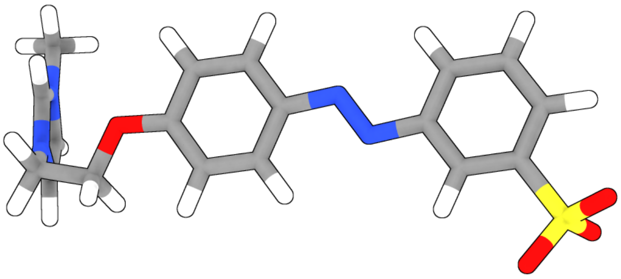

Figure S68. Optimized geometry of *trans*-**11** using the CPCM model for water at the BP86-D3BJ/def2-TZVP level of theory.

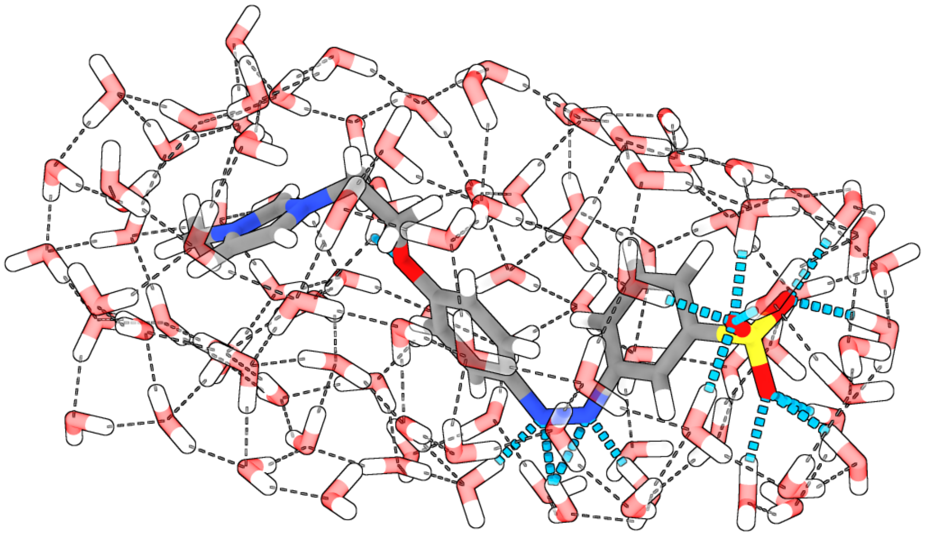

Figure S69*. Optimized geometry of *cis*-**11** using the CPCM model for water at the BP86-D3BJ/def2-TZVP level of theory.

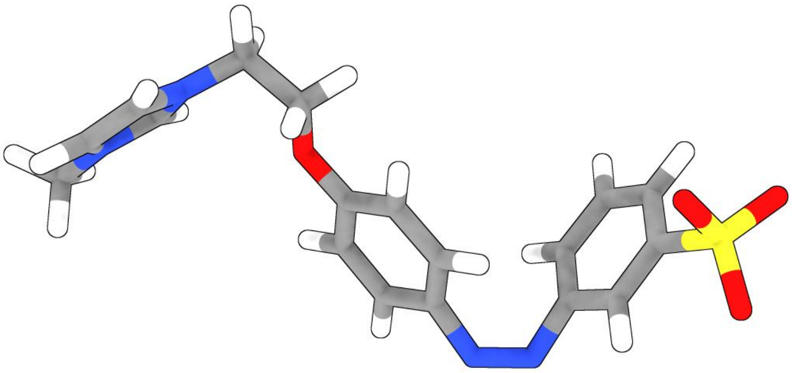

Figure S70. Optimized geometry of *cis*-**11** using the CPCM model for water at the BP86-D3BJ/def2-TZVP level of theory.

*Notably, the optimization of the structures using the explicit solvation allows the prediction of H-bonds between the molecule and the water shell. A comparison between Figure S67 and S69 suggests the enhanced interaction between the AzoSAIL and the solvent due to the configurational change. This may explain the experimentally observed solubility difference between the *trans* and *cis* isomer of **11**.

Table S8. Calculated single point energies and the energy differences between the respective *trans* and *cis* isomer.

	E_{DFT} [eh]	E_{DFT} [kcal/mol]	$\Delta E_{(trans-cis)}$ [kcal/mol]
cis-**10⁺**	-646.2028	-405494.1936	10.79
trans-**10⁺**	-646.2200	-405504.9843	
cis-**10b**	-1661.1525	-1042378.1527	24.66
trans-**10b**	-1661.1918	-1042402.8133	
cis-**10c**	-926.8416	-581595.9048	9.71
trans-**10c**	-926.8571	-581605.6138	
cis-**11**	-1615.6083	-1013799.0288	10.02
trans-**11**	-1615.6242	-1013809.0440	
cis-**12**	-1615.6089	-1013799.4619	8.04
trans-**12**	-1615.6218	-1013807.5019	
cis-**14**	-1066.7940	-669416.4427	10.06
trans-**14**	-1066.8100	-669426.5043	

5.2 Isomerization and excited states analysis of 11, 10c and 10⁺

The isomerization interconverting the *cis* and *trans* isomer of the respective structures was performed *via* a relaxed surface scan performed at the BP86-D3BJ/def2-TZVP level of theory. The dihedral angle between the heteroatomic substituents at the diazo functionality is scanned from the *cis* to the *trans* form (-11.22° to -178.94 for **11,** 14.97 to 179.51 for **10c**, respectively) in 12 steps. However, these calculations tend to fail due to an angle close to 180° between the diazo functionality and one substituent close to the transition state. In this case, the last optimized geometry was used as an input for the follow up calculation with slightly increased dihedral angle to avoid this situation resulting in 19 steps for **10c** and 18 for **11**, respectively.

For each relaxed geometry, an excited state analysis was performed using TDDFT at the wB97X-D3/def2-SVP level of theory.[35,36] The decreased size of the basis set was validated trough a comparison calculation for selected states using def2-TZVP as a basis set providing comparable results with the disadvantage of increased computational costs. For each isomerization step, the first two excited states were calculated. Additionally, triplet states, as well as spin orbit coupling were taken into account. Nevertheless, the rear two features were negligible for the interpretation of the excited states and hence, only taken into account for consistency reasons.

The isomerization of **10⁺** was investigated slightly different, nevertheless, at the same level of theory. The previous calculations indicate a transition state with a dihedral angle of roughly 90° along the C-N=N-C axis. Hence, a structure was optimized with a constrained angle at 90°, giving a starting point for further calculations. Based on this geometry, a conical intersection was optimised at the wB97X-D3/def2-SVP level of theory. Afterwards, a scan along the C-N=N-C dihedral was performed, including the angle at which the CI was found. The thereby obtained geometries were used to calculate the excited states, as described previously. The resulting scan curve indicates the possibility of thermal isomerization from *cis* to *trans*. Additionally, the presence of a conical intersection with lower energy than the first excited states of the *cis* and *trans* isomer allows for photoisomerization.

The energy of an excited state x ($\boldsymbol{E_{S_x}}$) from a TDDFT calculation can be described through the following equation:

$$E_{S_x} = E_{S(SCF)} + E_{disp} + E_{x(exc)}$$

With $\boldsymbol{E_{S(SCF)}}$ being the ground state energy obtained from the self consistent field calculational part, $\boldsymbol{E_{disp}}$ the dispersion correction, which is approximated using the D3 dispersion correction to the DFT energies, and $\boldsymbol{E_{x(exc)}}$ the excitation energy of the excited state x, corresponding to the absorption energy obtained from the TDDFT calculation. For the ground state S_0, the excitation energy is zero. From the equation above, the potential energy surfaces (PES) for the isomerization of the two structures were constructed for the ground state and the first two excited singlet states. The results are shown in the main text as well as in more detail in Table S9-14 and Figure S71-73.

To further validate the data shown above, a comparison calculation was performed by changing the level of theory to CAM-B3LYP/6-311 G**.[38] Besides increased computational cost, comparable results were obtained. Additionally, the absence of ghost states was verified using the ghost hunter index implemented in the MultiWFN software.[39–41]

The twisting of a double-bond around the N=N axis potentially involves the formation of a biradical(oid) at the transition state. Such biradicaloids potentially pose challenges for single-reference methods such as DFT. To avoid erroneous results, the transition states of the isomerization of **10c** and **11** (e.g. the state with the highest activation barrier in the potential energy curve of the ground state) was investigated with CAS-SCF-NEVPT2 to precisely describe its electronic nature and excited state energies.[42] This calculation was carried out using the QZVP basis set using the orbitals from the relaxed surface scan calculation as an input. The CASSCF-NEVPT2 calculations for **11** and **10⁺** indicate a near-degeneracy between the open-shell singlet ground states and this first excited states (6.5 and 4.18 kcal/mol, respectively), thus supporting the presence of a conical intersection and therefore indicating that DFT is adequate to qualitatively describe the photoisomerization.

However, the isomerization of **10c** does not show a conical intersection, even though the energy difference between the ground state and the first excited state is only 9.01 kcal/mol. Most likely, this system, due to the presence of a weakly coordinating anion, is too complicated to be described using the CPCM model. As discussed previously, the solvation is crucial for meaningful DFT calculations. Therefore, the absence of a CI does not mean that the photoisomerization cannot occur, it is just not describable with the here used DFT approach. However, the isomerization of **10**$^{+}$ can qualitatively be used to describe the system.

Table S9. Calculated data for the construction of the potential energy curve of the isomerization of **10⁺** from *cis* (small angle) to *trans* (large angle). Values stated with 'rel.' are referenced to the *cis* isomer energy of the respective state. Step 11* is the relaxed geometry of 11, which was obtained from the explicit search for a conical intersection.

Step	Torsion Angle [°]	$E_{S(SCF)}$ [eh]	E_{disp} [eh]	λ [nm]	$E_{1(ecx)}$ [eV]	E_{S1} [kcal/mol]	rel. E_{S1} [kcal/mol]	E_{S0} [kcal/mol]	rel. E_{S0} [kcal/mol]
1	5.00	-645.1407	-0.0125	460.70	2.6915	-404773.5075	0.0000	-404835.5689	0.0000
2	15.38	-645.1403	-0.0124	484.40	2.5594	-404776.2206	-2.7130	-404835.2369	0.3320
3	25.75	-645.1380	-0.0122	524.50	2.3639	-404779.1888	-5.6813	-404833.6975	1.8714
4	36.13	-645.1337	-0.0121	586.40	2.1143	-404782.1641	-8.6566	-404830.9161	4.6528
5	46.50	-645.1277	-0.0118	679.60	1.8243	-404784.9031	-11.3956	-404826.9693	8.5996
6	56.88	-645.1201	-0.0116	821.10	1.5101	-404787.3039	-13.7963	-404822.1239	13.4450
7	67.25	-645.1118	-0.0114	1043.60	1.1880	-404789.3893	-15.8818	-404816.7836	18.7853
8	77.63	-645.1044	-0.0112	1372.70	0.9032	-404791.1366	-17.6291	-404811.9637	23.6052
9	88.00	-645.1055	-0.0105	919.50	1.3484	-404781.1320	-7.6244	-404812.2251	23.3438
10	90.00	-645.1056	-0.0105	907.20	1.3667	-404780.7882	-7.2806	-404812.3019	23.2671
11*	90.76*	-645.1077	-0.0106	852.70	1.4540	-404780.1078	-6.6003	-404813.6345	21.9344
11	90.76	-645.0844	-0.0109	18078.90	0.0686	-404797.6671	-24.1596	-404799.2484	36.3205
12	92.00	-645.1057	-0.0105	900.70	1.3766	-404780.6026	-7.0950	-404812.3441	23.2248
13	102.25	-645.1058	-0.0105	897.70	1.3811	-404780.5419	-7.0343	-404812.3875	23.1814
14	112.50	-645.1059	-0.0104	903.30	1.3726	-404780.7998	-7.2923	-404812.4505	23.1184
15	122.75	-645.1234	-0.0103	919.60	1.3483	-404792.2583	-18.7508	-404823.3480	12.2209
16	133.00	-645.1346	-0.0101	739.90	1.6757	-404791.6485	-18.1410	-404830.2880	5.2809
17	143.25	-645.1448	-0.0100	625.50	1.9823	-404790.9107	-17.4031	-404836.6197	-1.0508
18	153.50	-645.1526	-0.0099	549.50	2.2563	-404789.4209	-15.9133	-404841.4481	-5.8791
19	163.75	-645.1580	-0.0099	503.10	2.4645	-404787.9121	-14.4046	-404844.7397	-9.1708
20	174.00	-645.1606	-0.0098	477.90	2.5943	-404786.5441	-13.0366	-404846.3644	-10.7955
21	179.61	-645.1608	-0.0098	473.90	2.6161	-404786.2024	-12.6949	-404846.5250	-10.9561

Table S10. . Calculated data for the construction of the potential energy curve of the isomerization of **10⁺** from *cis* (small angle) to *trans* (large angle). Values stated with 'rel.' are referenced to the *cis* isomer energy of the respective state. Step 11* is the relaxed geometry of 11, which was obtained from the explicit search for a conical intersection.

Step	Torsion Angle [°]	$E_{S(SCF)}$ [eh]	E_{disp} [eh]	λ [nm]	$E_{2(ecx)}$ [eV]	E_{S2} [kcal/mol]	rel. E_{S2} [kcal/mol]	E_{S0} [kcal/mol]	rel. E_{S0} [kcal/mol]
1	5.00	-645.1407	-0.0125	288.9	4.2916	-404736.6111	0.0000	-404835.5689	0.0000
2	15.38	-645.1403	-0.0124	293	4.2318	-404737.6588	-1.0477	-404835.2369	0.3320
3	25.75	-645.1380	-0.0122	298.5	4.1542	-404737.9080	-1.2969	-404833.6975	1.8714
4	36.13	-645.1337	-0.0121	305.6	4.0576	-404737.3542	-0.7431	-404830.9161	4.6528
5	46.50	-645.1277	-0.0118	314.1	3.9472	-404735.9524	0.6587	-404826.9693	8.5996
6	56.88	-645.1201	-0.0116	324.2	3.8249	-404733.9273	2.6838	-404822.1239	13.4450
7	67.25	-645.1118	-0.0114	334.7	3.7043	-404731.3687	5.2424	-404816.7836	18.7853
8	77.63	-645.1044	-0.0112	341.2	3.6336	-404728.1787	8.4324	-404811.9637	23.6052
9	88.00	-645.1055	-0.0105	318.4	3.8935	-404722.4478	14.1633	-404812.2251	23.3438
10	90.00	-645.1056	-0.0105	318.3	3.8953	-404722.4825	14.1286	-404812.3019	23.2671
11*	90.76*	-645.1077	-0.0106	319.1	3.8850	-404724.0522	12.5589	-404813.6345	21.9344
11	90.76	-645.0844	-0.0109	342.7	3.6177	-404715.8307	20.7804	-404799.2484	36.3205
12	92.00	-645.1057	-0.0105	318.3	3.8953	-404722.5251	14.0860	-404812.3441	23.2248
13	102.25	-645.1058	-0.0105	318.4	3.8940	-404722.5987	14.0124	-404812.3875	23.1814
14	112.50	-645.1059	-0.0104	318.6	3.8914	-404722.7218	13.8893	-404812.4505	23.1184
15	122.75	-645.1234	-0.0103	340.6	3.6398	-404739.4192	-2.8081	-404823.3480	12.2209
16	133.00	-645.1346	-0.0101	334.7	3.7045	-404744.8672	-8.2560	-404830.2880	5.2809
17	143.25	-645.1448	-0.0100	331.6	3.7394	-404750.3957	-13.7846	-404836.6197	-1.0508
18	153.50	-645.1526	-0.0099	331.3	3.7425	-404755.1515	-18.5404	-404841.4481	-5.8791
19	163.75	-645.1580	-0.0099	335.3	3.6978	-404759.4746	-22.8635	-404844.7397	-9.1708
20	174.00	-645.1606	-0.0098	341.1	3.6354	-404762.5382	-25.9271	-404846.3644	-10.7955
21	179.61	-645.1608	-0.0098	342.6	3.6192	-404763.0716	-26.4605	-404846.5250	-10.9561

Table S11. Calculated data for the construction of the potential energy curve of the isomerization of **10c** from *cis* (small angle) to *trans* (large angle). Values stated with 'rel.' are referenced to the *cis* isomer energy of the respective state.

Step	Torsion Angle [°]	$E_{S(SCF)}$ [eh]	E_{disp} [eh]	λ [nm]	$E_{1(ecx)}$ [eV]	E_{S1} [kcal/mol]	rel. E_{S1} [kcal/mol]	E_{S0} [kcal/mol]	rel. E_{S0} [kcal/mol]
1	14.97	-925.3018	-0.0173	472.20	2.6258	-580579.9460	0.0000	-580640.4974	0.0000
2	29.93	-925.2980	-0.0171	535.20	2.3165	-580584.5332	-4.5872	-580637.9518	2.5457
3	44.89	-925.2900	-0.0167	651.00	1.9044	-580588.8308	-8.8848	-580632.7469	7.7505
4	59.84	-925.2785	-0.0165	866.60	1.4307	-580592.3698	-12.4238	-580625.3632	15.1342
5	74.80	-925.2653	-0.0162	1319.20	0.9398	-580595.2428	-15.2968	-580616.9156	23.5818
6	83.20	-925.2590	-0.0161	1782.80	0.6955	-580596.8872	-16.9412	-580612.9247	27.5727
7	91.60	-925.2524	-0.0160	3171.60	0.39092	-580599.6670	-19.7210	-580608.6817	31.8157
8	100.00	-925.2597	-0.0157	1867.50	0.6639	-580597.7360	-17.7900	-580613.0458	27.4516
9	107.23	-925.2665	-0.0156	1400.40	0.8854	-580596.8465	-16.9005	-580617.2632	23.2343
10	114.46	-925.2753	-0.0154	1109.00	1.1179	-580596.9200	-16.9740	-580622.6999	17.7976
11	121.68	-925.2841	-0.0153	915.20	1.3548	-580596.8943	-16.9483	-580628.1361	12.3614
12	128.91	-925.2926	-0.0151	778.20	1.5933	-580596.6372	-16.6912	-580633.3782	7.1192
13	136.14	-925.3004	-0.0150	680.00	1.8232	-580596.1560	-16.2100	-580638.1987	2.2987
14	143.37	-925.3074	-0.0149	607.40	2.0413	-580595.4258	-15.4798	-580642.4991	-2.0017
15	150.60	-925.3132	-0.0148	553.20	2.2411	-580594.4268	-14.4808	-580646.1071	-5.6096
16	157.83	-925.3179	-0.0147	514.50	2.4098	-580593.4275	-13.4815	-580648.9980	-8.5005
17	165.05	-925.3211	-0.0147	484.80	2.5574	-580592.0265	-12.0805	-580651.0005	-10.5030
18	172.28	-925.3231	-0.0147	466.70	2.6563	-580591.0048	-11.0588	-580652.2607	-11.7633
19	179.51	-925.3237	-0.0148	459.80	2.6964	-580590.4994	-10.5534	-580652.6799	-12.1825

Table S12. Calculated data for the construction of the potential energy curve for the of the isomerization of **10c** from *cis* (small angle) to *trans* (large angle). Values stated with 'rel.' are referenced to the *cis* isomer energy of the respective state.

Step	Torsion Angle [°]	$E_{S(SCF)}$ [eh]	E_{disp} [eh]	λ [nm]	$E_{2(ecx)}$ [eV]	E_{S2} [kcal/mol]	rel. E_{S2} [kcal/mol]	E_{S0} [kcal/mol]	rel. E_{S0} [kcal/mol]
1	14.97	-925.3018	-0.0173	302.90	4.0934	-580546.1025	0.0000	-580640.4974	0.0000
2	29.93	-925.2980	-0.0171	309.60	4.0041	-580545.6152	0.4873	-580637.9518	2.5457
3	44.89	-925.2900	-0.0167	329.60	3.7616	-580546.0037	0.0988	-580632.7469	7.7505
4	59.84	-925.2785	-0.0165	354.70	3.4950	-580544.7671	1.3354	-580625.3632	15.1342
5	74.80	-925.2653	-0.0162	383.00	3.2375	-580542.2575	3.8450	-580616.9156	23.5818
6	83.20	-925.2590	-0.0161	390.20	3.1776	-580539.6488	6.4537	-580612.9247	27.5727
7	91.60	-925.2524	-0.0160	402.10	3.0836	-580537.5720	8.5305	-580608.6817	31.8157
8	100.00	-925.2597	-0.0157	370.90	3.3430	-580535.9547	10.1478	-580613.0458	27.4516
9	107.23	-925.2665	-0.0156	370.30	3.3484	-580540.0480	6.0545	-580617.2632	23.2343
10	114.46	-925.2753	-0.0154	364.40	3.4024	-580544.2388	1.8637	-580622.6999	17.7976
11	121.68	-925.2841	-0.0153	355.90	3.4834	-580547.8067	-1.7042	-580628.1361	12.3614
12	128.91	-925.2926	-0.0151	346.70	3.5764	-580550.9044	-4.8019	-580633.3782	7.1192
13	136.14	-925.3004	-0.0150	338.50	3.6630	-580553.7292	-7.6267	-580638.1987	2.2987
14	143.37	-925.3074	-0.0149	331.40	3.7414	-580556.2221	-10.1196	-580642.4991	-2.0017
15	150.60	-925.3132	-0.0148	326.10	3.8015	-580558.4434	-12.3409	-580646.1071	-5.6096
16	157.83	-925.3179	-0.0147	325.80	3.8059	-580561.2328	-15.1303	-580648.9980	-8.5005
17	165.05	-925.3211	-0.0147	328.60	3.7735	-580563.9825	-17.8800	-580651.0005	-10.5030
18	172.28	-925.3231	-0.0147	334.50	3.7061	-580566.7967	-20.6942	-580652.2607	-11.7633
19	179.51	-925.3237	-0.0148	337.70	3.6714	-580568.0154	-21.9129	-580652.6799	-12.1825

Table S13. Calculated data for the construction of the potential energy curve of the isomerization of **11** from *cis* (small angle) to *trans* (large angle). Values stated with 'rel.' are referenced to the *cis* isomer energy of the respective state.

Step	Torsion Angle [°]	$E_{S(SCF)}$ [eh]	E_{disp} [eh]	λ [nm]	$E_{1(ecx)}$ [eV]	E_{S1} [kcal/mol]	rel. E_{S1} [kcal/mol]	E_{S0} [kcal/mol]	rel. E_{S0} [kcal/mol]
1	-11.22	-1613.3832	-0.0209	446.10	2.7790	-1012351.8685	0.0000	-1012415.9527	0.0000
2	-26.47	-1613.3797	-0.0207	507.90	2.4409	-1012357.3055	-5.4370	-1012413.5930	2.3597
3	-41.71	-1613.3718	-0.0203	625.90	1.9807	-1012362.7313	-10.8629	-1012408.4080	7.5447
4	-56.96	-1613.3591	-0.0200	870.60	1.4242	-1012367.3734	-15.5049	-1012400.2156	15.7371
5	-72.21	-1613.3426	-0.0197	1489.70	0.8323	-1012370.5107	-18.6423	-1012389.7038	26.2489
6	-80.00	-1613.3337	-0.0195	2327.20	0.5328	-1012371.7119	-19.8434	-1012383.9977	31.9550
7	-87.46	-1613.3190	-0.0199	21051.30	0.0589	-1012373.6206	-21.7522	-1012374.9788	40.9739
8	-96.49	-1613.3274	-0.0198	2562.30	0.4839	-1012369.0349	-17.1664	-1012380.1933	35.7594
9	-102.70	-1613.3358	-0.0196	1757.40	0.7055	-1012369.0797	-17.2112	-1012385.3484	30.6043
10	-112.98	-1613.3500	-0.0194	1165.40	1.0638	-1012369.6439	-17.7754	-1012394.1765	21.7762
11	-117.95	-1613.3568	-0.0193	1001.70	1.2378	-1012369.7896	-17.9211	-1012398.3326	17.6201
12	-129.47	-1613.3716	-0.0190	752.90	1.6467	-1012369.4619	-17.5934	-1012407.4346	8.5181
13	-133.20	-1613.3758	-0.0190	697.00	1.7788	-1012369.0723	-17.2039	-1012410.0915	5.8613
14	-145.96	-1613.3885	-0.0187	565.50	2.1926	-1012367.3545	-15.4860	-1012417.9168	-1.9640
15	-148.45	-1613.3906	-0.0188	546.00	2.2707	-1012366.8430	-14.9746	-1012419.2067	-3.2540
16	-162.45	-1613.3994	-0.0187	476.10	2.6041	-1012364.6114	-12.7429	-1012424.6628	-8.7101
17	-163.69	-1613.3999	-0.0187	471.80	2.6277	-1012364.4367	-12.5683	-1012425.0316	-9.0789
18	-178.94	-1613.4031	-0.0185	446.70	2.7756	-1012362.9224	-11.0539	-1012426.9287	-10.9760

Table S14. Calculated data for the construction of the potential energy curve of the isomerization of **11** from *cis* (small angle) to *trans* (large angle). Values stated with 'rel.' are referenced to the *cis* isomer energy of the respective state.

Step	Torsion Angle [°]	$E_{S(SCF)}$ [eh]	E_{disp} [eh]	λ [nm]	$E_{2(ecx)}$ [eV]	E_{S2} [kcal/mol]	rel. E_{S2} [kcal/mol]	E_{S0} [kcal/mol]	rel. E_{S0} [kcal/mol]
1	-11.22	-1613.3832	-0.0209	277.70	4.465	-1012312.9892	0.0000	-1012415.9527	0.0000
2	-26.47	-1613.3797	-0.0207	287.70	4.3097	-1012314.2101	-1.2209	-1012413.5930	2.3597
3	-41.71	-1613.3718	-0.0203	301.00	4.1196	-1012313.4088	-0.4196	-1012408.4080	7.5447
4	-56.96	-1613.3591	-0.0200	317.50	3.9048	-1012310.1687	2.8205	-1012400.2156	15.7371
5	-72.21	-1613.3426	-0.0197	336.40	3.6859	-1012304.7064	8.2828	-1012389.7038	26.2489
6	-80.00	-1613.3337	-0.0195	346.20	3.5812	-1012301.4132	11.5760	-1012383.9977	31.9550
7	-87.46	-1613.3190	-0.0199	367.60	3.3732	-1012297.1910	15.7982	-1012374.9788	40.9739
8	-96.49	-1613.3274	-0.0198	350.20	3.5407	-1012298.5436	14.4456	-1012380.1933	35.7594
9	-102.70	-1613.3358	-0.0196	339.40	3.6527	-1012301.1168	11.8724	-1012385.3484	30.6043
10	-112.98	-1613.3500	-0.0194	325.90	3.8044	-1012306.4447	6.5444	-1012394.1765	21.7762
11	-117.95	-1613.3568	-0.0193	321.40	3.858	-1012309.3656	3.6236	-1012398.3326	17.6201
12	-129.47	-1613.3716	-0.0190	312.10	3.9722	-1012315.8336	-2.8444	-1012407.4346	8.5181
13	-133.20	-1613.3758	-0.0190	309.50	4.0054	-1012317.7263	-4.7371	-1012410.0915	5.8613
14	-145.96	-1613.3885	-0.0187	304.60	4.0704	-1012324.0525	-11.0633	-1012417.9168	-1.9640
15	-148.45	-1613.3906	-0.0188	303.50	4.0847	-1012325.0122	-12.0230	-1012419.2067	-3.2540
16	-162.45	-1613.3994	-0.0187	307.60	4.0304	-1012331.7216	-18.7324	-1012424.6628	-8.7101
17	-163.69	-1613.3999	-0.0187	308.50	4.0185	-1012332.3633	-19.3742	-1012425.0316	-9.0789
18	-178.94	-1613.4031	-0.0185	320.10	3.8735	-1012337.6053	-24.6161	-1012426.9287	-10.9760

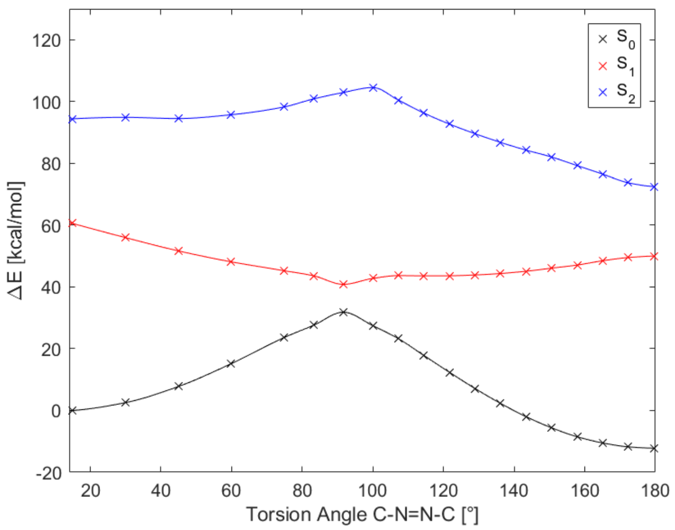

Figure S71. PES of the isomerization of **10c** for the ground state and the first two excited states referenced to the ground state energy of the *cis* isomer.

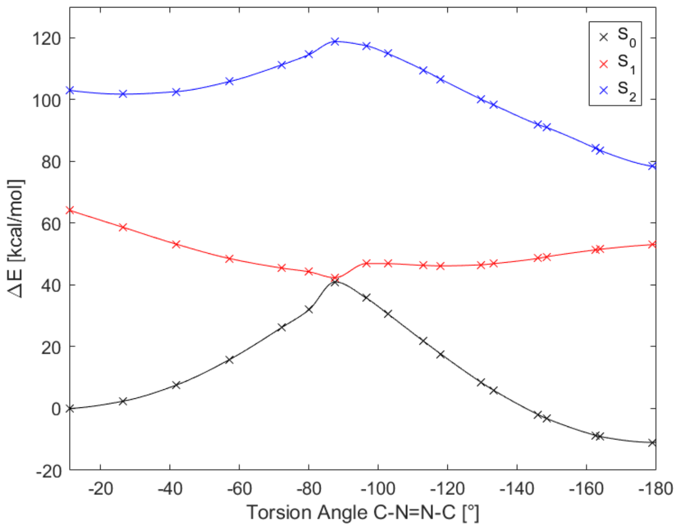

Figure S72. PES of the isomerization of **11** for the ground state and the first two excited states referenced to the ground state energy of the *cis* isomer.

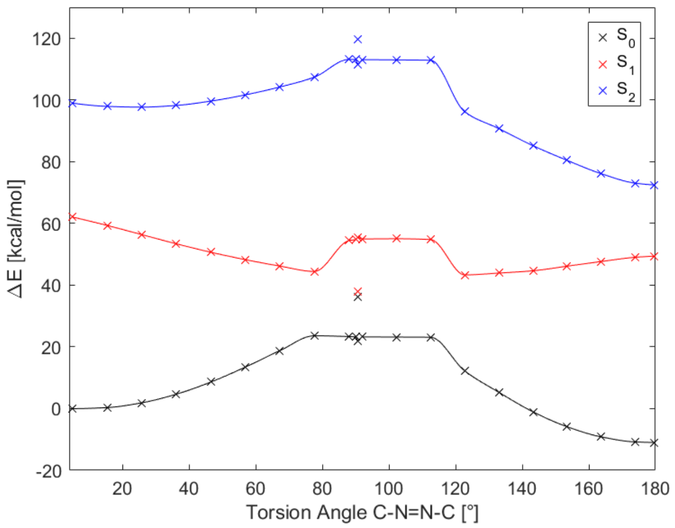

Figure S73. PES of the isomerization of **10⁺** for the ground state and the first two excited states referenced to the ground state energy of the *cis* isomer. The data points not connected with the line correspond to those of the structure obtained from the CI search calculation.

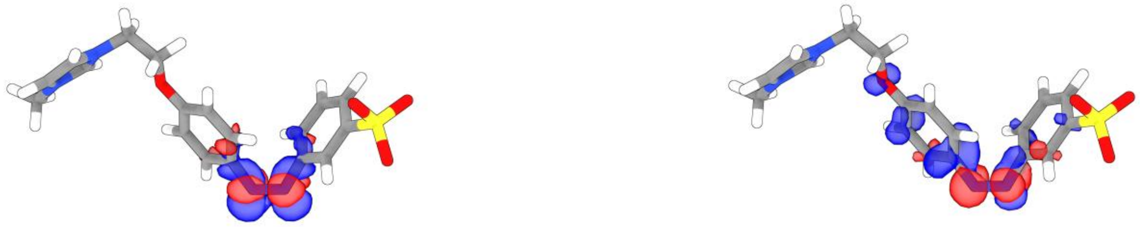

Figure S74. Electron (red) and hole (blue) functions of the first (left) and second (right) excited state of *cis*-**11**. Surface isovalues: electron and hole: 0.003.

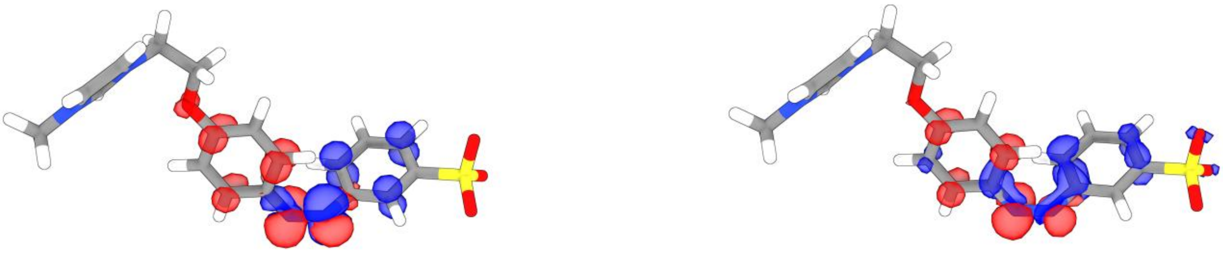

Figure S75. Electron (red) and hole (blue) functions of the first (left) and second (right) excited state of the transition states of the isomerization of **11**. Surface isovalues: electron and hole: 0.003.

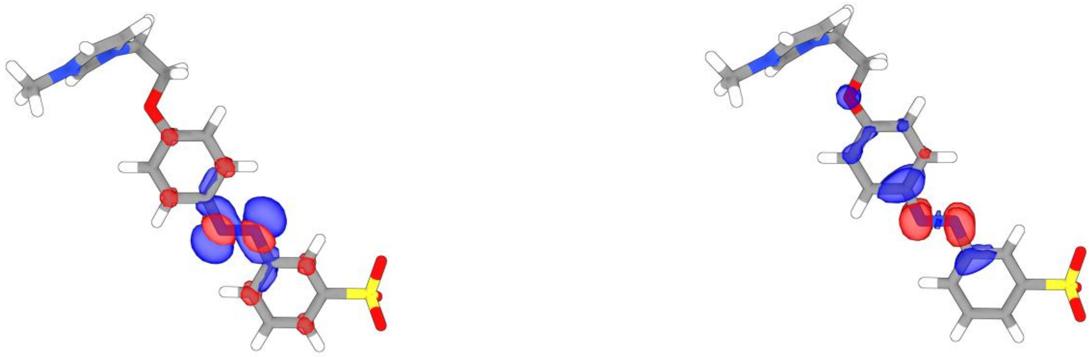

Figure S76. Electron (red) and hole (blue) functions of the first (left) and second (right) excited state of *trans*-**11**. Surface isovalues: electron and hole: 0.003.

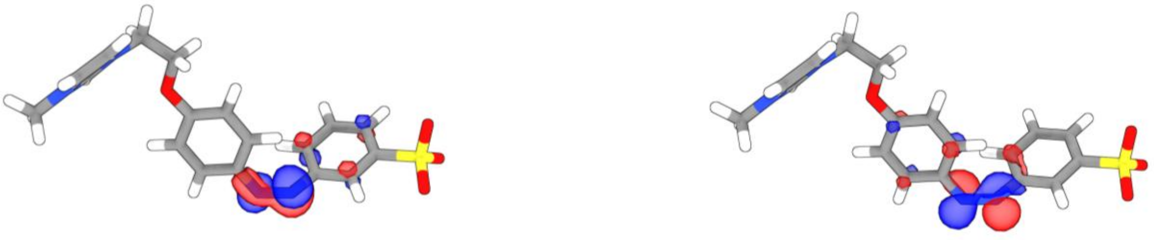

Figure S77. Fock orbitals of the active space of the transition state of the isomerization of **11**, obtained from the CAS-SCF calculation. Left: Orbital 100, occupation 1, right Orbital 101, occupation 1. Surface isovalues = 0.03.

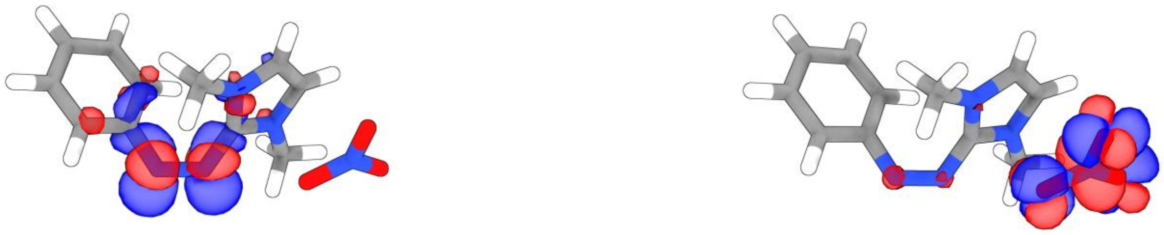

Figure S78. Electron (red) and hole (blue) functions of the first (left) and second (right) excited state of *cis*-**10c**. Surface isovalues: electron and hole: 0.003.

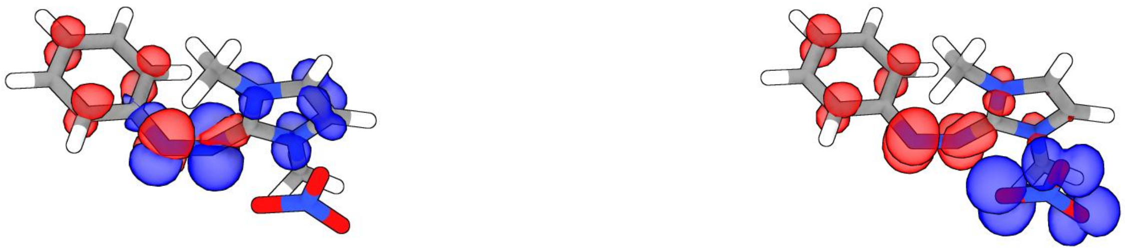

Figure S79. Electron (red) and hole (blue) functions of the first (left) and second (right) excited state of step 6 of the isomerization of **10c**. Surface isovalues: electron and hole: 0.003.

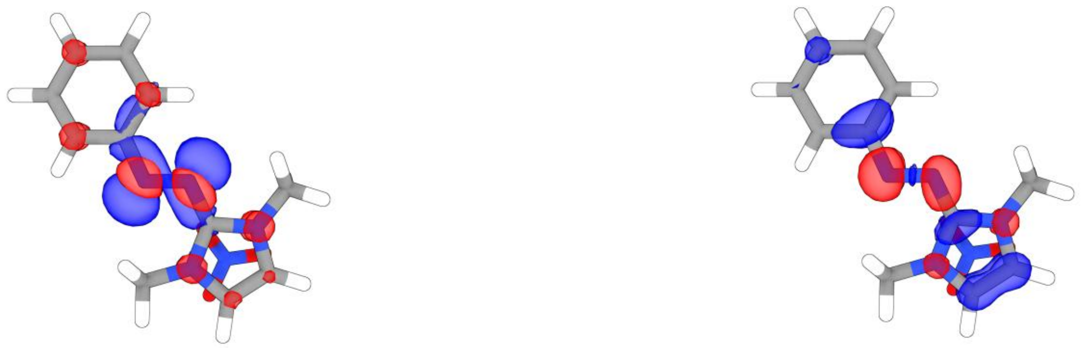

Figure S80. Electron (red) and hole (blue) functions of the first (left) and second (right) excited state of *cis*-**10c**. Surface misvalues: electron and hole: 0.003.

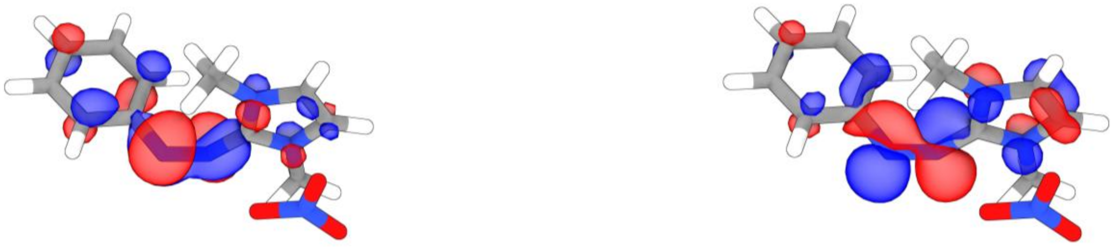

Figure S81. Fock orbitals of the active space of the transition state of the isomerization of **10c**, obtained from the CAS-SCF calculation. Left: Orbital 68, occupation 1, right Orbital 68, occupation 1. Surface isovalues = 0.03.

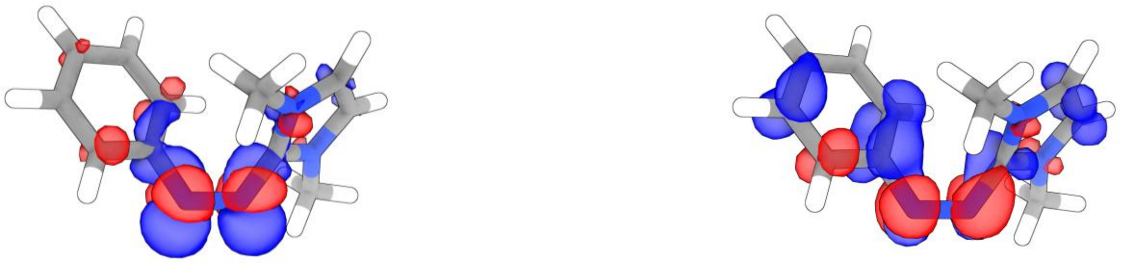

Figure S82. Electron (red) and hole (blue) functions of the first (left) and second (right) excited state of *cis*-**10⁺**. Surface isovalues: electron and hole: 0.003.

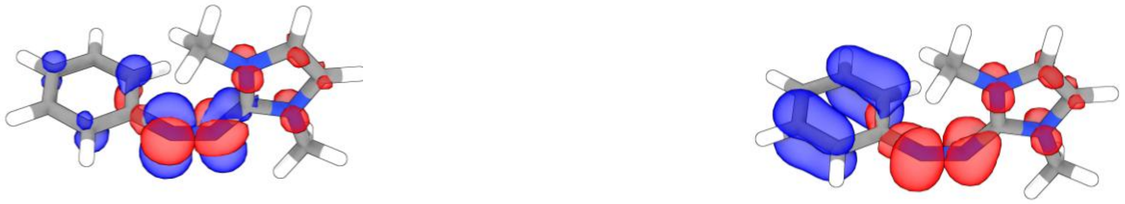

Figure S83. Electron (red) and hole (blue) functions of the first (left) and second (right) excited state of step 11 of the isomerization of **10⁺**. Surface isovalues: electron and hole: 0.003.

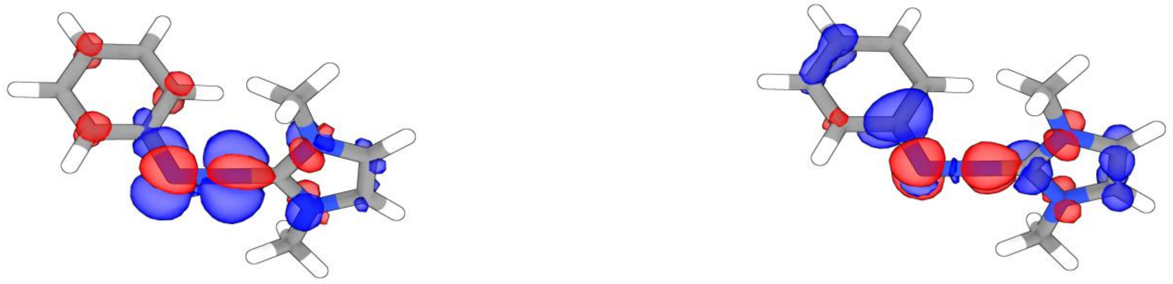

Figure S84. Electron (red) and hole (blue) functions of the first (left) and second (right) excited state of step 11* of the isomerization of **10⁺**. Surface isovalues: electron and hole: 0.003.

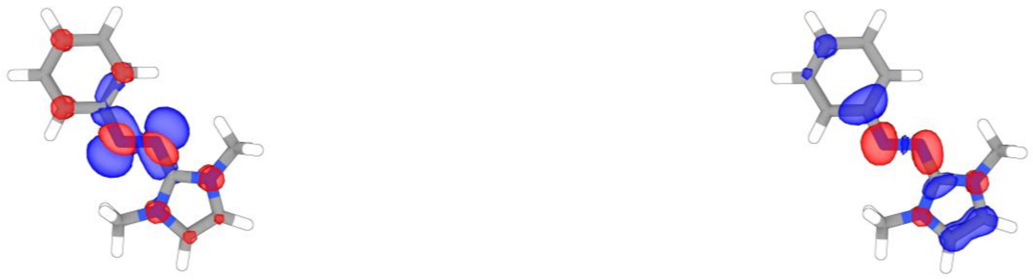

Figure S85. Electron (red) and hole (blue) functions of the first (left) and second (right) excited state of *trans*-**10⁺**. Surface isovalues: electron and hole: 0.003.

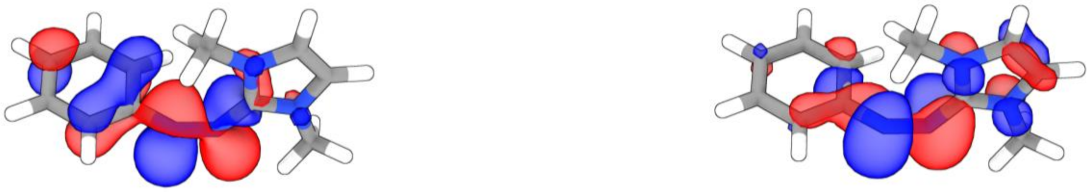

Figure S86. Fock orbitals of the active space of the transition state (step 11) of the isomerization of **10**$^+$, obtained from the CAS-SCF calculation. Left: Orbital 52, occupation 1, right Orbital 53, occupation 1. Surface isovalues = 0.03.

5.3 Cartesian coordinates of optimised structures

Cis-**10⁺**

C	-3.98094218808896	1.92230876260260	-0.64703164905909
C	-3.91652439917005	0.62969543449617	-0.20345381570305
N	-2.76304918697920	2.50539794458912	-0.37940895417147
N	-2.65040727194906	0.42477441085289	0.30451361656414
C	-1.94399929268356	1.57802065140323	0.16978975010989
N	-0.72722625974823	1.93809141228688	0.74663253228573
C	-2.36096918734619	3.87240019633305	-0.70466109027812
H	-3.26064698954560	4.49318311206549	-0.74465297581347
H	-1.84657010624300	3.89007637559892	-1.67310580446114
H	-1.68486747876946	4.23276048104268	0.07871043610891
H	-4.78502360720405	2.47219964903760	-1.12046300955875
H	-4.66597713182779	-0.15213809728080	-0.18106732781735
N	0.32490433838131	1.24213230464016	0.66948369537053
C	0.47731244085790	0.18083647812112	-0.24736517996553
C	1.32185003478153	-0.86767710697200	0.15966115776686
C	1.54825283050079	-1.94074471825974	-0.69710426879844
C	0.98986789910838	-1.93960245929673	-1.98034374689094
C	0.20279970052609	-0.86140347374016	-2.40789751653104
C	-0.06773019932524	0.19670469573058	-1.54725397639876
H	-0.64990824297902	1.04902922324554	-1.89717820718565
H	1.18606948620266	-2.76946255169957	-2.65996259962643
H	-0.19415521425685	-0.84365571225980	-3.42317118069858
H	1.76001174094989	-0.83343487470507	1.15737706266236
H	2.17466818383495	-2.77161305275066	-0.37209887256486
C	-2.18967263420465	-0.81468228332483	0.92701812108720
H	-3.06852751766603	-1.35733221453148	1.28715940751722
H	-1.53415961155614	-0.57994834899621	1.77200685021973
H	-1.64769013560039	-1.42329623822898	0.19238754583013

Trans-**10⁺**

C	-3.91244180658670	1.78801494760303	-0.01928132995410

C	-3.48692781111644	0.48040813332033	-0.03228614485106
N	-2.79619342244397	2.57647087988572	-0.01431538803830
N	-2.11674752593988	0.48595367699288	-0.03520367168958
C	-1.68973864136745	1.78353461656484	-0.02419993798059
N	-0.45269485981109	2.35456636950243	-0.02085388668989
C	-2.77423690817082	4.04110229010818	-0.00003097732873
H	-3.81094966271773	4.38807180148938	0.00531702131600
H	-2.25819728656802	4.40845210630213	-0.89331740421270
H	-2.25478298385050	4.39078072579654	0.89836379065540
H	-4.91165487295150	2.20587771778070	-0.01336283379524
H	-4.05139045734261	-0.44449709728273	-0.03948625031705
N	0.53507164339360	1.54184400141301	-0.03256859184463
C	1.79747598739907	2.14830348547726	-0.02776792845608
C	2.02304693711604	3.54202715554108	-0.01048189221553
C	3.32652814890409	4.01926715863658	-0.00700343732759
C	4.41097709943401	3.12579525674749	-0.02056783057859
C	4.18919021457676	1.74487268435301	-0.03770844586493
C	2.88577443036845	1.25446171693560	-0.04132687041189
H	2.68095466410811	0.18336282027833	-0.05449220142653
H	5.43064001360099	3.51314749956635	-0.01763653702282
H	5.03241048097384	1.05390873819401	-0.04816007149838
H	1.17421864052988	4.22434564686561	0.00003820034553
H	3.51069765699454	5.09423752080321	0.00639808034718
C	-1.28533560207536	-0.72000475312754	-0.04754341588930
H	-1.96046963029784	-1.58090507467672	-0.05223501439526
H	-0.64823100993408	-0.73720906896157	0.84244433137339
H	-0.65390343622538	-0.72297095610919	-0.94172136224872

Cis-**10b**

C	-3.84931125419670	2.04200274253476	-1.43014934400687
C	-4.23898368730798	1.06214533340920	-0.55651885789700
N	-2.74010486950971	2.64411596318372	-0.89232469329992
N	-3.34468582810581	1.04975042670421	0.48751711529235
C	-2.41738062683802	2.02211903625080	0.26811704584785

N	-1.45255607664651	2.57363727158373	1.10172217716257
C	-1.98908252731963	3.75292291777718	-1.47190033990791
H	-2.64539644416575	4.27825241034145	-2.17142766312504
H	-1.10303581475076	3.37554319981951	-1.99641974537235
H	-1.68097802306927	4.42400572527837	-0.66255535566936
H	-4.27373006300749	2.36274889146927	-2.37337133859566
H	-5.09479064836375	0.39972065203549	-0.57324314051559
N	-0.66340264361735	1.91356072230344	1.83303745059333
C	-0.46003882150181	0.52421633025499	1.68654624633126
C	-0.18663228841198	-0.19530655774176	2.86230673408589
C	0.05959969784854	-1.56378593424992	2.78987980493256
C	0.09879243822905	-2.20302186595849	1.54532956013589
C	-0.10945839315934	-1.46945952117561	0.36984225703516
C	-0.39858803408282	-0.10968321098223	0.43073856567965
H	-0.53272324316428	0.46432035894738	-0.48625470301430
H	0.31287985039600	-3.27068371390631	1.48784044112819
H	-0.03965012985471	-1.96154838175395	-0.60070124567834
H	-0.20599590255442	0.32919859690074	3.81763108341662
H	0.23648747277396	-2.13153795138135	3.70365870397667
C	-3.48410766093808	0.22913047031048	1.68659463932010
H	-4.51148472978869	-0.14263861766936	1.70829221044738
H	-3.31659415253647	0.87822419220420	2.55785018351422
H	-2.77615811285098	-0.60724060586464	1.65956928933268
H	-9.47360108409562	4.28058174128660	9.12853414203652
H	-7.48537514098182	5.09738770299246	7.84635130453382
C	-8.64646604163065	3.60446596182919	8.91076689969378
C	-7.54442740983016	4.06647986689224	8.19641959819071
H	-7.21841536407337	3.31323408143307	4.91682954779735
H	-8.08210678811030	1.86879153439035	4.31406359940303
C	-7.49123697287603	2.27213372483007	5.14090102596357
H	-8.05445950057259	2.19710883920035	6.07861835345762
C	-8.68156561650580	2.28080763801310	9.36403670350950
C	-6.50078755796124	3.18012394681978	7.87851604849826
N	-5.39807354074571	3.73081393049581	7.18942542847378

H	-9.53540108191135	1.92513680475865	9.94155710696934
N	-4.70654846218402	3.07206713582834	6.36193800089316
N	-6.26520792851210	1.48270327870016	5.22678922604325
C	-7.60969339840074	1.41720745052841	9.10145781672381
C	-6.52143472993315	1.85374059904341	8.35277747001281
C	-5.09433595967533	1.85628021485912	5.81041099401553
C	-6.07383628864493	0.26373432557650	4.61841636807781
H	-6.86499410145096	-0.21564025165395	4.05615409294136
H	-7.62132618854264	0.39831580771106	9.48948732218403
H	-5.67702996965877	1.18849797645495	8.17283124181600
N	-4.17735669883456	0.89641365600426	5.55074876113545
C	-4.77575395777217	-0.11224543370914	4.83905508366078
H	-2.37866254125820	1.93879060270654	5.58649053021478
C	-2.77198315092656	0.97587184367711	5.93195975247665
H	-4.23243353288158	-1.00018012234867	4.54126360337870
H	-2.66662571706082	0.89081593396300	7.01954007023437
H	-2.24296331358311	0.15597799309041	5.43864689243230
W	-5.06150212741016	3.62027308906689	2.46658148559115
O	-6.15717296268378	2.32244051056056	1.86227456399001
O	-5.94980043217054	4.65212919587997	3.65638774687479
O	-4.50117255742790	4.63200727546230	1.08127092824113
O	-3.63551936519711	2.86292426503129	3.27484120938353

Trans-**10b**

C	-4.32564827982185	2.20210223570305	-0.26078942618291
C	-4.19867497962355	1.23849303413310	-1.23182165602778
N	-3.17584284546129	2.18692981492675	0.47910846908902
N	-2.98221127939079	0.63406950214330	-1.06320038482334
C	-2.35689957255782	1.21233931294546	0.00181927886771
N	-1.09935997973935	1.05186519961154	0.50195576130676
C	-2.90368592409144	3.00677697581124	1.65611605461139
H	-3.39649440777650	3.97461797125297	1.52251311230144
H	-1.82192859901878	3.13747372219723	1.75090980748633
H	-3.29598601673690	2.50696189748810	2.55024259708502

H	-5.13972878338697	2.88146255171387	-0.04140660597287
H	-4.87933080482575	0.92655898889853	-2.01174313763090
N	-0.53292819438492	-0.05698752686217	0.21401868116384
C	0.81468672819451	-0.14367021119533	0.58040391806098
C	1.62853099017815	0.96941073253674	0.87011245073256
C	2.97116192835781	0.77187801121524	1.16657555596034
C	3.50689478847453	-0.52390687117415	1.19331544359452
C	2.69824062689617	-1.62993669821947	0.90787906839007
C	1.35799802186294	-1.44177931519903	0.58522481594632
H	0.70462044404040	-2.28207735345799	0.34815172347069
H	4.56184236336427	-0.66869657583217	1.42972682109440
H	3.11769656520560	-2.63596277360461	0.92997168666017
H	1.19960098246601	1.96993388347137	0.83378478902544
H	3.61133511383677	1.62850863056883	1.38047694857446
C	-2.46298462943563	-0.43707666814451	-1.91400702794193
H	-3.09466130135170	-0.47999239014406	-2.80557930680735
H	-2.50755582206848	-1.38493951255205	-1.36191462125798
H	-1.42694679735064	-0.21081283914546	-2.18761970092724
W	-4.81120892343701	-1.94681611350753	0.67175673590912
O	-4.77723389797211	-0.50328079653645	1.76067208219692
O	-5.39897207605472	-1.45640045664748	-0.96036233474477
O	-5.94012172387507	-3.17982841523902	1.34052633267139
H	-4.96735766251285	-1.33508041898282	5.58931858030173
H	-6.52646237359865	-1.98967833544534	4.98875812450164
C	-5.49182459960743	-1.75443186849178	4.72422966960841
H	-6.47144305327432	-4.38534588663446	4.47065908751438
C	-5.43910001341283	-4.21892851598437	4.19061948817046
N	-4.84552545799347	-2.99823509087285	4.30757799894553
H	-5.45268116735056	-1.05483267385424	3.87573133786498
C	-3.56460509601023	-3.08072393317937	3.85649794325236
C	-4.50678669775931	-5.07670350470966	3.65514278609345
N	-2.74733990423023	-1.99530624689253	3.95667217804072
H	-2.27875036577861	0.43757545791964	3.68533239768797
C	-1.21143855795247	0.26763324581007	3.82163732800181

N	-3.35586649986682	-4.36680294083582	3.44906825805582
H	-0.69750827590200	2.34918965462831	3.99294474428548
H	-4.58384731001208	-6.12362532444058	3.38756217925667
C	-0.32611328126314	1.32403146688902	3.99086798500381
N	-1.51797170696141	-2.19047456458611	3.66694498495391
C	-0.71394146738172	-1.05018179989184	3.79676143870628
H	-1.30210546270768	-4.83736108627653	3.46112568965838
C	1.04613012197495	1.07996379498614	4.14972136853270
C	-2.16158392600202	-4.89122635826660	2.78672020309404
C	0.66805990464807	-1.29118808904227	3.90966296857599
H	1.73267095608628	1.91683918964818	4.28225161647070
C	1.54107283321366	-0.22737851994823	4.11147284896893
H	-2.37105872929639	-5.92928944005044	2.51049024073774
H	1.02590960880311	-2.31978912270218	3.86168196201300
H	2.60979373206059	-0.41167924052361	4.21545636454065
H	-1.97839491892271	-4.28342027265552	1.89145580182931
O	-3.14969434350494	-2.64959752276983	0.51859649345056

Cis-**10c**

C	-4.01378415154845	2.04742289440980	-0.92531803318457
C	-3.97279098451393	0.76858832195413	-0.43965702813563
N	-2.80510670137364	2.63148824458930	-0.63161283944793
N	-2.72839055636574	0.57293750340187	0.11773709967225
C	-2.01265149360341	1.71976974860458	-0.02087275546963
N	-0.82102890214405	2.09968482599726	0.58674793394575
C	-2.40223911278492	3.99951531503219	-0.94375224053657
H	-3.30655864722344	4.60607856946380	-1.04658985342515
H	-1.82607295513550	4.02063749735708	-1.87666750206067
H	-1.78523070751436	4.37664592025581	-0.12042681336125
H	-4.79823224290833	2.58669537596187	-1.44102524893824
H	-4.73008101301426	-0.00513326656905	-0.41521811624446
N	0.22255387783438	1.38673712120085	0.61487068821091
C	0.40889794536955	0.26188230338513	-0.21735630819695
C	1.21307332704786	-0.76570691413799	0.30682231857431

C	1.47557905147489	-1.89545305153844	-0.46281677559123
C	0.99398360910579	-1.97456662520968	-1.77430651452024
C	0.24755530937189	-0.91964483539794	-2.31722890772299
C	-0.05870350654798	0.19578610680121	-1.54522549263003
H	-0.61244994750640	1.02673522506980	-1.98204789354469
H	1.21822626122931	-2.84980576135252	-2.38481759743744
H	-0.09046301605843	-0.96577970400451	-3.35279870062290
H	1.59302950536432	-0.67036323279529	1.32426737974492
H	2.07029305086864	-2.70900384066164	-0.04695879421821
C	-2.32213834580822	-0.62683221084170	0.84312328932004
H	-3.22864112114303	-1.13485175462169	1.18453407021689
H	-1.71638406638714	-0.34251401767855	1.70949707517084
H	-1.74549207373646	-1.28996782081442	0.18628310523022
N	-4.82157163211324	2.57767211562932	2.24866743358632
O	-5.67258275028534	1.66855557232756	2.48179323487551
O	-3.59685338408101	2.38458244029141	2.51468673040985
O	-5.19345462586917	3.68113793389036	1.74779705633100

Trans-**10c**

C	-3.88079315109880	1.72787372593523	0.01760973219336
C	-3.46263528264220	0.42821005053031	-0.14494064061142
N	-2.76044225970894	2.50946690556558	0.06351536190163
N	-2.09290968538750	0.43043505150854	-0.19312651570299
C	-1.65903610505697	1.71901136236819	-0.06253480843316
N	-0.42012907854100	2.28626546250309	-0.04682224256942
C	-2.73088811878531	3.96370408744521	0.23217717239411
H	-3.76543209138507	4.31722016113709	0.24559595853911
H	-2.18822204579876	4.41788749892932	-0.60326211111742
H	-2.23547972984668	4.21493894013670	1.17633004447888
H	-4.87674790905479	2.14541704834804	0.09968841557377
H	-4.03157514676634	-0.48978359898892	-0.23227510729945
N	0.56566713052762	1.47436323507131	-0.12190417101400
C	1.82889782235412	2.08007605862535	-0.11497311464526
C	2.05127366338538	3.47465828883677	-0.09759433591920

C	3.35333738321867	3.95537035484009	-0.09154263560755
C	4.43960233978603	3.06364384056567	-0.10101818945411
C	4.21911131950969	1.68275904638391	-0.11863113733984
C	2.91726523826029	1.18642565697270	-0.12843078409869
H	2.72408921821637	0.11072053967314	-0.13538292473779
H	5.45867182523917	3.45282596466883	-0.09586185022745
H	5.06390988481741	0.99324359758852	-0.12433608445670
H	1.20113253482561	4.15542931493883	-0.09257080271521
H	3.53512699591008	5.03082791467097	-0.08075097397478
C	-1.27153672001296	-0.77071606569550	-0.36652784729498
H	-1.95425744317683	-1.61438641931453	-0.50383556129336
H	-0.64032702564940	-0.92851069029222	0.51551205685805
H	-0.63082820257291	-0.65131510918914	-1.24576002009022
N	1.66618972453781	-2.40105439709882	0.59254939162028
O	1.33048449456154	-1.72669589489419	1.61380499895802
O	2.78954126693575	-2.19573520456363	0.04015858886769
O	0.87937915339892	-3.27372672720655	0.12279013721811

Cis-**11** gas phase BP86

S	-0.26695973119826	4.20266781024819	26.41084750951100
C	1.21815349089583	3.65224718220173	23.29110666047973
H	1.14167700290741	3.94909292006180	24.34521709235081
O	4.49801647216204	3.17345098680268	23.73604952673050
O	0.86946470743097	3.28908883722648	26.07922757850775
C	0.90644538794016	3.44000686234051	21.09996310426318
H	0.53566051836717	3.70344484509653	20.11751877120630
O	-0.79623065546024	4.86628108669377	25.19047340015426
C	1.64260551914724	2.36798095668942	21.52193109883646
H	2.03655355324414	1.52023533109858	20.97606267347856
O	-1.27728742698368	3.62022667573102	27.28579545113891
C	2.58358990238269	1.65433724395565	23.79076433794604
H	2.28370617551082	0.61098905725471	23.61939989865456
H	2.29250440881239	1.93989933821655	24.81256855706771
C	4.08542808611233	1.82325296417848	23.55727952935645

H	4.63376967947700	1.12461250498300	24.20910997225860
H	4.35024450496038	1.58332357824716	22.51785080471544
C	4.51193090777441	3.67816009959723	25.02791364058163
C	4.33824883617160	5.06306569017187	25.14231018104391
H	4.19192596154017	5.65101008052446	24.23568828629177
N	0.65117246062800	4.22192084233926	22.21657157345048
C	-0.12790428357634	5.46279481156053	22.25882533854782
H	0.49133719790040	6.30095977528255	21.91474816650813
H	-0.45060589409281	5.60850290541468	23.29929798131303
H	-1.00161023290730	5.35317217352567	21.60684790887007
N	1.81610276136194	2.51904590177035	22.88858058766986
N	4.56477420284006	5.45588876487266	28.86225821314513
N	3.63781032477131	6.17889358911747	29.30836725670790
C	4.33355025080667	5.66414136442826	26.39391706909596
H	4.16111316736384	6.73614067898907	26.48639495290046
C	4.52166030554746	4.88582531880428	27.54807480955055
C	4.77563483610196	3.51751921313544	27.42116980687636
H	4.95608752281875	2.92780962743365	28.32054005080079
C	4.73890395079473	2.90357687275107	26.16946688361364
H	4.89741396061790	1.82803546174518	26.10377713296990
C	2.39364694909217	6.34333784601244	28.61966464062793
C	1.79119598272425	7.60621195813378	28.71005246598815
H	2.30394989873034	8.39303851836421	29.26443440509601
C	0.57177520231434	7.83929154872068	28.07155251885547
H	0.11487381761677	8.82876374153618	28.12230768564842
C	-0.06731713161289	6.81333015900626	27.37143708465127
H	-1.01029791559132	6.98571552783200	26.85306251726818
C	0.51570341256851	5.54054567444923	27.33130676347106
C	1.72845198687570	5.29547723979458	27.96283604446751
H	2.15132996511064	4.29562643365985	27.90925006732799

Trans-**11** gas phase BP86

S	6.23796688785104	1.86346739673362	27.29215850751939
C	3.42748179886690	4.88123111846647	23.02203892553113

H	3.62453676391334	5.82985727505027	22.54074912856401
O	-0.19034029046247	6.01373137267415	22.36205457722228
O	7.63950806872721	1.52319795693230	27.52864568947119
C	3.59545591451762	3.11739334058746	24.36681355948003
H	4.11849610653162	2.48405931387271	25.09823861118514
O	5.90022775448288	2.04539440957182	25.83900126792663
C	2.47044569867835	2.96605478411696	23.59845097600489
H	1.74665269745488	2.16201358188847	23.55981544104433
O	5.22294130746056	1.08759126056754	28.00898558188255
C	1.42096981704044	4.26373873195964	21.65929363428212
H	1.40145831888665	3.32468065048713	21.08754922232551
H	1.82718260772933	5.04548599562131	21.00465454419762
C	-0.01784566290898	4.62861281417008	22.04773253704598
H	-0.65273477877268	4.45262591482467	21.16910904055096
H	-0.37689679159325	3.97703190963903	22.86074512022197
C	0.45845111739534	6.40060681501681	23.52675963357857
C	1.35318780170301	7.47032180902689	23.46778706884101
H	1.43707013106310	8.04697786417235	22.54588833212618
N	4.17252707931012	4.31743677990191	23.98271153205517
C	5.42859901860034	4.85760242417709	24.52497053918861
H	5.92639357146028	4.03602028497313	25.06142868439141
H	5.20618254306989	5.68766482043372	25.20664918759121
H	6.05220388569203	5.20620925263790	23.69412581671148
N	2.38183787567535	4.07315321204185	22.76106552378027
N	3.29267931456276	6.87500729463971	26.52105699374947
N	3.32505100549509	5.82815054074244	27.24051312639558
C	2.19522220555176	7.71225228255641	24.55589926203334
H	2.97444497090523	8.47420169666081	24.50537160836155
C	2.14435388886233	6.88000876498976	25.68298592512664
C	1.10933312834459	5.93113028120589	25.79731166683803
H	1.04579169206444	5.33120040107572	26.70340506351349
C	0.26567526655946	5.69836752088899	24.72599432111825
H	-0.50804049685884	4.93269228157649	24.79347338761714
C	4.56974500077204	5.49255799459984	27.79068315152908

C	5.65030963036891	6.35962152692464	28.05050888498388
H	5.49212384731234	7.43759479251156	28.05268219431486
C	6.90707292670889	5.80249482681036	28.26804324780783
H	7.75336453068314	6.45217671357407	28.49853010019016
C	7.11628347142468	4.41697370415275	28.13026542008206
H	8.11468719316248	3.98377998849568	28.20132225987269
C	6.03882021147667	3.57271801101482	27.85918963964966
C	4.75331216762206	4.10304733115654	27.79156280727026
H	3.89960080260849	3.45498295687742	27.59217825682214

Cis-**14** gas phase BP86

C	2.15501191505058	3.96081364710164	22.12956416854404
H	2.25675350345348	4.71070819749166	21.35620164774136
O	-0.33043985985327	5.74691678151362	22.37954127011621
C	2.56550651611698	2.65831513398472	23.89082859908301
H	3.21964688403607	2.32149985373481	24.72944775163439
C	1.29073665863510	2.40704340214175	23.45885837918687
H	0.53558454645846	1.72894772691305	23.83566660484713
C	-0.22429866128376	3.42392666333370	21.67889525732715
H	-0.77279165299214	2.47184937312091	21.67250322002400
H	-0.01220569011166	3.70597373618184	20.63945934367681
C	-1.05128574064070	4.52269093648428	22.35644583442273
H	-1.95358364333733	4.70084497127212	21.75386852358915
H	-1.37307139235025	4.21129256235125	23.35981503764278
C	0.45853485663856	6.05205150443255	23.48774367783512
C	1.40010514387237	7.06951568834676	23.28839559775727
H	1.46814776519028	7.54046581812445	22.30673868351804
N	3.07659521582721	3.62895968839403	23.04186937011000
C	4.42347055516768	4.20220105295710	23.16348211884135
H	4.84271322747939	3.78548055504437	24.10214309765646
H	4.33895274308647	5.29398882127918	23.22191080782566
H	5.02672791980820	3.91113916020654	22.29467295981391
N	1.05524840060005	3.21727190102261	22.35453106263340
N	2.79828939665772	7.53306360665564	26.68636292794580

N	3.25792031932883	6.92628513662896	27.68814450325823
C	2.19304110031227	7.49905964162590	24.34859302907061
H	2.89085518912770	8.32750541737365	24.21837867500749
C	2.10319964250532	6.89092958504533	25.61128993863898
C	1.15971502995776	5.86562631522648	25.79601382667872
H	1.06243575985713	5.39065141271293	26.77101578104959
C	0.33304262217676	5.45933224055520	24.75029846414488
H	-0.39913473842666	4.67776696457681	24.94723336290301
C	3.39256660397002	5.50883238744211	27.73455035883340
C	3.02027582034793	4.88411563809230	28.93610719569258
H	2.64082905818732	5.48513430426615	29.76165601588283
C	3.12141663952926	3.48996109426233	29.00747921632673
H	2.80556219417293	2.97711253299790	29.91929747630262
C	3.64465971228116	2.74796591438634	27.95230015894088
H	3.75337870686724	1.66427505944833	28.03292278783537
C	4.10279042782578	3.36489891566572	26.73957557214295
C	3.93826717809237	4.78081446720487	26.67133162466999
H	4.27072491625413	5.31160859876350	25.77922622230669
O	4.59455521012298	2.67056359163587	25.75722984853799

Trans-**14** gas phase BP86

C	2.83474414907432	5.02084548342262	22.28642842879189
H	2.89495995434849	6.09985205626461	22.35257577340617
O	-0.83611968986952	5.80125488155470	22.96026520838677
C	3.25218249585697	2.86683504958085	22.60591675657491
H	3.80916453215507	2.00105564246693	22.94176866947049
C	2.04263920913968	2.96923813847550	21.97613919390065
H	1.34973830171467	2.20452172253450	21.64894314442320
C	0.58884551475179	4.87861242052673	21.15708112037618
H	0.44212970470165	4.35429172665668	20.20194769965220
H	0.78832471212209	5.93518023834162	20.93819053680378
C	-0.68807244952324	4.75848361303428	22.00365719886502
H	-1.55229283932801	4.85738357315224	21.33290455792201
H	-0.73752144734248	3.75950752897561	22.47210329631530

C	0.19390539557546	5.87282567995395	23.89857953326953
C	0.83483609301338	7.10177938089941	24.07042786194920
H	0.47781943106529	7.96871287043918	23.51286808457065
N	3.73410024088798	4.15155378469391	22.78054286238054
C	4.98735567560377	4.49559260044038	23.45693286548156
H	4.90499775268419	4.24815388183763	24.52817959054822
H	5.15685075884436	5.57205746580977	23.35962332097058
H	5.81182605679610	3.94301519450964	22.99319782094055
N	1.79651882100997	4.31740153135525	21.78025791779629
N	3.61794010209936	6.17782459662449	26.31684550865512
N	4.22272362128540	5.05642241983272	26.42592590594649
C	1.92999566950889	7.19325409869855	24.93139889419885
H	2.45823019547772	8.13710701449079	25.07353849564281
C	2.41694484939930	6.05116096336557	25.58873584971351
C	1.70307571845241	4.83887452068931	25.47139148318043
H	2.05432203738456	3.97625529688238	26.03639208040163
C	0.59039936466770	4.75483227034491	24.64508387274602
H	0.03738625123381	3.81739218899402	24.56539276490157
C	5.46299320198281	5.03587534922837	27.05682987865328
C	6.05203997231941	6.13216582101263	27.72510953546604
H	5.50865265222038	7.06784837726283	27.83384123678657
C	7.34745732012974	5.94717262165323	28.21522765968803
H	7.83384232257166	6.76994980552169	28.74731897045406
C	8.04960194003874	4.75338542326126	28.04175681052507
H	9.06126500191966	4.64899469317350	28.44086363584818
C	7.49907052289638	3.59951963404768	27.34892425161377
C	6.14499114013696	3.81249949946331	26.89001736726375
H	5.62290510532862	2.98226014447135	26.40761548669683
O	8.12144063766429	2.51393079605502	27.17663886882042

Cis-**11** gas phase B3LYP

S	-0.24839059719905	4.24605319949824	26.33817556982265
C	1.23414831504164	3.90962556410883	23.18275413940568
H	1.25283812118352	4.24312564127437	24.20769504291232

O	4.43519779440131	3.20985228958892	23.63453360714217
O	-0.59852954741702	5.09491628382536	25.15252531225648
C	0.77357731658592	3.72033557486765	21.02608559366721
H	0.36771201942255	4.00869138876024	20.07227691982071
O	-1.39941795906145	3.67210125796995	27.04329046965718
C	1.43942609694956	2.59725136032619	21.40926137352197
H	1.72568719926002	1.72281563526032	20.85162445878113
O	0.83281920030402	3.26565547795011	25.98517460362695
C	2.44358998883270	1.79827319563209	23.62088733576699
H	2.09117649288905	0.78740501911956	23.40515944400537
H	2.17221005181309	2.04795538437169	24.64914620710907
C	3.94760042511595	1.90238020326938	23.37388170955690
H	4.46405307335797	1.14156119954705	23.96563768857158
H	4.18193255762425	1.71919494486914	22.32343388870480
C	4.50667980330793	3.63334209801784	24.94876620946730
C	4.37496767228699	5.00743726542087	25.15764275334838
H	4.22314040810401	5.65393373278798	24.30159633376547
N	0.65390615069934	4.52349248517572	22.14893473232088
C	-0.02758345665179	5.82126750813226	22.23250132489945
H	0.62955266130141	6.60685507000548	21.85678928553836
H	-0.28733452729816	5.98465533978144	23.27863274677686
H	-0.93607680305806	5.77668793147510	21.63311589052496
N	1.71007046517690	2.73490589917789	22.76102127342095
N	4.66964937151991	5.13907560305120	28.88612904151602
N	3.74511805206711	5.78704032053147	29.41482302190903
C	4.41735314781171	5.52412322504179	26.44363026399970
H	4.27702568236821	6.58499337794202	26.60918298715240
C	4.60244846605372	4.66665914031866	27.53291223512818
C	4.82019061868042	3.30972412849690	27.31273707995950
H	5.00514501996815	2.66133138588534	28.16080699588419
C	4.74575106512952	2.78309129174715	26.02651521345209
H	4.87902732376527	1.71871286794213	25.88700116512880
C	2.47769139501307	6.01927434989994	28.78307769102985
C	1.87422659653282	7.24176466036555	29.08723081911380

H	2.39739574533945	7.93075916873233	29.73956219525956
C	0.63119478411246	7.56007797549274	28.54401428421465
H	0.17499473868414	8.51789085398895	28.76635290931199
C	-0.02765289359459	6.65087543404837	27.72110593406410
H	-0.98624526523083	6.88935695913757	27.27762823391833
C	0.56278301288552	5.41274356205040	27.46375753478447
C	1.79732173677490	5.08080772841285	27.99527947823987
H	2.21182847914601	4.11086701670030	27.76952900153738

Trans-**11** gas phase B3LYP

S	6.18817753813410	2.08173300783996	27.17129661749139
C	3.49994132303473	4.65062637610093	22.96948540965403
H	3.72254622190241	5.52887720629565	22.39090183174115
O	0.03668438687876	5.87802046119948	22.21043214586327
O	7.03979036838660	1.32403640399052	28.09413376865216
C	3.61642368970852	3.04981570998733	24.49746417143298
H	4.03956881098248	2.48015696855395	25.32753785056382
O	6.83272147107739	2.46090188779504	25.87510610530483
C	2.50964932654088	2.82969831641755	23.73048520841060
H	1.78073508226165	2.03925485610397	23.76993282195361
O	4.80872612117748	1.55199313939493	26.94957581250473
C	1.48809889244602	3.94655578320883	21.66660174180036
H	1.39044796447920	2.94893090301812	21.23209924665290
H	1.93151488542854	4.59718416546062	20.91200127655337
C	0.09247097221010	4.46506435111104	22.02937576133095
H	-0.56435132605600	4.25879048463562	21.18307935938032
H	-0.30023317342790	3.93202980846357	22.89981608685989
C	0.62180598606333	6.34849592231330	23.37182607407544
C	1.57934603471687	7.35250474120922	23.27060983972856
H	1.77285011890069	7.80534962067544	22.30528077669457
N	4.21604722059297	4.19620039619274	23.99765705622640
C	5.45068111337391	4.82434856558320	24.50278707973773
H	5.19148387552765	5.57830838959124	25.24479647922721
H	5.96692648536898	5.28776780911407	23.66275087682988

H	6.07324920984436	4.04273076968308	24.95000991660375
N	2.45079490151895	3.83905311346927	22.77646126130303
N	3.23659276002707	7.02027921959460	26.50982781043977
N	3.22567945334635	5.99650138139971	27.23571730670398
C	2.33911921787560	7.68377433326653	24.39209067888231
H	3.16097846020864	8.38650487749272	24.31919687731448
C	2.14212554186953	7.00273397165826	25.59429327221749
C	1.05090794553660	6.13099941815798	25.72674820686161
H	0.87370798554213	5.64883985176655	26.67816821535128
C	0.28678911081341	5.81299031629806	24.61984205351433
H	-0.53118836468924	5.10860731364628	24.71050693021289
C	4.43919219428483	5.64211991012550	27.85753718136417
C	5.48251404791440	6.48404381437661	28.25715090864937
H	5.32794281639673	7.55412985853096	28.31573993990594
C	6.71892414774014	5.90965123813567	28.54635328660045
H	7.53385698914127	6.53828507434923	28.88768588031857
C	6.94787277051983	4.54487608456103	28.33174225896317
H	7.93663605167064	4.12253862783074	28.46594071479909
C	5.90201283475900	3.72717523997782	27.90577591603595
C	4.63164782708627	4.26008337402765	27.77019461147660
H	3.81038070888362	3.62855693739477	27.46238337380812

Cis-**12** gas phase B3LYP

S	8.39956162517585	2.20808398106193	29.29634899260486
C	10.20763976553270	5.36169249616190	28.88048692075409
H	9.68407139811211	4.51058106814598	29.34981336764501
O	8.18802263220830	7.40269394330309	31.63695024221890
O	9.23211169164905	0.99673584482420	29.34321129839506
C	11.11394187447369	6.75547489703886	27.41061252921797
H	11.43210460529806	7.09930554385595	26.44182867990571
O	8.85620469978893	3.27225844114958	30.26103040360752
C	11.22602989663476	7.32502691412307	28.64016847813771
H	11.65798726259513	8.26183107350100	28.94620403236436
O	8.18165070769582	2.77868924033970	27.94001833130963

C	10.47208481499264	6.68806125664346	30.97857907300934
H	11.45388315076232	6.91981174170389	31.39617821321091
H	10.11782637036054	5.75980790286267	31.42825715590129
C	9.49201130441840	7.83700816737538	31.28715685932595
H	9.84182943848270	8.36831981853144	32.17235493579970
H	9.46900244631484	8.55390235663441	30.45730100520375
C	7.48620365899434	6.63573807333776	30.72969031526801
C	6.64090803369422	5.66628439721987	31.26371874680212
H	6.56121988234966	5.57699452403603	32.33973382450588
N	10.48102849948298	5.53531656086253	27.58352436908385
C	10.15823655788865	4.58150084324831	26.51021527109512
H	9.67615727007408	5.12551866521378	25.69796621896903
H	9.47530671395259	3.82708251017837	26.91060900616177
H	11.07849089465729	4.11668773463442	26.15461658339548
N	10.65893686866498	6.43827265661636	29.54207178047153
N	5.53081649315817	4.05746262346245	28.04335027514895
N	5.28425387712789	2.85311472891940	28.20138198859198
C	5.96289604017179	4.79560429370039	30.42461179281215
H	5.33217872264094	4.03365921907973	30.85606486498506
C	6.12800229518786	4.89128382308173	29.03748071071067
C	6.88342760782443	5.94146768648755	28.51221720818549
H	6.93797366373108	6.03962213027889	27.43494501396991
C	7.57097030244460	6.81100907487418	29.34788130474953
H	8.16995473514102	7.60650601505452	28.92204149130291
C	5.53300378238876	2.09883352889755	29.40241107264465
C	6.77333373420297	1.69698504912918	29.91873222954093
C	6.81103085232715	0.84079015424082	31.02246548670731
H	7.77947041809466	0.52127542229163	31.38657845406835
C	5.64483073001015	0.39882728241136	31.63175166180457
C	4.40918232917030	0.78348898970953	31.10859265738789
C	4.35976169619720	1.59802595812656	29.98795311333645
H	3.41140931385868	1.87181637566783	29.54009959438922
H	3.48782084572842	0.42990369287155	31.55730014282444
H	5.69824049633816	-0.25889670088950	32.49126430247063

Trans-**12** gas phase B3LYP

S	5.88869613541501	3.05440577801703	26.73749813228874
C	2.88646247903769	5.25723285872451	22.50901241562216
H	2.44758601138185	6.22858156503155	22.36472638210265
O	-0.49177855033767	4.53963450736306	23.04084301335502
O	6.13848395180368	3.66776249247336	25.38719495314552
C	4.19190378062272	3.62431483827114	23.24391422793150
H	4.99543916003849	3.21823745381735	23.85344036618206
O	4.51336660743406	2.51711474323458	26.87059917980783
C	3.21558226422859	3.07087111502924	22.47149609705088
H	3.01690807013298	2.04309770519080	22.22324273663937
O	6.95801586262125	2.14178956081518	27.17320667588297
C	1.14264185290914	3.95953922860423	21.28662472291251
H	1.29978361016970	3.22365084639734	20.49461576508250
H	0.91986849998346	4.91933064406988	20.81873261854739
C	-0.04167270153851	3.52373092767206	22.15716144625385
H	-0.88425914583651	3.32874935820651	21.49155166284698
H	0.18542860466916	2.59283246318121	22.68402036462252
C	0.29740423923939	4.84142420883226	24.13989243013507
C	0.29650620725343	6.17023295334147	24.56607950999916
H	-0.34892978450863	6.88201085251698	24.06540258889598
N	3.96502206339945	4.98949429784436	23.24409831188196
C	4.80459967057312	5.98198082280842	23.93484345436093
H	5.35308615602930	5.44640790614030	24.71106811301754
H	4.15872168808950	6.74045100578263	24.37294353339209
H	5.49410886813468	6.42959337467659	23.21772679427622
N	2.40828569885414	4.10520634516497	22.01721361408879
N	2.99474923450746	6.06421709429987	27.11606164010235
N	4.02373819843359	5.35864051621722	26.99906749502886
C	1.14212788588136	6.56294532538815	25.60194390880531
H	1.16446306209349	7.59337445827168	25.93760783462318
C	2.02109916733539	5.63825226492282	26.16661502535677
C	1.95560497705886	4.28821028194770	25.79263561922167

H	2.63262810189052	3.57790662348853	26.25923542468856
C	1.07103579879930	3.88862201675904	24.80446068753049
H	1.03173856815858	2.84230114090513	24.53278246227825
C	5.07836668524804	5.51938061623112	27.90854680412462
C	6.03159636612317	4.48511981024903	27.88386800587746
C	7.11917457900441	4.53909481835237	28.74884276099684
H	7.83552238774711	3.72785335404952	28.72720703099285
C	7.26573101791375	5.62108789590889	29.61561614768505
C	6.31987386430947	6.65055156714666	29.63577695488219
C	5.22068027126355	6.59938334774265	28.79017461657147
H	4.46732369093754	7.37737694002840	28.78938728668179
H	6.44282191524101	7.48753922721619	30.31396217929540
H	8.12153292825337	5.66250484766731	30.28024900493051

Cis-**14** gas phase B3LYP

C	2.14873167087928	4.19678300500939	22.07727900648762
H	2.28114566661188	5.03335386939289	21.41452975136420
O	-0.25089521793282	6.04019716670789	22.54549651716273
C	2.49920439515546	2.67166399844030	23.65010618063929
H	3.10878526758034	2.20664830131136	24.43546776100747
C	1.22334248518692	2.52586312827267	23.19453679917954
H	0.44710378680676	1.83872157623959	23.48262757427239
C	-0.25032556644976	3.82411176894964	21.57192199403072
H	-0.83492316458303	2.90945455959256	21.45531724252841
H	-0.03288604405743	4.22042990321246	20.57937074266261
C	-1.02365550513148	4.86375408500392	22.38827759221897
H	-1.91816573649076	5.14822298267107	21.82966216146835
H	-1.34359552853744	4.44916763603953	23.34490098444743
C	0.52980076220803	6.22341589532359	23.67966742405898
C	1.47970408076548	7.24202862595587	23.58793565657427
H	1.55223325146877	7.81093834311446	22.66833329628225
N	3.05160906511084	3.72081845478773	22.93321767082765
C	4.42297631035773	4.22834219185872	23.11456636195877
H	4.37665431417958	5.30423577352045	23.28210171527276

H	5.01213550435871	4.00359865611059	22.22480166271166
H	4.82251748789086	3.71378199399779	23.99913333361365
N	1.02307781867875	3.47987444968287	22.20490612962407
N	2.92062368553038	7.31810318047722	26.99062708647927
N	3.40046164053392	6.60489275256562	27.89355750048920
C	2.27611425621785	7.54470209033861	24.68462375062388
H	2.98133690210443	8.36666827541008	24.64282578922802
C	2.17787073797849	6.80827282277025	25.86961154630372
C	1.22294309510298	5.78957981456728	25.95034209830452
H	1.11696291245140	5.21723888480865	26.86177161950846
C	0.39532246940294	5.50498132270576	24.86788235373674
H	-0.33728634537264	4.71799607146250	24.98047588942567
C	3.44520617196003	5.17683834704362	27.80754715341991
C	2.97614944783458	4.46270535086655	28.91389206716955
H	2.60195650069167	4.99058226904704	29.78171680222773
C	2.98857265662755	3.06674309446392	28.83698017899497
H	2.60418689237655	2.48725632040592	29.67121781038754
C	3.50175281189740	2.40665975070048	27.72934795472517
H	3.53020652225668	1.32254838442296	27.69473530981549
C	4.04749872101795	3.11448679384104	26.61155307827621
C	3.99308640767101	4.53623961196294	26.69943180867614
H	4.39468489939390	5.12882941763970	25.88645436210887
O	4.51822451026596	2.50666907930380	25.57283828170109

Trans-**14** gas phase B3LYP

C	3.20296758124774	5.35768720654453	22.59328152103222
H	3.19847415102135	6.38610707133354	22.28029219165121
O	-0.77044212153809	5.57074684354928	22.70408466512174
C	3.79354183700185	3.45821042513760	23.55032096526215
H	4.40242147488247	2.81103606556431	24.16070486033070
C	2.61832384642934	3.24939160369612	22.89245079335794
H	2.01285235344885	2.36482198363708	22.80659149736994
C	1.13017797806043	4.67987962480562	21.37201192698472
H	1.26734834795971	3.99788874850607	20.52909491239210

H	1.22488068500276	5.70032909895242	20.99987165910001
C	-0.28089150958551	4.47417240933202	21.94069715881725
H	-0.96230437094156	4.38374191133059	21.09313780333867
H	-0.32943591145771	3.54059882333650	22.51186728794868
C	-0.00689101902663	5.81293382383284	23.83957451801290
C	0.69072157112883	7.01053215417840	23.93950196331873
H	0.51074268967326	7.79351468953002	23.21179862805748
N	4.14562327053887	4.77820541824493	23.33927894187086
C	5.35142785456719	5.45096590493456	23.85942559582164
H	5.98976910734355	4.70165713405441	24.32223527773738
H	5.06161000995968	6.17623820020614	24.61928953268042
H	5.87165997199342	5.93158025911818	23.03171772505857
N	2.25901215039227	4.45187527464314	22.29848636932578
N	3.25677167147150	6.07727951223772	26.37350965818424
N	3.65323705403218	4.90519768307459	26.63413319202147
C	1.68211725608556	7.13678809148709	24.91195980920716
H	2.30656913360233	8.02159005308411	24.96162620330523
C	1.98282150252823	6.05863682309285	25.75080017850984
C	1.13154519894560	4.94118955021065	25.76652375000583
H	1.32899753454395	4.14395908133297	26.46993053720641
C	0.13274782823320	4.82488481104705	24.81723044997491
H	-0.49241630245287	3.93964338404054	24.78323004032813
C	5.00803313859594	4.73686423846352	26.93852759850392
C	5.90482560495267	5.77594767737496	27.24545811827285
H	5.54652384177385	6.77212097570209	27.46309878102203
C	7.26374456148924	5.45081760678634	27.23521089559328
H	7.98898355925691	6.21845414947951	27.49226763353760
C	7.72674486301887	4.18722482630027	26.88172382626894
H	8.79224427674471	3.98254483004285	26.85891662000742
C	6.84195382159402	3.10184691806765	26.51275216931338
C	5.44697913371625	3.43669193092315	26.65595983196608
H	4.70589934734713	2.66889766758822	26.46092275902653
O	7.23229702641775	1.98218551519406	26.08788215314639

Cis-**11** QM/QM

S	2.6118917395	1.9289865194	32.7083644943
C	0.5849250243	4.9210797239	20.2939245358
H	0.1982434629	5.9487785899	20.4083413703
O	1.6641054362	4.6523592679	23.6753956538
O	2.1864864215	0.7124334135	32.1343147955
C	1.6585771876	3.1955419810	19.4232704965
H	2.2489570318	2.6729872257	18.6753017888
O	1.6683878146	2.5522458272	33.5457492193
C	1.1503576115	2.8054243340	20.6081396967
H	1.2270534442	1.8583081465	21.1354725648
O	3.9252254032	1.9019564410	33.2093171364
C	-0.2516609595	3.8840243097	22.4128043750
H	-0.9740818423	3.0597447853	22.3805506697
H	-0.7699020188	4.8367728080	22.5271021285
C	0.7022518657	3.6173041341	23.6014148237
H	0.0913222854	3.5842739309	24.5163349566
H	1.1902348686	2.6454067773	23.4730107899
C	2.5531623406	4.5809953747	24.6996854429
C	3.5050125285	5.6016622576	24.7916962128
H	3.5037034330	6.3926368300	24.0615596593
N	1.2881762938	4.5187175565	19.2426313401
C	1.6089721754	5.3543693979	18.0781859817
H	2.3355710431	6.1107040869	18.3756369953
H	0.6960100409	5.8429829142	17.7125989646
H	2.0149267974	4.6996254680	17.3048418354
N	0.4765226601	3.8979581816	21.1363148064
N	5.4682005600	4.5063870163	27.7342278860
N	5.2614251305	4.2627208350	28.9229009432
C	4.4462312179	5.5801729503	25.7999791095
H	5.1804480594	6.3777874398	25.8851172622
C	4.4246323075	4.5487762786	26.7357847507
C	3.4901814387	3.5293728743	26.6397671734

H	3.5232051469	2.7086834358	27.3433074984
C	2.5525758260	3.5397743606	25.6281117223
H	1.8472702577	2.7316858746	25.5525881514
C	3.9530981963	4.1152113939	29.5375440717
C	2.8594245377	4.9109559216	29.2170985457
H	2.9270042086	5.6523976865	28.4387971666
C	1.6734493021	4.7439382979	29.9147316726
H	0.8150699335	5.3403768696	29.6384867641
C	1.5906985225	3.8142903606	30.9432694933
H	0.6581699254	3.6948107660	31.4779306554
C	2.7017710445	3.0576804918	31.2961000568
C	3.8829150383	3.2080111622	30.5880891411
H	4.7592209238	2.6290946170	30.8428619414
O	-0.8575282555	4.9147476172	33.9504230825
H	-0.2409592555	5.0574642107	34.6901592282
H	-0.7853744213	5.7061745043	33.4016561506
O	-2.4507135576	5.8551994030	23.7326103947
H	-2.5448417144	6.5309658088	23.0433545687
H	-1.8223442070	6.2359451358	24.3620530218
O	1.5333386626	0.4823610607	29.5116086586
H	1.7623652354	0.5774677787	30.4461370465
H	0.6155201545	0.1026324305	29.4831403355
O	0.7416571862	2.7377086100	16.6979170070
H	0.5690045462	1.7803682147	16.7111832623
H	0.0637886424	3.1282153568	17.2700786137
O	3.0266790145	5.9053578427	21.2556978453
H	3.3356273471	5.2892304607	21.9363310812
H	3.5172568594	5.6399152599	20.4652588171
O	0.1096296120	6.8852697527	28.2225787943
H	-0.7224514162	7.2311434341	27.8639439724
H	0.7878999176	7.0210511065	27.5484123803
O	-0.5358823413	6.9599869443	16.6561077496
H	-0.9774080480	7.6776935295	17.1505211601
H	-1.2206218864	6.3518621236	16.3391601494

O	1.4369231807	-0.6723739903	19.3316183273
H	1.9414553350	-0.3544818792	20.0928523029
H	0.5264883009	-0.7914005984	19.6793623634
O	3.3678840884	10.0532211296	19.4618057439
H	3.1350430978	9.5614142675	20.2768466925
H	2.6158366016	9.9408213294	18.8602985400
O	-1.1844152533	1.1885355654	23.3917444585
H	-0.6352802784	1.3879945570	24.1709231838
H	-2.0598368432	1.0521493508	23.8053363918
O	0.0246258582	3.9653188751	14.4326963886
H	0.7309048492	4.5922504107	14.2146817154
H	0.3733741580	3.4395082205	15.1864699185
O	0.5704277900	7.0470481059	22.7661244691
H	1.2410853064	6.3568041267	22.6803024173
H	0.2602116925	6.9823520842	23.6968532621
O	7.3692600943	4.0122835716	25.8819557235
H	8.1742255218	3.6689096163	26.2717672043
H	6.7673318335	4.2317861180	26.6282293114
O	6.6749542979	1.7228797771	32.9710590142
H	5.7094954671	1.7188162992	32.9090913702
H	6.9691173401	0.8132800831	32.8881728497
O	2.4424381507	0.3382094758	34.8017771480
H	2.5802144129	0.7518059937	35.6630475872
H	2.9028957008	-0.5272709920	34.8332339969
O	-0.0857124683	1.2653960495	25.8666963058
H	0.1978845823	1.7316986549	26.6786283479
H	0.2832261268	0.3697749144	25.8995858283
O	4.1683610167	6.8119495060	16.9502017468
H	3.3536040117	7.1585887901	16.5303435297
H	4.6379343983	7.5767429551	17.3307675444
O	3.0781069945	1.1398542915	24.1426896510
H	3.8736402076	1.6824035174	24.1966323477
H	3.1873009504	0.4327971617	24.8040333239
O	-1.4665045015	3.0880540918	31.9623083729

H	-1.2376006071	2.1589778019	32.1612709266
H	-1.3442687964	3.5819210836	32.7895954343
O	-1.5493892842	5.5001831309	30.6475275883
H	-1.6668594187	4.6597887131	31.1101143286
H	-0.9050981558	6.0030037190	31.1797846182
O	2.9119436080	9.5142248430	28.3867746927
H	3.4256652630	8.8799264531	28.9448678297
H	2.1234267510	9.6977111194	28.9110421110
O	6.1937360861	8.2169253490	26.2593048898
H	5.5982909360	8.9714765211	26.3720548556
H	6.7427683643	8.4395521050	25.4756302539
O	0.6413090084	2.5733798622	28.1062919586
H	-0.2306999524	2.6039243843	28.5444556835
H	1.1536788570	1.9143581075	28.6058119209
O	4.7279843081	-0.1962622039	31.3099528722
H	3.8643736598	0.0850695560	31.6299233823
H	5.1367719927	-0.6968690718	32.0529140192
O	7.0925778443	3.7976550003	30.7300298359
H	7.9164275504	4.1695924155	30.3993982543
H	6.4461448060	3.9568461399	29.9786488466
O	3.9380198948	7.7342334065	30.0541921108
H	3.9432069654	7.9745056970	31.0035370430
H	4.8641417696	7.4708309201	29.8957668637
O	-1.4321526009	3.8342275781	18.1944555698
H	-1.6315100372	4.6011784180	18.7621419280
H	-1.7836164194	4.0916984218	17.3233936372
O	5.2577185839	0.6363339178	35.3516715185
H	4.6743357122	1.0173004024	34.6775500202
H	5.9311023995	1.2948718019	35.5398848580
O	-2.3801732486	5.0771495376	28.0707557415
H	-2.5872417203	5.9757773441	27.7666433297
H	-1.9804008784	5.2220575973	28.9486675687
O	-1.8183392867	7.6360992195	21.7187438308
H	-0.9131217875	7.3892412883	22.0092668636

H	-1.7787105453	8.5979416613	21.6871019373
O	4.4427591878	3.8136974754	22.2766896609
H	4.3147315699	3.1139105717	21.6043066703
H	4.7663107201	3.3692636060	23.0750536775
O	-0.0946349926	7.1294972058	32.2725301888
H	0.5810997744	7.1820979575	32.9767168591
H	0.2992277840	7.6542606267	31.5412272644
O	0.4382138081	7.8228773022	19.9392342705
H	0.9968186457	8.1010354390	20.6809402119
H	0.8831425450	8.1611614993	19.1474033054
O	3.3993333338	8.9071465062	24.2274441019
H	4.1704018883	8.4232742012	23.8746196114
H	3.7627107580	9.5911628475	24.8062385276
O	6.5057820756	6.9890041473	30.2393822221
H	7.1034956561	6.3642282315	29.7874270861
H	6.3361238640	6.6084738870	31.1131964115
O	-0.7566121920	2.1881829179	34.8816521693
H	-1.1772114995	3.0505958978	34.9174445839
H	0.0636949375	2.3198417952	34.3837962266
O	5.2944992092	1.5478246943	28.0975409088
H	6.0456947791	1.4303107483	28.7210382399
H	4.7079869095	0.7902133697	28.2987025152
O	1.3091796454	9.3223105418	17.7616719462
H	0.3717335706	9.5335238848	17.6751398645
H	1.5751499249	8.8894532042	16.9400278306
O	-0.5412117538	0.5647384247	32.2567424102
H	0.4159667735	0.6944173225	32.1851591676
H	-0.6669056215	0.1444000815	33.1321871601
O	4.4685736718	1.8601313947	20.4080860342
H	4.1841664555	2.0327704946	19.5018408730
H	5.4528957767	1.8767867426	20.4001355579
O	3.1028411248	4.8809049774	33.4730928102
H	2.4112148093	4.2061903823	33.4635618704
H	3.6893715317	4.6524901473	34.2298833130

O	3.9536177333	4.2994858082	15.7779427704
H	4.8538910853	4.0610079145	15.4776625939
H	4.0688485357	5.1420252894	16.2491901546
O	6.2887112377	3.2809464728	18.0584847638
H	5.4984895386	2.7264284025	17.8811443271
H	6.7907097194	2.8308397815	18.7552386729
O	2.5549505881	0.3420457038	21.6819680780
H	2.7471200421	0.5657274882	22.6203765046
H	3.2909970704	0.7549633685	21.1993166814
O	-2.3409863530	5.7834497023	19.8606017076
H	-2.8608520405	5.1773364542	20.4348753038
H	-2.0933624022	6.5250772348	20.4495280944
O	-3.4739859585	4.0996459780	21.5750312667
H	-3.2332101024	4.3913174334	22.4621993264
H	-2.9898584313	3.2814711667	21.3891587344
O	0.2229457652	-0.9629214164	22.8048581125
H	1.0504488339	-0.6505228839	22.4156870976
H	-0.3182002249	-0.1566418573	23.0002161052
O	2.1992311720	2.9966510810	36.1855651279
H	1.9536050302	2.7467031371	35.2784872121
H	3.1343511171	3.2527821487	36.1438468564
O	5.7384658106	5.3867539218	32.4016058943
H	4.8154254653	5.1066733352	32.4417051713
H	6.2057401160	4.7289702014	31.8336348321
O	7.1183022635	2.2475126650	20.4223760290
H	7.4127851600	1.6484835928	21.1282547068
H	7.1704176111	3.1406138575	20.8228182925
O	-0.2802532058	4.5232102263	26.4212477257
H	0.3709210922	4.0539648918	26.9584097637
H	-1.0397637151	4.6739239106	27.0147988238
O	5.3293385127	7.5149656943	22.9504580594
H	5.4924090679	7.4015801165	21.9949880410
H	5.8986423558	6.8605198147	23.3966951873
O	5.9373624873	0.3650075991	25.7935211071

H	5.2021085350	-0.2553782841	25.7109962935
H	5.8093988724	0.8060177674	26.6669565143
O	1.9643140574	7.8941923147	26.3695309419
H	2.3052554874	8.4975329330	27.0541294683
H	2.4622206221	8.1155042442	25.5611830108
O	-3.4402160732	3.9321285105	25.5792324327
H	-3.1484139804	4.5839728883	24.9283505527
H	-3.1673160091	4.2646685708	26.4398955340
O	4.6397154683	5.1908679089	19.0809992743
H	4.4347313356	5.7099985282	18.2835557536
H	5.2737407578	4.5049296197	18.7918739091
O	1.8879340451	7.5486311821	15.6564900858
H	1.0015062838	7.2772442917	15.9752162484
H	2.0529593102	6.9675001620	14.8988547103
O	4.6402979508	3.6114293218	35.1879726746
H	5.5980280651	3.8444454358	35.0857880747
H	4.4698318771	2.9596823782	34.4859295832
O	-2.0604934173	4.8564876606	15.7339444250
H	-2.8208596790	4.8771030483	15.1546592657
H	-1.2907872770	4.5346862543	15.1860452607
O	2.5925809633	8.5800624628	21.5704332644
H	2.7907601714	8.8194987394	22.4887063501
H	2.8540625584	7.6424165727	21.4819639217
O	3.8882807233	2.1929465478	17.6141406776
H	3.7038295984	2.7993444154	16.8762503706
H	3.6839284941	1.2812878972	17.3073695386
O	8.1376440723	5.4065629146	28.6622642810
H	8.3915098322	6.2887561473	28.3102885954
H	7.3628827657	5.1330925272	28.1526425000
O	2.3638852778	5.4247147576	13.9435445067
H	2.9145063859	4.9221179365	14.5887140967
H	2.7583056367	5.2950206054	13.0815275046
O	8.2190845315	7.9811068491	28.0214433506
H	7.4959553361	8.0611136668	27.3620082476

H	7.8339169927	8.2487062067	28.8603233794
O	-2.1483008718	2.0372387295	20.1378315850
H	-2.9841960565	1.6872589396	19.8248230709
H	-1.8149561020	2.6143200766	19.4214542468
O	-0.1027166724	9.8098549074	22.2743660487
H	0.5405714311	10.3356420489	21.7979534148
H	0.3700611667	9.0214989902	22.5773191129
O	5.6748080787	2.4680186654	24.3139540832
H	5.7621418190	1.6517863563	24.8828521046
H	6.2205645757	3.1292546142	24.7697461259
O	3.4102100855	-0.8746234915	25.9611542231
H	3.5410208198	-0.8795789968	26.9214544523
H	2.5065909094	-1.1966657762	25.8104271546
O	1.1000747115	8.3141922885	30.2024922148
H	1.9832611729	7.9329892023	30.2880548202
H	0.6738138443	7.7946502257	29.4795034646
O	7.1045087175	4.0330302089	34.4160257754
H	6.8568206794	4.7306707434	33.7888083630
H	7.2070657282	3.2327008577	33.8738496410
O	3.8074620863	-0.6319631425	28.7069321014
H	4.2542554563	-0.7782633010	29.5543326492
H	2.9170605884	-0.2963593180	28.9361789423
O	6.9741720165	1.0828646622	30.1022340984
H	7.1422819990	1.9209021447	30.5654866683
H	6.2980107738	0.6039177338	30.6016414268
O	5.3749984864	8.8566122759	18.3246663363
H	4.6131454500	9.3288223767	18.7725955169
H	5.9292939698	9.5441778019	17.9574554514
O	-2.9352701168	1.2197687872	25.3937981813
H	-3.2473264753	2.1392160541	25.4408402753
H	-2.0944474703	1.2103197815	25.8726785932
O	0.2247469412	0.0988701600	17.0332460429
H	0.6660109555	-0.1056158718	17.8822139112
H	-0.6635908056	-0.2495074610	17.1098658569

O	3.2956428957	-2.2547086769	34.5636471314
H	2.6379377124	-2.2850994317	33.8236114336
H	3.0590391308	-2.9517207862	35.1761571852
O	-0.3147347757	6.8925135244	25.2496374810
H	0.4057668243	7.4237625118	25.6252962465
H	-0.2133782455	6.0004495806	25.6517633856
O	4.4375529528	10.4199755095	26.3448972601
H	3.8706454362	10.1833436918	27.1033020648
H	4.7950940938	11.2902343627	26.5197155741
O	-2.7179526190	7.9492926499	30.3322010752
H	-2.5023681562	7.0138114632	30.5013514776
H	-2.0581442576	8.4504909744	30.8170628107
O	4.1110741462	8.0245784514	32.7271348214
H	3.3267214761	7.9060745440	33.2792464970
H	4.7007749171	7.2864695614	32.9107814269
O	5.6987878151	-1.3293554249	33.5242453979
H	5.7278082575	-0.6763845045	34.2485935809
H	4.9869958101	-1.9280808514	33.7890675110
O	6.5901715550	3.8604517294	15.4371607081
H	6.6671884185	3.6125607521	16.3805224839
H	7.2066568180	4.5785065656	15.2924216601
O	1.4413588031	-1.9193682341	32.7289411314
H	1.7307620009	-1.0444228411	32.4310259290
H	0.6323952772	-1.7757886711	33.2335469916
O	7.3664102043	5.9624130518	23.9046797355
H	7.3868263110	5.3531188613	24.6566819808
H	7.3976957041	5.4073615482	23.1049155974
O	-1.0488248713	-0.5886371855	20.3243624220
H	-0.8417704622	-0.8982744747	21.2200686865
H	-1.3410111506	0.3324710448	20.4113479269
O	-1.5180313721	8.9103427233	18.2677788153
H	-2.4182925011	8.5404671530	18.2435205850
H	-1.1016554099	8.5642048043	19.0739759042
O	1.8337895109	7.0841620112	34.1797986537

H	2.2998866441	6.2841630936	33.8465510930
H	1.4761913899	6.8109201204	35.0327749459
O	-2.4552288347	7.7869051242	27.6902316262
H	-2.6245132452	7.9567156082	28.6471872777
H	-2.8863589027	8.4798391110	27.1919426122
O	7.4399668067	1.0064880419	22.7875643247
H	7.5225483406	0.2324654743	23.3451269138
H	6.8201675776	1.6013336110	23.2507996204
O	-1.8296027973	2.4884546074	29.2311669129
H	-1.8009669957	2.7372787523	30.1662265805
H	-2.3486403150	3.1563783506	28.7706221302
O	7.5539788058	8.7694288304	23.9958070760
H	7.9935568004	7.9100669979	23.9645084367
H	6.7684850289	8.6581110964	23.4361917355
O	-0.1804737648	-0.4645936644	34.7077081015
H	-0.6723494816	0.2625633326	35.1126252139
H	0.7532748650	-0.1834288036	34.7688116537
O	-1.0073318092	-0.1645780175	29.6462771362
H	-1.0479558812	-0.0727163644	30.6118956304
H	-1.4655532341	0.6121479454	29.2953829392
O	6.8716898852	4.6905548004	21.5548665973
H	6.8257298447	5.5128965814	21.0493937300
H	5.9429800035	4.4730209489	21.8080937146
O	0.8158484257	5.2794065278	36.1230517671
H	0.3330288096	5.3740642847	36.9442505229
H	1.3289500153	4.4373382911	36.2038154670
O	6.0218116953	7.0770823308	20.3576107767
H	5.4627854738	6.3914192453	19.9316125918
H	5.9903415822	7.8253784757	19.7439991551
O	0.7460302762	-1.3569483450	25.4143954730
H	0.1349466338	-2.0074258191	25.7607271250
H	0.5852441515	-1.3072457360	24.4417289142
O	-3.7603877545	7.3626195966	18.2294333130
H	-4.6033997515	7.4995309546	18.6604706852

H	-3.2853756814	6.6798417799	18.7534799643
O	3.0876783579	-0.2773114869	17.0360490199
H	2.3118328470	-0.2201800866	16.4690082265
H	2.7572139217	-0.6114392670	17.8823942469

Trans-**11** QM/QM

S	6.5473277742	3.8737256448	32.4316845451
C	1.3271013707	4.8629296674	20.2303281018
H	1.1583744216	5.8879487755	20.6137316606
O	0.6435781180	5.0730686064	23.0230013986
O	6.8818794989	4.7945463604	33.4390289075
C	2.2009482523	3.1401674979	19.1523300726
H	2.8962456546	2.6259706090	18.4999301997
O	7.5360907820	2.8884988913	32.2300177167
C	1.1337803972	2.6901255729	19.8432281012
H	0.6999385247	1.6960494933	19.9501942458
O	5.2556674074	3.3222616280	32.5211623178
C	-0.5425843702	3.7501045353	21.4310317230
H	-1.0741599626	2.8126557954	21.2651018300
H	-1.1985172856	4.5950453756	21.2030542476
C	-0.0123372643	3.8226201703	22.8856548849
H	-0.8514619201	3.7054075248	23.5878457883
H	0.6863559370	2.9953975218	23.0405748676
C	1.5934303119	5.1889333844	23.9880604110
C	2.5816275294	6.1606805054	23.7927926810
H	2.6063376158	6.6842852158	22.8449718396
N	2.3040589037	4.4990227797	19.4096477532
C	3.2771171425	5.4230989031	18.8127239741
H	4.2297957154	4.9029270593	18.7054923896
H	3.3788569514	6.2791088221	19.4916009812
H	2.8854243026	5.7540933287	17.8472408392
N	0.5962955789	3.7842595913	20.5041873613
N	4.4570782008	5.9308218774	26.9729144432
N	4.7719694716	4.9883585268	27.6990584836

C	3.5223756043	6.3863201427	24.7776440035
H	4.2963058168	7.1304740453	24.6409025502
C	3.4975229269	5.6285335438	25.9475492075
C	2.5270992789	4.6511705695	26.1269708615
H	2.4926856555	4.0947763978	27.0530553843
C	1.5697273435	4.4278171912	25.1557094363
H	0.8074590964	3.6836568313	25.3132915586
C	5.7215643007	5.3207681847	28.7401872535
C	6.6401103299	6.3542351090	28.5968793708
H	6.6639515710	6.9246527891	27.6858257334
C	7.5299193032	6.6204756742	29.6225471999
H	8.2506674736	7.4214730604	29.5427110139
C	7.4943138368	5.8611660573	30.7824131733
H	8.2065650705	6.0809159358	31.5649003466
C	6.5713789457	4.8325050787	30.9195998337
C	5.6947534252	4.5441508390	29.8880394806
H	4.9799056241	3.7364502323	29.9561905118
O	1.2745901367	8.9535428201	23.4605126314
H	1.7330705352	8.3057678341	24.0091477094
H	0.9340405721	9.6342534283	24.0654967931
O	6.8764453128	7.4290075109	25.1643839811
H	7.7000283741	7.7624224998	25.5927709662
H	6.7665019167	6.5143281700	25.4747338775
O	7.7058404853	10.4376870167	30.1158601724
H	7.4068612296	10.2468013027	29.2110850753
H	7.0147670781	10.1079492630	30.7098351837
O	4.4067471437	7.2748339437	34.7586008306
H	3.4514500537	7.0901542034	34.6622806929
H	4.7506348162	6.6016230120	35.3723616339
O	9.4308688194	5.1068890057	34.1379479551
H	8.5176941800	4.9334148251	33.8447137048
H	9.6881869909	5.9408472899	33.7058843542
O	3.0205200034	2.8805164430	22.6328989505
H	3.7706123717	3.3903400597	22.9627728497

H	3.3684271235	2.3272806901	21.9093678029
O	4.5611155648	8.3980346246	27.8983260371
H	4.5902709376	7.5117341511	27.4361681615
H	4.4877088248	8.2020638554	28.8480539422
O	0.7332980303	4.8052508050	16.9298069640
H	1.1395778405	3.9695761396	16.6318585596
H	0.5532093958	5.3258879994	16.1466380028
O	12.2601021847	6.0679627483	31.7131638763
H	12.1890244004	5.1958899721	32.1518523372
H	11.6774754032	6.6549166009	32.2037789454
O	5.1230856445	3.6287595617	20.6245928664
H	5.1430693917	4.5831086559	20.7945981865
H	5.7796887888	3.2669635619	21.2432007702
O	6.3994752761	0.8165851083	30.9234586589
H	6.8251266107	1.5620030052	31.3829389350
H	5.4519905604	0.8824833342	31.0618566534
O	5.5398013617	5.9693668010	22.0191290161
H	6.4494846288	6.2704446055	22.1821346091
H	5.3140700398	5.3897650140	22.7640276788
O	0.4727776001	0.4050139067	17.3189680172
H	-0.3453161977	0.8616918136	17.6643386036
H	0.1519832812	-0.3467760178	16.8201058186
O	6.4950849124	3.6498898786	17.1872091371
H	6.5281672981	4.3549339032	17.8531385051
H	6.5645842471	2.8271127468	17.7042503346
O	-0.2024080858	0.0803756973	20.4838890060
H	0.1032228133	-0.8283742648	20.5947982661
H	-1.1833940031	0.0829093493	20.4921084598
O	3.4267956895	7.4394457936	21.0929457596
H	4.2558421654	7.0344210251	21.4040676064
H	3.5757315547	8.3840319037	21.0356999026
O	1.7994903918	1.7031912736	27.0861849628
H	1.9122224283	1.4716617641	26.1468044054
H	2.7164172675	1.7781009836	27.4191492816

O	2.0472629630	6.1255852815	34.0762839433
H	1.9252135899	6.0026007193	33.1277460388
H	2.4017448461	5.2718430442	34.3847582539
O	-2.3464269047	3.0056617072	24.6492146128
H	-2.6177396526	3.8246141127	25.1170411991
H	-1.8494165631	2.4607145851	25.3003725050
O	8.9833901508	8.1610106105	26.6159450936
H	8.4044124658	8.6345528655	27.2372382910
H	9.1976875051	7.2772942998	26.9801965617
O	0.3993340049	7.5749269791	21.1484074738
H	0.5240453180	7.9415581114	22.0325809963
H	0.6017397155	8.3128317802	20.5341606338
O	9.1409212866	1.0813768861	33.4509529356
H	8.5795901013	1.7759579742	33.0674596850
H	8.5788177725	0.2718279963	33.4150003753
O	4.3707958544	2.3292948548	27.6928225410
H	4.4891954747	3.3130716767	27.6793100121
H	4.5973942129	2.0270727997	26.7831069687
O	2.5077487764	1.4412233476	33.8096868163
H	3.3572610140	0.9866362312	33.9428256895
H	2.5960809561	2.2754434838	34.2936465964
O	-3.8794699805	4.1023189730	22.5366765821
H	-3.1927356007	3.7057562419	23.0889280642
H	-4.6298790503	4.2317086691	23.1432044894
O	1.0168205984	3.0129774538	29.3883136735
H	1.6379306093	2.6549301717	30.0383469766
H	1.2272965456	2.5653736391	28.5504343131
O	4.7261997482	6.6690427292	16.0188306817
H	3.9885023301	7.0714354846	15.5372626859
H	4.4994360849	5.7299764929	16.1183423493
O	-1.5129323555	4.2244089420	18.4094743506
H	-1.6377307147	5.0201875526	18.9564681887
H	-0.7364456204	4.4321699675	17.8602225484
O	1.7026476966	7.8961672514	17.5233112324

H	1.8797431579	7.8273443715	16.5769128753
H	0.7439546437	7.7130915266	17.6274672357
O	3.9056282064	1.1141694022	17.3418368132
H	3.4241865827	0.4721209902	17.8957184183
H	4.0472542140	0.6972180655	16.4921303447
O	5.7898215679	5.1618730418	35.8454030468
H	6.1333674072	5.0574073236	34.9356619991
H	6.5909826372	5.3883113651	36.3628152409
O	-1.7045715417	6.4197626551	19.9900163107
H	-2.5013673642	6.6067368324	20.5456822619
H	-0.9548800352	6.8184842926	20.4796904619
O	-0.7189721774	5.5880690013	27.2331420250
H	0.0157711471	5.9981992602	26.7615262468
H	-0.6019005053	5.8316033489	28.1621762336
O	-1.5061637839	6.8317714717	23.6585654739
H	-0.7028644338	6.3996438047	23.3308347136
H	-1.2420717122	7.5310386951	24.2906660049
O	6.9928996088	4.7556335015	25.8544085044
H	7.2485548378	4.3356356769	26.6959589065
H	7.8184942350	4.7329758437	25.3304391358
O	3.9531850033	0.9017181702	20.9183717386
H	3.3325405453	0.5707899663	20.2438632633
H	4.8036466266	1.0112504937	20.4725511425
O	2.3878140784	5.0260398439	31.4794638290
H	2.5676270333	5.2788363446	30.5640663648
H	2.4625725617	4.0481240314	31.5054076412
O	6.9245397336	7.5945890370	33.5824429288
H	6.2077564957	7.7173782701	34.2200708285
H	6.8824230230	6.6537590520	33.3474866593
O	2.1476340896	1.0753686044	24.4032106949
H	2.4486645752	1.7951043960	23.8010845016
H	1.2460740277	0.8637728763	24.0862743831
O	9.7702276100	5.6544321804	27.2347705052
H	9.1843090238	4.9520789289	27.5654201177

H	10.4013838735	5.8251922018	27.9654490781
O	6.8699159993	1.3353850086	28.2609119767
H	5.9511546375	1.5997149497	28.0690593520
H	6.8574488738	1.0028017207	29.1701723510
O	4.1265153676	3.9281375891	16.0086424536
H	5.0265814347	3.7449011063	16.3690457036
H	3.5560960712	3.2155550632	16.3233371181
O	-0.1273888858	0.7259587663	23.1256129980
H	-0.0381023137	0.5211045952	22.1771308823
H	-1.0637882772	0.6162703274	23.3447836579
O	4.2177899605	7.8189641116	30.5350587025
H	4.5181816550	7.0574061043	31.0755362092
H	4.6885245174	8.5696824340	30.9365364482
O	1.3793196744	7.5204325713	26.6840573040
H	1.9840375027	8.2181728318	26.3760961220
H	1.8142745502	7.1384929323	27.4685686843
O	2.9552690625	2.4237078912	31.3999571478
H	2.6104806743	1.9582221165	32.2016653100
H	3.8422980484	2.7095173588	31.6686936757
O	5.4962053288	9.8750435871	22.2151709126
H	4.5812427641	10.0830026938	21.9760330541
H	5.7933894431	9.1887135325	21.5954864135
O	4.8145352135	1.5368527034	25.2009398286
H	4.9322881945	2.3374237078	24.6628502690
H	3.9858989729	1.1292475611	24.9280251714
O	-2.8754034862	5.4619762424	25.5577258718
H	-2.5019536809	5.9780146112	24.8137399554
H	-2.2604034772	5.6173196861	26.2927205779
O	5.2330626056	0.9673542023	33.9296285068
H	5.2635923385	1.7401457423	33.3451195501
H	5.6264666899	1.2957325859	34.7605605651
O	9.4584409103	3.5571592812	30.4314904182
H	8.7911419848	3.3180399666	31.0891736711
H	10.1570922483	2.8967552130	30.5256839862

O	6.6757714754	5.7456640608	19.0289581491
H	7.0674573949	6.1866407186	18.2479191167
H	6.3280724493	6.4660830099	19.5862885214
O	-3.6038721719	2.9278622466	20.0767027703
H	-3.0797333480	3.5080681000	19.5144357371
H	-3.7977507393	3.4152808949	20.8954821905
O	3.0823112439	-0.9473764498	22.7326560023
H	3.5521987303	-0.3213108503	22.1531296455
H	2.7135771449	-0.3982609662	23.4448982494
O	4.3510424992	8.4315509538	18.1339709643
H	4.6664005609	7.8455771435	17.4274517369
H	3.3977921218	8.2653114390	18.1690472871
O	0.7951240939	10.8300466611	25.3916436255
H	1.6331458129	10.4822429342	25.7692319987
H	0.8241424740	11.7822123315	25.4788163269
O	5.4191255772	9.7274455260	24.9002255454
H	5.5089946161	9.8781690757	23.9406565904
H	5.9042383264	8.9005495244	25.0634611613
O	9.7668322372	8.7535264050	30.3161355256
H	10.4174717935	9.0659280063	29.6586515357
H	9.0664997463	9.4430745189	30.3264267227
O	8.1618937257	3.6218928830	28.0582887168
H	7.7493495944	2.7253694592	28.0437697771
H	8.6159286778	3.6588870234	28.9240540023
O	-3.5496376402	6.7540226758	21.8188440194
H	-3.8095299398	5.8395603012	22.0217772258
H	-2.9201114040	6.9910987787	22.5190455675
O	8.0749185018	6.5866766273	22.8987660907
H	7.6330487330	7.1325429483	23.5773634666
H	8.6673338601	6.0180116479	23.4134236442
O	8.3241841442	5.3825946526	36.7100460779
H	8.8072235961	5.4817076636	35.8718412527
H	8.6258371067	4.5483538266	37.0797000153
O	5.9826382884	1.6792963654	19.0062370507

H	5.6581983694	2.4627192463	19.4949962619
H	5.2718225110	1.4305460642	18.3919632700
O	-2.8327488319	0.4500086787	20.7250422372
H	-3.2105468578	1.3244794156	20.4926348231
H	-2.9754306089	0.3692378132	21.6784484486
O	7.2453627015	-0.7419247733	33.0775019652
H	7.0891874439	-0.5657307945	32.1388589751
H	6.4913273211	-0.3257029481	33.5307979059
O	0.0734663473	5.5266945087	29.8571736968
H	0.3973155385	4.6113118108	29.7146666402
H	-0.1516363951	5.5925533872	30.7860980365
O	-2.8858991124	0.6103825996	23.5409099489
H	-3.0594223354	0.0561534413	24.3028176647
H	-2.8565513431	1.5271252785	23.8771042409
O	1.5025722364	-2.0716260910	20.9657826780
H	2.0362667339	-1.6556299844	21.6986635129
H	1.6681954068	-3.0118955864	21.0277006645
O	-5.3715348446	4.6143141762	24.7478640479
H	-5.7348327848	4.0574079995	25.4364427499
H	-4.5959766591	5.0589771560	25.1304186740
O	9.6696767238	7.4555835927	32.7263625856
H	8.8730607980	7.8315471533	33.1191602924
H	9.7985902668	7.9418400721	31.8883748543
O	1.0772380579	9.6453374261	19.5712450536
H	1.8284352062	9.9186952642	20.1175952138
H	1.4399273712	9.1800830047	18.8012917764
O	3.5670998632	3.9126584520	34.6023861370
H	4.1477682750	3.7950634224	33.8299488498
H	4.1435312499	4.1863247434	35.3251971462
O	4.7398660068	5.9355715567	32.3963361656
H	4.6065204186	6.4750877833	33.2038993768
H	3.8672081759	5.5423009703	32.1889213669
O	10.9877645299	1.5126916608	31.5722779429
H	11.6747091778	0.8470105079	31.5495546503

H	10.3438610507	1.2350607102	32.2643345706
O	11.6851572628	3.7853649822	33.0333183469
H	11.4680663117	3.0315854088	32.4619648072
H	10.8551185400	4.1099444073	33.4091839970
O	2.4413410194	-0.4020739034	19.0281993428
H	1.6273099494	-0.0781614591	18.6032120251
H	2.1529649357	-1.0839490687	19.6654165012
O	5.4329869778	3.8734626138	23.8642392697
H	6.1286104462	3.4564466297	23.3229151682
H	5.9011151781	4.2001047786	24.6582940996
O	11.3011743155	8.9995599009	28.0622853723
H	10.6339324260	8.8690836676	27.3718074437
H	11.6397080770	8.1178018902	28.2741144005
O	1.6902250198	2.4171436301	15.9916017749
H	1.3044257426	1.7013395551	16.5397152237
H	1.2492091808	2.3888781406	15.1417372559
O	2.4515671856	6.4189492007	28.9495392648
H	2.8955928756	7.0694925506	29.5155496697
H	1.5494236145	6.2911599479	29.3004097627
O	2.3278813303	7.3734241854	14.8092433660
H	2.2358378287	6.4367592011	14.5246817992
H	2.0175169041	7.9256343050	14.0921612302
O	-0.9573084868	7.8897378411	17.8497991978
H	-1.3128296988	7.2949755951	18.5421005464
H	-0.9960398245	8.7725250712	18.2251650337
O	5.8596030855	9.4340154824	31.9483632250
H	6.3243493895	8.7263787178	32.4526943274
H	5.5500773202	10.0670937991	32.5966952858
O	6.1026387877	8.1980419522	20.0922156308
H	5.3929570572	8.3323974654	19.4169712890
H	6.8821667071	8.6411196844	19.7548429956
O	9.1955551926	2.6858512254	35.6598793062
H	9.3428305916	1.9546870089	35.0341585456
H	9.5177741712	3.4737901075	35.2060403101

O	6.8838798774	9.6471452549	27.5893468241
H	6.7605431209	10.1939787433	26.8091077323
H	6.0212269575	9.1897816212	27.7212865861
O	6.4883815841	2.4407338016	35.7618863839
H	7.4635896330	2.4685441270	35.8099487999
H	6.1771590791	3.3117037481	36.0407405821
O	7.4179877220	6.5797190866	16.5855706868
H	7.6773248292	5.7245670291	16.2334454242
H	6.5152282574	6.7382135624	16.2627529722
O	-1.0854830335	1.4791440954	26.4274696978
H	-1.3996396003	1.9636685320	27.2211851053
H	-0.1388404310	1.3623469364	26.5615356067
O	2.7962152356	10.2785166371	21.6927331840
H	2.2634303542	9.7367652648	22.3230894316
H	2.5942481554	11.1945851472	21.8854495239
O	11.1873400351	6.4766021726	29.3110774077
H	11.7214375885	6.1662591170	30.0722500750
H	10.5130062313	7.0334801144	29.7224425167
O	7.2650813586	2.8610098872	22.1469525962
H	7.7868713522	3.5549335004	21.6516116661
H	7.6342464948	2.0119292175	21.9071578336
O	2.4067006001	4.7914028515	14.1036240255
H	3.0070971852	4.4463388907	14.7967072950
H	2.8481595494	4.6283975995	13.2695602310
O	8.4544541317	4.7539969936	20.8033172387
H	8.4852529297	5.5023885823	21.4137650757
H	7.8331374989	5.0266450484	20.0965333051
O	3.0630932615	9.5969329747	26.0787137789
H	3.4899600242	9.3513216727	26.9220016398
H	3.8474120843	9.7030451418	25.4895255377
O	-1.7511451677	1.5313544139	17.9984252353
H	-2.2546294483	1.1666505387	18.7319113680
H	-1.7060019575	2.4937825953	18.1414573547
O	9.4498017121	4.9330195135	24.6862846720

H	9.7516612698	5.2863266839	25.5516986281
H	10.0065803178	4.1813104185	24.4836068731
O	-0.8956273274	8.5478364471	25.6308680418
H	-0.6769484150	9.4826261267	25.5704236877
H	-0.1599573408	8.1405594969	26.1195363796
O	-1.6081499734	3.1641628973	28.4225119498
H	-1.4118829289	3.9779376040	27.9366357189
H	-0.8318206556	2.9958160327	28.9738938574

Cis-**12** QM/QM

S	4.9571011634	0.1267752492	27.8459650048
C	11.4777903264	6.0311526833	29.9371550441
H	10.7785834669	5.3236110822	30.4307364847
O	8.9214706012	6.6007418865	29.4061834765
O	5.2749218048	-1.2384795615	27.8650533693
C	13.1893069582	6.9343970855	28.8568265453
H	14.1188271191	6.9143517165	28.2942463907
O	5.8925441053	0.9427246483	27.1807294874
C	12.4393722941	7.9391159057	29.3533733453
H	12.5001185305	9.0264447454	29.3128111227
O	3.6136794993	0.4527377822	27.5966515631
C	10.2821201313	8.0671075541	30.6788476702
H	10.5750220252	9.1152306514	30.7661395416
H	10.1315321291	7.6212210737	31.6636209243
C	8.9904534873	7.9473627851	29.8150901813
H	8.1227112600	8.2540860464	30.4140870470
H	9.0609242843	8.6167629476	28.9531148887
C	7.7356450681	5.9688195631	29.2856495066
C	7.7944343483	4.5722628460	29.3133931134
H	8.7516205216	4.0997019005	29.4446541498
N	12.5635989129	5.7492596286	29.2206869007
C	12.9881474327	4.4109601988	28.7789679684
H	12.5723852346	4.2159513970	27.7838131751
H	12.6277033828	3.6698601502	29.4966563894

H	14.0781676182	4.3896024317	28.7351278056
N	11.3794375943	7.3525675867	30.0228639555
N	4.1607211466	3.7957851068	28.8956143459
N	3.8903185430	2.6549800026	29.2787179876
C	6.6437646479	3.8314453885	29.2097676936
H	6.7190425098	2.7591789455	29.2376818868
C	5.4134945074	4.4722999925	29.0626067771
C	5.3667760949	5.8630199519	28.9855410445
H	4.4146889518	6.3526050158	28.8397214763
C	6.5158349164	6.6238806873	29.1051949673
H	6.4751261012	7.7030559573	29.0496025728
C	4.7696535691	1.8167611463	30.0859488605
C	5.1939749676	0.5897930351	29.5875387172
C	5.9159280594	-0.2527424942	30.4209342223
H	6.2717637341	-1.2053135105	30.0545416925
C	6.2126240076	0.1155364805	31.7245213021
C	5.8062833650	1.3563748785	32.1983843324
C	5.0811767662	2.2055256395	31.3806234657
H	4.7703815553	3.1743319404	31.7312345486
H	6.0633104652	1.6790020919	33.1936117753
H	6.7675044370	-0.5664680407	32.3508780156
O	8.1193350263	-2.9080349818	30.0268007477
H	8.7252644951	-2.3429160686	29.4944707746
H	7.7201249209	-3.5226975906	29.3930762186
O	3.6270062436	4.9731600125	31.3054221625
H	4.2586784806	5.2623370107	31.9887573952
H	3.0019270735	4.3785279162	31.7648648862
O	13.3527728426	0.9521789791	29.6908394842
H	12.8832669072	0.5463801910	28.9429615765
H	12.6801566007	1.4220063641	30.2037301011
O	13.3197700006	8.7265185769	26.2831437974
H	12.4867446490	8.5173321613	26.7391348286
H	13.8120733732	7.8754899561	26.2646312981
O	5.0670879876	5.3875773491	33.6077176494

H	5.3680411272	4.6308010310	34.1390275376
H	4.2001541286	5.6553085320	33.9545829934
O	2.8783880551	0.8605215065	24.0769805344
H	2.7761822323	1.5973393274	24.7027955311
H	3.4711002857	0.2279503237	24.5439308356
O	12.9225904192	4.3421187146	32.6632017653
H	12.7739132425	5.2938209223	32.5092080632
H	13.6791533071	4.3001207021	33.2537774781
O	12.7192267865	5.1639736280	24.6252449051
H	13.1674413022	4.3386293018	24.3680312840
H	12.0285238711	4.8552830233	25.2584261141
O	9.6194310834	4.7449615211	31.7701491604
H	8.8021205836	5.2507866764	31.8924048499
H	9.9605037258	4.5943719275	32.6663055274
O	15.2954964820	3.6776607136	26.7429334663
H	14.8449221585	3.2163752370	26.0156562749
H	14.9896139942	4.5992891273	26.6937246373
O	4.1312949777	4.1028939722	24.0462090735
H	4.1919622194	3.4913778749	23.2924747914
H	3.8401695512	3.5599787375	24.8020082367
O	3.7193301122	-3.1436640359	25.9871231247
H	2.7614311879	-3.0803515193	26.2133091039
H	4.1368314582	-3.4741515399	26.7862619056
O	6.7297754370	9.8401170991	28.8989530536
H	7.4605703282	9.9975989058	28.2843434495
H	6.0041326728	10.4276090357	28.6338458908
O	12.1080956466	9.6010012931	32.9566861798
H	11.3029900206	9.9165784368	32.5130594457
H	11.8242652309	9.5180155801	33.8944523918
O	11.5590936439	6.6293601868	34.8405788434
H	11.3376600350	7.4846416167	35.2211153820
H	12.0698971484	6.8119605225	34.0312393266
O	11.8646772265	10.8217237572	29.6502467941
H	12.1444445503	11.1879808030	28.7894440422

H	12.4754653723	11.1707162385	30.3290721198
O	3.6776540077	8.3747162066	27.4987092061
H	3.8526029340	7.6951446760	26.8336740641
H	2.7565738181	8.1861593647	27.7917550878
O	1.7072436475	-0.2068446055	29.3413634856
H	2.4218340058	0.0460526377	28.7349930810
H	1.7668102139	-1.1754096475	29.4556062038
O	9.5887649008	10.4431970598	32.2302241458
H	9.2410274100	9.6846728854	32.7388698904
H	8.8124821476	10.7859344249	31.7347316703
O	7.1242016653	-2.6382060249	32.9845471561
H	7.8174065909	-3.3086114082	32.9449521465
H	6.9642570117	-2.4254895983	33.9157264889
O	10.9061006711	4.1243868620	26.2721753557
H	10.5205718632	3.4670364829	25.6552077702
H	10.1941889070	4.7965445191	26.3924444093
O	9.8932472113	12.3736265441	27.2094274395
H	9.8665726335	12.6646974028	28.1341052005
H	9.5420202497	11.4562797540	27.2081956394
O	7.1884515733	-0.0000133112	24.9615376682
H	7.6145010016	-0.8012354689	25.2899301574
H	6.7717744886	0.4144126167	25.7354785904
O	3.7190491317	2.1833627949	34.9326741177
H	3.8386352216	1.2255825470	34.8127794278
H	4.6069575660	2.5564884602	35.0250236003
O	12.9414747277	7.0634898133	32.4645894910
H	13.9124860155	6.9692858195	32.6321749466
H	12.7266635637	8.0122945335	32.5540419974
O	3.1526211396	2.6730432352	26.1218081729
H	3.3300425988	1.9236400930	26.7147625087
H	2.6412764773	3.3265969855	26.6327795884
O	4.6098036498	-3.5485911403	28.8845786729
H	4.8071059134	-3.5095547644	29.8548996554
H	4.8017690807	-2.6505270382	28.5435070442

O	2.8851729026	-1.6369913805	32.3682111388
H	2.5115592683	-0.7773744000	32.0988612095
H	3.2252551580	-1.4773968539	33.2599595515
O	2.0915644812	4.7721787510	27.6657892384
H	2.9132883423	4.5325201516	28.1964830124
H	1.4267855716	4.1574546705	28.0191956040
O	9.3200080466	-0.9282055625	31.9616606705
H	10.1551680113	-1.4290967076	31.8636702340
H	8.6286562469	-1.4870803791	31.5864405351
O	8.8078733556	-0.0546549288	34.5115623874
H	9.0319717464	-0.4310706742	33.6398283225
H	8.6767194099	0.8983668905	34.3537558418
O	2.2927091953	3.2269349962	32.9066555175
H	2.8095948682	2.8525706373	33.6550256605
H	2.0276645390	2.4475009609	32.3897034300
O	7.5804025863	2.9975567185	26.4602174454
H	6.8541485283	2.3900799020	26.6308708826
H	7.2559998835	3.6326362385	25.7848883231
O	16.2524959736	6.3657215670	29.2138998394
H	15.8958872400	5.7234954267	29.8510886924
H	16.8515051845	5.8424610315	28.6340495096
O	6.4245806863	6.5029476801	26.0575099881
H	5.4661476268	6.3875380920	26.1651866123
H	6.6661747292	5.9463209640	25.3013232771
O	8.1030850639	1.0383690685	28.7486611484
H	7.3911817977	0.9981952066	28.0931786710
H	8.7813314533	1.6566510202	28.4043470904
O	9.9868967049	12.7506200060	30.0086992674
H	10.3774248913	13.1898334605	30.7773738046
H	10.4677843959	11.9011572199	29.9552396321
O	14.9697000295	9.5199937477	30.5362999897
H	14.9635981578	9.7656218375	29.5900067045
H	14.4314465384	8.7255626116	30.6087847784
O	4.0480475972	9.1125028585	30.1312020779

H	4.0948660479	8.8408872099	29.1979775216
H	3.3582869397	8.5539719554	30.5323923376
O	7.2288494471	2.0381985538	23.0965154799
H	7.2658133661	1.2784234678	23.7015760563
H	6.3465888602	2.0001376347	22.6994152132
O	10.3078593885	2.4677958213	28.2993844406
H	10.5437005141	3.1441183695	27.6334861397
H	10.7353269738	2.6924509284	29.1376915597
O	10.7303310114	7.9853794089	26.7747976302
H	10.1542763310	7.2226707214	26.9797833525
H	10.8884786403	7.9062207210	25.8138577404
O	6.2020226715	8.4020888318	31.5586255938
H	5.4619327623	8.6251836972	30.9559135344
H	6.0406442374	8.9059661940	32.3606907367
O	14.5146794068	6.3254173081	26.1822773727
H	15.3652873168	6.5024448071	25.7497506397
H	13.9007704902	5.9254345008	25.5223648492
O	9.0618619186	5.9662270671	26.4486831447
H	8.1712198716	5.8558987617	26.8019642264
H	8.9373570031	6.3026372806	25.5284284963
O	7.0672442486	9.0172920581	25.3901528865
H	6.8030323526	8.1216237112	25.7043915788
H	6.3028739650	9.5845372874	25.5737040814
O	9.0068122099	1.6485520617	31.3361237992
H	8.5197033450	1.5994125729	30.5031728209
H	9.1754351210	0.7140732800	31.5766081931
O	7.2483670800	11.0949541396	31.1928202817
H	7.1960331683	11.9849315602	30.7747072514
H	7.0389869763	10.4693371252	30.4629959006
O	6.7672675685	-1.3850534652	35.4266923575
H	6.9299055214	-1.5827954969	36.3481812062
H	7.5368716365	-0.8277696281	35.1172646294
O	2.3382772554	5.7395757459	34.0512259143
H	1.9209762196	5.7057972030	34.9120973497

H	2.1528258871	4.8822683810	33.6288983450
O	4.4420840785	-0.4350223909	34.3456360069
H	4.8086272496	-0.1666123446	33.4928037843
H	5.1935541480	-0.8138438871	34.8364452416
O	11.4355525315	2.6661090780	30.8387141177
H	11.7961293713	3.1894800675	31.5634987450
H	10.5513525315	2.3521630824	31.1300477492
O	6.9309559037	-4.5383292216	28.0857478191
H	6.0172989111	-4.2627991440	28.3529644839
H	6.8896322360	-5.4740226424	27.8896886244
O	7.4668166408	6.3192445962	32.5625965726
H	7.0121696340	6.9884411716	32.0091646614
H	6.7368044908	5.8852325980	33.0249811359
O	6.2648464357	3.3851527814	35.0659253102
H	6.5775264288	3.7022273631	35.9139979389
H	7.0690961617	3.1122768083	34.5629057756
O	7.0528236067	12.5314377170	27.1663968233
H	7.9586907526	12.5820491384	26.8340166140
H	6.5918096172	11.8427328100	26.6764000687
O	1.2450344957	-2.7992038463	26.9202626749
H	1.2539033858	-2.8516495120	27.8814813589
H	1.0835517126	-1.8621093381	26.6910307724
O	3.7281571742	6.1368408119	25.8854895556
H	3.7491308919	5.6508053438	25.0512886576
H	3.0757329954	5.6851021347	26.4431862821
O	15.2569836075	4.4123986363	30.9173128426
H	14.3730444592	4.1951267181	31.2357625747
H	15.5362838040	3.6291813876	30.3804576962
O	8.5603033238	8.4899508552	33.8209481495
H	8.2501779503	7.6385448398	33.4552230217
H	7.7671374468	9.0508808361	33.9127898043
O	7.4711590432	-2.1848991063	26.6779136563
H	7.4335157305	-3.1240122559	26.9105520908
H	6.6569123214	-1.8099274535	27.0696375811

O	1.1797754495	2.5224164441	28.8131113098
H	0.8790456676	1.6372397692	28.5948447851
H	2.1281081706	2.4311683994	29.0466472000
O	1.2543175492	-0.1777833530	26.2288070626
H	1.4093115318	-0.0044984230	25.2935602014
H	2.0865814868	0.0266948976	26.6784930540
O	10.2751489730	11.9985112064	34.3194001236
H	10.8244419112	12.6975853703	33.9529408085
H	9.9461738978	11.5242665602	33.5278269148
O	14.7827753560	10.2011752134	27.9389226150
H	14.4599296150	11.0962357735	27.8020449628
H	14.2434024222	9.6409626600	27.3312620010
O	2.0885057820	-2.8385510080	29.7553929407
H	2.9110830932	-3.2180861207	29.3940826336
H	2.2136524393	-2.7871522227	30.7101640781
O	15.4839860388	6.4517766258	32.7908990584
H	16.1487582579	7.0782659006	32.4295315394
H	15.5840960914	5.6612781506	32.2396933821
O	8.8800541640	9.8980277471	27.0995668573
H	8.2321900313	9.6272803491	26.4056897719
H	9.6233520042	9.2686191788	27.0070504111
O	13.5096832562	11.5012235753	31.7088480941
H	13.0891278801	10.8658071378	32.3257770510
H	14.2780089213	11.0130593317	31.3670743183
O	2.3135340606	7.2538185898	31.1634365270
H	1.9959143589	7.2987655613	32.0672359335
H	2.8172457521	6.4027147063	31.1122454603
O	4.5468715238	-0.6824691584	25.4576495972
H	4.2336195549	-1.6126093903	25.6054065533
H	5.3673077814	-0.7406427889	24.9460559116
O	11.3331429369	-2.6882020465	31.9235451894
H	10.6814396895	-3.4089927560	32.0042714412
H	11.9293662731	-2.7522560935	32.6693603528
O	4.9407452827	-3.2583685267	31.4617439393

H	4.2434738744	-2.6666244985	31.7883216733
H	5.7526135515	-3.0245769169	31.9462872387
O	1.6841776860	0.7855116324	31.7472100029
H	1.7164427970	0.5523583407	30.7824553552
H	0.7652250565	0.6807707328	31.9995556314
O	10.6460387862	4.1864545763	34.2642753097
H	11.4958041785	3.9482700911	33.8654237093
H	10.8039613431	5.0700079914	34.6512246452
O	7.2584509313	13.3041083392	29.7744487974
H	8.2260879629	13.3318847177	29.8347377276
H	7.0699706888	13.0835476696	28.8432662813
O	4.9921742808	10.5355207360	26.4749370000
H	4.6976434474	11.2234939309	27.0850104006
H	4.4980676323	9.7475648788	26.7709342015
O	15.6153743970	2.3953992247	29.2646659534
H	15.5614493894	2.6843198890	28.3432536473
H	14.8641313003	1.7865942711	29.4083347469
O	13.8817657954	2.7036259381	24.5853792423
H	13.1599515837	2.0580476510	24.8320253961
H	14.4081657350	2.2550410696	23.9241950332
O	8.4675447014	2.5753659157	33.8240907505
H	8.5438767192	2.4231573357	32.8599131154
H	9.2316117324	3.1244540628	34.0698512077
O	9.5731204301	2.2710862379	24.8744137935
H	9.1511818035	2.2837693221	24.0109182711
H	8.8403758841	2.4013997574	25.5249432612
O	11.6108463316	0.2009724662	27.6805568364
H	10.9966110972	-0.5217803602	27.8904120948
H	11.1121783347	1.0223993861	27.8765083630
O	4.4855314967	1.9983190464	22.3310241252
H	3.8762824863	1.4876570045	22.9304550746
H	4.1408785055	1.8974515093	21.4441403863
O	12.0093142106	0.9669790416	25.1083442292
H	11.1234398331	1.3355127856	24.9640121144

H	11.9918767421	0.5949449401	26.0118536011
O	11.6695077179	13.5088992044	32.1756774058
H	12.1077493579	14.3594776094	32.1986064286
H	12.3635702708	12.8471725309	32.0130800291
O	10.7472248784	9.4267843511	35.2332230401
H	10.6063279367	10.3900245301	35.2205206578
H	9.9303545228	9.0477426996	34.8633082102
O	9.3801688455	-1.2781608973	28.3486532991
H	8.8367635265	-1.6130142504	27.6075708945
H	8.9509455368	-0.4363830745	28.6145878058
O	6.7567095477	4.4006827910	24.3555153447
H	7.0917301716	3.7013451245	23.7647381806
H	5.7824820464	4.3748346512	24.2384090605
O	1.4459713593	7.3443075077	28.4983946105
H	1.5537861268	6.4245256909	28.1968842646
H	1.5692718390	7.3308274314	29.4585608476
O	8.8527073796	7.1844923623	24.1002308293
H	8.2819941084	7.9454229845	24.2699486607
H	9.7262886035	7.5147373503	23.8624479030
O	16.9986222234	8.5614016042	27.6175289514
H	16.7866028428	7.8966199130	28.2889566096
H	16.3277916082	9.2551412223	27.6992053597
O	17.6518503852	5.0138155740	27.3731942852
H	17.5882931245	5.6338167792	26.6310308172
H	17.0366791234	4.2937992719	27.1718513770
O	6.5718264413	10.3523125379	33.6559479000
H	6.7832167448	10.7018679617	32.7580461522
H	6.7037959357	11.0806790298	34.2630336962
O	9.1514687171	-4.3342239991	31.9801757021
H	9.0899034169	-5.2559815962	31.7308682187
H	8.7679860131	-3.8139691185	31.2234672949
O	11.6459794099	7.6884427911	24.2186047139
H	11.9809433363	6.7765546184	24.1790780043
H	12.3999785400	8.2411098095	24.4598246722

O	12.4592228246	11.6356372140	27.1208067450
H	12.4872066299	10.9223558553	26.4777452193
H	11.5689637957	12.0473413517	27.0284353631
O	4.5717624433	11.6120899431	29.0298141043
H	4.2403404233	10.9043477716	29.6068239092
H	4.9824344307	12.2791018188	29.5842731085
O	17.1300063901	7.9659279367	31.3093629605
H	17.0511106785	7.3835678330	30.5339943585
H	16.5631936781	8.7270271621	31.1145090176
O	17.0482662192	7.0273650604	25.4832499531
H	17.6807443350	7.3539763362	24.8442838304
H	17.0740300551	7.6595435455	26.2505443082

Trans-**12** QM/QM

S	7.6567055600	3.7164539878	29.5701778425
C	1.5181756618	4.5608119894	20.3786741014
H	1.6075808869	5.4793427815	20.9899712051
O	1.6206170105	4.4122543749	23.1958588070
O	8.3424053417	4.8630216976	29.1294108014
C	1.7731270406	3.0997832953	18.7373964618
H	2.1661908523	2.7179802980	17.8024117214
O	7.0367763763	2.9337492973	28.5855148198
C	0.9199892441	2.5644956216	19.6324055191
H	0.4050614217	1.6120493886	19.6467793552
O	8.3528393201	2.9755536231	30.5458503721
C	-0.0372608680	3.3208448667	21.8698440906
H	-0.6267681754	2.4085439950	21.7507383713
H	-0.7095783190	4.1843854388	21.9530892029
C	0.8583860148	3.2211359421	23.1270762860
H	0.1930636436	3.1106183786	23.9956581573
H	1.5090380312	2.3423738506	23.0664830858
C	2.3375084573	4.6388948566	24.3275010033
C	3.0736322117	5.8298617398	24.3948684822
H	3.0789688368	6.4887767656	23.5456392216

N	2.1324818388	4.3483457835	19.2198208059
C	3.0599638737	5.2764307321	18.5544306664
H	4.0705139072	5.1181130810	18.9385381493
H	2.7431665972	6.3016768387	18.7487603682
H	3.0359761829	5.0661442676	17.4812419231
N	0.7767031602	3.4910757890	20.6559038658
N	4.3537010005	5.6731938205	27.8880399562
N	4.7738319505	4.7215562470	28.5520016779
C	3.7505023769	6.1651198285	25.5479346422
H	4.2948901253	7.0986356835	25.5939259083
C	3.7204947423	5.3024325815	26.6459257232
C	2.9912147084	4.1265798518	26.5727724481
H	2.9106249886	3.4987042663	27.4448327005
C	2.3105493274	3.7788523323	25.4210715430
H	1.7669479542	2.8523376584	25.3968545470
C	5.1700673041	5.0337787511	29.9153836549
C	6.3077096167	4.4826801162	30.5062439491
C	6.4920591116	4.6653325617	31.8686345891
H	7.3589863398	4.2428516254	32.3516446151
C	5.5621599759	5.3530278148	32.6389239619
C	4.4531120222	5.9167623940	32.0310042401
C	4.2594671693	5.7625225906	30.6679514606
H	3.3708639232	6.1446982431	30.1848730828
H	3.7212805272	6.4478604981	32.6156546432
H	5.6898345922	5.4315206237	33.7121863433
O	8.3541701271	7.2024659362	30.7317188397
H	8.1574951268	7.2306405044	31.6994124588
H	8.2993245145	6.2938161367	30.4127699754
O	1.8196325322	1.6297698260	28.6389895865
H	1.5418247894	1.4619900564	27.7130453638
H	2.7852574311	1.7719538892	28.6057980354
O	5.9864084135	4.3650420647	23.7855996107
H	6.1244643941	5.2679561978	24.1534643110
H	6.8736332035	4.0065014670	23.6580693563

O	1.0068263359	7.5920141087	17.5035656130
H	1.2125415475	8.2019628473	18.2576873905
H	0.4911327884	6.8662927567	17.9145796418
O	8.7930160264	5.0948114220	34.4411147923
H	8.5099812331	4.1623467510	34.4951565205
H	9.4143334502	5.1170631774	33.7007392727
O	6.5284792844	9.0968558967	27.0030612055
H	7.0673543843	8.3144950511	27.2510943523
H	6.5780314437	9.6679763987	27.7749622865
O	2.4883615744	6.5621050420	15.4815264947
H	1.8003363088	6.0299312825	15.0730579233
H	2.0692507311	6.9583387749	16.2723391067
O	1.5210728985	-0.0037299740	21.0912794656
H	2.2195307847	0.6725252042	21.1087333611
H	1.4682714965	-0.3118155612	22.0155852372
O	10.5481463795	2.1329390575	33.2312003070
H	10.5593238646	1.5321880984	32.4731478301
H	10.6355798374	3.0267621781	32.8633112897
O	4.4753231051	2.0632596384	28.2326408450
H	4.6349870961	1.9676996460	27.2805027445
H	4.7246043092	2.9975498025	28.4260562085
O	6.8513896805	4.8127769575	26.7076455126
H	7.4387772311	4.4756674367	26.0036509429
H	6.7696926417	4.0863253746	27.3368819272
O	-0.9894876018	-0.8059994054	20.2296948884
H	-0.1053499955	-0.6212027858	20.5845082661
H	-1.5883703480	-0.1653348335	20.6430636856
O	0.4616575795	3.7211237357	29.5737225497
H	0.6438045298	3.8300089686	30.5274165186
H	0.9885914201	2.9381229181	29.2911125578
O	5.1934308149	1.8046005692	19.3156289298
H	5.9237650482	2.2560126802	19.7892755869
H	4.5397633487	1.6253161016	20.0075409400
O	2.2594598675	8.4024993566	29.9770071098

H	2.8590728975	8.6820304148	29.2648061995
H	1.8468811059	7.6032271661	29.6047284060
O	-3.8742777601	6.1750520888	20.6847227451
H	-4.1630626047	6.7672714345	21.3873913568
H	-2.9389462750	5.9653052986	20.9222554758
O	2.0784817266	-0.7870939260	18.4131916191
H	1.1706430273	-0.8146658820	18.0718535064
H	2.0000386268	-0.6233856643	19.3638020284
O	3.9548808292	8.3255268640	27.8283239261
H	4.8069929280	8.6770559175	27.5301185944
H	4.0949595140	7.3547206311	27.9528055443
O	9.6056309173	2.8698501540	27.9997619475
H	10.4827507510	2.7928843084	28.4159617219
H	9.7245996134	3.5511651165	27.3162692274
O	3.0737446857	7.0290620646	34.8001984986
H	3.3475537915	7.1860343730	35.7060399331
H	2.7496250304	6.1066203951	34.7656893019
O	4.8945580418	9.0404854586	17.1313369031
H	4.9207817976	8.0746379688	16.9517678051
H	4.3068107707	9.3874825924	16.4323268104
O	3.7340329111	0.6461581845	24.3474091186
H	4.0836688423	1.4757722834	24.7365490227
H	4.0246002140	-0.0011068068	25.0141174297
O	2.1095940521	7.1864377745	21.5022952676
H	1.9692298719	7.8390948518	22.2368903101
H	3.0600980897	7.1909140964	21.3148281303
O	-0.9931468391	2.5427003968	25.5518557704
H	-1.4616794478	1.8445557077	25.0633791404
H	-1.4393217153	2.7024172765	26.3958370671
O	7.7406859959	7.3856673472	33.2785298724
H	7.7960211475	6.5049616539	33.6822478135
H	6.8312577184	7.7258567022	33.4404813054
O	6.0964148308	8.3602862900	22.8882008178
H	6.8492942354	8.9138525960	23.1423136992

H	6.0187357001	7.7430295843	23.6365677957
O	6.1158783758	5.4225313825	35.7364739659
H	6.0478206564	6.3380880859	36.0961676174
H	7.0570065513	5.2430162248	35.6692148196
O	0.3198908882	6.2618917053	26.2154857749
H	0.1486594070	6.5488297256	25.2775190559
H	0.7705263303	5.4144434714	26.1337873339
O	1.4730852019	7.0239029656	32.3225295060
H	1.7893840989	7.3969217204	33.1513807353
H	1.7015476498	7.6502459468	31.6221180461
O	6.4827544979	5.9570224899	19.0963191196
H	6.1626822452	5.4079310690	18.3574806441
H	6.8817839635	6.7515018535	18.6903870528
O	-2.3116154638	3.1903196389	19.2528992064
H	-1.9765108668	3.2027601429	18.3443163399
H	-1.9441138348	3.9692782849	19.6850498124
O	4.3504461041	4.3765014830	21.7211257022
H	4.8232385210	4.3800809195	22.5795097315
H	4.3537275237	5.3037448389	21.4287525404
O	10.9818797156	5.3403724685	29.2820547522
H	10.0345895079	5.1215263734	29.1825449308
H	11.4615541227	4.5020787322	29.3553751853
O	1.9854715877	9.0723772402	23.3000493750
H	1.9566671422	8.9694386759	24.2707309306
H	2.7703118437	9.6221034439	23.0831489645
O	-1.2515080234	5.8961645789	21.1230693192
H	-0.8454086015	5.8248946034	20.2444418449
H	-1.2586545158	6.8646422616	21.3062919099
O	-2.2885185781	1.3279280932	21.3248300273
H	-2.8631377667	1.7932155075	21.9631826416
H	-2.3667756584	1.8460493216	20.5061538810
O	6.6904097189	0.1848598236	28.6333432068
H	6.6212589208	-0.0562614405	29.5650664554
H	6.5654009586	1.1510465977	28.6215028211

O	5.5333986741	2.2567720154	33.4151068473
H	6.4384177224	2.4683423455	33.6866288058
H	4.9914291291	2.5207692849	34.1961372433
O	0.5647859048	1.5003593141	16.2563577468
H	1.4950316301	1.2604071963	16.2982412598
H	0.0860405687	0.7816172052	16.6978828457
O	8.2844791039	7.1693214052	27.6102209920
H	8.1418252121	6.2793841366	27.9674338352
H	9.0135565814	7.5729480236	28.1426416277
O	4.8447300501	6.9798709179	20.8981411533
H	5.3947897445	7.4268239532	21.5670848541
H	5.4700093030	6.6185546630	20.2218151668
O	6.1680434188	8.3680432957	29.7134168026
H	5.5949976814	7.6003856120	29.6278658945
H	7.0085551384	8.0442821691	30.0953015345
O	1.5185273601	9.0088160125	19.6327371558
H	2.4514541989	9.3179036333	19.5884147309
H	1.5371823479	8.2996246655	20.3109037821
O	-0.1525605595	6.6900592347	23.6783829615
H	0.5311095035	6.2754219240	23.1415702880
H	-0.9575703254	6.1614326507	23.5484417823
O	6.7053274773	0.8985446542	31.3533352554
H	6.2630858234	1.2265276216	32.1539359565
H	7.3126632966	1.6070874391	31.0979222929
O	1.0446357870	0.9502872686	26.1925908095
H	0.2584259180	1.4930920795	25.9617116446
H	0.7626562537	0.0355125147	26.2234168206
O	4.1790541634	0.3308141920	30.3437451968
H	4.3695753961	0.8567675278	29.5476105086
H	4.9996589989	0.3332379987	30.8531709495
O	1.4928688759	4.2748492494	32.0529615496
H	2.3454602655	3.8667864073	31.7776013282
H	1.5711956233	5.2438504307	31.9679891659
O	-1.2897415437	0.3583086582	23.9733607165

H	-0.3625745301	0.0855838994	23.8895891230
H	-1.6203562248	0.4724179323	23.0780846046
O	10.0658980221	4.5567562038	31.9233247553
H	9.4511131247	3.9953685533	31.4179606865
H	10.5651805791	5.0684523417	31.2736341662
O	8.8794354555	7.7837669134	35.7573421116
H	9.1533431241	6.8613269316	35.7542466850
H	8.6579451630	7.9763012545	34.8308973897
O	5.7569733243	3.8318543859	17.4696237329
H	5.6585054898	3.0712376024	18.0617706442
H	4.9325891595	3.8700632843	16.9497077350
O	3.5495030916	3.4974078200	15.8862493025
H	3.8151752717	3.5384807839	14.9675804165
H	3.5456982546	2.5574438312	16.1432882619
O	-0.5890824708	-0.6024884252	17.6466012741
H	-0.8459155292	-0.6767242781	18.6020279898
H	-1.0564093937	-1.2990904698	17.1860352340
O	1.9619598070	8.6736944039	25.9795498215
H	2.6711269461	8.6292858873	26.6465754705
H	1.3493350305	7.9607138556	26.1891309586
O	1.6080076343	6.0410808108	28.7432430149
H	1.1579808188	5.2385466474	29.0720418254
H	1.1063159149	6.3221592818	27.9694064089
O	10.2980895210	1.1142724153	30.6615960617
H	9.5536438248	1.7432710737	30.6577361077
H	10.0902344101	0.5012781566	29.9213783969
O	-2.4365710668	5.1730242175	23.8078245202
H	-2.8675666997	4.3561709614	23.5089334547
H	-2.4511175762	5.1658569733	24.7909704514
O	6.5899539687	6.6906638739	24.8865478018
H	6.4904591382	6.2937320965	25.7654235307
H	7.5564303127	6.8186655761	24.7853951068
O	5.1804341887	2.4976362316	25.5724615993
H	5.3537909871	3.2815711941	25.0197365221

H	6.0464905315	2.0711697141	25.6980510875
O	6.7545623267	3.5702761199	20.6161749366
H	6.9831732021	4.3335491869	20.0705170441
H	5.9341054654	3.8282460358	21.0735163947
O	5.3237067063	8.3966542250	33.8340836436
H	5.0227148890	8.8936297023	33.0436916992
H	4.5740338397	7.8293195619	34.0738749653
O	-0.9367524343	3.7015792947	16.8287038063
H	-0.3552446651	2.9399785205	16.6345912577
H	-0.8499679951	4.3015038769	16.0744652923
O	7.4076489877	8.2337745478	17.9225070932
H	6.5973505269	8.7292178131	17.7109518946
H	7.8728596170	8.1238193365	17.0927797783
O	7.6439426899	1.5432865413	26.2556123811
H	7.6071112511	0.6078812149	26.4709786804
H	7.5967578392	2.0295360359	27.0978807391
O	4.4161041742	9.4370216551	31.5426541902
H	5.1000871231	9.2145131358	30.8861346649
H	3.5811082772	9.1429513063	31.1539772857
O	-0.5625334564	5.7284005149	18.5468392736
H	-1.3338998072	6.3520679603	18.4532717396
H	-0.6946098840	4.9906646430	17.9195859809
O	11.9500318339	2.7397065923	29.4479200224
H	12.7778863512	2.3167001488	29.2197574522
H	11.4488518351	2.1015799058	30.0229901861
O	7.8400893772	9.6487789871	24.6448015232
H	7.2948432761	9.4535513447	25.4357626391
H	7.9036195225	10.6025841412	24.5958787248
O	3.0985190259	9.3233943540	15.1185962455
H	2.1827867658	9.6199209371	15.2479081151
H	3.0278181117	8.3688282388	14.9782891247
O	4.3316512994	3.3035984928	35.5340118376
H	3.4934028028	3.6970817163	35.2479314911
H	4.9371241763	4.0483890992	35.6902983624

O	9.2184943578	7.2087009168	25.1509108265
H	9.1742847437	8.1112555446	24.8148052529
H	8.9048559348	7.2629773529	26.0858663760
O	-3.5169340150	7.5531721421	23.1338313986
H	-3.1304657625	6.6790948431	23.3689852238
H	-3.5101839733	8.0648980063	23.9430107578
O	1.9753952161	4.5259193414	34.6545444623
H	1.7802217907	4.3614681816	33.6963768049
H	1.1448882783	4.4166497322	35.1173047979
O	0.4056766139	9.6113788910	15.7863405211
H	0.5445216419	8.9031920756	16.4429620380
H	0.1421213670	10.3876335780	16.2813811951
O	8.1689858603	2.4622165324	34.3148357231
H	8.1017959698	1.9411717353	35.1162627994
H	9.0411071485	2.2319054291	33.9021109500
O	3.5606720927	1.7365822085	21.6567316911
H	3.7533858619	2.6869151263	21.7185050606
H	3.8312077241	1.3372870610	22.4963832713
O	3.6663505233	2.8554662439	31.3969009470
H	4.3132719779	2.7853601946	32.1160532680
H	3.5358287830	1.9461510460	31.0826167612
O	10.4463677680	4.9964458499	26.4068038804
H	10.8251980752	5.3721961314	27.2123329193
H	10.1536766640	5.7507539757	25.8728987906
O	4.1352991709	9.3603673821	19.6624457798
H	4.4340129321	9.3660811687	18.7279290810
H	4.4341667960	8.5079774014	20.0256202344
O	-2.4787606784	7.5069893687	18.7032549871
H	-2.1331845251	8.0745588785	19.4168580373
H	-3.2288713341	7.0552698399	19.1223188035
O	-3.8235743530	2.9442736384	22.8851842238
H	-4.5360964278	2.6425571111	23.4479631797
H	-4.2483623675	3.2547708428	22.0337358351
O	4.1799474770	10.2566245893	22.3506367352

H	4.8783970106	9.6239478318	22.5911375241
H	4.1335922876	10.2377974708	21.3854343137
O	5.0503416273	6.4651981932	16.4706941219
H	5.7480189352	6.0961997774	15.9117793745
H	4.2135868949	6.3529118361	15.9952987331
O	3.5113645245	1.0926737761	17.1439164372
H	4.2131448367	1.2705008466	17.7883204917
H	3.0311228026	0.3329327654	17.5394561305
O	7.0927765516	4.9137398153	15.3702466189
H	8.0485353722	4.9047864689	15.4163473767
H	6.7734050187	4.3763656348	16.1191742445
O	2.5990989463	-1.0564242642	28.5460191442
H	2.0962027910	-0.2289995096	28.6217265683
H	3.1738612038	-1.0323300805	29.3226859023
O	-1.7679861768	3.2247664677	28.1546439731
H	-2.4151094465	2.9452887378	28.8022744311
H	-0.9549009248	3.4476476393	28.6630395931
O	1.4041876826	-0.4958817222	23.7478281510
H	1.5702997160	-1.3939755483	24.0372413882
H	2.1804600603	0.0326375354	24.0566507682
O	10.3156252761	8.0332617193	29.0787740475
H	9.7546926779	8.0441926185	29.8716941835
H	10.8405696527	7.2256461578	29.1545156041
O	-0.4014840472	6.0433351909	15.3075595904
H	-0.9412383386	6.4787790591	14.6471705826
H	-0.1959134564	6.7163364509	15.9695012203
O	4.5754260899	-0.5822660873	26.6850317419
H	3.8181639972	-0.8104046608	27.2594922159
H	5.2953622482	-0.3753288328	27.2936943286
O	-2.2879510432	5.4034031275	26.4719935251
H	-1.4229199452	5.8493624452	26.4970377562
H	-2.2014425099	4.6508172971	27.0755742603
O	9.4249618496	0.1015769050	28.3544583860
H	8.4738292161	-0.0678922093	28.4734374677

H	9.4821769710	1.0005737281	27.9961240233
O	-1.7474702871	8.4732388737	21.1827024439
H	-2.4065816369	8.4157306652	21.8908801893
H	-1.1041376317	9.1798839442	21.4376747710
O	-4.7668404391	3.6716600129	20.5678202913
H	-4.6141524380	4.6411940317	20.5785890935
H	-4.1273658722	3.3314567843	19.9265052601
O	6.2186666883	7.9901492665	36.4369039320
H	5.9118356614	8.4031412609	35.6151781890
H	7.1914678008	8.0545106319	36.4057133127
O	8.4822724886	3.6758138172	24.7801846865
H	9.3535671813	3.9885937032	25.0411303834
H	8.3753804413	2.7833962144	25.1421936010
O	0.1067707534	10.2534267779	21.7097907080
H	0.6007775715	10.1119802780	20.8878357025
H	0.6765287938	9.8995178261	22.4189188953

Cis-**14** QM/QM

C	1.5152019085	4.8242228972	19.6782792050
H	1.0126439864	5.8079446890	19.6006117319
O	1.0722177217	4.7460477237	23.2786882180
C	2.9665079424	3.1918922521	19.3133707140
H	3.8099073317	2.6926824010	18.8434566950
C	2.0933223191	2.7664002698	20.2479218656
H	2.0111219045	1.8179696450	20.7703644097
C	0.0345189109	3.7319641265	21.3726553798
H	-0.5796838870	2.8708137570	21.0787957132
H	-0.5326859846	4.6564666803	21.2671509982
C	0.4996534961	3.5333376060	22.8369898691
H	-0.3742344888	3.2668860212	23.4444956974
H	1.2047370429	2.6954866222	22.8743407951
C	1.9425858190	4.7383161621	24.3190555979
C	2.5112846901	5.9761264529	24.6468227660
H	2.1912710561	6.8586785020	24.1150818567

N	2.5874627123	4.4835171500	18.9700347852
C	3.3155164249	5.3851127308	18.0646238850
H	4.0613009351	5.9482878679	18.6353956165
H	2.6068517339	6.0825653107	17.6050490144
H	3.8091551885	4.7811353438	17.2995841717
N	1.1900968758	3.8012152369	20.4608919727
N	5.0477676691	4.9810486841	27.1444175257
N	5.0639354340	4.6464742771	28.3208198865
C	3.4902336628	6.0454286268	25.6090238035
H	3.9646938280	6.9841258130	25.8487000918
C	3.8775702452	4.8985227661	26.2993613053
C	3.2545641845	3.6907359852	26.0408471252
H	3.5560968515	2.8082044584	26.5869810947
C	2.2958925964	3.6018712144	25.0385244375
H	1.8550799955	2.6501394467	24.8034464262
C	3.8949177610	4.2805535210	29.1132494567
C	2.7619622552	5.0949359638	29.0910442390
H	2.6937995673	5.9254502887	28.4183819156
C	1.7916913849	4.8465458901	30.0540690194
H	0.9313617016	5.4999391063	30.1094786142
C	1.9152956523	3.8262780505	30.9752796664
H	1.1733173450	3.7293736784	31.7461462780
C	3.0540797501	2.9409594095	30.9885167345
C	4.0554059136	3.2421260954	29.9942585763
H	4.9597003490	2.6634327933	29.9759800378
O	3.1813283239	1.9973691818	31.8262418002
O	1.5722798504	8.9449607418	29.9649122749
H	1.0608411287	8.1881910330	30.3613651388
H	0.9036732967	9.6335256926	29.7680391635
O	-0.2537844468	1.2698725103	26.0467130762
H	0.2965448076	1.9892348008	26.3849576973
H	0.0467612703	0.4368183038	26.4486005249
O	0.5752789638	7.5228168389	19.1216209232
H	0.6123583295	8.1258562335	19.8887763750

H	-0.3671231591	7.3830727467	18.8920054333
O	2.1511794094	7.9688171207	27.6213623799
H	1.9946882747	8.3796400107	28.5138837835
H	1.3154135899	7.5105864552	27.4180059032
O	3.3600669111	8.6183127615	18.6560359748
H	2.7917546014	7.8947875789	18.9430841187
H	2.7704071782	9.2454672945	18.2185037092
O	5.9369732037	2.3810416749	26.7763932771
H	6.8420844372	2.6976914834	26.9782721658
H	5.8585539018	2.3744341037	25.8073210335
O	6.2780352259	8.1308472771	25.8217814061
H	5.8358039903	8.1153321188	26.6908518620
H	6.8002557686	7.3069963937	25.8052269403
O	5.8815585063	6.7069030596	19.8666695840
H	6.4466078286	6.9943569561	20.6257722275
H	5.9176862088	7.4415549801	19.2045935317
O	-1.2670480862	4.2987102210	25.3958170735
H	-0.9259133974	5.1111769618	24.9754685144
H	-0.6305693981	4.0544968689	26.0914339511
O	6.9148975366	4.7221399694	18.4704549518
H	6.9157719909	3.9676010352	19.0702206679
H	6.5572556903	5.4798477458	19.0032247776
O	-0.3853435148	6.1346666041	33.3721265470
H	-0.0215098643	6.4204975879	34.2379870182
H	-0.3007883100	5.1571412445	33.3906304948
O	0.3480780194	7.3643520976	22.1317657987
H	1.1657378614	6.8895713895	21.8665269682
H	0.6507279912	8.2621973975	22.3274335720
O	-1.5408064841	4.4294393039	31.0582735453
H	-2.0713984989	5.0506425980	31.5740575689
H	-1.6725050958	3.5456010336	31.4200335228
O	2.5937456652	-0.5293567609	31.3782201451
H	3.4098208907	-1.0216909077	31.4859648127
H	2.8168484567	0.4139826124	31.5777288903

O	-0.7520018436	6.7591909765	24.4607254522
H	-1.7105964002	6.7503958545	24.2254476281
H	-0.2565031938	6.8144685428	23.6127177968
O	2.1106629177	0.8760222531	23.5817900468
H	2.5745663724	0.5425878347	24.3668969259
H	1.3110977214	0.3163834481	23.4609285962
O	-3.3633202899	6.5428174247	23.9766288634
H	-3.3706959371	5.8551517675	23.2946949926
H	-3.6990655327	6.1362669223	24.7906548130
O	-0.0541919405	7.1256206760	30.8602594040
H	-0.9246922945	7.5387305810	30.7305281029
H	-0.0414092320	6.7929296230	31.7761855136
O	-1.8500371931	0.6611871805	23.9335551585
H	-2.6052213030	1.2490420186	24.1236262665
H	-1.2053901746	0.8957418373	24.6235319509
O	6.3030031221	2.5445068992	20.3985714054
H	5.6320751545	1.8266773526	20.5027435195
H	6.8792651232	2.4649780467	21.1613768716
O	5.2122887702	0.6607544879	32.7050333403
H	4.5118502660	1.2767440807	32.3456984745
H	4.9403186281	0.4590145895	33.6011563571
O	3.8622501133	-0.1669657666	25.4473516800
H	4.6622284906	0.1074348144	24.9922195208
H	4.0279093337	-0.0113240399	26.3993054459
O	-1.3347703192	1.0158948626	21.2845047982
H	-0.6523077626	0.3783936996	21.0485787544
H	-1.5284668049	0.8685473956	22.2300114857
O	4.7923623212	7.6964648140	23.5188278492
H	5.2726865487	8.0280371714	24.2975889685
H	3.8890041585	8.0484426526	23.5765929482
O	4.2431462870	8.4450589418	30.6366524063
H	3.3547485284	8.7968431293	30.4671357626
H	4.1127048599	7.7089517880	31.2545420506
O	5.3635032377	2.5047857071	24.0702147800

H	4.4568593992	2.3496119892	23.7921315741
H	5.5179716792	3.4588920759	23.9460139409
O	2.7277310765	6.6508805898	21.2203865060
H	3.1856209107	7.5044149828	21.3405812941
H	3.3054629889	5.9714594271	21.5769902985
O	2.4520446925	5.3221428218	14.5196972698
H	3.2468093455	5.6312974991	14.0751666238
H	1.7267064922	5.2128623717	13.8722508192
O	-0.5533220766	4.7518076649	16.0114369833
H	0.3549781601	4.8029369573	16.3217542278
H	-1.0767208753	4.4487244800	16.7754903395
O	2.0140285282	2.0085533913	34.1561680207
H	2.6975530456	2.4145041495	34.6962014881
H	2.3940956605	2.0318682965	33.2380410693
O	-1.4872756997	4.1520362806	18.5364364761
H	-0.8659035949	3.4039328416	18.5823355490
H	-2.2989764050	3.7820803412	18.9160068981
O	7.2384145988	5.5549854566	25.8096175640
H	6.9344580589	5.3209148022	24.9236506325
H	6.4385762544	5.3840063663	26.3880335093
O	1.1447923297	-0.1692984697	20.2710062678
H	1.8172612082	-0.3190314938	19.5917586327
H	1.4101171605	-0.7154454012	21.0224543650
O	-1.7610422947	4.8029907864	28.4680541277
H	-1.0648228267	5.3694130420	28.0895785354
H	-1.5150629708	4.6149639429	29.3992360644
O	2.5048287985	9.9924212335	25.9152849266
H	2.4806897745	9.2374290992	26.5422899977
H	1.7095083097	10.5154000836	26.1475957952
O	5.3327138751	1.6227633689	17.7482780238
H	6.0114892195	1.7289707358	18.4206015658
H	5.4947702248	2.2949858035	17.0730171857
O	4.8479548868	7.6445469473	28.1175870809
H	3.9267975861	7.7974158168	27.8581031174

H	4.8732802153	7.9763083609	29.0366030920
O	7.2322175061	4.7930250179	29.6555328783
H	6.3692851887	4.7496035781	29.0921012662
H	7.4983746283	5.7129080729	29.6451800554
O	1.6374886145	7.2215215893	16.2954490777
H	1.8430996327	6.5522942395	15.6205974189
H	0.6938237656	7.4590239648	16.1736045530
O	-1.3213055415	1.6997110422	31.8546649170
H	-0.8515912753	0.9743234103	32.3143817672
H	-1.0032034791	1.6707744325	30.9378965617
O	4.7185964332	4.6518513808	21.1444337123
H	5.1865393391	3.8604577140	20.8239975239
H	5.0725809752	5.4071370111	20.6384094265
O	-3.0686087122	4.7966839174	21.8767625235
H	-2.7947002184	5.6159895325	21.4242637921
H	-3.0751645929	4.0781117939	21.2140207352
O	8.3006961803	3.5800452294	27.3429089979
H	8.1034329461	4.0444384547	28.1727027216
H	8.1554243723	4.2630599878	26.6593329530
O	-0.2467138921	6.7435727378	27.1660554981
H	-0.2481160823	6.6736490590	26.1975924133
H	-1.0021567097	7.3252132089	27.3649858816
O	0.5366295295	3.5963555510	27.3243014470
H	1.1700581628	4.2567960484	27.6246095135
H	0.2252366627	3.1243521179	28.1291992230
O	1.9621560352	0.2643115244	28.9054384983
H	2.8430213270	0.4823077727	28.5323261645
H	2.1406030123	-0.1601331448	29.7699841550
O	3.1228581107	5.7261309301	34.8383230509
H	3.2833896025	6.0988454303	33.9553076303
H	3.5184414121	4.8424933238	34.8125105790
O	-3.4012419656	2.6689791849	24.8456098088
H	-2.6744149163	3.3023677508	24.7333329526
H	-3.3539194691	2.4461615228	25.7983201823

O	2.2542012633	8.8902098557	23.5075078431
H	2.6572255350	9.5487217951	22.9341656451
H	2.2376446993	9.2968627291	24.4018642514
O	5.9482165031	4.3649484188	32.1297176327
H	6.3062983531	4.6135763993	31.2621431688
H	6.5713025339	3.6739222260	32.4492219070
O	-2.1275036255	7.2246428107	21.1037137759
H	-2.5706898840	7.9505093374	21.5913280002
H	-1.2018383322	7.2567872964	21.4194294251
O	-0.0467710721	1.8391375523	18.5549447383
H	0.1971952306	1.1795908947	19.2169806051
H	0.5034745174	1.6489323070	17.7749859240
O	-0.2339032348	3.4793039254	33.7090707680
H	-0.7217088925	2.8575507745	33.1449575835
H	0.5651943940	3.0007768505	33.9967676444
O	0.2653968633	-0.9894260353	23.1228780455
H	0.6895877312	-1.5734421807	23.7790846278
H	-0.6140929255	-0.7684156409	23.4488859512
O	3.9523244902	9.1207715939	21.2094367972
H	4.1547166278	9.0460386507	20.2604071021
H	4.7501864898	8.9319291931	21.7170925677
O	3.1448455151	2.8034293157	15.2186164321
H	2.8084660909	3.6888422531	14.9848061724
H	4.0211439330	2.9622200339	15.5922416894
O	4.5619552247	3.3567751686	34.3712589894
H	5.3562247710	3.3610542292	34.9084951130
H	4.8450317716	3.6728199368	33.4987282805
O	1.1980540571	9.6704492320	20.6865427954
H	1.1190870724	10.2177614579	19.9001728944
H	2.1448949123	9.5637301431	20.8540856114
O	-1.9409335397	6.8917425085	18.4947587158
H	-2.2383128723	7.0961408022	19.4053348634
H	-1.8580101616	5.9259596266	18.4550661661
O	7.4402100557	2.2387505240	32.7609435808

H	6.7037142351	1.5986203866	32.7985270310
H	7.8776642805	2.0766929885	31.9156637962
O	-2.5644934764	2.3346064412	27.3653767013
H	-1.7694444348	1.8912246165	27.0316559311
H	-2.2537156078	3.1477649311	27.7954917410
O	0.0960399928	2.1132042024	29.4855385882
H	0.5725836346	2.7453229750	30.0545816755
H	0.7433819412	1.4143919763	29.2652276981
O	3.1635856349	-0.0373558538	18.2195044859
H	4.0012344866	0.3139613727	17.8852084672
H	2.4974199695	0.3270066220	17.6117533849
O	3.1462168741	-1.1904109690	22.1512691618
H	2.9453369108	-1.9361888027	22.7192491973
H	2.8422827381	-0.3929596068	22.6307105224
O	6.0637154352	8.4156695600	17.8842504343
H	5.1638231648	8.5176210740	17.5365634770
H	6.5511284123	7.8857561415	17.2366950766
O	-1.4676312049	9.3238344132	25.1339397287
H	-0.9589166296	8.5410082766	24.8574845390
H	-2.0586290508	8.9925333548	25.8290816775
O	4.4405424910	0.5653812902	27.9808414358
H	4.9589756396	0.2089563496	28.7315355063
H	5.0089994754	1.2794194163	27.6188050598
O	-2.4972784513	8.3401442762	30.5590922752
H	-2.9613087213	7.6606256161	30.0155388105
H	-2.7294774245	8.1019970069	31.4654669358
O	-2.7094883577	7.9064716950	27.1581804091
H	-3.2667012653	8.1566645417	27.8961725149
H	-3.0515000777	7.0386904896	26.8587685772
O	-1.0025633349	7.5331345884	15.9931277445
H	-1.0757266372	6.6113105633	15.7045267330
H	-1.4678814057	7.5518660675	16.8432407316
O	0.4783597958	-1.0978979134	27.2158850615
H	-0.2316718844	-1.5018800673	27.7155379948

H	1.0783372436	-0.6624008428	27.8692430948
O	5.7546046289	5.2035690493	23.4952596245
H	5.4008390460	4.9513121703	22.6132919169
H	5.2799252699	6.0410921606	23.6876417814
O	5.4590099950	3.9620475278	16.2794632961
H	5.4075193533	4.7107889995	15.6526778116
H	5.9990219582	4.2857970121	17.0256970889
O	3.7917931344	6.1320988787	32.1834498911
H	3.1367144564	5.6684739953	31.6447500977
H	4.5932180415	5.5855015597	32.1700769816
O	6.9341666804	9.5995267800	23.4975238011
H	6.0743517134	10.0479694179	23.5405699060
H	7.0495363434	9.2287179106	24.3825194220
O	-5.2801854677	3.7344509707	23.1766664172
H	-4.7417030799	3.3228237851	23.8749541501
H	-4.6477482410	4.2488196734	22.6507866399
O	7.8413252819	2.0864737309	29.9418972544
H	8.4040652128	1.9875120375	29.1694691140
H	7.5788379486	3.0221037480	29.9424057954
O	-2.8778589780	6.4288199955	32.6187906044
H	-3.4477302904	6.2071091741	33.3547679287
H	-1.9510172986	6.4088714119	32.9774135171
O	1.4811869391	1.1902930283	16.4284977800
H	1.0250769367	0.7158612405	15.7334518845
H	2.1161995520	1.8082015838	15.9851165485
O	-2.6621506025	1.4728941950	17.5155005621
H	-3.2103768871	1.7548688658	18.2550036417
H	-1.7549716579	1.4734232293	17.8540735681
O	1.0232580946	9.8115680455	17.7132446595
H	0.8092525817	8.9130319555	18.0428971808
H	0.5130637115	9.9227144571	16.9112026875
O	4.5084245929	0.6197569224	20.6168285256
H	4.0166262365	0.4314290167	19.8045462269
H	4.2876822373	-0.1086444555	21.2117814756

O	0.1190811220	10.9526478281	26.6699751174
H	-0.4667403365	10.4915516545	26.0408712720
H	-0.1472865665	10.6563172423	27.5488020589
O	-5.3604179205	1.7370275452	21.4123276585
H	-5.1981844585	0.9475592236	21.9305108677
H	-5.4963989680	2.4707111778	22.0589828338
O	-0.8051038381	10.2184746074	29.3403886847
H	-1.4209879639	9.5549891200	29.6973274280
H	-1.1095751751	11.0704058248	29.6565055999
O	4.4562207471	10.7953657784	24.1141132796
H	3.8986010941	10.5091229598	24.8590169732
H	4.5977951655	11.7339631320	24.2322049861
O	5.9840240164	0.0073007846	30.1074540656
H	6.6939526293	0.6601401658	29.9908694107
H	5.7335382319	0.0746924250	31.0389401628
O	-3.5110226798	5.4085418037	26.3891336788
H	-2.6752368219	4.9339409585	26.1679381974
H	-4.0100548946	4.8338766346	26.9698315166
O	5.3051157308	6.2520040212	14.8071137042
H	4.8021382837	6.9896455473	15.2048954554
H	6.2224870730	6.4313166583	15.0468698798
O	7.2750289260	7.2978741024	22.0259553722
H	7.1920148620	8.1663769589	22.4682777448
H	6.9529440780	6.6497084119	22.6652414847
O	4.0007245525	8.3935128969	15.8920299447
H	3.1189919428	8.0528042496	16.1625833491
H	3.8533246607	9.2352649153	15.4622934710
O	0.7009123761	6.4061135748	35.7817284447
H	1.6266175141	6.2493332392	35.4967720139
H	0.4395127390	5.6171791382	36.2599270170
O	-3.1065642237	9.0245614084	22.8495006267
H	-3.5085190332	8.2749267927	23.3131096002
H	-2.4864926191	9.4064220045	23.4897701677
O	7.6103740788	6.5377224217	16.4535953447

H	8.5261592416	6.8141299492	16.4958364103
H	7.4969837156	5.8492154743	17.1295519579
O	1.8671474948	-2.0870875993	25.0205153726
H	2.6776774955	-1.5692842483	25.1391311883
H	1.3420878236	-1.9048380722	25.8146660827
O	0.3757786449	-0.0288098522	33.0865958290
H	1.0358427877	-0.3910192685	32.4798889200
H	0.8599024136	0.5692195804	33.6737483527
O	-3.1983846465	2.6762290687	20.1515746487
H	-4.0609912793	2.3446378450	20.4851862659
H	-2.5440201172	2.0327595848	20.4800108492
O	-3.7395708424	6.3562428693	29.3037500892
H	-4.0957126435	5.8697797455	30.0479454877
H	-3.0312525530	5.7818271182	28.9149481915
O	0.1189121456	4.7922474312	13.3296912870
H	-0.3072925909	4.7422590335	14.2026612236
H	-0.1359473262	3.9998864925	12.8562351108

Trans-**14** QM/QM

C	1.0179411465	5.3228853595	20.7033404985
H	0.7937674744	6.3940796908	20.8723463101
O	0.4270958824	5.6873141533	23.6092480615
C	2.0444873712	3.4622604941	20.0859768790
H	2.8208415664	2.8671143158	19.6073892268
C	0.9666610119	3.1141779980	20.8182835611
H	0.5819539260	2.1447165958	21.1127086670
C	-0.8370079668	4.3857834160	22.0712878056
H	-1.4518129670	3.4921720653	21.9252745667
H	-1.4081313324	5.2678935174	21.7803539636
C	-0.3354505296	4.4916999109	23.5343682226
H	-1.1876787803	4.5150174409	24.2234900786
H	0.2764854432	3.6116884201	23.7627437741
C	1.5283724198	5.6875747932	24.4095156192
C	2.5652805938	6.5543145791	24.0619624688

H	2.4266565065	7.2006767199	23.2135897931
N	2.0520606129	4.8471837184	20.0165716726
C	3.0089505651	5.6346589375	19.2249868154
H	4.0213909291	5.3275741343	19.4913321399
H	2.8566935451	6.6995118516	19.4253117674
H	2.8208555549	5.4489124338	18.1652389810
N	0.3339139296	4.2902671238	21.1873389655
N	5.0566271177	5.7107833059	26.7151792811
N	4.7936073513	5.8582251071	27.9044833664
C	3.7411864282	6.5580534011	24.7885867861
H	4.5647869553	7.2012352376	24.4998881830
C	3.8546150244	5.7283180375	25.8967578978
C	2.8131017436	4.8867941654	26.2594894419
H	2.9521910850	4.2315992837	27.1092398778
C	1.6490718058	4.8503725892	25.5149263394
H	0.8520503362	4.1818280769	25.7893601901
C	5.8973530394	5.7523879674	28.8472606454
C	7.1541567916	6.2576071976	28.5619138010
H	7.3478666270	6.7307721708	27.6190730373
C	8.1262082027	6.1207943813	29.5576842826
H	9.1198959795	6.4964521684	29.3764015356
C	7.8658726325	5.4893554144	30.7492518889
H	8.6575417073	5.3751781177	31.4641122484
C	6.5698250306	4.9407000378	31.0636496883
C	5.5786622150	5.1497934726	30.0502196988
H	4.5825074114	4.7751705507	30.1910233767
O	6.3279390633	4.3110924926	32.1507173207
O	4.7038770672	0.9556564231	26.5695425614
H	4.6017773693	1.8577130130	26.9776957292
H	5.3971396651	0.5128830771	27.0914983059
O	3.4708242279	3.7610339637	22.9408103896
H	4.3790053820	3.5082558761	23.2260356023
H	2.8732360939	3.4677582428	23.6375366589
O	1.5516272272	2.0188701944	24.2190487084

H	2.0217581036	1.5740975933	23.4786015998
H	1.8940775012	1.5756222388	25.0322938479
O	4.5547518110	5.9577051974	21.8102648305
H	5.1379248897	6.5098127048	22.3951801591
H	4.0240168458	5.3713636671	22.3753822688
O	1.3361881382	2.2035438218	28.4256434602
H	1.8116530948	1.8643732809	29.1954732681
H	1.3518870749	3.1787762325	28.5187698757
O	6.9888898724	5.8846370235	19.0744865215
H	6.7348743744	5.2016040964	19.7380000245
H	6.4142858749	6.6675298945	19.2206381929
O	-1.6351235540	7.5023830057	23.4681099911
H	-0.8613013955	6.9102312404	23.4993025822
H	-1.2902789727	8.3541634412	23.7633718514
O	5.2881319433	5.2860660725	34.6479396991
H	5.9104877293	5.9656750014	34.9370681643
H	5.5104889598	5.0817713082	33.7280321833
O	0.0025702361	6.7630493859	17.9284347554
H	-0.8000632001	6.4317701936	18.3687409882
H	0.4891461038	5.9312891457	17.7548307415
O	1.1617054909	7.0391413092	15.3798236749
H	0.8588246185	6.2347589074	14.9392686776
H	0.6296225767	7.1263557383	16.1826873166
O	-1.1386642845	2.2143561314	27.2585224242
H	-1.0754379487	1.6120800847	26.5012574241
H	-0.2851497025	2.1367762052	27.7192926263
O	5.7915202286	8.3033788531	26.4986106424
H	5.0733964952	8.6899081570	25.9671008490
H	5.5969597141	8.6207757633	27.3995228143
O	4.7838170813	2.2079030389	19.4128107292
H	5.3105233841	2.0642968890	18.6070319719
H	4.5062782735	1.3175515915	19.6838639816
O	6.7593062553	1.6354653912	31.8162157391
H	7.6969438474	1.5309396809	32.0502802392

H	6.5901883053	2.5967730682	31.8968771280
O	0.9364302083	4.2916866930	17.3011798503
H	0.2248977619	3.7123446529	17.6102392546
H	0.8413794243	4.3410394874	16.3158283132
O	3.3637654585	1.7081071858	30.2486720091
H	3.5293655745	2.2413595193	31.0582897449
H	3.9796333652	0.9740706124	30.2742087438
O	6.0468460474	7.6674946619	23.1147970912
H	6.3671360432	8.2819059996	22.4275271489
H	6.8457015619	7.1400850910	23.3367143555
O	3.2696404859	2.9729879440	16.9202197944
H	2.4274101771	3.3695278072	17.2161446855
H	3.9919631473	3.4906262106	17.3122160296
O	2.0681961896	0.5760636173	26.3617288504
H	3.0411155333	0.5084625632	26.4208695258
H	1.8049546659	1.1336172993	27.1145180956
O	1.0745225167	-0.0462181189	20.9158556026
H	1.2837613512	-0.9255410121	20.5165591043
H	1.8719300367	0.2102619161	21.4142018504
O	0.8768491598	8.1533873436	21.4347874260
H	0.2795854824	8.5726628336	20.7816373956
H	0.6247964149	8.5116496866	22.3046607948
O	-2.1943076215	1.8561519591	20.7269877233
H	-1.9650305792	1.2257428294	21.4478867602
H	-3.1170387720	2.1414649453	20.8562538657
O	5.2845431941	4.8350956250	17.2450568712
H	4.8057976430	5.5695266039	16.8183599119
H	5.9915196773	5.2252889495	17.7935321113
O	-2.2186215815	4.4089161010	26.0246480053
H	-1.9121643239	3.7010137571	26.6193388629
H	-1.7543263019	5.2113141829	26.3196764780
O	5.5232541812	8.1269042873	19.3148565991
H	5.1912421916	8.4457747828	18.4465493103
H	4.7655526127	8.2215450031	19.9213142417

O	-3.7752168293	5.3399561258	21.1079929220
H	-3.7614638967	5.3957785267	22.0785480633
H	-4.2059396406	4.4948858766	20.9052017989
O	7.7891868546	6.6959741801	34.7343159961
H	7.5393594715	7.2022425502	33.9385770588
H	8.0042185323	5.7857998532	34.4411588518
O	4.1884639102	10.2671898521	22.9345857449
H	4.1056091710	9.5103220447	22.3122414682
H	5.1500542462	10.5058005375	22.9873158279
O	8.7502652523	9.1732494229	28.0778116620
H	7.9600019941	8.9114827612	28.5566188121
H	9.4451521144	9.3632035575	28.7487408368
O	6.5405886942	1.4901521615	29.0112021355
H	7.3646423384	1.9550100198	28.7668359453
H	6.5892701138	1.3981816621	29.9755104745
O	9.9902853455	7.8232967989	32.0922469856
H	10.3233733679	6.9717709124	31.7381353132
H	10.2864001008	7.8574657754	33.0161429137
O	7.4426138507	8.2927107916	32.5423131862
H	8.3497602617	8.0706240362	32.2113006555
H	7.4599056557	9.2249322239	32.7639851156
O	3.5606673829	8.3688876080	21.1871852169
H	3.8152929268	7.4560412232	21.4315973548
H	2.5846206593	8.3852325131	21.1343507198
O	10.1392918362	7.0335005743	27.0786661088
H	9.5840837186	7.7824133945	27.3749755685
H	10.8753959501	6.9820577834	27.7055985733
O	8.2460499438	4.1272348993	33.8977887314
H	7.7453567155	3.6794123417	34.6291933543
H	7.5531691970	4.2251686855	33.1808705250
O	7.0691288112	4.7546806355	25.2972115200
H	6.3877241192	5.2288868330	25.8507452581
H	7.4352799575	5.3860339082	24.6558701887
O	1.9059881605	4.8076070420	31.5746409167

H	1.0209778280	4.4797135656	31.8405979104
H	2.5595347017	4.2238031895	31.9918969552
O	-2.4998998716	-0.5123248969	19.3344453856
H	-2.0920370650	-1.1393785447	19.9349192351
H	-2.4541545367	0.3493814172	19.7884910995
O	5.1032743899	7.8178845810	31.2133049372
H	5.2689368142	6.9510205686	30.8017807689
H	5.9061637588	8.0237153777	31.7185374621
O	8.6268266369	3.1734215001	28.7325019784
H	8.9914459134	3.5155670417	27.8692724407
H	8.2210551120	3.9516884279	29.1370875779
O	-2.6904720445	2.3632226389	24.1135990166
H	-2.2096098964	1.5751607392	24.3839639081
H	-2.5602644755	3.0164523609	24.8135361185
O	1.2432851731	4.8086119890	28.9677082902
H	1.6083704416	5.6374673006	28.5947624762
H	1.5160536885	4.8056223159	29.9091830955
O	1.4360937393	1.1414385834	18.4819959551
H	1.4024335643	0.7502715912	19.3757205943
H	0.6515075752	1.7118101723	18.4310461230
O	-3.6534458215	5.6319517243	23.8987969126
H	-3.0974266023	6.4243168858	23.8289564488
H	-3.3491704186	5.1922606041	24.7044515924
O	-0.9775568714	2.5229015036	18.3665523300
H	-1.3621319482	2.2999430822	19.2339056340
H	-1.2269344470	1.7835726821	17.7725561755
O	10.6422932491	3.0525845438	30.5164502648
H	9.9992768155	3.0131129321	29.7802461035
H	10.2223281013	2.5874639388	31.2556440864
O	5.1450706264	0.6295679238	23.8942860594
H	5.6243268097	1.4559880451	23.7096285411
H	4.9413236761	0.6777642577	24.8464882637
O	2.5510067797	7.0972451468	28.4783955277
H	2.4523882733	7.2932698419	29.4413423950

H	3.3879825883	6.5585719616	28.3633033307
O	10.7086940285	9.2959022408	29.8811017065
H	11.3324861347	8.6462297566	29.5257792811
H	10.4329170082	8.9298498707	30.7371721753
O	3.7494039334	0.3087018929	16.7701473857
H	3.4232171122	1.2236505101	16.8862921400
H	4.6933197384	0.4268647239	16.6054168051
O	1.4758921098	8.4059622973	26.3118238584
H	1.7891081429	7.9306968637	27.1000434479
H	2.2856751228	8.8164618928	25.9599974232
O	-0.6719091760	6.5545686454	26.7816244642
H	-0.0047348702	7.1721220014	26.4525557367
H	-0.6774492740	6.6411542846	27.7366778032
O	-0.8501525577	9.1021419159	19.5882925441
H	-1.6536984537	8.7913018182	20.0628641323
H	-0.7802954813	8.5495317603	18.8039142901
O	-2.7901102989	8.0087430721	21.0292972946
H	-2.4153262849	7.8723950664	21.9183596125
H	-3.2952111712	7.2116115120	20.8323331481
O	2.0916672738	8.4344056163	18.6773682819
H	1.8015818000	9.2788261714	19.0583498548
H	1.3116769414	7.9644012136	18.3477892352
O	0.3422678425	9.3396946777	23.8633271523
H	0.7197792920	9.0387418049	24.7016401951
H	0.8099089505	10.1463294332	23.6005727857
O	3.9394623585	3.1595024806	32.4202995968
H	4.0704481448	2.5714053399	33.1747607107
H	4.7939596925	3.6438084415	32.3137031590
O	6.1681534895	4.0730950591	20.8543224350
H	5.5157816405	4.7219131328	21.2034637524
H	5.6619963281	3.3827177622	20.3853179237
O	4.2222188593	9.0430254906	17.1907091912
H	4.2866353216	9.9891101663	17.3890645920
H	3.3857877923	8.7720983395	17.6341238934

O	11.9988940617	6.8983970501	29.1780972685
H	11.7261802485	6.3700101945	29.9569039142
H	12.8594351759	6.5678456385	28.9194554567
O	11.1259571744	5.5027911360	31.3323530364
H	11.3245621432	5.2983886988	32.2679319835
H	10.9505103735	4.6115925703	30.9332795750
O	9.4246705053	4.3624100621	26.5672520513
H	8.5833960208	4.4176176921	26.0712902051
H	9.6890548102	5.2810102286	26.7359513759
O	7.8738775573	9.5768438858	25.4275685740
H	8.3152468967	9.8412941278	26.2433945659
H	7.1220092714	9.0168265158	25.7333969595
O	4.6556519804	9.0811022583	28.8168811666
H	3.7851667529	8.7183889497	28.6040541728
H	4.8430797009	8.7756796200	29.7210117892
O	-1.8058110942	5.2607693581	19.1794315258
H	-2.5492652283	5.2851166835	19.8026496531
H	-1.7349769844	4.3570953048	18.8545083064
O	10.9619228782	5.0177749733	33.9846381734
H	10.0633057496	4.6632217279	34.0349509986
H	10.9218603246	5.8938775389	34.3962008209
O	6.2661022828	1.4067356379	17.1765757460
H	6.2151454213	2.0472116780	16.4216008658
H	7.1976903267	1.3289505250	17.3821212245
O	1.9167004057	-2.1330215778	19.5455045643
H	1.5422602515	-2.0003460192	18.6578692389
H	2.7833742013	-1.6876520008	19.5240443180
O	3.5487184928	4.1693339915	14.5085218404
H	3.7685234647	5.0711866118	14.7834666471
H	3.4575783726	3.6536447562	15.3379855705
O	6.1604115851	3.2421344121	15.2819418257
H	5.9618643692	3.9268131942	15.9563993900
H	5.4307282319	3.3021303141	14.6520052939
O	3.7153939929	9.7042067188	25.4341877088

H	3.8774344224	9.8641994140	24.4675151252
H	3.8514209596	10.5397418947	25.8824912319
O	0.4862128844	1.7612876401	15.4498462245
H	-0.2779799083	1.2533691722	15.7442962922
H	1.2140361011	1.5247367723	16.0320775565
O	4.1017927251	-0.4840639121	19.2708008099
H	4.9469316531	-0.9315296106	19.2306048999
H	3.8753122548	-0.2187083981	18.3405864669
O	7.3079606120	9.0316477043	21.1216225479
H	6.7169970855	8.8409003943	20.3681954287
H	8.0311049784	8.3882895549	21.0489466513
O	4.5173671165	3.0405092871	28.1442659153
H	3.9088898324	2.6664346020	28.8087889766
H	5.3924914256	2.7375284300	28.4585873953
O	5.2034878841	1.1679305522	33.9485274321
H	5.7859272498	1.2029067102	33.1547377261
H	5.0954563979	0.2469863717	34.1842349688
O	-1.2563632726	-0.0590057253	22.2933754301
H	-1.0652776393	0.0741519930	23.2320439046
H	-0.3947442847	-0.1437511453	21.8378096138
O	0.8564181991	4.3324889116	14.6519967220
H	0.5576030827	3.4090850388	14.6325663426
H	1.8035892785	4.2949516334	14.3927966675
O	6.5238861606	3.0356781180	35.5478572639
H	6.0521382978	2.3102698393	35.1084133402
H	5.9286586038	3.8071109204	35.4689752164
O	3.9148846110	11.2746948090	18.7906513327
H	2.9877486401	11.2167707072	19.0617022338
H	4.4358802970	11.1376962156	19.5841507713
O	-4.7556621260	2.7708546745	20.9214610555
H	-4.9906086423	2.8659266640	21.8863492589
H	-5.4804304440	2.3104847824	20.4998082449
O	-5.1611242454	3.1826650785	23.4620303923
H	-4.3437371074	2.7387304662	23.7618523378

H	-5.0084344427	4.1197810493	23.6330606853
O	-1.3757591482	4.2995825338	29.3566260642
H	-1.5952544202	3.5125888619	28.8489450675
H	-0.4682750866	4.5484416568	29.0882681931
O	8.9039847251	6.8018138362	20.6902701480
H	9.7923635006	6.7337685469	20.3432687320
H	8.2910775983	6.4298077921	20.0112538215
O	6.7494862428	10.8044930223	23.1800150797
H	7.1646468444	10.4288370825	23.9752651784
H	7.1409688489	10.3332537185	22.4292021889
O	1.3361690373	10.8123783301	19.8869880371
H	0.4068879670	10.5821527437	19.7571228149
H	1.4558158983	10.9454112299	20.8444675332
O	10.2807469316	7.5843089115	34.8352964743
H	10.5116532542	8.0955022146	35.6099768339
H	9.3351621164	7.3057055524	34.9404202277
O	-0.5797341727	0.6306799096	24.9571891815
H	-0.1025005759	-0.0569860202	25.4305205650
H	0.1332241224	1.2265118818	24.6125019942
O	3.7850756612	6.6439126858	15.8802225982
H	2.8349025703	6.8079398690	15.6912576965
H	4.1332218945	7.5162487491	16.1089490443
O	9.3203002825	1.9679782416	32.7417786157
H	9.9623069355	1.5413210121	33.3090738542
H	9.0292991486	2.7870979275	33.2095668939
O	-0.6152251564	3.9459550208	31.9164967797
H	-1.2131921561	4.4460088602	32.4724301426
H	-0.9662262553	4.0274326477	30.9995761259
O	9.8586423111	7.9546074851	24.4942999891
H	10.1339936812	7.5607106914	25.3378542836
H	9.1852463010	8.6234900916	24.7286659692
O	8.2536974716	6.2444453899	23.3516629064
H	8.4953559152	6.2209315498	22.4143626700
H	8.9252586848	6.8421650467	23.7736838267

O	-1.3111279293	0.1954336099	17.0067241154
H	-1.8779763257	-0.1663380330	17.7143937697
H	-0.5542604075	-0.4138123229	16.9631910354
O	2.3524401879	7.4661606218	31.0876712694
H	3.2481203479	7.6961868837	31.3707706120
H	2.1741442692	6.5684697552	31.4180187648
O	1.8072336819	11.3505503685	22.5775466769
H	2.7213699858	10.9958735789	22.6863131302
H	1.8649619024	12.2981404530	22.7002141604
O	3.1176110667	1.0469472421	22.2863553808
H	3.8810182634	0.7489104981	22.8462943260
H	3.3472528606	1.9358713050	21.9815406494
O	1.1742150351	-1.0678692963	17.0862418209
H	1.3476721210	-0.2304669570	17.5886012099
H	1.8822343810	-1.1143008012	16.4404408108
O	6.0074339532	3.1668417647	23.4589694766
H	6.4361807353	3.6936911003	24.1718503260
H	6.3895621518	3.4510315745	22.6121561692

Cis-**11**

S	2.33591259112006	2.05550949832614	31.98166964376141
C	1.13481068297721	4.93830491522146	20.37886809571275
H	0.68201146639927	5.87756705424098	20.67352556523147
O	1.38831803785143	4.75918198881911	23.53959619228629
O	2.06831995546835	0.96038741172786	31.01858789779955
C	2.37854560761756	3.43565005441696	19.33125665160154
H	3.09300046797275	3.06686889724935	18.60574936337089
O	1.26992828694606	2.21073187519620	32.99566852127030
C	1.65085705688938	2.78527439083071	20.28737074066834
H	1.60898642491950	1.73900821170966	20.56455117356474
O	3.69661974214832	2.00014600912882	32.55977396084636
C	-0.00133691706321	3.50993593866994	22.07556839593677
H	-0.51925618802298	2.55526533686646	21.92608596135724
H	-0.74345503623712	4.31633816423322	22.09293302422988

C	0.77967858067836	3.46944051155896	23.37616501652647
H	0.08276672190845	3.25906808770319	24.20249634323123
H	1.55365696423945	2.68631878953129	23.34895476197029
C	2.19490038394614	4.95798225174175	24.62032284513034
C	2.85084751095093	6.20005880546143	24.67323201858189
H	2.69586616137545	6.91577899110093	23.86549323524324
N	2.04041625468743	4.77506196796470	19.40610482607070
C	2.57620366349984	5.84088759004920	18.55764325872505
H	3.66170883772044	5.89895978815752	18.69578429984530
H	2.11165094670694	6.78674076884090	18.85072581318187
H	2.34387979449803	5.61865525381235	17.51010798678232
N	0.87829655935895	3.73938791523155	20.92427816153255
N	4.79229657379501	5.92897333641713	27.79963145315663
N	4.63461156312208	5.63727212961182	29.01529750213929
C	3.70029117766795	6.48632083960087	25.73098405586658
H	4.23599004822176	7.43579383433435	25.76915510536774
C	3.85788678122825	5.57091715973582	26.78684081812422
C	3.21716554707485	4.32290112974907	26.71707433758106
H	3.37224826527032	3.58055816708113	27.49827551030196
C	2.40300450000052	4.00695207545194	25.63355561125097
H	1.93462764125381	3.02547132885854	25.58962492305567
C	3.40038512121110	5.12728951757190	29.53238592434703
C	2.18897570461318	5.81072919706456	29.33493306803822
H	2.16076862719711	6.69490159028918	28.69841998931630
C	1.03600418559496	5.35801330481192	29.97274985479245
H	0.09730527371317	5.89657184072850	29.83488197358500
C	1.07169639373827	4.21990996454865	30.78480575175622
H	0.16998273755951	3.86095279858639	31.27999023685318
C	2.28599300136357	3.55520300289352	30.98571557839252
C	3.45716904821430	4.02111116944634	30.38767691903146
H	4.40866325060320	3.52058714542749	30.56730763258349

Trans-**11**

S	6.32771758173820	3.53192001220331	32.87923629212356
C	1.33836067358009	5.14401325690331	19.87504522722096
H	1.23492552000717	6.20378369262505	20.07516596256579
O	1.22622286611104	5.32360872326389	22.99771691105958
O	6.52173054502625	4.41528969373755	34.05078245976468
C	2.03335618217170	3.21581051020305	19.03653949621841
H	2.62120260795533	2.55861400986258	18.40762736434960
O	7.42354333293743	2.54892106414092	32.70701372787022
C	1.04660971318371	2.94806360164056	19.94263668092851
H	0.60820284402197	2.01175235008374	20.26574419443443
O	4.98156052869564	2.92232657376268	32.81635083024907
C	-0.35810112570474	4.35331363286835	21.52236219667076
H	-1.13196449815332	3.58436536961431	21.41468084972199
H	-0.81787623247894	5.33949213687460	21.38835145258727
C	0.28969167894385	4.23873160701983	22.89029189999807
H	-0.48629239630578	4.31898286279972	23.66708569193280
H	0.81166622564802	3.27514000477037	22.99584777351702
C	2.04557093481921	5.37283358900339	24.08245052054151
C	2.99585709702411	6.40856615770077	24.08178596980222
H	3.02844920472695	7.09642964294250	23.23664884759851
N	2.19758898979714	4.58915171409835	19.01063779270020
C	3.14840687514677	5.31901818323343	18.17042603445378
H	4.16607169603071	4.99193981737462	18.41145261600447
H	3.04262526452870	6.38874813527936	18.37251653898156
H	2.92944828069801	5.11407616636877	17.11647306150562
N	0.62321482049214	4.16369047268350	20.44869246642230
N	4.76112234837901	5.86031785725882	27.25307983977563
N	4.70228356337329	5.06190307665293	28.23930583701874
C	3.87263839125196	6.53460063106520	25.14746741106003
H	4.61885563367513	7.33025751653745	25.16300560448213
C	3.82149619026847	5.63708188010251	26.23265182406998
C	2.86309892054841	4.60484484458996	26.22448402112957

H	2.81945755484349	3.91060900260081	27.06282677634129
C	1.97996182630457	4.46941684523264	25.16198251693667
H	1.24233104470593	3.66877189168220	25.17484744601086
C	5.66386101356787	5.29294980986135	29.25109755562741
C	6.68481352039942	6.26037781117454	29.18822874202775
H	6.76768858385877	6.89503182650386	28.30713279309697
C	7.57263708338692	6.39246353638207	30.25228151079819
H	8.36444416851723	7.14200242629264	30.20818311309387
C	7.45958650141860	5.57421577207660	31.38292707820573
H	8.15207343742952	5.68751601231538	32.21733270708984
C	6.44553492542263	4.61159771166280	31.44206330428334
C	5.55339248813651	4.46665828060610	30.38173794275323
H	4.75872359387075	3.72192028634342	30.42420111697464

Cis-**12**

S	5.56304702540205	-0.24366172871114	27.97780728556965
C	11.91043929429422	6.64273391764777	29.93048512439818
H	11.79946200897857	5.84613302185733	30.65627384055361
O	8.83576347499008	6.35199443338065	30.30723277831660
O	6.31952828683175	-1.45143563087786	28.37149953423178
C	12.58473735366207	7.81755284579836	28.17876878914474
H	13.15367644631747	8.02909164950093	27.28187474631895
O	6.39017294413376	0.75154208814211	27.25535427393154
C	11.66606536173798	8.56660733454726	28.85830839311312
H	11.27011868914182	9.55652816788233	28.66755166997482
O	4.28994054701739	-0.55395947025794	27.28974929823264
C	10.21384939624223	8.18584268531254	30.90624279016997
H	10.25326690450192	9.27116640983243	31.05450567084566
H	10.43768335749344	7.68950380800072	31.85800822080896
C	8.84107308279985	7.78488071546799	30.39778279154749
H	8.07493021218165	8.13171211403387	31.10875700500680
H	8.64503158617140	8.22897902131692	29.40929340798898
C	7.75776555653709	5.74282230339488	29.73826700055123
C	7.86730829426932	4.34936225428249	29.56982935388825

H	8.79332225328000	3.85477442366701	29.86496025634962
N	12.72096657314114	6.62397667772987	28.86474719424618
C	13.60827614892662	5.52075785104337	28.49282754193954
H	13.33742676740831	5.16353197279545	27.49294632311208
H	13.48678375423275	4.71484080500782	29.22212128256523
H	14.64528829280199	5.87479373309032	28.49550413428288
N	11.26381343555097	7.81863845613591	29.94989516094862
N	4.55763593457510	3.64718009347826	27.96957928636365
N	4.16265629174248	2.49909639871804	28.30195143666564
C	6.81713541347032	3.62744841044375	29.02880743448051
H	6.91687782156602	2.55490841804845	28.86799169019288
C	5.64484707432221	4.29409328561035	28.61757706603108
C	5.57018507587211	5.68924656045958	28.72468762664884
H	4.67507101723035	6.19991701311614	28.36676808954984
C	6.60237698446146	6.41681394404637	29.31327074480945
H	6.50498660369628	7.49506581434208	29.42377393907326
C	4.57347099230213	1.86525922887478	29.51807856026474
C	5.10306242065445	0.56193589757209	29.52031047955428
C	5.33415228762419	-0.08581288643115	30.73560131217474
H	5.77491738718413	-1.08223478971550	30.71773234498039
C	5.01824350081553	0.53495528107631	31.94639889305561
C	4.47187955993235	1.82200284246339	31.94218164100522
C	4.25482837671137	2.48627439016184	30.73692867523314
H	3.82587688955805	3.48870479707098	30.72498929988374
H	4.21625258547691	2.31551182628788	32.88085020898037
H	5.19681673475831	0.01438361435478	32.88769740301412

Trans-**12**

S	6.25799203488540	2.76371604652322	29.91669182825652
C	1.52815033557164	5.17227327008605	20.09003672053361
H	0.93166624151062	6.01547414441137	20.41738311582847
O	1.37860826041875	4.69110660634766	23.21582812871745
O	6.79034377647616	2.95000154964539	28.54811604254910
C	3.06640303467992	3.94731241242633	19.07225838238334

H	3.90578000290404	3.74716493654792	18.41779588213558
O	4.95830461608457	2.05676436222228	29.95947242830690
C	2.31156311468827	3.11336238462128	19.84769714230446
H	2.36595903838391	2.04365332240074	20.00879080893738
O	7.25415078585845	2.18440613725093	30.84581031003703
C	0.38109085959110	3.43809033218128	21.46490696939832
H	0.02144051919270	2.44787452062129	21.16111377187821
H	-0.46190055468748	4.13898217808591	21.45579249129173
C	1.00015512047227	3.35464092543944	22.84794360600513
H	0.25450758894121	2.95435638946679	23.55223010744403
H	1.88411166156748	2.69816283755951	22.84377918804528
C	2.06238047923898	4.87658068593988	24.37720974592111
C	2.48436424372477	6.19271365008813	24.63305112655303
H	2.25285971138172	6.97310372327665	23.90790270816250
N	2.56036182925294	5.22399597638570	19.23865166202487
C	3.05656415388533	6.43259864934247	18.57872931704467
H	4.12536878789438	6.54325990168447	18.79225329821091
H	2.50693779741620	7.29357563697706	18.96971115198583
H	2.89781749031447	6.34614400580530	17.49773320738762
N	1.35465787470517	3.89623453840481	20.46790600080615
N	4.19956657693449	5.85227898868224	27.86255205536907
N	4.47094914484516	4.92275263495257	28.68278888331477
C	3.18687159888162	6.47338571293337	25.79432626115832
H	3.52525129642322	7.48812839102008	26.00866782868843
C	3.48028202574200	5.45414617960937	26.72079970706198
C	3.05030165802775	4.13959760027513	26.45717463205260
H	3.27558803184284	3.35155883322798	27.17493141472096
C	2.34727149958504	3.84707739319542	25.29635897867120
H	2.01834009162629	2.82619783536020	25.10978919734407
C	5.12276775906466	5.34969881564335	29.85846128505870
C	5.93979774174324	4.42777692799412	30.55223752225425
C	6.55278175750447	4.81641035105322	31.74475721857704
H	7.19433415692254	4.10057076373940	32.25694742941282
C	6.35153192239580	6.09636715101934	32.26858756988504

C	5.53104624848202	7.00543023794756	31.59207860800196
C	4.92412921619026	6.63498998144961	30.39556116274149
H	4.26460242641819	7.32119778129082	29.86464310949658
H	5.35740914106395	8.00079670821591	32.00361185021512
H	6.82860890195326	6.37812858864814	33.20812014382477

Cis-**14**

C	1.20563339197692	5.02728716214451	20.33806864166608
H	0.81015482763107	5.97208290503233	20.69122159645253
O	1.27265034899642	4.76487397232010	23.43920914440224
C	2.37439196274569	3.52126923670440	19.21184271696426
H	3.08432494060747	3.15749731129725	18.47940853197045
C	1.57453786617518	2.85569401018982	20.09743735805140
H	1.45281411234168	1.79769460099007	20.29502631841979
C	-0.07162527906691	3.56426068082849	21.89846160849774
H	-0.59293977152000	2.61958745268556	21.70528039606697
H	-0.80620658877963	4.37789707903798	21.91185738695569
C	0.66692868200828	3.48366875282425	23.22251452101164
H	-0.05601480134103	3.25118562731774	24.02051907201233
H	1.43828674718967	2.69754356919618	23.19528227927586
C	2.08802791786335	4.91699050403279	24.52650633345619
C	2.76507728535228	6.14368637161952	24.61591060897909
H	2.62126858663297	6.88706614350977	23.83113481976276
N	2.12754063120818	4.87226016275840	19.37945995148265
C	2.76326753023605	5.95793328037759	18.63114717044653
H	3.84565160455398	5.92407112682501	18.79854546153195
H	2.35998704338399	6.90985122161943	18.98862096042371
H	2.54559379183285	5.83655521816768	17.56409203786831
N	0.84982193561299	3.81269515073363	20.78431608896543
N	4.73856302346914	5.71744037280490	27.71764998920263
N	4.57191305088978	5.42347122326022	28.93351248339132
C	3.62340782806389	6.38308810173971	25.68115318796815
H	4.17455062320120	7.32259899741514	25.74493074594603
C	3.77089262166654	5.43359764901059	26.70564541950652

C	3.10365255815560	4.20321194031131	26.60140834530379
H	3.23772172481772	3.44352014667327	27.37060129903063
C	2.27898832672647	3.93441890009409	25.51163324320563
H	1.78445857032949	2.96690023171171	25.44433980062141
C	3.31159669549612	4.98765349276766	29.45933660339525
C	2.12819901258856	5.70878012072099	29.20682793980230
H	2.13039472056085	6.57169053341426	28.54314714444963
C	0.96317700169085	5.28576690967948	29.85539816443650
H	0.03447099222078	5.84016062111075	29.69357822617224
C	0.95530928265788	4.17400704986652	30.69730545428644
H	0.02727653978979	3.86447066300458	31.18627572352566
C	2.14743881800992	3.41511238945315	30.98425873640612
C	3.33766452040624	3.90518642518791	30.34122489319585
H	4.28759851611779	3.39955106025281	30.53507057868056
O	2.15400280749983	2.39512163130823	31.77242901680864

Trans-**14**

C	1.28726686042609	5.19102629383578	19.87655658524635
H	1.16038681805048	6.24264997156877	20.10434528630223
O	1.24218081576999	5.27926297871779	23.00537587717888
C	2.01641839196258	3.30260952767459	18.97862853706427
H	2.60830557935384	2.67676116738404	18.32217639213935
C	1.05622040617024	2.98713294895261	19.89776592820425
H	0.64942750742240	2.03202748681624	20.20690816521160
C	-0.35368056338615	4.31767385042984	21.53770664628504
H	-1.11343651652870	3.53562172906901	21.42374663093746
H	-0.83552001031292	5.29737560696930	21.43758524426581
C	0.32218761627965	4.18374266585574	22.89039241558666
H	-0.44262807813454	4.23258585588054	23.68128699239722
H	0.85939285098530	3.22530122479250	22.96604888498101
C	2.07691700508712	5.31524526652571	24.08387519462219
C	3.01922535667759	6.35676415829350	24.09263899706567
H	3.03879456961717	7.06049100400701	23.26003255258932
N	2.14318323410180	4.68014405768264	18.98222144530516

C	3.05647728773190	5.45560518603473	18.14129518833814
H	4.08712389776322	5.14910901282104	18.35229149483935
H	2.92808184010824	6.51664861986065	18.37347202488973
H	2.82000862378497	5.27199543047486	17.08719262995726
N	0.61086115360374	4.17854903860204	20.44108600019743
N	4.82712867949107	5.77159517049793	27.24343097662904
N	4.79491443635369	4.93651767685505	28.20033821056582
C	3.90642899734801	6.47182244354159	25.15315370931330
H	4.64545677989750	7.27438589031479	25.17360118582969
C	3.87666781605794	5.55671540103794	26.22296327900449
C	2.92607110667862	4.51917935849754	26.20427776226185
H	2.89512559450508	3.81061862147172	27.03117620871423
C	2.03097576528196	4.39486708656274	25.14781282952375
H	1.29893821059475	3.58882575087389	25.15658031754327
C	5.74390158296186	5.14487613006880	29.22932857211783
C	6.70095529608668	6.18377543875318	29.23872085674514
H	6.75223177409284	6.89583923345081	28.41798236910496
C	7.56724824828446	6.25370029178696	30.33198799056660
H	8.31924760562426	7.04745433571257	30.36501436810974
C	7.50319690385245	5.34073641963095	31.38874640525515
H	8.19845287010890	5.42879370759035	32.22865883004756
C	6.54505719360231	4.26610872369860	31.42207043112332
C	5.67004023970349	4.21999124557433	30.28143382319135
H	4.91162610834612	3.43408333482849	30.22808982428505
O	6.47135014459389	3.41667065700268	32.38938293646260

Isomerization of 10c

14.97°

C	-4.01395385526578	2.04724596238119	-0.92525118040871
C	-3.97296214446559	0.76845697691088	-0.43948154527314
N	-2.80524910235184	2.63144773076194	-0.63154421788506
N	-2.72857124004784	0.57285167702438	0.11786449896715
C	-2.01284011222773	1.71968403852987	-0.02086347634897
N	-0.82119398844124	2.09987343314147	0.58662176316185

C	-2.40198292229478	3.99930809449628	-0.94389056008228
H	-3.30625072782407	4.60587349452786	-1.04695114987322
H	-1.82577086956852	4.01997484081784	-1.87678882377985
H	-1.78483836866232	4.37631934362724	-0.12060244223825
H	-4.79853609390000	2.58645613217219	-1.44084008736937
H	-4.73019099541574	-0.00531261543594	-0.41491336510266
N	0.22253070222567	1.38705579309120	0.61459741582309
C	0.40896483982743	0.26217051819906	-0.21757511003246
C	1.21274886218591	-0.76562630107101	0.30685210529786
C	1.47519890971702	-1.89542365208433	-0.46271020305202
C	0.99400048480432	-1.97438190624563	-1.77438055468315
C	0.24800339648830	-0.91927964182270	-2.31750792724575
C	-0.05824949444716	0.19622837116791	-1.54559282390355
H	-0.61168115290682	1.02727382270369	-1.98261162660750
H	1.21824081695967	-2.84962258001733	-2.38488731628768
H	-0.08969776110683	-0.96534483753306	-3.35318065994817
H	1.59231168000484	-0.67041365666988	1.32445772129522
H	2.06954493704221	-2.70914174187151	-0.04665539357607
C	-2.32236190408944	-0.62699861133374	0.84326176490063
H	-3.22885740248874	-1.13491775506828	1.18487352077386
H	-1.71651967976794	-0.34272183808614	1.70963149356791
H	-1.74566093643129	-1.29010268506252	0.18643700217849
N	-4.82153644588846	2.57784813341670	2.24877978056962
O	-5.67213792464772	1.66839419204942	2.48213853828747
O	-3.59673862590639	2.38461919375374	2.51487820240260
O	-5.19347288110896	3.68113607352911	1.74796465647215

29.93°

C	-4.07530055628155	2.07182757174868	-0.89175157102166
C	-4.03064916078424	0.79228043628002	-0.40515210084101
N	-2.85094930414622	2.64126041142953	-0.64893873933353
N	-2.76860902885923	0.57890556535666	0.10091560367033
C	-2.04344137741780	1.72206293240094	-0.06129514847151
N	-0.83678405552570	2.08951165292036	0.48969135133970

C	-2.44169700766044	4.00173704362569	-0.98180261880947
H	-3.34142706868151	4.61870121211734	-1.06061178074073
H	-1.89368017643981	4.01064664792012	-1.93176510814132
H	-1.79431138114445	4.37480190741644	-0.17996853464619
H	-4.87671214241942	2.61966370545196	-1.37114688708729
H	-4.79835938769845	0.03086315959125	-0.34459553527952
N	0.14532229165511	1.30051238237255	0.65138353757653
C	0.38556560312068	0.20582497991085	-0.19649988243587
C	1.18476763760842	-0.82510444541631	0.33489944739161
C	1.51818281530036	-1.91943844514122	-0.45646246846392
C	1.10404339137959	-1.96694214560842	-1.79274252056014
C	0.35309507662092	-0.91498342699978	-2.33695474681818
C	-0.02053050833523	0.16595479266631	-1.54748373035295
H	-0.57784693481476	0.99411809742710	-1.98496966765613
H	1.38313341779458	-2.81422568832707	-2.41981901911524
H	0.06582139282656	-0.93722055487886	-3.38856955846128
H	1.50910194510120	-0.75520213535007	1.37348194099144
H	2.11369776898323	-2.73106234296603	-0.03801718121912
C	-2.35989309560002	-0.62108191466819	0.82240428920549
H	-3.26548679056132	-1.13319705869164	1.15996684842762
H	-1.75675668963450	-0.33700028738297	1.69147592884019
H	-1.77617923602056	-1.28047674115891	0.16750415667360
N	-4.76865088953036	2.62017947375229	2.29256350084161
O	-5.61984173363095	1.71823669799351	2.55158148346449
O	-3.53838279853960	2.41602040237381	2.52400474071988
O	-5.14495201666433	3.72575611383392	1.80080397031260

44.89°

C	-4.17232872437537	2.13563702563332	-0.84040276660784
C	-4.13423100863460	0.84815946207680	-0.37046938753795
N	-2.92275496146498	2.66937158598630	-0.66885071514866
N	-2.85169997876083	0.59153910413773	0.05360326166539
C	-2.10397898191822	1.72352882098445	-0.12929194136855
N	-0.87334901792277	2.05635548506629	0.34424668411735

C	-2.49919230219195	4.02340823662337	-1.00491498985494
H	-3.38688582092296	4.66201330790441	-1.03261252203211
H	-1.99613665887112	4.03363161058109	-1.97949481652901
H	-1.80482806529274	4.36987253121447	-0.23040090389440
H	-4.98856509299138	2.70994492018661	-1.26023846033579
H	-4.92031885554228	0.11022951187422	-0.26857168819153
N	0.04697253876315	1.21918642803082	0.63559984543174
C	0.36885955973108	0.14517293368549	-0.19765628556408
C	1.18498669421231	-0.85760826305870	0.36827453598769
C	1.61286132100314	-1.92484094533776	-0.41240892815783
C	1.26751611282204	-1.98206476637333	-1.76862219628589
C	0.48696440598863	-0.96692014177116	-2.34214754329060
C	0.02242427789448	0.08754101054341	-1.56721004432909
H	-0.55828286683356	0.88843369634066	-2.02453000735410
H	1.62163350656153	-2.80772151744061	-2.38651286762422
H	0.24914278616484	-0.99829955422399	-3.40589119495328
H	1.45241951192440	-0.78123528945758	1.42254268845531
H	2.22727449001905	-2.70945996594155	0.02957665366762
C	-2.43385338220575	-0.62971050634581	0.72933807371664
H	-3.33477609426272	-1.16206140671914	1.04725866294256
H	-1.83128245695902	-0.37282651260143	1.60845501625875
H	-1.84269027139426	-1.25952271049175	0.05157864567505
N	-4.67661630322715	2.66035554675269	2.38088377504663
O	-5.54390061379923	1.78111940964934	2.66265740292226
O	-3.44423087145309	2.41150587649403	2.54802837629324
O	-5.03886287606044	3.78819507599717	1.93031363687972

59.84°

C	-4.28002162898301	2.22129795827615	-0.77138404190466
C	-4.25678731937356	0.92142351484281	-0.32966146229833
N	-3.00620466168090	2.71028651783335	-0.67725782559982
N	-2.95968122910780	0.60936154069940	-0.00386544646591
C	-2.18306951070969	1.72605304919601	-0.20088410493755
N	-0.92542320757534	2.00334917133839	0.17614985089795

C	-2.55840834145786	4.05557886760487	-1.01047503773844
H	-3.42666066633492	4.72065220020911	-0.99039121435734
H	-2.09565699169704	4.06882528179741	-2.00494019777707
H	-1.82077141066426	4.36771131030551	-0.26107154261681
H	-5.10372003689157	2.83102497946328	-1.12079200504153
H	-5.06225139347994	0.21127556751654	-0.18877270142185
N	-0.05410891387121	1.14698462403399	0.58246959522679
C	0.36313045738022	0.09238892944090	-0.21550247218482
C	1.20897211935718	-0.86036071994621	0.39993077448312
C	1.72857790844593	-1.90896581387243	-0.34684077241713
C	1.43890108430794	-2.00665318901587	-1.71492363278440
C	0.61846649048087	-1.05062380028562	-2.33440013259634
C	0.06521612690966	-0.01395339373616	-1.59652321309813
H	-0.54840602838403	0.74314527501493	-2.08475057919976
H	1.86606987710894	-2.81779265782940	-2.30527297345894
H	0.41989541828101	-1.11620233416904	-3.40466421374946
H	1.42820971454802	-0.75392079758647	1.46268873299376
H	2.37020742545687	-2.65011927108699	0.13019165790896
C	-2.52931817876962	-0.64307962768262	0.59883049886114
H	-3.42306662088357	-1.20123637771636	0.89113016934994
H	-1.92184005270244	-0.43021568909849	1.48761962961069
H	-1.93409919643945	-1.22833913477699	-0.11528293343987
N	-4.55960707696675	2.67897724186019	2.46836222134324
O	-5.43872406881147	1.82011981782171	2.77512138277987
O	-3.32895147458497	2.38654769341146	2.56012295398495
O	-4.90857861290695	3.82938926613649	2.06716903564780

74.80°

C	-4.41215511833478	2.33459846327007	-0.72700471660063
C	-4.42091453849585	1.02294202391493	-0.31536194142801
N	-3.11550819207614	2.76920091830571	-0.70356668935704
N	-3.12272222291168	0.64439319090008	-0.08481557926630
C	-2.30464434243114	1.73727315887386	-0.29297892408161
N	-1.02514363523354	1.92954713486634	-0.02406986742328

C	-2.62798082024976	4.10180868353183	-1.02489145860197
H	-3.46320172520173	4.80347258330914	-0.94328208040684
H	-2.21361406907849	4.12584205864355	-2.04050404053779
H	-1.83995973608040	4.35974478637500	-0.30594694521445
H	-5.22968432640755	2.98554658309380	-1.01071743059225
H	-5.24970968042326	0.34868687117508	-0.13831925366817
N	-0.17784894188756	1.09391132364397	0.49018407831187
C	0.34564578304219	0.05417660639194	-0.24193040922424
C	1.23301610870777	-0.81263816424533	0.44704795567206
C	1.85355692522029	-1.84939619703693	-0.23256509818833
C	1.62204505154230	-2.02797956637482	-1.60537332636986
C	0.75568001032501	-1.16486025927796	-2.29669143341012
C	0.10383260744377	-0.14004192092462	-1.62847224536303
H	-0.54259043094494	0.55207608751816	-2.16859085569010
H	2.13070111705366	-2.82876411501508	-2.14286594595442
H	0.60158481896791	-1.29487716943096	-3.36843100072638
H	1.40374923019969	-0.64406907537556	1.51067274655910
H	2.52935750329288	-2.52029143447664	0.29821921255600
C	-2.69019537564745	-0.64485670024680	0.42668092926905
H	-3.57998334479029	-1.21825517571159	0.70103475254058
H	-2.06289224036175	-0.49176043897009	1.31492250975254
H	-2.11025244803857	-1.18491622065024	-0.33526219053374
N	-4.30273502825964	2.60578742313534	2.59199253192918
O	-5.16593823329711	1.71624846383932	2.85285031330763
O	-3.06743731950968	2.31921625676880	2.62446761864552
O	-4.67176738613372	3.78116382017954	2.29569878409508

83.20°

C	-4.47572114284431	2.39797808576804	-0.68988149685339
C	-4.51242302060040	1.08140065707593	-0.29507353538730
N	-3.16537816864830	2.79420323180395	-0.70719880614888
N	-3.21914833481179	0.65578109268431	-0.12614647925278
C	-2.37212559524969	1.72977255690163	-0.33651164045379
N	-1.08554055478267	1.85579118178374	-0.12936713648390

C	-2.64527203653509	4.11579490746813	-1.01704326463046
H	-3.44581650172929	4.84673005456248	-0.86949011408750
H	-2.28365532513487	4.15724083629298	-2.05230683882881
H	-1.80993553001831	4.31970669478635	-0.33442245271231
H	-5.28254756848929	3.07801767282270	-0.93326245182273
H	-5.35748934804566	0.43565534098025	-0.09077914430114
N	-0.22819295531629	1.06169141717693	0.43597766358587
C	0.35385766588697	0.03425115869628	-0.26185589593558
C	1.27118029806178	-0.77344865118739	0.46177434994588
C	1.93734358379217	-1.80453515310549	-0.18091797146306
C	1.72250714085434	-2.03513160666906	-1.54922373668002
C	0.82773789409463	-1.23022807095373	-2.27518497284750
C	0.13263199268734	-0.21086151990441	-1.64509690317029
H	-0.53442369839336	0.44240379991009	-2.20824236946106
H	2.26813605599218	-2.83062082345144	-2.05759428965039
H	0.68865450539642	-1.40148004678964	-3.34313178957950
H	1.42505608348613	-0.56455704037355	1.52077012896449
H	2.63585450058233	-2.43107273738419	0.37403124178277
C	-2.79228026975203	-0.65457354293358	0.33044946827668
H	-3.68246046449974	-1.23847685646259	0.58043177471649
H	-2.16315897476961	-0.54390249410682	1.22424113495422
H	-2.21493069127674	-1.16292658674557	-0.45521588635563
N	-4.20333872630928	2.58469066974701	2.63859653398721
O	-5.06808168968598	1.70558899186296	2.92783529161215
O	-2.97349120116903	2.27605714035691	2.60167522947651
O	-4.56525792277203	3.77198963938656	2.38429435880383

91.60°

C	-4.50890121751495	2.40529607499293	-0.66149993042504
C	-4.54626600585330	1.09138757757792	-0.25931950499947
N	-3.19573671674466	2.79191527952611	-0.71346082637987
N	-3.25186981949255	0.65624841016027	-0.11662232684216
C	-2.40040177461619	1.72361110736348	-0.35222478520445
N	-1.10785554694835	1.83361510031047	-0.19469211477595

C	-2.67323723488284	4.10704867199702	-1.04302561265051
H	-3.46518370986637	4.84498206390390	-0.88355445686383
H	-2.33262834838114	4.14039701976692	-2.08587868630672
H	-1.82244929208795	4.31088317373308	-0.37939941294612
H	-5.31629027932866	3.09126598937692	-0.88564339496529
H	-5.39114590944151	0.45313427667738	-0.03222825245407
N	-0.29582164704163	1.00343221960977	0.41083265518251
C	0.33533717830628	0.01142806654205	-0.27760914552018
C	1.21400705959979	-0.81903029309598	0.47467602716005
C	1.91905787541774	-1.82760613217730	-0.15908800492547
C	1.78414410701663	-2.01757568831829	-1.54485958117240
C	0.92842141402544	-1.19367269933281	-2.29827409999461
C	0.19530225748943	-0.19532767024477	-1.68109269128388
H	-0.43929909488821	0.47621276092227	-2.25986335945360
H	2.36340223766809	-2.79472527599539	-2.04425098195716
H	0.85348787842434	-1.33393224757155	-3.37712616183080
H	1.30358431249867	-0.64338667272900	1.54693659018312
H	2.58716831211284	-2.46931457508847	0.41554586293599
C	-2.82472102523250	-0.65492919121004	0.33471666955807
H	-3.71102948280777	-1.22311831111974	0.63107505909703
H	-2.15609530572662	-0.54296646792437	1.19971716580776
H	-2.28799749936816	-1.18173410259732	-0.46745164247921
N	-4.14443012319662	2.60493587776630	2.65704184619427
O	-5.00638160114596	1.73303477506598	2.97448160169564
O	-2.91819433477633	2.28833345202132	2.58806005927261
O	-4.50568666321647	3.79308743009071	2.40621143634380

100.00°

C	-4.30177112078857	2.18343576553598	-0.77033776454733
C	-4.43409558175242	0.91992476156045	-0.24373592949052
N	-3.00272757219307	2.57436709975882	-0.59863397501633
N	-3.22224580868427	0.55420291669816	0.28807589496294
C	-2.31218763802636	1.56253023755840	0.03544359787063
N	-1.01942106492664	1.60620504635838	0.21520040608759

C	-2.39672524722075	3.83756167715069	-0.98035190170670
H	-3.01402615761781	4.30056548413173	-1.75590925231691
H	-1.38829324987448	3.63188630635568	-1.36451840299271
H	-2.32295388828637	4.50167633781681	-0.10967585425354
H	-5.02988408164637	2.81478326405124	-1.26414905394642
H	-5.28970291799975	0.25597927743315	-0.22116648328871
N	-0.25164286026297	0.65035152416358	0.66046064914586
C	0.44582813022330	-0.05659195737759	-0.28450457731625
C	1.38731252126486	-1.00134804728401	0.20822988474903
C	2.02694506127955	-1.85760924889498	-0.67163677541692
C	1.74976851570915	-1.78395835020520	-2.04731250339406
C	0.82998250760877	-0.84385989326885	-2.54248622436501
C	0.18656496785039	0.02940931835174	-1.68025541951224
H	-0.54333793877696	0.74874676590027	-2.04837047303768
H	2.24320875906523	-2.47051462045666	-2.73612380673032
H	0.61540503575709	-0.80763864917396	-3.61103910325734
H	1.57025968714009	-1.04075667047656	1.28232864728892
H	2.74155903568542	-2.59098173031699	-0.29787178931403
C	-2.96107045657241	-0.63959788401889	1.07741744973264
H	-3.71836750823463	-1.39015748479469	0.82985327987819
H	-3.00460143789415	-0.39678295939894	2.14687197957021
H	-1.95985960583437	-1.01997328542122	0.84278528400454
N	-4.61136077809644	3.04798891374081	2.39111396436344
O	-5.46733399190822	2.20200241880923	2.78601154079546
O	-3.37350474743824	2.81764016855998	2.54812632174382
O	-4.98943056754805	4.12344349715321	1.83829038970983

107.23°

C	-4.35659483685059	2.21143064408214	-0.73260852855751
C	-4.48100021101479	0.94675559316033	-0.20425578280501
N	-3.05059983784174	2.58805699395295	-0.60466026365685
N	-3.25342892159442	0.56520933204267	0.27313674334909
C	-2.34716694496893	1.56814810357376	-0.00234670267422
N	-1.03821042966831	1.60897092907669	0.10855961930144

C	-2.45255800299544	3.85219608933543	-0.99961869229388
H	-3.09590896233979	4.32211685789728	-1.74918321460397
H	-1.45844908672162	3.64700297974409	-1.41799041171404
H	-2.34909698450313	4.50855471238560	-0.12622614593393
H	-5.09616887863063	2.85264093015239	-1.19576142063731
H	-5.34165447166230	0.29115214118405	-0.14970308551220
N	-0.31481530653621	0.63730545096356	0.59566742539091
C	0.42750172664590	-0.07635478587093	-0.31087896210281
C	1.34843703012990	-1.01617511356871	0.22540970260896
C	2.03983360147794	-1.86453768012446	-0.62333615281935
C	1.83209179912401	-1.79114128136479	-2.01118653524230
C	0.92817933671596	-0.85983663062435	-2.54843416151318
C	0.23365260108726	0.00537698935203	-1.71697049192129
H	-0.49147921675564	0.71171123094758	-2.11822764484337
H	2.36448203148238	-2.47272357942214	-2.67553501197230
H	0.76250400653211	-0.82537246936619	-3.62588227621685
H	1.48104535163131	-1.05644242306385	1.30697530974362
H	2.74152654645494	-2.59200983902436	-0.21494179270058
C	-2.98299342983574	-0.65054354129361	1.02850937788885
H	-3.76600381944268	-1.37909666629558	0.79564265657947
H	-2.97896167828730	-0.42797849014730	2.10285720578044
H	-2.00017358020200	-1.04366328426098	0.74580130106405
N	-4.53144835022981	3.05082985408932	2.44238603551292
O	-5.38056433069809	2.20986818797726	2.86194073977059
O	-3.29085000677824	2.80591582319341	2.54852803673082
O	-4.91883674372376	4.13556294131644	1.91446312399989

114.46°

C	-4.42868930933800	2.24136050977930	-0.69581185722434
C	-4.55323897276988	0.97966706442919	-0.15995260642450
N	-3.11172701835944	2.59121276133334	-0.62384643462353
N	-3.31334816617132	0.57422511685373	0.25959030144982
C	-2.40482982229883	1.56146871427296	-0.04959758910324
N	-1.08409506656555	1.58361174861324	0.00225030476947

C	-2.51214620371570	3.85129610073427	-1.03402176404696
H	-3.16736097335121	4.32415985824581	-1.77136026659811
H	-1.52863918071849	3.63902396584490	-1.47169464897998
H	-2.38849765280694	4.50701571738708	-0.16302892947072
H	-5.17441057232510	2.89619010672069	-1.12904788719267
H	-5.42267494297511	0.34061476030659	-0.06420930104514
N	-0.39631794610434	0.60877570560895	0.52489195540368
C	0.40434087214443	-0.09785037013538	-0.34196206181830
C	1.30565513400361	-1.02520423618128	0.24193660088059
C	2.06377437256496	-1.85536747489332	-0.56871123295537
C	1.93789169169544	-1.77697751182614	-1.96510882427856
C	1.04816152512077	-0.86052610632129	-2.54874392965238
C	0.28864625458682	-0.01432234515463	-1.75437551375651
H	-0.42968658027604	0.67736086669217	-2.19218124989185
H	2.52152654795614	-2.44394384851263	-2.60060726320960
H	0.94321880522064	-0.82335034637346	-3.63370403073252
H	1.37534999287501	-1.06988449083046	1.32920785039501
H	2.75348480157360	-2.57224779144325	-0.12274687973647
C	-3.03537228786906	-0.66457056411006	0.97617077665023
H	-3.87090471034716	-1.35002010221030	0.80257315932922
H	-2.92923908458143	-0.46020479100010	2.04822766209779
H	-2.10259271745664	-1.10092251443518	0.60282786637923
N	-4.40253179798115	3.05095691645956	2.50689484403530
O	-5.27262682789978	2.24016072693469	2.94229450179985
O	-3.17131309742467	2.74846625108904	2.56115419541072
O	-4.75951706640492	4.16275560212169	2.01482225213995

121.68°

C	-4.49591864824057	2.27676892185705	-0.64156372843690
C	-4.62404692581646	1.01702112408967	-0.10327708779353
N	-3.16838288456262	2.59201966894608	-0.63563530003768
N	-3.37445576326568	0.57871050738461	0.24853110201074
C	-2.46182025619485	1.54651675961063	-0.09727590246933
N	-1.13295547294497	1.54151809618901	-0.10681018896774

C	-2.56324309071805	3.84402251433403	-1.06622328000364
H	-3.24276259526549	4.33237929537095	-1.77073899377903
H	-1.60465217924428	3.61831955905534	-1.54879103741341
H	-2.39103395076703	4.49134302331834	-0.19726159216255
H	-5.24590542256738	2.95321752644194	-1.03203249341351
H	-5.50295536803809	0.40016568813234	0.03938162330709
N	-0.47436509114640	0.56781729247175	0.44680974366063
C	0.38408559195436	-0.12606792289456	-0.37912957767962
C	1.26836077556473	-1.03682316780404	0.25099615286053
C	2.09334569800417	-1.84329325310786	-0.51885446005885
C	2.04751646736296	-1.75861936740757	-1.91898558322040
C	1.17026628891239	-0.86098378355502	-2.54830032987654
C	0.34511392039039	-0.03906960427150	-1.79395292128485
H	-0.36613745835228	0.63618974152049	-2.26780780617146
H	2.68316858927515	-2.40702072538804	-2.52303216624449
H	1.12640251715795	-0.81958287172065	-3.63726917552118
H	1.27662357664062	-1.08708021221374	1.34022768566963
H	2.77297753578346	-2.54649173150702	-0.03695841703760
C	-3.09581402720458	-0.68527823810572	0.92149145180594
H	-3.95898436422818	-1.34176697287214	0.77261318738982
H	-2.93152619484243	-0.51132458100878	1.99126870137884
H	-2.19595474621047	-1.13726272101876	0.49105882727988
N	-4.28969654512658	3.06725240389116	2.56153871369573
O	-5.15371101010555	2.27338210286765	3.03819735245349
O	-3.06398626675107	2.73845133132075	2.55212030992977
O	-4.64726269945250	4.18849959607332	2.09179519013033

128.91°

C	-4.55811343022308	2.30763801632721	-0.58802996032436
C	-4.69338910721541	1.05218748293830	-0.04259172577228
N	-3.22321720652880	2.58498052889547	-0.64895098043376
N	-3.43946604644577	0.57958867925624	0.24427782976219
C	-2.52205369050113	1.52389741564629	-0.14210820392829
N	-1.18857522286002	1.48722090560624	-0.21034091947356

C	-2.60954080874483	3.82420037565417	-1.10709830988618
H	-3.31045624807096	4.32814609822930	-1.77889085415355
H	-1.67999876408905	3.57916767253605	-1.63399501729051
H	-2.38392069856132	4.46582164692213	-0.24632278916767
H	-5.30723981582441	3.00567862641112	-0.94022517586766
H	-5.58020662053305	0.45967796345966	0.14650126943424
N	-0.55490222488171	0.51604741222903	0.36758586579148
C	0.36397924904927	-0.15484771942845	-0.41829888146008
C	1.22371560197491	-1.05739227202253	0.25214805504087
C	2.11403476775269	-1.83404482696744	-0.47648096585468
C	2.15453804629634	-1.72714184384660	-1.87454843122722
C	1.29856778235076	-0.83837568940490	-2.54420160821150
C	0.40910344869724	-0.04744667293528	-1.83006830571040
H	-0.28647845430067	0.62057200749798	-2.33651095041243
H	2.84149788949518	-2.35145624179765	-2.44707656051700
H	1.32218551432838	-0.77949275756661	-3.63303168063541
H	1.16453650305904	-1.12585310052522	1.33887057011596
H	2.77692980073984	-2.53087381371564	0.03701063035481
C	-3.16384662213927	-0.70309607895573	0.88436712744783
H	-4.05418119547004	-1.33001411574819	0.77229864197564
H	-2.93782221370010	-0.55058662249273	1.94588147786541
H	-2.30256630636274	-1.17446072317581	0.39951783991493
N	-4.17371188496094	3.08365784497590	2.60866662826325
O	-5.02973888892048	2.30852389855332	3.12876370986540
O	-2.95777074530642	2.72712778839022	2.53518441021451
O	-4.52960240810279	4.21387811505384	2.15982726428013

136.14°

C	-4.61540106286782	2.33778559629969	-0.53430345275054
C	-4.76130153989109	1.08829814610360	0.02096944283136
N	-3.27678435596543	2.57250017272164	-0.66361914728776
N	-3.50910799931395	0.57784633672469	0.24403404242350
C	-2.58603097071274	1.49449653000527	-0.18566792674446
N	-1.25191128954896	1.42168668446541	-0.30996614440230

C	-2.65126702600142	3.79465051981383	-1.15377755161133
H	-3.37085428176163	4.31760030925342	-1.79040494627763
H	-1.75685141254706	3.52474880148688	-1.72647110483375
H	-2.36586516502960	4.42936702291108	-0.30585367694626
H	-5.35871772244716	3.05944614207858	-0.84932982357238
H	-5.65485761078337	0.52344736815164	0.25710732456060
N	-0.63888233039529	0.45332977721930	0.28629764703894
C	0.34076078572539	-0.18573489511900	-0.45864131413087
C	1.17628025266340	-1.08134308017342	0.24726583290144
C	2.13316900968756	-1.81968998106870	-0.43732394033504
C	2.26100427342945	-1.68115923445503	-1.82654636719898
C	1.42643208715019	-0.79995905552259	-2.53224996965701
C	0.47173754646245	-0.04804820371684	-1.86096435506359
H	-0.20654739112757	0.61428421233059	-2.39754896971863
H	3.00051267267483	-2.27447718016143	-2.36566312525659
H	1.51891252815663	-0.71616529600679	-3.61577158036376
H	1.04850992465194	-1.17512955398795	1.32621424115614
H	2.77938545656817	-2.51071598294645	0.10450227451424
C	-3.24308288437758	-0.72194498857916	0.85485390882989
H	-4.16209172121005	-1.31323839085191	0.78997971855977
H	-2.94864012552221	-0.59038473566390	1.90198473628867
H	-2.43142437642679	-1.21897336206540	0.31349521715042
N	-4.04719611517884	3.09792344364586	2.64790573691433
O	-4.89460021616760	2.34470801651216	3.21238508694116
O	-2.84685916186232	2.70925525504072	2.50896210347584
O	-4.39613977803082	4.23851960555391	2.22027608256472

143.37°

C	-4.66407163292777	2.36436144841572	-0.48300520663378
C	-4.82593870998405	1.12426641281571	0.08790738333335
N	-3.32536540808093	2.55062359928808	-0.67804570672350
N	-3.58235857709202	0.57211968651493	0.25332856018421
C	-2.65163679178761	1.45487156419835	-0.22256700368053
N	-1.32065744553054	1.34196744013153	-0.39871132645611

C	-2.68406361185811	3.75146616162318	-1.20221613987985
H	-3.41610732243398	4.29462824608252	-1.80692050060811
H	-1.82617891044176	3.45305457280927	-1.81446658231810
H	-2.34164089851499	4.37884774543280	-0.37011602421981
H	-5.39539970145753	3.11109624849541	-0.76597177163436
H	-5.72558149735844	0.59207546940188	0.37191979520511
N	-0.72662366586121	0.37127151367803	0.20517978054057
C	0.31308228308664	-0.22400744602362	-0.50056629031965
C	1.12350686793068	-1.12021355884542	0.23027352213457
C	2.14825973850530	-1.80679428278333	-0.41045515779614
C	2.36590676047551	-1.61559435070826	-1.78141944738943
C	1.55396926081550	-0.73442130085905	-2.51320679589010
C	0.53274219001312	-0.03461512347462	-1.88434339944204
H	-0.12543972547386	0.62908979821928	-2.44379134753734
H	3.15943300148779	-2.16674263549315	-2.28755906074454
H	1.71784569725501	-0.60888347313537	-3.58418943847700
H	0.92583615373820	-1.25496600617760	1.29428430206577
H	2.77704310033355	-2.49743466626643	0.15198378885321
C	-3.33290896303450	-0.73941278198550	0.84761108918912
H	-4.27766625945199	-1.29225460148172	0.83739220255921
H	-2.97122325120333	-0.62191713555945	1.87493382265843
H	-2.57597928443322	-1.26724912136948	0.25828635925802
N	-3.91764407734590	3.11480965732167	2.66475439671512
O	-4.75815444205547	2.39012234728457	3.27479994181812
O	-2.73838213795154	2.68857738031036	2.46759111554176
O	-4.25231273936185	4.26418719213938	2.24943513969393

150.60°

C	-4.70799163882186	2.38483913100185	-0.43398100056815
C	-4.88498583877109	1.15709368979591	0.15797509197892
N	-3.37452963125697	2.52019799326436	-0.69730201112114
N	-3.65490160856386	0.56190148056846	0.26567164586653
C	-2.71935806312823	1.40717302339672	-0.26203494686190
N	-1.39505792035086	1.25497652559245	-0.48750505986578

C	-2.72003443740296	3.69765241637318	-1.25937533230687
H	-3.46250308119282	4.25977054685884	-1.83328167927337
H	-1.90105303255443	3.37064482858179	-1.90842481442580
H	-2.32208668179795	4.31841685848006	-0.44735085581045
H	-5.42454058993381	3.15653773787995	-0.68601471370840
H	-5.78693047302291	0.66097405868061	0.49503560080418
N	-0.81695922189244	0.27916560246922	0.11747354000476
C	0.28200787555460	-0.26384719157694	-0.54628324934926
C	1.05841657358037	-1.17547865827226	0.19975910082851
C	2.14915175157432	-1.80373702343392	-0.39177282996478
C	2.46507483973806	-1.53757419940501	-1.72989815598833
C	1.68601001919886	-0.64062883589950	-2.47847219210599
C	0.59960758115229	-0.00000427821771	-1.89743524557602
H	-0.02969607639151	0.67817215045279	-2.47246912867488
H	3.31149084034130	-2.04078651678342	-2.19896544043300
H	1.92867055766402	-0.45529236592542	-3.52556197322337
H	0.78433053401898	-1.36785416701270	1.23770470999095
H	2.75251223636266	-2.50602655709083	0.18393473676756
C	-3.42359299268065	-0.75587123072910	0.85496902474758
H	-4.38945530973869	-1.26804434947072	0.90814842521184
H	-2.99426966018497	-0.64627270897214	1.85668312793190
H	-2.72719664655207	-1.31879337433886	0.22503837619577
N	-3.77600500364077	3.13428948700398	2.66953270850518
O	-4.60807036088604	2.44522694016950	3.33025793079155
O	-2.62468116305370	2.66440644185369	2.41460547292314
O	-4.09108337736613	4.29170254470481	2.26146913670921

157.83°

C	-4.74543519659955	2.40236478476354	-0.39344883717673
C	-4.93819201323172	1.18894599504285	0.22258654538180
N	-3.42163982165384	2.48487560096219	-0.72080670306610
N	-3.72676687880254	0.55038069338316	0.27898789657309
C	-2.78803706336631	1.35454962604381	-0.30134310030953
N	-1.47288925995888	1.16481961046456	-0.57116505407611

C	-2.75311367496816	3.63718241703120	-1.31950433009704
H	-3.50061783051268	4.21851479524062	-1.86715115615736
H	-1.97166039107529	3.28153026696207	-1.99844006847784
H	-2.30491617336867	4.24984011932241	-0.52778384079619
H	-5.44430982797236	3.19764347366080	-0.62052475822088
H	-5.83972018396685	0.73133697451113	0.61144439152781
N	-0.90976041220205	0.17789849647720	0.02264429824390
C	0.24749269147857	-0.30426702132226	-0.59233559721831
C	0.99077578810924	-1.23678938072605	0.15916030507917
C	2.14402348871101	-1.79997363431429	-0.37892100213883
C	2.55316749026155	-1.44708485420915	-1.67037900948897
C	1.80615941798861	-0.52888801127866	-2.42608592170176
C	0.65841075649334	0.04682857104433	-1.89689017475556
H	0.05689293657200	0.74413450458930	-2.47877668836079
H	3.44949409927449	-1.89772225164397	-2.09860539972211
H	2.12353831817784	-0.27451422047977	-3.43818667938790
H	0.64378904305930	-1.49675835625245	1.15994733628561
H	2.72296423080191	-2.51818573459884	0.20216205452888
C	-3.51604741003839	-0.76956913173445	0.87250061424610
H	-4.49876352460034	-1.23356108945944	1.00218751839111
H	-3.01224354057133	-0.66559629384035	1.83944353675216
H	-2.89288590287912	-1.37390239962904	0.20551484555519
N	-3.62033962883154	3.14520343525987	2.66219894661673
O	-4.44173409759946	2.49512375867677	3.37375911890454
O	-2.50389906323542	2.62772583903181	2.35019007228150
O	-3.91144636549258	4.31084341702073	2.25975084078451

165.05°

C	-4.77343026715921	2.41843510610296	-0.34569032151287
C	-4.97450046022085	1.21884499707744	0.29366018868454
N	-3.46595986770036	2.45193757960882	-0.74216773643630
N	-3.78390057419918	0.53978197932183	0.29407664897746
C	-2.84986983074020	1.30726822718996	-0.33959432316848
N	-1.54933350545773	1.07864082306522	-0.66095515972226

C	-2.79235112969259	3.57581010038587	-1.38861161271546
H	-3.54798335327444	4.17394245956084	-1.90616434333922
H	-2.05749548430346	3.18878652932041	-2.10105497984921
H	-2.28675071861216	4.18319992499420	-0.62805179313167
H	-5.45535618656984	3.23554473856525	-0.54491528022376
H	-5.86895044287429	0.79550248888043	0.73429068511213
N	-0.99383786260562	0.08824355467339	-0.06849643145965
C	0.21338220079200	-0.33661227413480	-0.63458289380388
C	0.91844543025139	-1.30369910186122	0.10859720705422
C	2.12203140343171	-1.81003602359250	-0.37463721511515
C	2.62028342390145	-1.36337829317686	-1.60361040314621
C	1.91289301261631	-0.40780741733241	-2.35147475774855
C	0.71488110569433	0.10962893125957	-1.87645895904076
H	0.14787389783339	0.83834360750516	-2.45428524766703
H	3.55747568050854	-1.76669671238219	-1.98942480481062
H	2.30332998274017	-0.07670215007690	-3.31463647319081
H	0.50245723340023	-1.63571801570591	1.06058675249108
H	2.67068472855569	-2.55570098650426	0.20123141745341
C	-3.58750801714270	-0.78117265873171	0.89074474094046
H	-4.57774492775290	-1.20244026927372	1.09029473443263
H	-3.01784743308379	-0.68607927803958	1.82126401336612
H	-3.03478280042217	-1.41850864776052	0.19345528227817
N	-3.47195338561709	3.15136642460348	2.64037698914968
O	-4.27581162780891	2.53437774139186	3.39990439351490
O	-2.38971134571122	2.59572860711494	2.27729714736900
O	-3.74636887877568	4.32209800795054	2.24116253525820

172.28°

C	-4.79923252349287	2.41699719190447	-0.32211499969485
C	-5.00250087275185	1.24529411330510	0.36656450113123
N	-3.51169590714967	2.39888027395810	-0.77934062823756
N	-3.83393840426995	0.53085361494836	0.33206341730439
C	-2.90990749278517	1.24962102072362	-0.36953995220319
N	-1.62212152151245	0.99223689738534	-0.72564285159432

C	-2.84184474497201	3.48140335543600	-1.49795592153394
H	-3.60775286380729	4.08650301910052	-1.99164396117204
H	-2.15975135700599	3.05232446467017	-2.23742004909826
H	-2.27818500064248	4.09560792569358	-0.78516932338239
H	-5.46773170040118	3.24558739932678	-0.51919664664834
H	-5.88473854968409	0.86608470012681	0.86782477120805
N	-1.08960711211629	-0.03538850630436	-0.18042003378410
C	0.17142785189126	-0.38203363719556	-0.68374725849232
C	0.80585665812139	-1.44974794752741	-0.02019285628190
C	2.05948073020286	-1.88514178980196	-0.44250414479193
C	2.68096877707989	-1.26256775354291	-1.53014045179265
C	2.04654225127982	-0.20151932880696	-2.19747786785483
C	0.79825050592230	0.24320565806216	-1.78291742940395
H	0.29228575830972	1.05888690209390	-2.29773359282174
H	3.66044215950577	-1.60507174630743	-1.86644581382447
H	2.53602455485412	0.27247532584252	-3.04922635639212
H	0.29601556233159	-1.91745722269370	0.82292913139717
H	2.55233914051304	-2.71029087167288	0.07220515306291
C	-3.64198521397508	-0.76832079327958	0.97699340022958
H	-4.62221732808532	-1.11035204344392	1.32317525004203
H	-2.95417290871257	-0.66091400972826	1.82219954559201
H	-3.21989477448079	-1.47713222421700	0.25776290566503
N	-3.31855578335681	3.17381900597491	2.59049761527917
O	-4.07659790107431	2.58111760367443	3.41378002635182
O	-2.26498837428159	2.60112560937736	2.17379987331565
O	-3.60992361545315	4.33684379291742	2.18116454842600

179.51°

C	-4.82595821598548	2.41677120489636	-0.29177750101868
C	-5.01844309883835	1.27053253526912	0.44142712065897
N	-3.56777127243822	2.35166294835406	-0.82052152658905
N	-3.87341021643202	0.52296021576574	0.35838469857228
C	-2.97308930880883	1.19761050518345	-0.41427728062648
N	-1.70097588606056	0.91630648017656	-0.80912430092943

C	-2.91442417236699	3.40046850266087	-1.60258363024186
H	-3.69375870780235	4.01380611637993	-2.06408670282636
H	-2.28804794389727	2.93999289740081	-2.37133201253728
H	-2.29618966581726	4.01559070566996	-0.93738631273851
H	-5.48297215732343	3.25793625994641	-0.47365888468067
H	-5.87795322626769	0.93171850777857	1.00693295173080
N	-1.18313085125775	-0.13693601777131	-0.30147120291110
C	0.12281058055322	-0.41550829116155	-0.72838258074133
C	0.70431533258580	-1.56122279130569	-0.15275377111557
C	2.00042749618006	-1.93399057469719	-0.50088918318773
C	2.71903105875005	-1.16717074900360	-1.42378610703127
C	2.13977663467032	-0.02432998970398	-2.00043438361503
C	0.84871283666565	0.35678796711758	-1.66044355218994
H	0.38847280944346	1.23987016349098	-2.10158206203854
H	3.73418038458094	-1.45658616109182	-1.69862395853037
H	2.70806234594610	0.56651525759980	-2.71995335076575
H	0.11973792656270	-2.13913473471110	0.56400263824176
H	2.45175774780087	-2.82053749339464	-0.05476475531102
C	-3.67867270225829	-0.76368754491334	1.02754144254591
H	-4.62937531748315	-1.03524421086709	1.49660213640608
H	-2.89250429051945	-0.66882013903136	1.78375894918328
H	-3.38338193391324	-1.52083525473690	0.29452006303612
N	-3.11967635950411	3.17779456952541	2.51992108048342
O	-3.83409612693222	2.62162294068598	3.40537604380731
O	-2.11216229457948	2.56899503830091	2.04443459688225
O	-3.40900140525217	4.33999113618664	2.10706133807790

Isomerization of 11

-11.22°

S	2.33637724033957	2.05543001709185	31.98172519663951
C	1.13487089621108	4.93835900088765	20.37868660495680
H	0.68162845209090	5.87760526381020	20.67273966236026
O	1.38763801544611	4.75945346987743	23.53962685076227

O	2.06847637356252	0.96026578100350	31.01877099915961
C	2.37918537157136	3.43562786528060	19.33195347643740
H	3.09405394600435	3.06671289759649	18.60692266179415
O	1.27066216710748	2.21073910406020	32.99601455466852
C	1.65131135291968	2.78542052773775	20.28807143057958
H	1.60974749432097	1.73925059107686	20.56566839953677
O	3.69722638567766	2.00003551013664	32.55949337739982
C	-0.00180232979955	3.51030663775708	22.07534889938889
H	-0.51969367571610	2.55562621956696	21.92576821497986
H	-0.74387098524938	4.31676233076247	22.09234962815681
C	0.77887138502230	3.46981936295853	23.37612569296272
H	0.08176294793633	3.25953080002903	24.20231263885641
H	1.55278475041527	2.68661303574548	23.34913094748650
C	2.19448311435541	4.95805716564703	24.62019278623421
C	2.85063448755233	6.20003507624956	24.67303047704632
H	2.69566881407976	6.91581416405507	23.86535294027095
N	2.04076575118524	4.77500621983953	19.40620556416134
C	2.57659227006912	5.84068997317000	18.55758078368149
H	3.66200712929897	5.89919536966431	18.69620913360433
H	2.11152916394624	6.78647864068157	18.85006724804455
H	2.34480856261848	5.61796010203805	17.51002653252623
N	0.87822750385418	3.73955625988428	20.92431091343164
N	4.79216209999935	5.92881524756846	27.79934649111591
N	4.63459352629032	5.63722618638991	29.01508298796802
C	3.70012156351072	6.48618229616955	25.73076338086865
H	4.23589833716494	7.43560874643034	25.76888918997720
C	3.85772069489477	5.57076454662542	26.78661490131605
C	3.21690568778173	4.32280460764681	26.71682028560302
H	3.37204161207894	3.58041061998392	27.49795417122825
C	2.40258581834420	4.00698545747285	25.63338281222311
H	1.93411871402836	3.02554459143734	25.58942899063354
C	3.40047782000941	5.12735809093671	29.53244744109004
C	2.18900951466729	5.81073396466048	29.33515153444474
H	2.16073791109166	6.69494791722412	28.69870091792180

C	1.03606612569750	5.35782104449607	29.97288365677491
H	0.09731294495068	5.89629666758741	29.83508817188735
C	1.07185477655760	4.21964294743318	30.78482124205767
H	0.17017913960482	3.86057245605947	31.27999604040885
C	2.28624889966080	3.55508846136609	30.98572820053331
C	3.45735151771564	4.02113704596000	30.38770165564702
H	4.40886671113085	3.52064771794346	30.56733231317213

-26.47°

S	2.47557958321285	2.03222639975707	32.10315703224413
C	1.19967584770857	4.93093984967458	20.30000835609367
H	0.74095702870534	5.88113751217444	20.54644085516320
O	1.22839003091082	4.81975211244411	23.47400470053951
O	2.12667908486946	0.93086686990458	31.17408348512461
C	2.49037206012267	3.39357118542423	19.36588099766411
H	3.24786404465734	3.00239867874466	18.69806616115368
O	1.48109328608401	2.21737496161417	33.18278728409941
C	1.68947902648794	2.77005018747239	20.28034258740041
H	1.61476512261881	1.72951319519698	20.57183113664364
O	3.87026869403836	1.95946893777061	32.59106667666401
C	-0.06437251505381	3.54802043924278	21.94396566017353
H	-0.58327119686328	2.59623711611414	21.78007660689677
H	-0.79619821224189	4.36300330167307	21.90020216467407
C	0.63844811899145	3.52358295282149	23.28888596462172
H	-0.10065577315347	3.31640308445421	24.07840173114528
H	1.41749673097466	2.74568947129174	23.30980850343633
C	2.02526996471712	5.01895720139284	24.56018316845044
C	2.65968249669182	6.27182433148835	24.63086394024425
H	2.49093301683500	6.99624418431420	23.83375098955208
N	2.16731759833892	4.73841407872670	19.39463592630696
C	2.77604426817396	5.78138356029142	18.56738463656277
H	3.84915576445263	5.83137371004404	18.78351433570194
H	2.30206229514693	6.73756968380048	18.80704700895034
H	2.61833134296522	5.54032951827922	17.51017932918930

N	0.88876344514491	3.74538327747949	20.84634957143697
N	4.64138672861885	5.96670532279140	27.72303120153780
N	4.61673694673527	5.49104973786483	28.89416260462144
C	3.50503623108536	6.55577228303321	25.69095174260522
H	4.02470770322095	7.51349092474750	25.74291306103045
C	3.69210284605827	5.62300894572377	26.72982329910721
C	3.06217255283705	4.36796905636229	26.64542704093299
H	3.23163704768934	3.61528036018607	27.41370094297314
C	2.24752783847128	4.05786762715822	25.56241436251020
H	1.79144010170608	3.07117477208940	25.50601716652675
C	3.42003405026229	5.03262505655983	29.51430796674147
C	2.21852589017648	5.76033173145020	29.42298183368268
H	2.16285423797261	6.64053399408601	28.78294494017890
C	1.11783599503649	5.36266419145398	30.17662678694555
H	0.19306699218666	5.93916024821211	30.12368598349127
C	1.18556742900473	4.23329071541204	31.00054343436112
H	0.32318954650664	3.91906400953981	31.58745185243070
C	2.38616108096585	3.52136775681879	31.09354381260563
C	3.50998184813280	3.93125030602916	30.37696338992737
H	4.45410377879650	3.39461715888900	30.47140976765466

-41.71°

S	2.50866995964063	2.10153371333559	32.33837203894147
C	1.21065029772869	4.94508991975216	20.21176114554985
H	0.72932942695977	5.88147633566985	20.46784679757382
O	1.23679100409416	4.78202344421902	23.38391820551839
O	1.80194377172454	1.03247725212293	31.59274885387858
C	2.54082769730485	3.44961918608991	19.26528133014279
H	3.31052452997907	3.08441013187076	18.59669760207899
O	1.81485196972962	2.49102737001798	33.58640137345754
C	1.74930251340659	2.79614794484925	20.16701702336895
H	1.69639112068050	1.75049694663573	20.44465933503333
O	3.94960233312504	1.82510012022747	32.52601692313254
C	-0.03238349570233	3.51448062782969	21.82933428445096

H	-0.53297984052411	2.55599381689211	21.64829724644373
H	-0.77876016184229	4.31635799653790	21.79193763714618
C	0.65954354331933	3.48247562941465	23.17965702386648
H	-0.08509120972711	3.26098065710610	23.95988549765105
H	1.44594331556520	2.71209164136360	23.20066840203137
C	1.99964214603327	4.98420126207982	24.49224638932311
C	2.61788561929121	6.24430665836862	24.58802149476673
H	2.46168515589242	6.97182977730760	23.79117700748356
N	2.18713173755844	4.78639492203120	19.30952786806226
C	2.77561396828565	5.85441056259958	18.49966539175523
H	3.84594308627674	5.92722221625664	18.72305360652703
H	2.27743082996365	6.79575604569016	18.74894923963864
H	2.62994250159323	5.62352780959515	17.43847629828375
N	0.92388947912750	3.74586980355517	20.74125821481305
N	4.51874816217157	5.93000021689313	27.72370843140478
N	4.55926895347831	5.32090259443675	28.83839829790011
C	3.42786748738101	6.53182442949310	25.67292301378304
H	3.93175814545158	7.49638997924880	25.74792648666916
C	3.60503165418262	5.59023598980639	26.70926094887736
C	2.98505921260844	4.32923591027454	26.60120238460766
H	3.14289809377509	3.57426647947350	27.37017076887090
C	2.20461972947610	4.01850973838284	25.49550469659350
H	1.76208613023829	3.02700360893899	25.41816952517900
C	3.39557030769290	4.94258907997096	29.54450756064388
C	2.21440584155069	5.71119137059746	29.50221101140984
H	2.14561705616255	6.56667197929814	28.83090395948517
C	1.15813614575886	5.39402756786396	30.35119868791987
H	0.25568819083541	6.00723820356006	30.33746924795474
C	1.24067210465518	4.30227083803604	31.22244636335724
H	0.41056025613630	4.05666320004661	31.88423488095328
C	2.41689544983680	3.54264982118086	31.26148007928954
C	3.49788083939809	3.86944269234336	30.44804594056763
H	4.42111493972574	3.29252450873533	30.49918148361096

-56.96°

S	2.69474242588251	2.20163865583694	32.65266336659185
C	1.27586452798534	4.97623717275806	20.09480415233534
H	0.81221099697449	5.91913967213280	20.35937285312473
O	1.19928074537281	4.77876552942341	23.25529025921243
O	1.78991109101195	1.14292570296609	32.14445092565692
C	2.59032027435010	3.46682061799773	19.14850441667648
H	3.36675758812181	3.09594154100918	18.49093173929890
O	2.26966496295219	2.74397292697955	33.96303202372608
C	1.76079910768712	2.81542558973337	20.01694887755915
H	1.67482655334772	1.76594885789581	20.27084942200322
O	4.12315549140835	1.81954650185187	32.60967464635396
C	-0.04158439349143	3.54535053210253	21.65165844686419
H	-0.55205433924549	2.59705228671965	21.44637274328567
H	-0.77447267237254	4.35915121067907	21.60495828972192
C	0.61341817343151	3.48746493123849	23.01939720226182
H	-0.15406001461313	3.26587285474785	23.77686252572975
H	1.39129912432512	2.70901383543929	23.05199305977373
C	1.91580589133204	4.96833502148839	24.39410684881130
C	2.52861354310736	6.22902546026486	24.52822609539889
H	2.39897298814897	6.96593378408778	23.73527608783980
N	2.26985241529334	4.81117827701648	19.21313174568385
C	2.90602396222559	5.88075786328203	18.44217263211006
H	3.97160839021971	5.92132287176226	18.69447807851018
H	2.42511527580490	6.82899879013282	18.69916280663804
H	2.78236783510548	5.67637933713795	17.37280057399735
N	0.94620077184936	3.77409049061603	20.59173533734958
N	4.31056296097577	5.87066870286052	27.71754100871749
N	4.41280104822969	5.15117448080088	28.77097029363125
C	3.29606968415565	6.50492770823001	25.64426098858766
H	3.79252897183650	7.47010719799007	25.75237224688795
C	3.44583729181567	5.54518642942625	26.67359823415454
C	2.82297427555320	4.28498004339628	26.52865795648173

H	2.95686875848970	3.52186524379985	27.29462587939292
C	2.08333148993617	3.98928228276880	25.39371172967994
H	1.64458055750613	2.99891236421055	25.28555527543235
C	3.32653041755467	4.84119456262455	29.59382382149187
C	2.14457819626536	5.61691957262596	29.61750515259840
H	2.01631239992611	6.43184467429261	28.90572322277907
C	1.17758046430199	5.36870224943670	30.58595729585504
H	0.28258429924832	5.99202354086909	30.62207385098247
C	1.33817367449352	4.33536545461285	31.51626544353642
H	0.57719445313023	4.14770852180408	32.27328321505312
C	2.50712141141716	3.56097771153721	31.48529273406529
C	3.50077634826297	3.81522246434533	30.54818649013230
H	4.41715258068576	3.22558647906748	30.54356000402161

-72.21°

S	2.91752768456072	2.34179048028416	32.95073537621670
C	1.37212512919765	5.01221255251761	20.02419177918946
H	0.96062164485868	5.95982410630524	20.35066245441762
O	1.14918746044253	4.74431532962522	23.11754765851337
O	1.83750119872508	1.34361026687985	32.76471825284935
C	2.61209939588207	3.49831157888931	18.98837969380154
H	3.37770512246341	3.13007258448977	18.31677907675284
O	2.83079753987280	3.04806820065497	34.24978736359289
C	1.72898288383593	2.83706382282620	19.79443066336177
H	1.57571294588659	1.77925695438261	19.96981432768665
O	4.26553052332895	1.80382363131232	32.66481514905699
C	-0.05526865274610	3.55526960818132	21.45439066503689
H	-0.56549689932706	2.61541999162500	21.21373985460371
H	-0.78223263927881	4.37456491880434	21.40809293019160
C	0.56268968326886	3.46069282152205	22.83748034366540
H	-0.22511169336966	3.22861611588441	23.57007856203834
H	1.33674559853248	2.67877896147119	22.87238459885483
C	1.81771457799148	4.91776789758901	24.28457998260556
C	2.41836966495015	6.18159259026342	24.46030466559662

H	2.31026718378847	6.92947037856141	23.67447903837176
N	2.37111991481432	4.85128835705202	19.14766225470954
C	3.08528950507528	5.93365077465687	18.46841344577122
H	4.14830425594221	5.88186048459540	18.72926397535359
H	2.66577257840592	6.88841379328350	18.79843792391338
H	2.95983733335632	5.82415845052405	17.38538498270177
N	0.96186711811948	3.79907018878309	20.42623495899528
N	4.06606305351517	5.77965080400098	27.70268068081824
N	4.20690319549068	4.98780570823200	28.71099965577145
C	3.14285148215072	6.44475442366503	25.60440643609386
H	3.62625901207750	7.41168054547019	25.74884711801617
C	3.26623704323716	5.46476529132836	26.62497200426106
C	2.65012425618238	4.20091734885673	26.43768523355781
H	2.76824139463708	3.42795524780805	27.19695377261792
C	1.95405567959319	3.92126299722419	25.27421991360402
H	1.52654130865718	2.93047756246292	25.12987976864968
C	3.23167158751586	4.75469529268418	29.65695477708947
C	2.05736097380591	5.54620395057986	29.76019919413242
H	1.86265880935402	6.31981076908977	29.01769681874100
C	1.20210093093918	5.37473734734382	30.84238984088477
H	0.32164682021077	6.01332013344270	30.93487515214239
C	1.45333332645103	4.40081101487432	31.81651651551067
H	0.77939601788208	4.27659952314411	32.66365601169947
C	2.60525040310766	3.60404354951980	31.70411028768444
C	3.49010900116692	3.77563978464445	30.65031789605694
H	4.38973664544748	3.16484386466349	30.58166894481545

-80.00°

S	3.00432341872890	2.48662617456625	33.04691934200289
C	1.39339410863002	4.89471039806137	19.90829727583698
H	0.84808953879739	5.80033029686864	20.14607963217494
O	1.10701081574360	4.63853059412090	23.04594583425765
O	1.85133598830308	1.55472197856231	33.03624778439355
C	2.87742092160013	3.49271553829481	19.05162406917462

H	3.72536436463318	3.18535858213008	18.45212689962336
O	3.12628742508725	3.23607810765044	34.31955767193546
C	2.03084279904718	2.77322792386961	19.84670497790082
H	1.99837595456116	1.71656934555371	20.08272544519114
O	4.27090313855971	1.85338821398960	32.61772178786956
C	0.06198232684886	3.35398448273789	21.34389641639559
H	-0.36397148816947	2.37591386193237	21.09041253521621
H	-0.72006126321699	4.11612090313398	21.24839650586079
C	0.61349590781076	3.31741059497230	22.75706357952490
H	-0.19349019168724	3.04696483169093	23.45455895035840
H	1.43137859469076	2.58594412261009	22.84307078166577
C	1.72882254350766	4.85939305807196	24.22846526774317
C	2.25613719356849	6.15685486359512	24.40653300594663
H	2.12729960171217	6.89002502943719	23.61010297545943
N	2.46102588208989	4.81098635930124	19.10482652226349
C	3.07977853030613	5.93275526097777	18.39628008855615
H	4.11949838269515	6.03697602537000	18.72620532773108
H	2.52065777853181	6.84349017466782	18.62947679869290
H	3.04720189263243	5.73945395227705	17.31821361259585
N	1.11023739045204	3.66541613608607	20.36626805712117
N	3.83302865461860	5.86726457136487	27.69035550815168
N	4.00740187098232	5.07152560626144	28.69467087963271
C	2.93783475821082	6.46812009284075	25.56302244650494
H	3.36548766958623	7.46048935435259	25.71036034070200
C	3.08819807060453	5.50292752550689	26.59723633096197
C	2.54272184839988	4.20466819949963	26.40729218869581
H	2.68859128223784	3.44667021014661	27.17688525034311
C	1.89188764014863	3.87886555239845	25.23158239529826
H	1.52182064835361	2.86547271678056	25.08556077753840
C	3.11232501201562	4.85050359975153	29.70259923131695
C	1.93464570918705	5.63433550397013	29.86449406844685
H	1.69244094223756	6.39385175748895	29.12111781232743
C	1.15170765742819	5.47923924143893	31.00219935603321
H	0.27497068422192	6.11573735668483	31.13661946302618

C	1.46669929659248	4.52443539925180	31.97661385793355
H	0.84573994115504	4.40935541474271	32.86431967298662
C	2.61318686696033	3.72817614488160	31.80207495052152
C	3.43259191782543	3.88473085628551	30.69639773867152
H	4.32357797376957	3.26862408582203	30.58069658541122

-87.46°

S	5.97457209611389	3.43697060001531	32.42688862229573
C	1.17248692745002	5.25245965385359	20.31313268551675
H	1.41146071794556	6.30722276694983	20.38081785541696
O	2.18031291851600	5.54725963707963	23.30509334862995
O	4.66628542369194	2.76196800864456	32.60615972989243
C	1.03050869312379	3.16675433755108	19.58361017304262
H	1.18175791988235	2.33876257644135	18.90210413100191
O	6.30662665058663	4.35114114412001	33.54509697957671
C	0.38369500817694	3.23526872690447	20.78485899355759
H	-0.13501807967334	2.47858980515473	21.36080078745900
O	7.07141569985463	2.50322458447303	32.08874317681776
C	-0.00529763481841	5.06418336208538	22.50278513382934
H	-0.95574125242394	4.56917110573491	22.73418454200043
H	-0.18686164622972	6.13878786714958	22.38458230802114
C	0.98467069764375	4.80376096148075	23.62185669977060
H	0.55931288423027	5.15765735911445	24.57277110802525
H	1.22426786676825	3.73316747706856	23.70127570699678
C	3.26010866812313	5.41537167116411	24.10027440125998
C	4.41469692444582	6.12420404473294	23.67032277006869
H	4.36001643247829	6.69559629573417	22.74368936905117
N	1.51293042727689	4.43401418696075	19.30987319105691
C	2.26292759530323	4.82194838283661	18.11391177394638
H	3.17667904948504	4.22036379785344	18.05322667441916
H	2.51828865928974	5.88278723482812	18.19095818303990
H	1.64197296207171	4.64978066694825	17.22759417964301
N	0.47912756959629	4.54517718126553	21.21938838129009
N	6.68778428685582	5.47456222080157	26.42941504360110

N	6.76715102050780	4.70163461800490	27.49392445468423
C	5.55863281450263	6.08415424167330	24.42314612247569
H	6.45228344243711	6.63085276651212	24.12069014541289
C	5.59754243822879	5.33897305280187	25.64456326061984
C	4.43711474561353	4.60400404605740	26.04515087788268
H	4.46618797651865	4.02908104809581	26.96846928205817
C	3.27812558038414	4.66294453144257	25.29207347308180
H	2.38627081887162	4.14177583970315	25.63537287690328
C	6.22354927523359	5.05688410831005	28.67079653343730
C	5.33834094393136	6.17894307678943	28.78263778525948
H	5.16664392397416	6.81294895849760	27.91483354112440
C	4.65992232591587	6.40772975719626	29.97851417716340
H	3.96544033206022	7.24793769337854	30.04142426965410
C	4.85202459980724	5.58846586307871	31.09127492554941
H	4.31588131748077	5.77307717174866	32.02129071960517
C	5.75371872171793	4.50207714534984	30.99314192860940
C	6.43351615245372	4.23644892140009	29.82400652803504
H	7.12045610459549	3.39303150301198	29.76367314921089

-96.49°

S	3.08518029170998	2.64506955117194	33.08769124182107
C	1.41140409330601	4.90974952711764	20.02163345970204
H	0.96918478876252	5.85925501232123	20.29876064878630
O	1.14485613400922	4.73842021108222	23.13028990580768
O	2.17593100770092	1.47896445852018	32.98912761306104
C	2.71147298263326	3.38638177932688	19.07752534500801
H	3.50084262027938	3.01029396810555	18.43855851466937
O	2.84575632815023	3.46062420874495	34.30176994726057
C	1.82680311109412	2.73666725059842	19.89111214769000
H	1.69601459130678	1.68332161001531	20.10702193308211
O	4.50864862410449	2.29639735214939	32.88757222435727
C	-0.00938149309264	3.47761392839229	21.48285099317804
H	-0.50245928391695	2.52333634723536	21.26405784508117
H	-0.74725334700438	4.28402414067905	21.39874804447725

C	0.58622778267915	3.43433609033588	22.87788258901223
H	-0.20821643872827	3.21203649441858	23.60610692212812
H	1.37463408632762	2.67022334653926	22.94950471507311
C	1.83506529633364	4.94362865044481	24.27366937709886
C	2.42605939468606	6.22738032825601	24.39791171491112
H	2.30847786202101	6.93749267625604	23.57918482975406
N	2.43376845892110	4.73823629039750	19.17465101414357
C	3.13879428320946	5.80970190087862	18.46886232249246
H	4.19357234600718	5.80187119210015	18.76575327967119
H	2.68125226703847	6.76542562500049	18.74021838162495
H	3.05283369434533	5.64683897268726	17.38874662977371
N	1.02219027322421	3.70418886253857	20.46537317909907
N	3.81876996501400	6.02748071583228	27.75739462155321
N	4.07853677638461	5.11101389313298	28.66672699797894
C	3.12018960428067	6.55421766212449	25.53561955876474
H	3.57340329482258	7.53900258154010	25.65333285890872
C	3.25212936531719	5.61023910860350	26.60037309508854
C	2.66921239619867	4.31583541255342	26.45077525245576
H	2.75203998672889	3.60118468377229	27.26770706643516
C	1.95411795318013	3.99578752574758	25.30834983125029
H	1.46991573494534	3.02370052153096	25.23155628957652
C	3.19334553298515	4.90302782141725	29.66911724213310
C	1.85577561982966	5.39960798195438	29.60571904548608
H	1.55788615184753	6.02533158023368	28.76630821451941
C	0.93293019293198	5.02150660859272	30.57911580842921
H	-0.09565034634184	5.38014636666168	30.50807032264984
C	1.29938202681759	4.18781438570652	31.63670507340607
H	0.56855450850118	3.88954456595690	32.38786939303931
C	2.62870430547925	3.71334117651252	31.71257716154464
C	3.56343118614762	4.05763838443164	30.75832943247215
H	4.58386598982148	3.68103924838072	30.82158791553938

-102.70°

S	6.25193126496934	3.49495715471990	32.59401236450531

C	1.17241792168531	5.26551210562293	20.18250013898170
H	1.37382542810265	6.32541508339620	20.28308760856452
O	2.02583681170322	5.50032309722188	23.20986274650557
O	4.90685617050763	3.05930331280587	33.03805032581650
C	1.14108361236419	3.19106779689350	19.40947686638433
H	1.35850517744719	2.38142638936052	18.72380378792387
O	6.92813700637110	4.37183405770025	33.57835148020507
C	0.42443070944141	3.21610079227697	20.57235877436584
H	-0.10100406763150	2.43248996326769	21.10451011449261
O	7.10131086437319	2.38076627646731	32.12007354947675
C	-0.11112939314840	4.99821198917604	22.30146247656218
H	-1.05975669130876	4.47650675748023	22.47462963937659
H	-0.31161582641725	6.07071776191399	22.19515228648352
C	0.83055431672844	4.73760466838283	23.46222959347722
H	0.35360006307087	5.07115835513743	24.39634561134227
H	1.07921794340013	3.66841719192322	23.53979565485555
C	3.07441258706479	5.36327681239426	24.05443105944536
C	4.24463123079764	6.07164253308700	23.68522560769484
H	4.23670586422590	6.64760464284995	22.75962504633051
N	1.59689051032820	4.47808129982395	19.18648697521278
C	2.40335402452439	4.91387927620118	18.04503731466071
H	3.33363526072697	4.33541741034907	18.02194918433033
H	2.62807235039414	5.97785139532677	18.16274938143652
H	1.83839183585356	4.75040220831189	17.12038122819836
N	0.45151590984551	4.51967065283377	21.03434126279640
N	6.41407912137884	5.37007636931238	26.53345018055098
N	6.46469375179272	4.53357752957149	27.54320705173096
C	5.35546958699313	6.02937605533115	24.49304714219186
H	6.26209105962683	6.57430536718538	24.22794405277050
C	5.34152782030374	5.27491125058337	25.70376724764493
C	4.16224721390450	4.55665394257931	26.05370633215922
H	4.13916001389439	4.00509870324703	26.99221827925256
C	3.03776609859191	4.61362056474439	25.24585028823938
H	2.12829332555661	4.10095204459632	25.55455997088349

C	6.13736928407536	4.99062229265232	28.78150556600760
C	5.41571666210835	6.20550423197670	28.96524715770807
H	5.18063764809751	6.82059622103170	28.09809670482797
C	4.96249219573712	6.55324205373129	30.23601388314640
H	4.38753555528909	7.47174374940654	30.36699196231415
C	5.22216623731296	5.73940908901723	31.34021692022546
H	4.85450866115209	6.01270410938587	32.32899816171764
C	5.94891701229943	4.54245064679756	31.16159798868756
C	6.40455676362704	4.16726003730911	29.91308914476376
H	6.96075110283778	3.23937675661530	29.78296188574732

-112.98°

S	3.37470207234614	2.71320643300211	33.25892202183469
C	1.50844587216717	4.93332512022102	19.86529613424196
H	1.03113848417045	5.87725308346659	20.09996108046982
O	0.99219087797973	4.72135979576342	22.96075267292004
O	2.31166817144142	1.70153816928686	33.46147602879395
C	2.90778075639639	3.42613266180781	19.04506188502797
H	3.75517337085460	3.05947269161578	18.47903770251460
O	3.53645415925828	3.62093039242837	34.41913708578260
C	1.96446674692814	2.76601873432600	19.78051760608388
H	1.83009218489266	1.71141020131978	19.98792716855259
O	4.65090060595177	2.13721766450709	32.78278654431138
C	-0.01385216156799	3.48236220499411	21.20285720353882
H	-0.48459777068929	2.53034176204710	20.93078091233535
H	-0.74445694933053	4.28861658478221	21.06839445966974
C	0.46555505580568	3.42073864555167	22.64116617889512
H	-0.38409827308165	3.17967886015203	23.29820064233785
H	1.24869369610156	2.65730792907977	22.76300503328032
C	1.62806621427191	4.89106141637477	24.14585344245840
C	2.19880940273800	6.16875571776271	24.34949963906144
H	2.11357396083707	6.91337604019661	23.55780022806282
N	2.60366636827130	4.77409696065160	19.11190348980532
C	3.35308138371252	5.85273841643200	18.46529404909056

H	4.37910560573980	5.85741975057052	18.85001306503709
H	2.86357186514306	6.80352009502850	18.69488869801663
H	3.36051761815147	5.68708071109120	17.38219764072723
N	1.09861994479958	3.72350319716657	20.27777314586648
N	3.49128546843327	5.83378880222320	27.75323020552075
N	3.78163263794865	4.87449890150636	28.58652889400746
C	2.84009360963709	6.45288135731600	25.53417723138118
H	3.28079491984370	7.43493857246247	25.70946346692233
C	2.94056423290117	5.46755693918074	26.55562779982470
C	2.36769635700333	4.18724844789892	26.33498258493112
H	2.40882394073100	3.44667353640085	27.13263000059517
C	1.70590268242771	3.90633809621561	25.14902290104033
H	1.22868628663970	2.93685520582568	25.01676305495454
C	3.06283477042357	4.80253770143312	29.75076070181098
C	1.80055962157795	5.43677811296808	29.89423498871044
H	1.41625192693253	6.04437162758873	29.07610386315872
C	1.04484241979867	5.21562459229685	31.04387824558293
H	0.06338969840192	5.68308239669400	31.13986649794316
C	1.51901647278853	4.39067466059155	32.06607792029838
H	0.91560352900825	4.20943710941618	32.95544617793071
C	2.77461182121519	3.76614418205137	31.92662929206324
C	3.53989645885568	3.96141069016988	30.79102405626482
H	4.50644388214240	3.46963582813330	30.68486835833730

-117.95°

S	6.54510405380177	3.42874627263494	32.67420427310477
C	1.20626994658600	5.23223790723214	20.01889398340398
H	1.37983845794012	6.29748200090368	20.11478633720565
O	1.86438653509098	5.51177764802039	23.10580318464262
O	5.29421209654930	3.31935275028603	33.46019056920193
C	1.26305295082822	3.14664016153890	19.27735835288398
H	1.53766278304345	2.33214593340845	18.61848971026151
O	7.63266448329020	4.11386784629679	33.41155726032994
C	0.47821649397143	3.17299868285234	20.39529004745281

H	-0.06168959070062	2.38618455428752	20.90784769026326
O	6.96695404151423	2.15054676795105	32.06099885612568
C	-0.19721089759641	4.96542724766528	22.06185593540408
H	-1.14776473650289	4.43152983777513	22.17796606388541
H	-0.40553788613854	6.03386680361993	21.93267383728462
C	0.67104230804790	4.72785213354949	23.28309886399979
H	0.12508061691549	5.05482636444236	24.18135462898665
H	0.93242734664823	3.66319743541281	23.38202474176547
C	2.86373570403351	5.38669143672368	24.01439341731346
C	4.04301307237643	6.10801631476159	23.72223586120080
H	4.08791671500024	6.68926749586396	22.80095458698037
N	1.70519902772354	4.43966456622842	19.06187889039487
C	2.56568954573914	4.87549236504169	17.96060928465731
H	3.50759134169381	4.31691018464645	17.99866876521984
H	2.76179258404875	5.94561131737487	18.07311053278080
H	2.05706450409450	4.68602523724819	17.00863555785108
N	0.45153532293382	4.48322642533188	20.83795880700379
N	6.07390291985097	5.39000902037597	26.68052809043797
N	6.09316260368085	4.52002991579411	27.64547583183187
C	5.10470501851922	6.07260661401243	24.59993208614641
H	6.02005437440708	6.62781261726734	24.39142194638566
C	5.02750811676029	5.30955326855352	25.79629461340036
C	3.83898235681011	4.58814186049693	26.07524577675722
H	3.76400377001062	4.03819192623091	27.01254534543760
C	2.76369233827402	4.63453102131006	25.20052491253410
H	1.84279855318328	4.11158436156621	25.45309062387322
C	6.01319197227681	5.00155167420744	28.93083476258228
C	5.50971502529555	6.29469823523138	29.22134111932809
H	5.22311397071948	6.94901379308895	28.39907596497515
C	5.32636903067808	6.68154347079966	30.54727033042201
H	4.91609679462550	7.66843232636748	30.76772552607640
C	5.64324883311478	5.81566317420434	31.59647442757856
H	5.48169722632981	6.11860097678661	32.63094477142386
C	6.14447613597512	4.53183009954825	31.30806203526885

C	6.32819040622877	4.12256498702554	29.99872750898284
H	6.70863373232644	3.12517496603408	29.78004428694873

-129.47°

S	3.59628154953554	2.73281305731868	33.35020243539281
C	1.60895269744965	4.95630256002448	19.74625563583606
H	1.10474160811078	5.88941148633620	19.96761263493761
O	0.88430397553718	4.66622006144792	22.81042420449392
O	2.45848792147934	1.90634276782194	33.81496752046100
C	3.07624617671825	3.48235750906249	18.98627805610428
H	3.96094138161663	3.13671109738267	18.46593396524048
O	4.12188924210388	3.63695462638583	34.39961325877238
C	2.09943768743012	2.79698169343810	19.65137820003672
H	1.96719678886776	1.73710289652206	19.83137483969933
O	4.65022052788438	1.95083607789412	32.66833570057097
C	0.02455795247225	3.45717100372000	20.95578809871864
H	-0.41241537483556	2.50586738592203	20.62985106858612
H	-0.70741765298938	4.25669279641211	20.79175788935706
C	0.40806599881600	3.36632866506565	22.42093761734051
H	-0.47952762066732	3.08881909416233	23.01032574018168
H	1.19642278401364	2.61382665550771	22.57516525575852
C	1.44256289870992	4.80875735969561	24.04030475057011
C	1.98119663429375	6.08218844392773	24.32035895203504
H	1.93408722090411	6.85410154976254	23.55195043830769
N	2.74992610065344	4.82475701529067	19.05772526633141
C	3.52051391630114	5.92250519966717	18.47094226149771
H	4.52367085607328	5.93245044941262	18.91172875707250
H	3.00776144704723	6.86398043345968	18.68789336721184
H	3.58947877271200	5.77474114166277	17.38741032272938
N	1.19186181133371	3.73387288459606	20.11154145534717
N	3.12127946197462	5.64948950674811	27.77714332548663
N	3.40349006602888	4.66755019355139	28.56519873874966
C	2.54879847176124	6.33118976337024	25.55373196935325
H	2.96616066359678	7.31157023631487	25.78705401495975

C	2.60682496250447	5.31428171417779	26.53934513043939
C	2.05802899286714	4.04373364510927	26.24824756926955
H	2.06182952869085	3.27744226503921	27.02270211634541
C	1.47149771567093	3.79373175112472	25.01552750103608
H	1.01625342072044	2.82357352393398	24.82411897287224
C	2.89525892192361	4.73891942429458	29.85267937928578
C	1.79012232335176	5.55342215915379	30.19001828069827
H	1.34241209744372	6.18432944641508	29.42310568338552
C	1.25683563172347	5.49276461921149	31.47610188766546
H	0.39101426308445	6.10616275174557	31.73082655011613
C	1.80548652361249	4.64040933245697	32.43791632266066
H	1.37264312774330	4.58527847109114	33.43692409244878
C	2.90171346208187	3.82764208168163	32.09992410535003
C	3.44275958098563	3.86855373921822	30.82407483338159
H	4.28634548266212	3.23080146346178	30.56112183389917

-133.20°

S	6.81900302074355	3.31361041640435	32.63598521891222
C	1.30797031994119	5.16210305536330	19.92935710723200
H	1.50147293928250	6.22198650690277	20.04362521516433
O	1.67620437287329	5.55584009812153	23.02166327511817
O	5.80756242583291	3.45159484577764	33.70879133006790
C	1.37323365259155	3.07592448816566	19.18955478975153
H	1.68380884827917	2.25450938563654	18.55575710825006
O	8.17315582771745	3.74166563749665	33.05884332221259
C	0.49096825455564	3.12399566414902	20.23151492713073
H	-0.11524958377425	2.35260216936398	20.69043369152250
O	6.80206262070180	1.98896996671882	31.97847210082521
C	-0.28308830556843	4.93441766472828	21.83531624309723
H	-1.23132855089227	4.38730712519749	21.89203118703644
H	-0.49780716187132	5.99622055562420	21.66625319047252
C	0.49701636839792	4.73933026765974	23.12203168066117
H	-0.12347789771993	5.06100186408600	23.97264082529251
H	0.77850763730410	3.68358361705038	23.25585270511597

C	2.62106387877025	5.45415884347406	23.99219519750869
C	3.79352806224569	6.20823671356047	23.77970659722934
H	3.88037327586933	6.80083571265631	22.86875776671381
N	1.86948057865701	4.35598237589121	19.01929468229689
C	2.83812079150693	4.76650005780143	18.00126753586449
H	3.75406072995710	4.17706872329450	18.12008731106584
H	3.05877328225442	5.82911871920384	18.13633277785948
H	2.41023203699769	4.59676727854839	17.00681989869619
N	0.46074787047392	4.43433094255992	20.67390103128713
N	5.69262871247605	5.49458251528446	26.83377325169002
N	5.69239653998927	4.60068610997437	27.76063934142024
C	4.80152746399466	6.19058582789026	24.72334340526732
H	5.71216521396151	6.77278333351555	24.57629544906694
C	4.67483502971943	5.41073327761414	25.89884776400160
C	3.49259569765456	4.66375323830051	26.10293572511302
H	3.37669946059675	4.09989202181600	27.02795531567322
C	2.46925586254990	4.69072001076711	25.16539710100716
H	1.54954029598515	4.14090047638557	25.35845428532104
C	5.87262035251969	5.06040667947355	29.05906309768280
C	5.60772695944286	6.39568301848646	29.43664617716227
H	5.28448069663080	7.10717614739282	28.67766002484753
C	5.70811740209905	6.76535390741372	30.77654194974311
H	5.48236375486637	7.79120980582130	31.07202495138025
C	6.07488896279966	5.83155759693494	31.74923572753187
H	6.13454881999464	6.12200423673081	32.79822704089315
C	6.33293228154058	4.50238458084501	31.37306248641979
C	6.22882823148284	4.11334177455753	30.04565884304463
H	6.41724296656849	3.07970274535865	29.75615134634521

-145.96°

S	3.80273858137603	2.76257053746551	33.38268898547010
C	1.71228973434136	4.97914624738389	19.66884766640688
H	1.19293978929980	5.90583961426671	19.88204008035549
O	0.81540721011594	4.61316665548784	22.68599026302651

O	2.68418606954458	2.11047505953055	34.10157488097601
C	3.22362762386732	3.52575208972510	18.95697481641785
H	4.13616987229474	3.19376775242170	18.47735968012145
O	4.62125197710391	3.63592269032869	34.25576874067884
C	2.21531047967945	2.82369553836248	19.55419513896842
H	2.07754851005606	1.75989613934536	19.70435542691456
O	4.60785126932926	1.82280657435281	32.57290566578893
C	0.06872771442549	3.44955421506497	20.75553352687039
H	-0.34733409751823	2.50645020827907	20.38182430192131
H	-0.65425635027223	4.25257547557694	20.57013418063127
C	0.36854319424265	3.32266255807308	22.23733498101565
H	-0.55030557882659	3.02753995052789	22.76726794294842
H	1.14932191170878	2.56762904291108	22.41710032689928
C	1.28228452173626	4.72950546221691	23.95760107612569
C	1.78210603924797	5.99830008590498	24.30998205063499
H	1.77921155917063	6.79250467517093	23.56316988176109
N	2.88871955762521	4.86537148652651	19.03915466130244
C	3.68369350340233	5.97706990263605	18.51460153544630
H	4.66318995653633	5.98163698444210	19.00581349953739
H	3.15670369881032	6.91242223458181	18.72379692613048
H	3.80813901365217	5.85173033257985	17.43318412154149
N	1.28088107761748	3.74804097869748	19.98495814655446
N	2.71253309508597	5.48916319537498	27.82462113529775
N	2.96887317234580	4.49381351552162	28.58723797134780
C	2.25691395483623	6.21739910474828	25.59028425696157
H	2.64124425902498	7.19611652613946	25.88090936638433
C	2.26295172533211	5.17455280773368	26.54389542713156
C	1.75223387714156	3.91047866087387	26.18243792698298
H	1.71732299703329	3.11932886523169	26.93077692749008
C	1.25676673363231	3.68786495334945	24.90441786662671
H	0.83248838289359	2.71692089875777	24.65497761912350
C	2.69691339512410	4.68585504516469	29.94604534779486
C	1.78842644906151	5.65414748057445	30.41915030133608
H	1.29099586839106	6.31045948914604	29.70595898320596

C	1.50664918847480	5.72540082068057	31.78247026314519
H	0.79012985857268	6.46202545910780	32.14929914319548
C	2.11948754924744	4.85056786252542	32.68385303204197
H	1.88519401468316	4.90331162329153	33.74735822815830
C	3.01885759294694	3.88266065736429	32.20944977116101
C	3.30144618235072	3.79099786100236	30.85264416568850
H	3.98982486525420	3.03184268155067	30.48187376247456

-148.45°

S	7.02585362980243	3.18043262910626	32.51299562523428
C	1.40388915715905	5.09716737249882	19.86412734289517
H	1.57296878744309	6.16285392293243	19.96158367142634
O	1.49899133573911	5.60682645943920	22.95917496641836
O	6.33435537775001	3.47321044680233	33.78891591208270
C	1.55830160851497	2.99136534500183	19.19631702018934
H	1.93128908301929	2.15655636822871	18.61591850048488
O	8.49572041885423	3.35088031991852	32.59855571725165
C	0.59055251023763	3.05659922896316	20.15823944507815
H	-0.04219500773921	2.28966037185113	20.58784812813312
O	6.62126631591146	1.89402502646471	31.90545203711848
C	-0.33945906194393	4.89899267247844	21.63715182093193
H	-1.27903324260199	4.33419492807565	21.63461173372486
H	-0.55863723540961	5.95049045804574	21.41698951151838
C	0.33795252255163	4.75967325473866	22.98803432990115
H	-0.35715561979254	5.08529354475605	23.77731567733668
H	0.63437511798397	3.71577781799502	23.17230991052854
C	2.38283114211099	5.53407323410078	23.98977755878309
C	3.54570081508215	6.31588703071189	23.85039471172095
H	3.67620958292226	6.91110844915658	22.94642518995376
N	2.05022165002280	4.27338531029938	19.02906703578656
C	3.09386921963179	4.66834884589964	18.08152127376833
H	4.00097705803296	4.08890737518429	18.28595708173956
H	3.29679330302506	5.73555722211854	18.20882614666131
H	2.74737154488960	4.47292894630565	17.06037502494614

N	0.50619281889245	4.37852263216278	20.55674654134033
N	5.29865514999279	5.63192129585613	27.00169770759951
N	5.27219148138524	4.73164501399435	27.90928500404856
C	4.49310816150130	6.32189369118941	24.85802154777118
H	5.39660782667596	6.92654671937373	24.76802410691876
C	4.31446652780897	5.53617777855641	26.01827192842646
C	3.14136379369269	4.76526850909561	26.15207423578097
H	2.98471144117784	4.19256342882316	27.06572266628778
C	2.17669609927859	4.76793239591470	25.15300678400201
H	1.26172699219436	4.19371625565088	25.28873317595676
C	5.71520338870501	5.13939457694185	29.17386950751329
C	5.71405874637583	6.48551855575785	29.59081137706942
H	5.37894131319795	7.25638229139621	28.89772683602784
C	6.09630274855056	6.80321601175183	30.89285926918160
H	6.07640531830401	7.84265033166459	31.22398909743206
C	6.48795011116525	5.80077128370873	31.78498152478811
H	6.77300548404885	6.05146330702019	32.80685441423677
C	6.48246629059002	4.46084829882985	31.36889967304513
C	6.08901323889811	4.12622875357721	30.07895039918199
H	6.06971305436653	3.08426228766045	29.75998882977428

-162.45°

S	3.85754965022308	2.71249669861511	33.30256684300512
C	1.79831254220861	4.98125692810234	19.70585143885515
H	1.24353807285793	5.88106265248495	19.94308928577395
O	0.70387829910739	4.50609175193856	22.58931987336715
O	2.74411167409559	2.24232879805422	34.15908923775090
C	3.38957665814042	3.61015338568096	19.00553483836635
H	4.33956188828336	3.32992480618541	18.56738068873966
O	4.89735948444689	3.44815006106817	34.05853846513918
C	2.36321756892386	2.84932874831162	19.48991940963211
H	2.24349988588901	1.77540589605562	19.56456639668209
O	4.40679406012304	1.65901984646749	32.42187378453223
C	0.13279639444659	3.35509678990277	20.59521304505038

H	-0.21595069750552	2.40475706773704	20.17458978817646
H	-0.61059209654974	4.13156710969551	20.37922757125927
C	0.34117757647911	3.20765921773763	22.09119111898067
H	-0.59628128544640	2.86407801208541	22.55524461244812
H	1.14051824571135	2.48178510568587	22.30639942263529
C	1.08685377572407	4.61995772269645	23.88947231956267
C	1.51008960485254	5.90009872029786	24.29060473823053
H	1.51671442377537	6.70815038054467	23.55886439623360
N	3.01542781652963	4.93423624435176	19.15001760538966
C	3.81294937711940	6.09678282415510	18.75588795524844
H	4.76067329284867	6.08576752079075	19.30584812633120
H	3.25128512374928	7.00337474234242	18.99865542291112
H	4.00585857940299	6.05329136531537	17.67809407275944
N	1.37927445196505	3.72359797092401	19.91528299049717
N	2.29475834343395	5.37669859139144	27.83977843297611
N	2.50451280582174	4.38607796507785	28.61124811308659
C	1.90250130577705	6.11306574941572	25.60151198238870
H	2.22809551709140	7.10086818075041	25.93077707962015
C	1.89966089972635	5.05519595209821	26.53408085173762
C	1.46454731443710	3.77966060895785	26.12433157062791
H	1.43015259679507	2.96961749419292	26.85213878020504
C	1.05476038618184	3.55965456694095	24.81580953253680
H	0.69660850513333	2.57393231828353	24.52399497276841
C	2.51817075009602	4.69381878321331	29.98583833079723
C	1.94195942669752	5.85412592224573	30.53561124206511
H	1.46726148877948	6.57881030043064	29.87505394420738
C	1.96241389951364	6.04394804190736	31.91609375551157
H	1.50575223113672	6.93618073373086	32.34726576204572
C	2.55223753597197	5.09515398248881	32.75718185229437
H	2.56035638848190	5.24738606118888	33.83685369897967
C	3.11805860081052	3.93651324605240	32.20717604369736
C	3.09364837266430	3.72847192899076	30.83216368306585
H	3.52454926404796	2.82433920541420	30.40258692382372

-163.69°

S	6.95110741178645	3.12510492282217	32.46516258063326
C	1.46250961426653	5.07278500594549	19.89171784059950
H	1.57634724256754	6.14054619097517	20.03481095800182
O	1.32584934098413	5.54926198684043	22.93989928290796
O	6.56493742607088	3.62072179095183	33.80564171765504
C	1.76473134798381	2.99854284254477	19.17762039755841
H	2.22035135240531	2.19990991219018	18.60531244994550
O	8.41233734934325	2.92402955515274	32.32098292070305
C	0.72132731858795	2.99088871715446	20.05964144565815
H	0.09158915805256	2.18422651878261	20.41438413126229
O	6.15302722772068	1.96385309434973	32.01572751483685
C	-0.39396548227456	4.74149961798753	21.52172121221588
H	-1.28851955119051	4.11082772201468	21.46165139117344
H	-0.67472131677204	5.77774347832658	21.29848843909417
C	0.21898425060230	4.63315829361048	22.90640474989724
H	-0.53454796629564	4.90950773613933	23.66007281277491
H	0.56858798916080	3.60747602237872	23.09987375310167
C	2.16367707503526	5.52359280351736	24.01089517352297
C	3.28413308940932	6.36966638264573	23.92735618384297
H	3.42211423214386	6.97747723172269	23.03297507138434
N	2.21062090709107	4.30524111370353	19.08914487940588
C	3.31217740203280	4.77742259071286	18.24830416461986
H	4.22086969478119	4.22137416069945	18.50401502779814
H	3.46247728892142	5.84439030936319	18.43664796538032
H	3.05855971750967	4.61535125986658	17.19450462027532
N	0.54569769743181	4.29431354823820	20.48782376560547
N	4.96088777222083	5.75750221059569	27.13431292059295
N	4.90518045720258	4.89963593072687	28.07268074349859
C	4.18503945499558	6.41946453321161	24.97773252957899
H	5.05748606494918	7.07275281863886	24.93144384639984
C	3.99906435087765	5.61955087898063	26.12398991080083
C	2.86741449097992	4.78409827098718	26.20224759301442

H	2.70612350547231	4.18844690273436	27.10019771617368
C	1.95141404355923	4.73634315378033	25.15948501561657
H	1.07056394315532	4.10237999405933	25.24658031513134
C	5.61786048207044	5.24858267753688	29.23746808923738
C	6.00644764376519	6.56389589057588	29.55455486333713
H	5.76222293982584	7.37273602075314	28.86697485153148
C	6.66869454767769	6.81495297014266	30.75431675351081
H	6.95757778498477	7.83540452598130	31.01076912636374
C	6.95351631557986	5.77257378555522	31.64247357534036
H	7.46121892208719	5.97525109229054	32.58575360595859
C	6.55699540506824	4.46572687364170	31.32841924980967
C	5.88347401427407	4.20279925378482	30.13947569773923
H	5.56034804389785	3.19010940738598	29.89874314650708

-178.94

S	3.22082307119084	2.57045058713700	33.40970757992395
C	1.69885357835774	4.98247263486520	19.62373579678160
H	0.97269746384446	5.77186918988354	19.77753267230960
O	0.70171073061628	4.31778979219123	22.57890724796420
O	2.02768299446931	2.61609384364288	34.28758071437551
C	3.55113639236587	3.90127818742558	19.07384204611290
H	4.55611738787495	3.78191179726149	18.68832922289781
O	4.47893424517682	2.87409842829914	34.12831614994730
C	2.66721933611867	2.98975192991633	19.57796487384601
H	2.75085043861256	1.91986585543366	19.72486027304571
O	3.29442756573360	1.34447426265440	32.58575240563042
C	0.33001099266545	3.12196618339623	20.56226078289587
H	0.13509416176419	2.13478370123749	20.12677103428681
H	-0.51710233033146	3.78129048301989	20.33939792872658
C	0.53176625384514	2.98856142712984	22.06028307310917
H	-0.35617617319316	2.51017297947722	22.50191264045949
H	1.42041333488956	2.37804009374948	22.28334471655120
C	1.01986101326396	4.46483469680720	23.89343028892973
C	1.25827324921703	5.78226853109051	24.32183870238417

H	1.18177797506486	6.59582362810721	23.60021306998773
N	2.92661219926021	5.13500226782013	19.11120094789356
C	3.50867708932528	6.39965587598471	18.65890859330241
H	4.40715892096952	6.61345042023751	19.24870615928754
H	2.77089992596391	7.19447506354155	18.80156061345588
H	3.76880969452435	6.31709469175722	17.59768456066534
N	1.51725589992591	3.68372034326884	19.90854509045144
N	2.03161450162099	5.32464401947433	27.88225911558799
N	2.14884352565528	4.36214291862967	28.70287830618787
C	1.58719961107879	6.02331233278657	25.64646412782766
H	1.77756139865008	7.03923151711253	25.99561304102207
C	1.68785439303862	4.96271899560775	26.56873596536283
C	1.44181143804645	3.64716032618080	26.12974822238067
H	1.51170505668381	2.82529025016477	26.84122401886008
C	1.10936507810758	3.39476396020226	24.80598777724478
H	0.91587089100289	2.37204593558785	24.48711119580675
C	2.47201812243414	4.74098447789623	30.02679537071996
C	2.59951974715706	6.06762765919364	30.47982437111015
H	2.44254662808304	6.88913437400544	29.78229108921810
C	2.92416053878757	6.31004509136437	31.81193148314352
H	3.02515296168925	7.33687597643231	32.16699480278507
C	3.12465905346173	5.24908385831788	32.70309592360243
H	3.38514273311445	5.44569417647504	33.74344466986143
C	2.99449871385289	3.93074255099373	32.25085260298164
C	2.66710549234844	3.67524789971393	30.92145978957113
H	2.56778470367042	2.65099678452409	30.56252094149598

Isomerization of 10^{+}

5°

C	-5.79750116379685	1.76201688927161	-2.58670453306468
N	-5.87457264790583	1.95766449985434	-1.19600270678859
N	-5.59976005650439	1.06891963530569	-0.34306777015069
C	-6.10793973134369	0.54164409686137	-3.24508240822886
C	-6.04191686352007	0.48105742580887	-4.64003175251726

C	-5.65014729749735	1.61502926271331	-5.38247969469719
C	-5.36808805154448	2.83044685787477	-4.73171838192751
C	-5.47811188724400	2.91870428294512	-3.33931831024140
C	-5.08893964869152	-0.20177501238907	-0.60202641749045
N	-5.55397574236742	-1.29630883833536	0.06426343109717
C	-4.68062373749014	-2.35363321816172	-0.13893018552644
C	-3.63945552087375	-1.88075867641511	-0.90349738073939
N	-3.89791124131255	-0.54455129911222	-1.18028602319288
C	-3.03155667279257	0.35623323263420	-1.93976834414395
H	-1.99864780193920	-0.03257781576279	-1.90558386040118
H	-3.05229378905636	1.36170135319371	-1.48153072011429
H	-3.37602323873391	0.42632708964195	-2.99016327512978
C	-6.78611955911612	-1.30633042094372	0.85426168393870
H	-6.94243548181085	-0.28785880875427	1.25802410384120
H	-6.67487119878694	-2.02782579972234	1.68289864292031
H	-7.65301755300288	-1.59000464150990	0.22623906689887
H	-4.86358906731357	-3.34833969689467	0.27887959468285
H	-2.73106376073370	-2.37949050338700	-1.25555442632964
H	-6.31481590119763	-0.44855125668598	-5.16126736101445
H	-6.46155349316560	-0.33037369253530	-2.67546334372496
H	-5.30195223571208	3.86064979387707	-2.80006532619218
H	-5.08949942355091	3.71883520191553	-5.31719245101095
H	-5.59531723299487	1.55525005871189	-6.47971185075212

15.38°

C	-5.77345298497958	1.76396001072694	-2.60301496393604
N	-5.83457217950038	1.95767729615610	-1.21469250609655
N	-5.66530080390904	1.03438553431163	-0.36806481120836
C	-6.13227329779180	0.55492366279604	-3.25959922051048
C	-6.09237526899425	0.49645076857131	-4.65509508460502
C	-5.67873447289013	1.61978841804954	-5.40243653220205
C	-5.34499550272506	2.82377819320548	-4.75429258674230
C	-5.42521964514160	2.91065572506173	-3.36038928895541
C	-5.11310658768440	-0.21483345577788	-0.60478683075637

N	-5.54781992134015	-1.31529573124136	0.07671140838729
C	-4.63954951808148	-2.34582133795182	-0.10012665392769
C	-3.60774341297565	-1.85302834107419	-0.86532181484494
N	-3.90652337704746	-0.53209842034611	-1.17182263609887
C	-3.05222207309182	0.38055354914388	-1.92945931651412
H	-2.01658120470869	-0.00140069218388	-1.90339754478536
H	-3.07743187877460	1.38329395827201	-1.46502397367537
H	-3.40183596907129	0.45659681082256	-2.97783733892172
C	-6.78605949972389	-1.35066567202541	0.85533322865559
H	-6.97507063817904	-0.33157926387230	1.24366706993053
H	-6.66230318014250	-2.05788244035836	1.69447145455417
H	-7.63909370623308	-1.66671722616560	0.22370193565685
H	-4.79254835432867	-3.33849890513543	0.33423146107756
H	-2.67972531778984	-2.32785140093852	-1.19875870876915
H	-6.40346761663861	-0.42266183878717	-5.17357409996701
H	-6.50172611978672	-0.30744275870729	-2.68537493302304
H	-5.20730393813446	3.84456172470393	-2.82228849475975
H	-5.04811337662850	3.70404413721439	-5.34300212158448
H	-5.64655015370657	1.56120769552977	-6.50063709637768

25.75°

C	-5.74920254740117	1.76380685376445	-2.62788581732992
N	-5.78796942754288	1.94533811175923	-1.24115621960208
N	-5.71958496638412	0.99356679835923	-0.40767463887434
C	-6.14078333507140	0.56676769281163	-3.29024446376460
C	-6.13285159952445	0.52345437895447	-4.68616414526929
C	-5.71789997169338	1.64840219781444	-5.43120341199216
C	-5.34647973465652	2.83905992474915	-4.77831984546472
C	-5.39119401969759	2.91104981943628	-3.38271436106945
C	-5.12886455140414	-0.23485331377968	-0.60581559212025
N	-5.54193715212380	-1.33375331011286	0.09656466798037
C	-4.60263322326347	-2.34051311814807	-0.04163231827673
C	-3.57537124176217	-1.83840200443715	-0.80729733405189
N	-3.90622089846379	-0.53546888962366	-1.15431882924250

C	-3.06063792663971	0.37860263187619	-1.91838510339650
H	-2.02223545207867	0.00448895990988	-1.89048430727591
H	-3.09281811410011	1.38486372397183	-1.46134801091068
H	-3.41078799285870	0.44666504200335	-2.96735592099131
C	-6.78879169463353	-1.38439300140715	0.85911264527711
H	-7.00774056518607	-0.36243714221361	1.22390498788988
H	-6.65949350942379	-2.07164565055118	1.71388202528515
H	-7.62585238325506	-1.73329301003020	0.22331611352174
H	-4.73143405892206	-3.32547660080416	0.41742440037131
H	-2.62907736801814	-2.29471657362247	-1.11409981657922
H	-6.47038295798170	-0.38462014101162	-5.20749807246822
H	-6.50957220231256	-0.29616308011775	-2.71649529629228
H	-5.14209178930471	3.83431610954477	-2.83981797228761
H	-5.04669397685901	3.71982828035794	-5.36474452439125
H	-5.71309733943662	1.60162531054667	-6.53042883867443

36.13°

C	-5.72845963597690	1.76079972677273	-2.66250982784122
N	-5.74454111017299	1.91790730983840	-1.27705226586522
N	-5.76868712037505	0.94170081654890	-0.46373181031669
C	-6.13672890958672	0.57697907577410	-3.34159301439283
C	-6.15950921139187	0.56249443141165	-4.73717744815080
C	-5.75714009945346	1.70153143895745	-5.46872334931391
C	-5.36276823456808	2.87678926040018	-4.80069526617996
C	-5.37333151205682	2.91980026229904	-3.40405448419181
C	-5.14037650676683	-0.26380254414059	-0.60480507761170
N	-5.53541962182070	-1.35277648572234	0.12884347696866
C	-4.56728312544694	-2.33597129920909	0.04000130960726
C	-3.54264541839202	-1.83432114369067	-0.73005631222311
N	-3.90253330264269	-0.55537589575111	-1.13212725197009
C	-3.06757235838960	0.35143547647246	-1.91439671280659
H	-2.02708399253902	-0.01684529947976	-1.88790123424062
H	-3.10208456443813	1.36487710742334	-1.47258029560069
H	-3.42395447373633	0.40421004029043	-2.96250706287402

C	-6.79096853626818	-1.40950310917847	0.87477943814806
H	-7.03598778378505	-0.38266030802623	1.20858291645079
H	-6.66022893851650	-2.07113888991172	1.74934550064075
H	-7.61243437265386	-1.79168579016021	0.23761780004709
H	-4.67438374058587	-3.30662559093701	0.53381313056414
H	-2.57936091318845	-2.27531449295499	-1.00463640203108
H	-6.51107539028158	-0.33369857396964	-5.26969115530616
H	-6.49364352451376	-0.29677886415437	-2.77676994882746
H	-5.10443444930318	3.82966101836543	-2.84809115459175
H	-5.07047490946238	3.76714277931262	-5.37627554804832
H	-5.77858824368643	1.67726954341945	-6.56848795004252

46.50°

C	-5.71421043209356	1.75619642096483	-2.70678849823046
N	-5.71012385116429	1.87472707765910	-1.32270521828901
N	-5.81234378738419	0.87533243590627	-0.53708421742044
C	-6.12055119579400	0.58684727601571	-3.41521746782600
C	-6.16939425966707	0.61444283817456	-4.80911629125167
C	-5.79289561177473	1.78004862562810	-5.51340121878534
C	-5.39361328191186	2.93840689103275	-4.81814169900054
C	-5.37484594271744	2.93879987720255	-3.42182699576329
C	-5.14716665328849	-0.30309186349620	-0.60198520452374
N	-5.52734695435022	-1.37197843926178	0.17468927994809
C	-4.53249137856226	-2.33032743114799	0.14619474427032
C	-3.50860640176528	-1.83950882367176	-0.63262852568159
N	-3.89479739559438	-0.59302290301276	-1.10495260851667
C	-3.07495061586151	0.29481841105258	-1.92194154036797
H	-2.03282974019258	-0.06914268412051	-1.90154321707606
H	-3.10679037004205	1.32038210658441	-1.50743882014886
H	-3.44618871922916	0.32032172158352	-2.96640879617466
C	-6.79152293945788	-1.42540500712896	0.90387606352424
H	-7.06138292288230	-0.39107019796282	1.19329087535248
H	-6.66168018599789	-2.05006592106045	1.80541720128801
H	-7.59702457598975	-1.84716671530056	0.27108177008065

H	-4.62028183349174	-3.27848378038454	0.68545517014356
H	-2.52948256076988	-2.26751490282811	-0.86902640558804
H	-6.52045349220181	-0.26917572748230	-5.36261410493616
H	-6.45519822411690	-0.30745160330614	-2.86908534667191
H	-5.09993796845673	3.83280712061092	-2.84349377625119
H	-5.11897513813446	3.84747674398663	-5.37272711473618
H	-5.83661356710700	1.78889845376293	-6.61275803736739

56.88°

C	-5.70758799347643	1.75189003497988	-2.75532177202566
N	-5.68310813279353	1.82228442999092	-1.37387640302228
N	-5.84472403529697	0.79836433298286	-0.62285375889756
C	-6.10210160329478	0.59862445483621	-3.49974834375739
C	-6.17184529986504	0.67369731347231	-4.89014282812580
C	-5.82660540291168	1.87066151637405	-5.55839044405474
C	-5.43365065972226	3.01121808313377	-4.82951282112343
C	-5.39084301731191	2.96360971193378	-3.43549745303679
C	-5.14720428517272	-0.34795299886279	-0.59795787064491
N	-5.51762838875338	-1.38950267967240	0.22599044508553
C	-4.49969201718343	-2.32209826635133	0.26200466692745
C	-3.47404267589756	-1.84916948346974	-0.52641261816437
N	-3.88119997581630	-0.64100711952208	-1.07401691947714
C	-3.07803890220630	0.21862926524003	-1.93416777236084
H	-2.03481061778808	-0.14245808133837	-1.92064666877185
H	-3.10441815445992	1.25869660985898	-1.55551854866343
H	-3.46681363068671	0.20904372430088	-2.97311763199731
C	-6.79114586276180	-1.43348578245566	0.93668692986638
H	-7.08535602506905	-0.39144044664458	1.17044796648802
H	-6.66526656696524	-2.01085404372543	1.86987903943305
H	-7.57933253529506	-1.90007952225985	0.31335019296145
H	-4.57246292891577	-3.24178743096487	0.85054686914392
H	-2.48081710903112	-2.26536747776436	-0.72138603859916
H	-6.51413691149685	-0.19635150888373	-5.47001830659182
H	-6.41119495519188	-0.31917038055578	-2.97785294174621

H	-5.11796685396721	3.84031193173425	-2.83034124639111
H	-5.18117751945510	3.94250785521093	-5.35706116044538
H	-5.88852793921344	1.91728595842206	-6.65594456200849

67.25°

C	-5.71386514909942	1.74786204453792	-2.80634830958262
N	-5.67130856358000	1.76282568153325	-1.42945232476181
N	-5.86545255442685	0.70902484226388	-0.72117062794850
C	-6.09049657602403	0.61424887897173	-3.59288818505858
C	-6.16961910727529	0.73974425565712	-4.97821304982430
C	-5.85213295816863	1.96874330553853	-5.60217335621336
C	-5.47519128742695	3.08944885616240	-4.83358902565127
C	-5.42053775858302	2.99122096448814	-3.44367425031219
C	-5.14057240506800	-0.39701657140954	-0.59208436762569
N	-5.50484017572156	-1.40474687427197	0.28145819727609
C	-4.46712755186051	-2.31045001930556	0.38238903347432
C	-3.43739893020474	-1.85919878290154	-0.41409394280916
N	-3.86179044346416	-0.69555914399242	-1.03928069045739
C	-3.07979232235206	0.12647913504123	-1.95156573680078
H	-2.03679433096717	-0.23550633658556	-1.95148620482182
H	-3.09408758112499	1.18181280211767	-1.61522816169588
H	-3.49395884635782	0.07747752545753	-2.98018427397533
C	-6.78731300314262	-1.43504656596373	0.97360404194467
H	-7.10428772089927	-0.38719753957870	1.14459542485744
H	-6.66709964596231	-1.95722551267164	1.93962857100222
H	-7.55826613503352	-1.94897386269229	0.36583489761214
H	-4.52860936154059	-3.19689226659036	1.02105446824840
H	-2.43126302198570	-2.26241674235193	-0.56521569362593
H	-6.49760777489838	-0.11405480887654	-5.58961716334938
H	-6.37888661141674	-0.32648377580127	-3.10057326123320
H	-5.15687174066241	3.84851245054895	-2.80746070021814
H	-5.24320810286939	4.04373756249059	-5.32840126415109
H	-5.92332033988349	2.05573049818408	-6.69674404429876

77.63°

C	-5.73579725317608	1.74831906892268	-2.86307523508907
N	-5.67357035180408	1.70368798578596	-1.48994082000408
N	-5.85134347940214	0.60952439156475	-0.83960116044370
C	-6.07827923062699	0.63548600585019	-3.69531984982067
C	-6.15857367011429	0.81375637598344	-5.07387363918302
C	-5.88172456194990	2.07837710934211	-5.64562496392818
C	-5.54322195338747	3.18008077246407	-4.83211748303177
C	-5.48492045824988	3.02761672914001	-3.44775824859555
C	-5.11772111201058	-0.45107766979497	-0.58660133667999
N	-5.48821431505014	-1.41356586255198	0.33841763617282
C	-4.44069883936290	-2.29995721943433	0.50693823249133
C	-3.40017810375660	-1.88231807979492	-0.29358671667490
N	-3.82821278408623	-0.76662668804585	-0.99933841660940
C	-3.06125478535959	0.00617782056553	-1.96392559186447
H	-2.02250965503327	-0.36758742072452	-1.97744171087285
H	-3.05206545320020	1.07609900627979	-1.67511683623614
H	-3.50123789217950	-0.08270469236331	-2.97956110810613
C	-6.78104893293015	-1.41807947804471	1.00846105423861
H	-7.11204674915392	-0.36556295024901	1.11332266880214
H	-6.67356757605005	-1.88122453244862	2.00573311094828
H	-7.53827629637477	-1.97420132411158	0.42044014677542
H	-4.50350467100361	-3.14982184996355	1.19330958429521
H	-2.38507433513835	-2.27835554922579	-0.39570565122921
H	-6.45653869756152	-0.02425829366129	-5.72140423878929
H	-6.33675714519106	-0.33090547770664	-3.23707141964170
H	-5.24683648802813	3.86515885057991	-2.77608726781255
H	-5.34293367488864	4.16033025559282	-5.28844829726343
H	-5.95559153492962	2.20773271604979	-6.73590244184765

88.00°

C	-5.87214070916330	1.77957048833130	-2.97631219719343
N	-5.82013784059335	1.64507906970726	-1.58362875789692

N	-5.50546513822237	0.53611810423323	-1.08438164654953
C	-5.77560790315983	0.70064869247186	-3.89779000078661
C	-5.84915614419829	0.96544254764165	-5.26476836541319
C	-6.01578976869752	2.29347480373655	-5.72147372703068
C	-6.12757369709158	3.36050782013048	-4.80996201413247
C	-6.07273396706287	3.10721710222928	-3.43632513070316
C	-4.93366100338022	-0.50899219121300	-0.59456704089472
N	-5.44045898535258	-1.32090227292755	0.41572346339369
C	-4.53079572315030	-2.32975611149766	0.68055930355593
C	-3.41815610093715	-2.11319400557563	-0.10133892262736
N	-3.68232191536157	-1.02997540135973	-0.92411535261894
C	-2.78190989385929	-0.41232365161994	-1.88627287129355
H	-2.06906495139931	-1.17266640691630	-2.25250650725944
H	-2.21862445536620	0.42866102408381	-1.43346387445415
H	-3.37298047580889	-0.02647901731203	-2.74074678649630
C	-6.74785060527806	-1.12287343624024	1.02583750158732
H	-6.98089719198916	-0.04061482661638	1.00498620182021
H	-6.71423104538527	-1.47214738799409	2.07364992842397
H	-7.53977467448488	-1.67352142167025	0.47916748699377
H	-4.73859663099821	-3.12290109844795	1.40530305744837
H	-2.45888260321765	-2.63950458251344	-0.12293231594817
H	-5.79198701156468	0.14086006279660	-5.99071959077157
H	-5.68327308324986	-0.33154535762884	-3.52748677104151
H	-6.16750995804804	3.90753025965033	-2.68806016683418
H	-6.26724109932221	4.38767369308517	-5.17678803019642
H	-6.07487742365712	2.49071350143551	-6.80246687308090

90.00°

C	-5.880808	1.769005	-2.992699
N	-5.833984	1.640053	-1.598628
N	-5.488205	0.542680	-1.095883
C	-5.747655	0.690756	-3.909937
C	-5.820700	0.949226	-5.278194
C	-6.022698	2.270524	-5.740015

C	-6.170129	3.336538	-4.832513
C	-6.115906	3.088977	-3.457716
C	-4.923806	-0.497651	-0.589391
N	-5.442171	-1.303114	0.420621
C	-4.536827	-2.311635	0.700693
C	-3.414908	-2.099832	-0.069303
N	-3.668516	-1.020944	-0.900597
C	-2.756517	-0.408868	-1.855375
H	-2.050356	-1.175242	-2.222134
H	-2.186555	0.424034	-1.396085
H	-3.340192	-0.012416	-2.710013
C	-6.757080	-1.102240	1.013579
H	-6.991061	-0.020492	0.983360
H	-6.735840	-1.444503	2.064031
H	-7.541747	-1.657521	0.461112
H	-4.754037	-3.101238	1.426556
H	-2.456095	-2.627362	-0.077540
H	-5.734913	0.124593	-6.001263
H	-5.626407	-0.337002	-3.535737
H	-6.237365	3.888585	-2.712573
H	-6.336601	4.358275	-5.203295
H	-6.080622	2.462913	-6.821942

90.76°

C	-5.837893	1.589116	-3.082581
N	-5.885043	1.316719	-1.737330
N	-6.123464	0.381531	-0.958132
C	-5.969166	0.536206	-4.012125
C	-5.927434	0.828211	-5.371433
C	-5.739348	2.139274	-5.810433
C	-5.602707	3.173604	-4.881356
C	-5.657968	2.912525	-3.518240
C	-5.173673	-0.482390	-0.488410
N	-5.481332	-1.384279	0.462742

C	-4.361207	-2.128231	0.742959
C	-3.355825	-1.657778	-0.045476
N	-3.869823	-0.631091	-0.808474
C	-3.115921	0.152543	-1.782946
H	-2.078716	-0.199292	-1.771442
H	-3.140527	1.216612	-1.516125
H	-3.532946	0.017147	-2.790230
C	-6.799714	-1.542030	1.067889
H	-7.364518	-0.615180	0.919437
H	-6.678102	-1.734216	2.140323
H	-7.335226	-2.375796	0.594959
H	-4.365373	-2.927269	1.480145
H	-2.316851	-1.968519	-0.123618
H	-6.053561	0.020132	-6.095737
H	-6.144635	-0.483254	-3.658544
H	-5.568689	3.712959	-2.781676
H	-5.462327	4.200689	-5.225278
H	-5.709712	2.358158	-6.879745

90.76*°

C	-5.96274514683146	1.70028728486977	-3.04445211356362
N	-6.05717866701658	1.56553125005089	-1.63992108630787
N	-5.55668717958341	0.54381805789778	-1.11700889294675
C	-5.60532934818425	0.65383912567272	-3.91767266693552
C	-5.57419440343629	0.89467365270005	-5.28597397647552
C	-5.89094680644200	2.16784357580763	-5.78624436129423
C	-6.26128900377593	3.20155101448830	-4.91700140086487
C	-6.31535649640351	2.96694697581712	-3.54594013822227
C	-4.95601267273625	-0.44613782771252	-0.57214840902268
N	-5.46976317728794	-1.27335756846557	0.41780154882372
C	-4.53658534803920	-2.22621366151181	0.73813315261349
C	-3.39272409524765	-1.94980983759261	0.02634288279756
N	-3.66348435919351	-0.90070705571818	-0.81528486680440
C	-2.72822127816615	-0.23820237616127	-1.71230817906635

H	-1.95116926313969	-0.95651353905538	-1.99122161792334
H	-2.27284152970527	0.63278076859713	-1.22329840647155
H	-3.27176537510107	0.09369973341266	-2.60640019590981
C	-6.83251197453346	-1.17362447684668	0.92022381481929
H	-7.12207223917266	-0.11587229199842	0.91735566102736
H	-6.85406105338901	-1.56803756939993	1.94102606206181
H	-7.52319402915920	-1.74370007714920	0.28523972083308
H	-4.75118478543379	-3.02297939562048	1.44050208334986
H	-2.41278287440349	-2.41051841137540	0.06922439606418
H	-5.31100134651540	0.09030899532494	-5.97369902862774
H	-5.39215270170368	-0.33942188637045	-3.52142166037893
H	-6.61035605496321	3.74676737452700	-2.84261633708616
H	-6.51378925739517	4.18569278949922	-5.31240430824509
H	-5.86230053304056	2.34745637631266	-6.86170867624359

92.00°

C	-5.88802291404961	1.75825456018570	-3.00779977995865
N	-5.84790617491557	1.63450617776947	-1.61272067230567
N	-5.48015882715243	0.54697822569366	-1.10501788681705
C	-5.72553383284538	0.68047404980506	-3.92053571199957
C	-5.79596199527505	0.93302324746916	-5.29006875142766
C	-6.02425983196175	2.24805649456756	-5.75739655855405
C	-6.20052768727061	3.31333104691778	-4.85421086616026
C	-6.14888130580988	3.07131057998778	-3.47828929047061
C	-4.91853350615225	-0.48731067919171	-0.58419531428404
N	-5.44457091119584	-1.28801352968518	0.42594385833189
C	-4.53988424799829	-2.29304321607315	0.72019459333634
C	-3.41080987043966	-2.08258782721528	-0.03973557873411
N	-3.65862512060096	-1.00889741234372	-0.87926647160575
C	-2.73865901293797	-0.40100487992768	-1.82906226425361
H	-2.03335930014184	-1.17040738843833	-2.19116241707504
H	-2.16845485601072	0.43035954518908	-1.36738180213663
H	-3.31657388852517	-0.00302446099275	-2.68687347126474
C	-6.76633024786083	-1.08827993747773	1.00402966858826

H	-7.00487021166425	-0.00796053074308	0.96262583296889
H	-6.75399351795874	-1.42145399710750	2.05755933523275
H	-7.54287214498441	-1.65194688527610	0.44859628027538
H	-4.76254904215870	-3.07971139108182	1.44760403883350
H	-2.45085190565020	-2.60808743102178	-0.03551406061878
H	-5.68733726391667	0.10831143380341	-6.00997950234446
H	-5.58330105000027	-0.34305364177371	-3.54221851619948
H	-6.29223295537585	3.87040912700453	-2.73650504081023
H	-6.38708836759396	4.33004493772112	-5.22923110719785
H	-6.07955000955299	2.43582378223521	-6.84026854334871

102.25°

C	-5.88949113035977	1.75628098759085	-3.01529578448914
N	-5.84153127346107	1.62511370875847	-1.62129223101767
N	-5.45493823353518	0.53961027292298	-1.12404423822366
C	-5.71226037898053	0.68715355511135	-3.93519148266912
C	-5.79193909275363	0.94673212427109	-5.30277442255463
C	-6.04435627927270	2.26061225270496	-5.76112676376401
C	-6.23439265526145	3.31720706657765	-4.85087355238223
C	-6.17294338967246	3.06785987952388	-3.47663066018156
C	-4.90407048140422	-0.49023623258305	-0.58481698981216
N	-5.44097025473865	-1.28104758907473	0.42786101379708
C	-4.54587225729712	-2.29178957346554	0.73144630009766
C	-3.41091682683563	-2.09330773053368	-0.02299327462797
N	-3.64576771429211	-1.02228941501594	-0.86905083407253
C	-2.71729003491486	-0.42363649746605	-1.81633985663317
H	-2.01964995112609	-1.20005003594610	-2.17840625778087
H	-2.13908748761996	0.40102658307308	-1.35263178826466
H	-3.29048925569730	-0.01805306132753	-2.67373761318535
C	-6.76617389327589	-1.07135304549211	0.99476563997598
H	-7.00324994722775	0.00839161305777	0.93744863286140
H	-6.76135668634292	-1.38981699648286	2.05293716907782
H	-7.53969790543389	-1.64198842810303	0.44214691522561
H	-4.77779161610630	-3.07407472436560	1.46069148858769

H	-2.45539473831361	-2.62670458729871	-0.01055971469533
H	-5.67108177487572	0.12856663651211	-6.02814844925816
H	-5.54953014090482	-0.33557966320184	-3.56313696672829
H	-6.32551113728141	3.86036267373713	-2.72966417283548
H	-6.43894825824766	4.33302638605107	-5.21886875138938
H	-6.10699720476703	2.45408384046438	-6.84259335505778

112.50°

C	-5.89460128349807	1.75023321734967	-3.02499364529391
N	-5.84530144595020	1.60837670045377	-1.63230646402097
N	-5.45778333647299	0.51754189794488	-1.14674740853205
C	-5.71521762730758	0.68862109865287	-3.95327343951153
C	-5.79308464422053	0.95935032878126	-5.31877500026734
C	-6.04622567781384	2.27669962773939	-5.76663722702261
C	-6.23884958301289	3.32579220127965	-4.84801795865082
C	-6.17920954164132	3.06524501345532	-3.47578410297558
C	-4.90261643248576	-0.49777780090605	-0.58498601266510
N	-5.43803339367886	-1.27990302403047	0.43495667031399
C	-4.53946905000633	-2.28381290564760	0.75135383997884
C	-3.40384083047696	-2.08843817142160	-0.00296151516212
N	-3.64120595724742	-1.02694603244957	-0.86012874189844
C	-2.71520007695575	-0.43731946479520	-1.81515522166988
H	-2.01435858813847	-1.21550181370698	-2.16703787258229
H	-2.14045575815847	0.39609900915378	-1.36295380733378
H	-3.29064609294719	-0.04523795338509	-2.67741930004803
C	-6.76576845444979	-1.07152677182799	0.99663634457194
H	-7.00875471091135	0.00628304600025	0.92990126233566
H	-6.76207842619872	-1.38043925353588	2.05763724432289
H	-7.53504046822341	-1.65118585048653	0.44743846297103
H	-4.77053838454012	-3.05838542180087	1.48902724732888
H	-2.44584813286993	-2.61720132975432	0.01553404741327
H	-5.67054460271977	0.14738709070571	-6.05080945405888
H	-5.55185105881326	-0.33649752415987	-3.58823363099114
H	-6.33379459268662	3.85130135739633	-2.72244245958301

H	-6.44395179013298	4.34431379438515	-5.20813120203276
H	-6.10743005844110	2.47902893460997	-6.84657065493615

122.75°

C	-5.69737742900376	1.65642267486803	-3.06605083608437
N	-5.27986225462762	1.52767154969503	-1.75869709074425
N	-5.74063763060724	0.52342834301957	-1.08546478318734
C	-6.45353163939807	0.66806132010179	-3.76686270424649
C	-6.74198059339688	0.86158296026017	-5.11502386602080
C	-6.30843460465434	2.03533723231476	-5.77515343283987
C	-5.57549908167711	3.02323264030495	-5.08527403044197
C	-5.26995114460329	2.84178383370170	-3.73710759289117
C	-4.95111455294542	-0.38111748312352	-0.51155088425006
N	-5.47548081672611	-1.43429103361309	0.21318335379644
C	-4.45889938206309	-2.30652432397800	0.53869617716958
C	-3.28770643535473	-1.79418119008927	0.02015160602959
N	-3.58778496674196	-0.59392557035708	-0.60167305859736
C	-2.61070763832820	0.31417521245637	-1.19703980301133
H	-1.76997931264725	-0.27751027793423	-1.60286130741488
H	-2.23624968605045	1.03071344790350	-0.43977031024080
H	-3.10183301812887	0.89875915401103	-1.99616006566001
C	-6.88985194303501	-1.55656467978285	0.54682770944674
H	-7.47029760317019	-1.04125378180317	-0.24264260518391
H	-7.10828156271051	-1.07374586701169	1.51977399459599
H	-7.16434095221508	-2.62580501860173	0.59059794942431
H	-4.63759416216661	-3.22779348610998	1.10170166620034
H	-2.26979839714120	-2.19590873749965	0.04213292472414
H	-7.30577007389810	0.09909094656849	-5.67271251449315
H	-6.77055714915676	-0.23968453668018	-3.23527535273344
H	-4.70131643411436	3.58779430201919	-3.16298967684315
H	-5.24680806061436	3.93220326649645	-5.60961716257161
H	-6.54005347482282	2.17414910286339	-6.84201830393097

133.00°

C	-5.68693659988874	1.61107696982398	-3.13413607695254
N	-5.23042057791525	1.41009951698516	-1.84539159603817
N	-5.72806235199551	0.40856442366739	-1.19797603535490
C	-6.51910257246021	0.69302882898394	-3.84202332207712
C	-6.85742593615531	0.96218365135520	-5.16588895610436
C	-6.39414690492704	2.14180916344677	-5.79373939829553
C	-5.58127239214677	3.05871014009649	-5.09645779865655
C	-5.22617951421046	2.79990289033439	-3.77226831522404
C	-4.92744923474346	-0.43347560357200	-0.52968953503122
N	-5.45247332970605	-1.45588574843303	0.23116973365058
C	-4.42688848924636	-2.26686726033828	0.66695116135270
C	-3.24857302572371	-1.74503327102115	0.17480502586236
N	-3.55817475975613	-0.60391537109599	-0.54516223304862
C	-2.57662166237446	0.27748740025985	-1.17599989936571
H	-1.69554293649374	-0.32263602401917	-1.46755852240513
H	-2.27238966137724	1.08040011824440	-0.47661259756461
H	-3.03467654183553	0.75652037986698	-2.05963369836671
C	-6.87718465656355	-1.60714630357502	0.51203281109010
H	-7.43974633226891	-1.14953532828992	-0.32347833527485
H	-7.14720637153329	-1.08631335515208	1.45170403249411
H	-7.11975600715902	-2.68158193697053	0.59705078233309
H	-4.60485827907947	-3.15408310756864	1.28242775553457
H	-2.21916597401747	-2.10302869502353	0.27591362408073
H	-7.48289222775528	0.25391689602176	-5.72936164178631
H	-6.85556101483753	-0.22262399564345	-3.33674296590240
H	-4.59514611317203	3.49061245069264	-3.19407306345807
H	-5.22865406113567	3.97314753330816	-5.59509739707829
H	-6.66519247152118	2.34076563761564	-6.84164353841289

143.25°

C	-5.66742881049510	1.55681097327807	-3.20456156595219
N	-5.16342016757758	1.27784831316092	-1.94548029350342
N	-5.69566384736567	0.28546091665909	-1.31557039316257
C	-6.59550051307044	0.72814168442145	-3.90038846702993

C	-6.99099380746533	1.08079668623345	-5.18850182949990
C	-6.48591675888092	2.25585929512031	-5.79195466324996
C	-5.57380928864819	3.08268246590254	-5.10688665960248
C	-5.16193463312333	2.73829425182570	-3.81839303103793
C	-4.90006797646813	-0.49408385595828	-0.55691026050404
N	-5.42894494373222	-1.48143925694744	0.24059676023966
C	-4.40170496904965	-2.21522385010448	0.79329890325848
C	-3.21766403616618	-1.67919624410619	0.33069662359280
N	-3.52888994307185	-0.61055491682382	-0.49066204824370
C	-2.54694874863335	0.24648542484560	-1.15751043021034
H	-1.58288012345969	-0.29204704877765	-1.19153473237952
H	-2.42968234757122	1.20123903586424	-0.61165879411213
H	-2.89379075494389	0.47699376013020	-2.18052507643732
C	-6.86100651312071	-1.67217584702011	0.46176148791746
H	-7.39958307992768	-1.24033235798758	-0.40158729451776
H	-7.18263846106440	-1.15043691660067	1.38428380660449
H	-7.07565698382282	-2.75256252747265	0.54845105623481
H	-4.58245851995535	-3.06226741268619	1.46214716095330
H	-2.18277353966329	-1.98339764703167	0.51642484329438
H	-7.69445914817608	0.44195840066922	-5.74278632070937
H	-6.96336911898883	-0.18652044407599	-3.41564767884169
H	-4.45289488212365	3.35801253685978	-3.25039902964658
H	-5.18807568546175	3.99418090119661	-5.58602022975411
H	-6.80354239797196	2.52157367942551	-6.81156184370018

153.50°

C	-5.64601849028461	1.49886877893526	-3.27953476761650
N	-5.08699421891323	1.14594396081925	-2.05968023094163
N	-5.64164493204222	0.16293559261589	-1.43839915762889
C	-6.66783246162928	0.75914742020285	-3.94148562346697
C	-7.12457082344807	1.19109888012171	-5.18472737884712
C	-6.58474695899538	2.35652499999785	-5.77633819091558
C	-5.57679600801861	3.09371816292156	-5.12514522275065
C	-5.10422575432522	2.66841480683794	-3.88153062070361

C	-4.86147728650235	-0.54968860971636	-0.59016359772715
N	-5.40676157951691	-1.50107180469376	0.23519403659921
C	-4.39414968413023	-2.15642563676888	0.90208463110280
C	-3.20173693903660	-1.60666129627723	0.47881626939771
N	-3.49602196589835	-0.61137250325742	-0.43520684260798
C	-2.49844098393108	0.22183600205228	-1.11184819398315
H	-1.51739336463169	-0.27924323436736	-1.02806525487964
H	-2.45423330469079	1.22540582886471	-0.64960565648702
H	-2.77839111231296	0.34427070009641	-2.17296118089411
C	-6.84321113499525	-1.73347636965422	0.38514658535753
H	-7.34634612290391	-1.36876100617882	-0.52825312273793
H	-7.23319110610824	-1.17719483796077	1.25983323140010
H	-7.02365101935119	-2.81527034786116	0.51887996458261
H	-4.58981514561512	-2.96123680080769	1.61725496916456
H	-2.17123341402541	-1.85580291019164	0.75110093409744
H	-7.90422558936102	0.62213829506431	-5.71271934234115
H	-7.06301277083688	-0.14984806188962	-3.46783865403942
H	-4.31964520715573	3.21779761641650	-3.34103416228840
H	-5.16387256612061	3.99834097379424	-5.59449395539182
H	-6.95206005521832	2.68571140088411	-6.76015946545299

163.75°

C	-5.61426818826976	1.43214178379088	-3.35710352515930
N	-4.99135635749040	1.00102258653728	-2.19272227716372
N	-5.57459667977036	0.04708422221395	-1.55536170983320
C	-6.75854277530836	0.81430092649964	-3.93750631907236
C	-7.28182368820073	1.32595389260159	-5.12306967402756
C	-6.68482923373100	2.45056249402027	-5.73807978207326
C	-5.55404624706355	3.06606920119597	-5.16824530272666
C	-5.01541353026265	2.55953627867277	-3.98282590035082
C	-4.82022796218764	-0.60330592249412	-0.62986368338308
N	-5.38514941814300	-1.52705614974513	0.21074365737245
C	-4.39890385770412	-2.09602447639897	0.98787998666322
C	-3.20217711402204	-1.51984025755221	0.61563038723623

N	-3.46884844684360	-0.59675446718361	-0.37835807898132
C	-2.45598752329895	0.22841628194130	-1.04440287406882
H	-1.46120946571289	-0.16391966022857	-0.76748678760720
H	-2.55159415428759	1.28388291565420	-0.73168832387622
H	-2.59539899050815	0.17788043340121	-2.13881234453124
C	-6.81823737244408	-1.81747441252939	0.28006722495858
H	-7.28594810333889	-1.45699711366509	-0.65259685096229
H	-7.27216810212597	-1.29238071055430	1.14286689816563
H	-6.96300064269512	-2.90786111102820	0.38663345379525
H	-4.61498046863584	-2.86342315304090	1.73742795575626
H	-2.18544172845143	-1.70237198315558	0.97768380084744
H	-8.15992468268814	0.85254488837092	-5.58676845641358
H	-7.19997825984336	-0.06407645102651	-3.44749920399534
H	-4.13433070455186	3.01425550995090	-3.50723158019572
H	-5.09696427606014	3.93986553323578	-5.65478226613614
H	-7.10635202635951	2.84406892051586	-6.67540842423696

174.00°

C	-5.57964219765050	1.35481262459018	-3.43648348574508
N	-4.89462458496206	0.83732846697161	-2.34302756675969
N	-5.50945933749056	-0.06590758083187	-1.66434438141274
C	-6.86075042025887	0.91306366386350	-3.87279757015856
C	-7.44283721123331	1.50700496677926	-4.99052357181876
C	-6.76717763197474	2.53945603170754	-5.68133941189908
C	-5.50046544174492	2.98023569054775	-5.25464292672723
C	-4.90427123923531	2.39073801167178	-4.13674385732707
C	-4.78181158208073	-0.65557061892203	-0.67441433420485
N	-5.36253621825666	-1.56474877575415	0.17025896418134
C	-4.41279789750433	-2.03914305318277	1.04939565572903
C	-3.22227498669080	-1.41565019417223	0.73880471530452
N	-3.45646965031173	-0.56291580844432	-0.32302224587882
C	-2.43673247574184	0.28251444302663	-0.95404521630104
H	-1.46306242846943	0.04021084013602	-0.49225758487014
H	-2.67816281904159	1.34950798102198	-0.80231820795036

H	-2.40463192164363	0.08582636139413	-2.04031563729263
C	-6.77824756730089	-1.93954237929487	0.14859246564543
H	-7.21935146671889	-1.55396822381191	-0.78610379924101
H	-7.30030875201693	-1.49159148812301	1.01604928320654
H	-6.86510982880557	-3.04096405793568	0.18667689008464
H	-4.64778939859139	-2.77818111672152	1.82138920268923
H	-2.23026817849935	-1.51848569899180	1.18999325682878
H	-8.43066321168290	1.17233510900653	-5.34028821985818
H	-7.36572036427054	0.10699399278374	-3.32381583567621
H	-3.91647581265094	2.70905339718240	-3.77344457992086
H	-4.98287899012181	3.78346227869243	-5.79878637166348
H	-7.23717838504886	3.00022513681066	-6.56332562896340

179.61°

C	-6.03678614164972	2.85653407025202	-0.09323202467712
N	-5.21162714090392	1.75003120349037	0.07338025250748
N	-5.74543909619220	0.60381749454202	-0.16160415387572
C	-5.42853587024076	4.10990450232169	0.18680588344276
C	-6.16618736393891	5.28920053289428	0.05264208572719
C	-7.50976418136348	5.22560104637591	-0.36166496168377
C	-8.12042354060894	3.98147663442304	-0.64287441991921
C	-7.39623019967730	2.79855625745511	-0.51202390856104
C	-4.92138921444836	-0.47079369480315	-0.00191327921986
N	-5.39022009612024	-1.74290921804515	-0.19919099863926
C	-4.36912541792512	-2.64263749173450	0.01929890743223
C	-3.24845115432589	-1.91282394766704	0.35651190694957
N	-3.59499032296339	-0.57537018194440	0.34166794308625
C	-2.67433257511732	0.52697643664282	0.64332215565916
H	-1.67932753101595	0.09114912023761	0.84308337876569
H	-3.03103377860413	1.08902509484865	1.52466305200680
H	-2.62403031662137	1.22171600444381	-0.21340721560155
C	-6.76407518720199	-2.08068224299255	-0.57895269439666
H	-7.32610236268584	-1.13889772361507	-0.69513298622447
H	-7.22706678367312	-2.70013531304481	0.21235635835490

H	-6.75827735074425	-2.63692234506511	-1.53527873205603
H	-4.51021502741493	-3.72325375706274	-0.07977212731656
H	-2.23501312736296	-2.24510417751407	0.60321317876985
H	-5.69997715243777	6.26132281918240	0.26899701275614
H	-4.37694494779779	4.11972662328560	0.50804614137064
H	-7.85065540938261	1.82200283056710	-0.72613840584414
H	-9.17145648488557	3.95057623710048	-0.96637668018756
H	-8.09258222469610	6.15298318542567	-0.46881566862572

6. References

[1] M. Hegelmann, J. Zuber, J. Luibl, C. Jandl, W. Korth, A. Jess, M. Cokoja, *Chem. Eur. J.* **2024**, e202402985.
[2] W. A. Herrmann, A. M. J. Rost, J. K. M. Mitterpleininger, N. Szesni, S. Sturm, R. W. Fischer, F. E. Kühn, *Angew. Chem. Int. Ed.* **2007**, *46*, 7301.
[3] M. Lee, U. H. Choi, S. Wi, C. Slebodnick, R. H. Colby, H. W. Gibson, *J. Mater. Chem.* **2011**, *21*, 12280.
[4] Z. Li, X. Yuan, Y. Feng, Y. Chen, Y. Zhao, H. Wang, Q. Xu, J. Wang, *Phys. Chem. Chem. Phys.* **2018**, *20*, 12808.
[5] P. Fatás, E. Longo, F. Rastrelli, M. Crisma, C. Toniolo, A. I. Jiménez, C. Cativiela, A. Moretto, *Chemistry* **2011**, *17*, 12606.
[6] C. Lin, L. Yang, M. Xu, Q. An, Z. Xiang, X. Liu, *RSC Adv.* **2016**, *6*, 51552.
[7] F. Schmidt, B. Zehner, M. Kaposi, M. Drees, J. Mink, W. Korth, A. Jess, M. Cokoja, *Green Chem.* **2021**, *23*, 1965.
[8] APEX4 Suite of Crystallographic Software, Version 2021-10.0, Bruker AXS Inc., Madison, Wisconsin, USA, 2021.
[9] Bruker, SAINT, V8.40B, Bruker AXS Inc., Madison, Wisconsin, USA.
[10] L. Krause, R. Herbst-Irmer, G. M. Sheldrick, D. Stalke, *J. Appl. Cryst.* **2015**, *48*, 3.
[11] G. M. Sheldrick, *Acta Cryst.* **2015**, *A71*, 3.
[12] G. M. Sheldrick, *Acta Cryst.* **2015**, *C71*, 3.
[13] C. B. Hübschle, G. M. Sheldrick, B. Dittrich, *J. Appl. Cryst.* **2011**, *44*, 1281.
[14] Ed. E. Prince, International Tables for Crystallography Volume C, Mathematical, Physical and Chemical Tables, International Union of Crystallography, Chester, England, 2006, 500–502, 219–222, 193–199.
[15] C. R. Groom, I. J. Bruno, M. P. Lightfoot, S. C. Ward, *Acta Cryst.* **2016**, *B72*, 171.
[16] D. Kratzert, FinalCif, V139, https://dkratzert.de/finalcif.html.
[17] F. Neese, *WIREs Comput. Mol. Sci.* **2012**, *2*, 73.
[18] F. Neese, *WIREs Comput. Mol. Sci.* **2025**, *15*.
[19] F. Neese, *WIREs Comput. Mol. Sci.* **2022**, *12*.
[20] R. A. Kendall, H. A. Früchtl, *Theor. Chem. Acc.* **1997**, *97*, 158.
[21] F. Neese, F. Wennmohs, A. Hansen, U. Becker, *Chem. Phys.* **2009**, *356*, 98.
[22] S. Grimme, J. Antony, S. Ehrlich, H. Krieg, *J. Chem. Phys.* **2010**, *132*, 154104.
[23] S. Grimme, S. Ehrlich, L. Goerigk, *J. Comput. Chem.* **2011**, *32*, 1456.
[24] E. C. Meng, T. D. Goddard, E. F. Pettersen, G. S. Couch, Z. J. Pearson, J. H. Morris, T. E. Ferrin, *Protein Sci.* **2023**, *32*, e4792.
[25] A. D. Becke, *Phys. Rev. A* **1988**, *38*, 3098.
[26] F. Weigend, R. Ahlrichs, *Phys. Chem. Chem. Phys.* **2005**, *7*, 3297.
[27] A. D. Becke, *J. Chem. Phys.* **1993**, *98*, 5648.
[28] C. Lee, W. Yang, R. G. Parr, *Phys. Rev. B* **1988**, *37*, 785.
[29] C. Bannwarth, S. Ehlert, S. Grimme, *J. Chem. Theory Comput.* **2019**, *15*, 1652.
[30] S. Ehlert, M. Stahn, S. Spicher, S. Grimme, *J. Chem. Theory Comput.* **2021**, *17*, 4250.
[31] R. Sure, S. Grimme, *J. Comput. Chem.* **2013**, *34*, 1672.
[32] M. Garcia-Ratés, F. Neese, *J. Comput. Chem.* **2019**, *40*, 1816.
[33] J. Tao, J. P. Perdew, V. N. Staroverov, G. E. Scuseria, *Phys. Rev. Lett.* **2003**, *91*, 146401.
[34] L. Wittmann, I. Gordiy, M. Friede, B. Helmich-Paris, S. Grimme, A. Hansen, M. Bursch, *Phys. Chem. Chem. Phys.* **2024**, *26*, 21379.
[35] F. Neese, *J. Comput. Chem.* **2003**, *24*, 1740.
[36] Y.-S. Lin, G.-D. Li, S.-P. Mao, J.-D. Chai, *J. Chem. Theory Comput.* **2013**, *9*, 263.
[37] S. Maeda, K. Ohno, K. Morokuma, *J. Chem. Theory Comput.* **2010**, *6*, 1538.
[38] T. Yanai, D. P. Tew, N. C. Handy, *Chem. Phys. Lett.* **2004**, *393*, 51.
[39] T. Lu, F. Chen, *J. Comput. Chem.* **2012**, *33*, 580.
[40] T. Lu, *J. Chem. Phys.* **2024**, *161*.
[41] M. Campetella, F. Maschietto, M. J. Frisch, G. Scalmani, I. Ciofini, C. Adamo, *J. Comput. Chem.* **2017**, *38*, 2151.
[42] C. Kollmar, K. Sivalingam, B. Helmich-Paris, C. Angeli, F. Neese, *J. Comput. Chem.* **2019**, *40*, 1463.
